# Phospha-bicyclohexene-germylenes exhibiting unexpected reactivity†

**DOI:** 10.1039/d4sc04034a

**Published:** 2024-07-30

**Authors:** Marie Sophie Würdemann, Steffen Kühn, Tobias Bötel, Marc Schmidtmann, Thomas Müller

**Affiliations:** a Institute of Chemistry, Carl von Ossietzky Universität Oldenburg Carl von Ossietzky-Str. 9-11 D-26129 Oldenburg Federal Republic of Germany thomas.mueller@uni-oldenburg.de

## Abstract

Introducing phospha-bicyclohexene (BCH)-germylenes (BCHGe's) as a novel, multifunctional compound class: the title compounds 15–18 are obtained from simple salt metathesis reactions of dipotassium germacyclopentadienediides K_2_[1] with phosphorusdichlorides. The BCHGe's 15–18 are stabilized by homoconjugation of the germanium(ii) centre with the remote C=C double bond. Despite substantial thermodynamic stabilization, phospha-BCHGe's are reactive and undergo a reductive elimination of elemental germanium to give the corresponding phospholes. The elimination is a nucleophilic, bimolecular process and is prevented by large substituents. The reaction of phospha-BCHGe's with small electrophiles gives the corresponding phosphonium salts. Oxidation with chalcogens takes place at both the germanium and the phosphorus atom, and after elimination of germanium chalcogenides the corresponding phosphole chalcogenides were isolated. The introduced germylenes exhibit strong nucleophilic but also non-neglectable electrophilic properties.

## Abstract 2

Featuring mostly nucleophilic but also electrophilic properties, phosphorus-bridged bicyclic germylenes require steric shielding to be protected from decomposition. The two nucleophilic sites, Ge and P, show competing reactivity.

## Graphical abstract

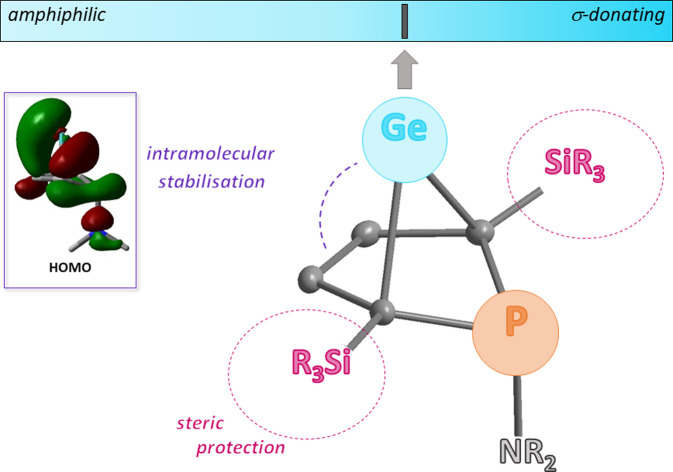

## Introduction

**Fig. 1:**
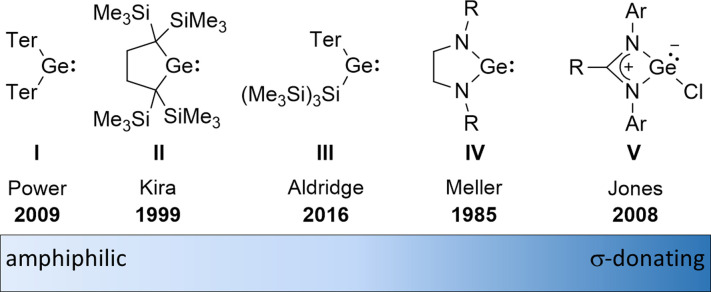
Literature-known germylenes exhibiting different properties (Ter = 2,6-dimesitylphenyl).^5–9^

The amphiphilic reactivity of heavy carbene analogues as well as the development of methods for their synthesis and the strategies to control their reactivity are in the focus of modern molecular main group chemistry.^1–3^ Germylenes are the outriders in this field due to the moderate strength of bonds between germanium and other elements and due to the relative stability of the formal oxidation state +II of germanium.^4^ For these reasons, germylenes are also attractive goals for catalyst design based on main group elements. The σ-donating and π-accepting properties of germylenes can be tuned applying different stabilisation strategies combined with sophisticated substituent design. This resulted in a variety of germylenes known today, ranging from amphiphilic to solely σ-donating (Fig. 1). The fine tuning of their reactivity enables their application for different purposes such as small molecule activation, bond activation in larger molecules and ligand design.

**Fig. 2:**
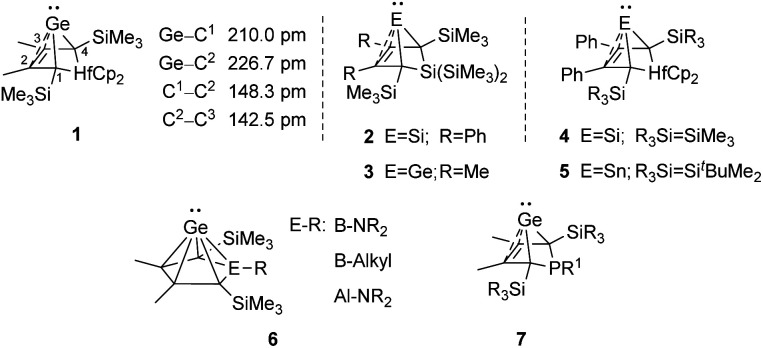
Bicyclohexene-type germylenes (BCHGe's) with different bridging groups and related silicon and tin compounds.

An alternative type of intramolecular stabilisation, previously only known for bicyclic boranes^10^ and group 14 element cations^11–16^ and applied for matrix isolation of silylenes,^17^ was introduced to the chemistry of stable germylenes by our group in 2016.^18,19^ Germylene 1, with the germanium centre integrated into a bicyclo-[2.1.1]-hexene (BCH) framework, is stabilised by through-space interaction of the germylene centre with the C=C double bond in the homoallylic position.^20^ This homoconjugation leads to a destabilization of the LUMO and to preferentially nucleophilic reactivity of BCHGe 1. Two closely related BCH silylenes (BCHSi's) 2 and 4 were reported shortly after.^21,22^ Their denotation as bicyclic tetrylenes was justified based on structural and NMR spectroscopic parameters and it was supported by the results of quantum chemical calculations.^21,23^ It contrasts with the interpretation of the related tin compound 5 as a Sn(0) butadiene complex by Saito and co-workers.^24^ The very different life times of hafnocena-BCHGe 1^18^ and the sila-BCHGe 2^22^ suggest already a remarkable influence of the second bridging group, the spectator group, of the bicyclic cage on the stability of the germylene. Recently, our group reported on boron- and aluminum-based germa[5]pyramidanes 6.^25–27^ Despite the close similarity of their topology to the BCHGe's 1 and 3, the inclusion of the electron deficient group 13 elements results in a quite different electronic structure of these *nido*-clusters. Bearing this significant effect of the spectator group in mind, we introduced here the electron-rich aminophosphanyl group into the BCH framework. This resulted in the formation of germanium(ii) compounds with two Lewis basic sides in close neighbourhood: phospha-BCHGe's 7 (Fig. 2).

## Results and discussion

**Scheme 1:**
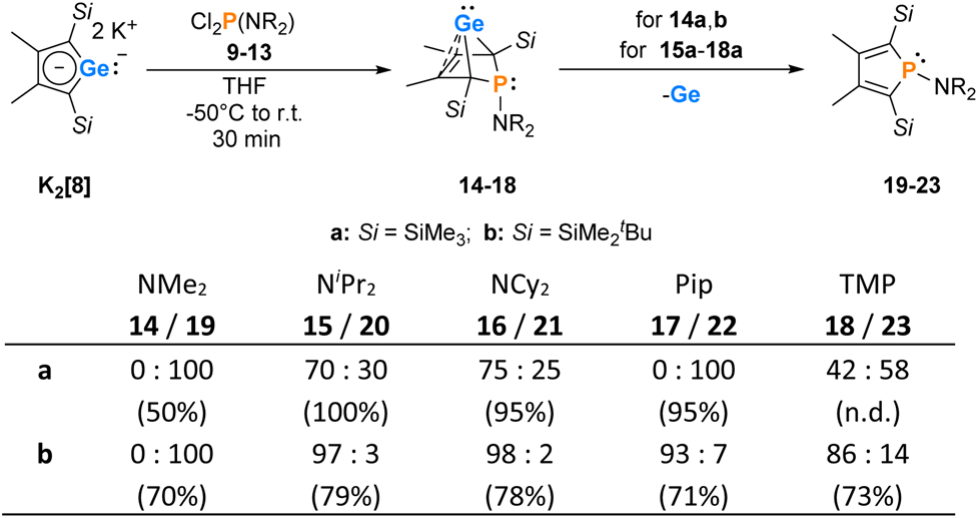
Synthesis of BCHGe's 15–18 and phospholes 19–23 (Pip = piperidyl; TMP = 2,2,6,6-tetramethylpiperidyl). The obtained molar ratio, applying standard reaction conditions, was determined by ^31^P{^1^H} NMR spectroscopy. The overall yield is given in parentheses.

The synthesis of aminophospha-**b**i**c**yclo**h**exene (BCH)-germylenes 15–18 was achieved by double salt metathesis reaction, using dipotassium germolediides K_2_[8] and amino-dichlorophosphanes 9–13 as starting materials.^28,29^ The ^*t*^BuMe_2_Si-substituted BCHGe's 15b–18b were isolated in yields between 71 and 79% (Scheme 1). In the case of the trimethylsilyl-substituted potassium germolediide K_2_[8a], the reactions were also selective, but the formation of elemental germanium was observed as a follow-up reaction, giving the corresponding aminophospholes 19a–23a as the final products (Scheme 1). These were isolated after filtration as pure materials. This kind of reductive elimination had previously been reported by our group for a related sila-BCH-germylene 3 (Fig. 2).^22^ The complete reaction sequence, shown in Scheme 1, describes a germole to phosphole transformation. In the case of phospha-BCHGe's 15a, 16a and 18a, the elimination process is slow, enabling their characterisation by NMR spectroscopy, although they cannot be isolated as pure substances. The elimination proceeds only in solution and is independent of the solvent.

**Fig. 3:**
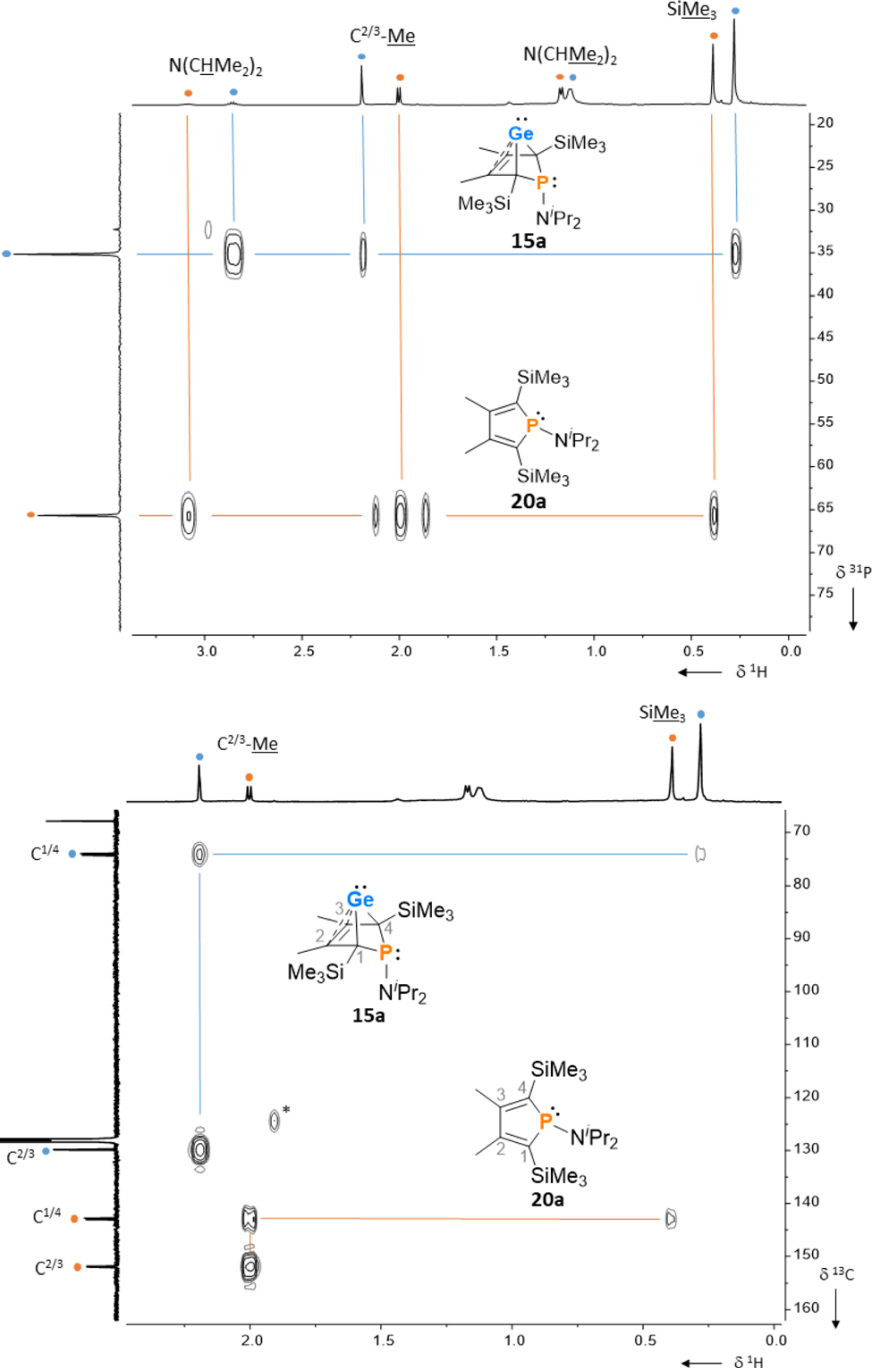
^1^H^31^P HMBC NMR spectrum (top) and ^1^H^13^C HMBC NMR spectrum (bottom) of a mixture of germylene 15a (•) and phosphole 20a (•), (500 MHz, benzene-d_6_, reaction mixture after exchange of solvent) *impurity.

After a standard reaction time of 30 min in THF, the germylene/phosphole mixtures were usually obtained in high yields with the germylenes being the predominant component (Scheme 1). We were not able to detect the BCHGe's 14a and 17a as the elimination of germanium was too fast. We assume the elimination of germanium from the BCHGe's 14–18 to be a bimolecular process, in the case of germylenes 14a–18a induced by the germylenes themselves. This is supported by the following observations: (i) dissolution of the pure crystalline material of germylene 15a immediately led to the formation of small amounts (3%) of phosphole 20a, increasing over time (see ESI: Fig. S13†) and (ii) exchange of the SiMe_3_ groups for SiMe_2_^*t*^Bu substituents enhanced the stability of the BCHGe's 15b–18b. In the latter cases, the elimination of germanium was not observed after their formation and the germylenes were isolated with only small contamination of the corresponding phospholes 20b–23b (see Scheme 1). Solely the NMe_2_ substituted germylene 14b eluded detection. In a test reaction, the germylene/phosphole mixture 15b/20b obtained under standard conditions was heated in toluene for several hours without any signs of decomposition of the germylene 15b. We attribute the increased stability of the SiMe_2_^*t*^Bu substituted germylenes to sterical factors, arising mainly from the bulkier silyl groups and, to a minor extent, also from the amino substituent. We presume that the small amounts of phospholes 20b–23b obtained during the synthesis result from the reaction of already formed BCHGe's 15b–18b with the nucleophilic germole dianion [8b]^2−^ (see ESI: Fig. S119†). Separation of the small amounts of phospholes 20b–23b (Scheme 1) from the corresponding germylenes 15b–18b by crystallization on large scales was not possible due to their similar high solubility in all tested solvents. The sensitivity of germylenes 15b–18b*versus* air and moisture excluded other separation techniques. Overall, seven different aminophospha-BCHGe's and ten different aminophospholes were identified by NMR spectroscopy (Scheme 1, Table 1 and see ESI†). The reaction sequence shown in Scheme 1 is very selective with germylenes and phospholes being the only products. For that reason, also the mixtures (Scheme 1) obtained with the trimethylsilyl-substituted germolediide [8a]^2−^ were analysed using NMR spectroscopy and both products, phosphole^30,31^ and germylene, were characterised. An exemplary analysis is shown for the 70 : 30 mixture of germylene 15a and phosphole 20a. This example demonstrates that BCHGe's, phospholes and their derivatives can clearly be distinguished by heteronuclear NMR spectroscopy. This feature is of importance for the mechanistic studies on the reactivity of the BCHGe's. The ^1^H NMR spectrum displayed two signals for each functionality: trimethylsilyl protons (Si**Me**_3_), isopropyl-methyl protons (N(CH**Me**_2_)_2_), backbone-methyl protons (C^2/3^–**Me**) and isopropyl-methine protons (N(C**H**Me_2_)_2_) (Fig. 3, *δ*^1^H axis), already indicating the presence of two compounds. Two signals were also displayed in the ^31^P{^1^H} NMR spectrum (Fig. 3, top, *δ*^31^P axis). The agreement between quantum mechanical predicted ^31^P NMR chemical shifts for DFT-optimized molecular structures of germylenes 14–18 and phospholes 19–23 and experimental ^31^P NMR chemical shifts supports our assignment (Table 1).^32,33^^1^H and ^31^P NMR spectra indicated the same molar ratio 15a : 20a = 7 : 3. Detailed characterisation of the products was enabled using 2D NMR spectroscopy. After assignment of the ^1^H NMR signals to the different phosphorus species, using ^1^H^31^P HMBC NMR spectra (Fig. 3, top), ^1^H^13^C NMR spectra were recorded to determine the structure of the backbone (Fig. 3, bottom).

**Table 1:** Selected NMR spectroscopic data of the synthesised germylenes 15–18 and phospholes 19–23, recorded in benzene-d_6_, and calculated ^31^P NMR chemical shifts (*italic*) (GIAO/M06-L/6-311+G(2d,p)//M06-2X/6-311+G(d,p))

	*δ* ^13^C : C^1/4^ (^1^*J*_C,P_ [Hz])	*δ* ^13^C : C^2/3^ (^2^*J*_C,P_ [Hz])	*δ* ^31^P	*δ* ^31^P calc.
**Germylenes**				
15a	74.4 (36)	129.9 (8)	35.4	*31*
16a	74.7 (36)	129.8 (8)	39.4	*37*
18a	81.9 (48)	134.1 (8)	30.8	*28*
15b	72.8 (41)	131.1 (7)	53.8	*48*
16b	73.0 (41)	131.1 (7)	57.7	*50*
17b	73.4 (37)	130.4 (8)	59.8	*50*
18b	81.6 (53)	135.1 (8)	39.7	—

**Phospholes**				
19a	143.6 (31)	153.4 (18)	94.3	*88*
20a	143.0 (33)	151.9 (19)	65.6	*56*
21a	143.0 (33)	151.6 (20)	63.8	*61*
22a	142.9 (32)	154.3 (16)	92.2	*85*
23a	142.7 (34)	147.0 (26)	54.2	*46*
19b	141.2 (31)	154.2 (17)	98.5	—
20b	140.7 (36)	154.0 (16)	71.7	*56*
21b	141^a^	154^a^	69.7	*60*
22b	140.2 (34)	155.1 (16)	95.9	*87*
23b	139.7 (38)	148.9 (23)	61.2	—

a Data extracted from the ^1^H^13^C HMBC NMR spectrum.

The ^13^C NMR data of the bicyclohexene and the phosphole carbon backbone are characteristic. Due to the correlation of the trimethylsilyl protons with the C^1/4^ carbon atoms as well as correlations of the methyl protons with the C^1/4^ and the C^2/3^ carbon atoms, triangular patterns are displayed in the ^1^H^13^C HMBC NMR spectra. The NMR chemical shifts of the C^1/4^ carbon atoms allow facile differentiation between the bicyclohexene and the butadiene (phosphole) backbone. The signals of the formally sp^3^-hybridised bridgehead carbon atoms (*δ*^13^C(C^1/4^) = 72.8–81.9; 15a: *δ*^13^C = 74.4) are shifted to lower frequency than those of the sp^2^-hybridised carbon atoms (*δ*^13^C(C^2/3^) = 129.8–135.1; 15a: *δ*^13^C = 129.9). The ^13^C NMR signals of the butadiene part of the phospholes appear at even higher frequencies (*δ*^13^C(C^1/4^) = 139.7–143.6; 20a: *δ*^13^C = 143.0; *δ*^13^C(C^2/3^) = 147.0–155.1; 20a: *δ*^13^C = 151.9, see Table 1). Comparison of the data, summarized in Table 1, suggests slight influence of the different silyl and amino groups on the electronic structure of the compounds 15–23. Interestingly, the exchange of the SiMe_3_ groups for SiMe_2_^*t*^Bu groups leads to a significant high frequency shift of the ^31^P NMR resonances of all phospholes and phospha-BCHGe's. The differences comprise about Δ(*δ*^31^P) = 9–18 for the germylenes with the same amino-substituent and about Δ(*δ*^31^P) = 4–7 for the phospholes with the same amino substituent.

**Fig. 4:**
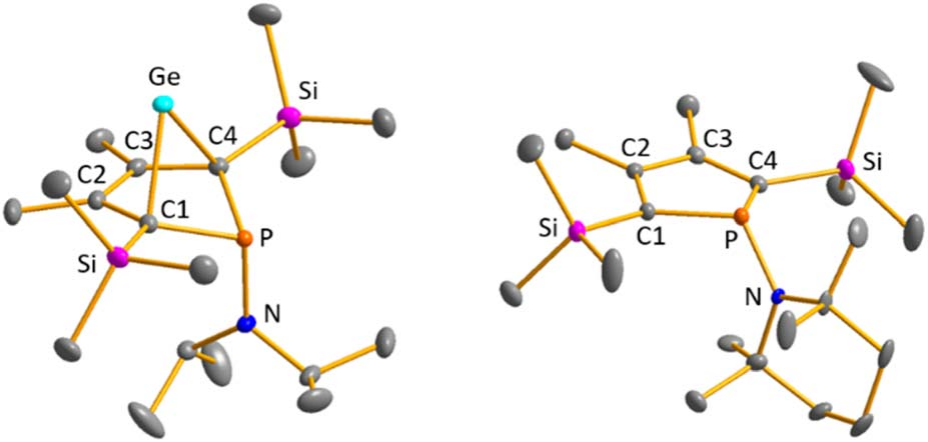
Molecular structure of phospha-BCH-germylene 15a (left) in the crystal. Thermal ellipsoids at 50% probability. Hydrogen atoms are omitted for clarity. Selected atomic distances [pm] and angles [°]: C^1^–C^2^ 146.53 (6), C^2^–C^3^ 142.33 (8), Ge–C^1^ 216.32 (4), Ge–C^2^ 219.64 (5), P–N 170.18 (7), ΣP 302.3, ΣN 360.0. Molecular structure of phosphole 23a (right) in the crystal. Thermal ellipsoids at 50% probability. Hydrogen atoms are omitted for clarity. Selected atomic distances [pm] and angles [°]: C^1^–C^2^ 138.31 (22), C^2^–C^3^ 145.28 (22), P–N 168.97 (21), ΣP 325.3, ΣN 359.1.

Colourless crystals of germylene 15a, suitable for single crystal X-ray diffraction (sc-XRD), were obtained upon recrystallization from pentane at −30 °C. In agreement with the NMR spectroscopic results, the structure solution revealed a bicyclohexene structure for germylene 15a. The molecule is symmetric, featuring a mirror plane spanned by the Ge, P and N atoms (Fig. 4, left). The C^2^–C^3^ bond (142.3 pm) is shorter than the C^1^–C^2^ bond (146.5 pm), but still longer than a typical C=C double bond (134 pm).^34^ The Ge–C^1^ bond of germylene 15a (216.3 pm) is long, elongated by 20 pm compared to the sum of the single bond radii (196 pm).^34^ The Ge–C^2^ separation (219.6 pm) is only slightly larger than the Ge–C^1^ distance, suggesting interaction of the C^2^–C^3^ double bond and the germanium atom. The C^1^–Ge–C^4^ angle in germylene 15a of *α*(Ge) = 70.3° is even more acute than that of hafnocena-BCHGe 1 (*α*(Ge) = 85.0°). Overall, the structural parameters of the GeC_4_ skeleton are very close to that of the related hafnocena-BCHGe 1 (Fig. 2).^18,19^ We therefore conclude that in both BCHGe's, 15a and 1, the same stabilising mechanism is operating. Germylene 15a is stabilised by homoconjugation which is delocalisation of π-electrons of the C^2^=C^3^ bond into the vacant germanium 4p-orbital. The P–C^1/4^ bonds (184.1 pm) are within the expected range of phosphorus–carbon single bonds (186 pm).^34^ The coordination sphere of the phosphorus atom is, as typical for tricoordinated phosphorus atoms, pyramidalised (Σ(P) = 302.3°). This underlines the lack of delocalisation of its lone-pair electrons into the backbone. The observed planarisation around the nitrogen atom in germylene 15a (Σ(N) = 360.0°) as well as the short P–N bond (170.1 pm, compared to 182 pm expected for a typical P–N single bond)^34^ can be assigned to negative hyperconjugation as studied and described by Haaland *et al.*^35,36^

Crystals suitable for sc-XRD analysis were obtained from phospholes 21a and 23a.^30,31,37^ Both molecular structures are very similar, and therefore, only that of the TMP-substituted phosphole 23a will be shortly discussed here (Fig. 4, right). Structural data for aminophosphole 21a are given in the ESI.† Phosphole 23a possesses a slightly folded five-membered ring with a flap angle of *α*(P) = 15.3°. The phosphorus atom is less pyramidalised (Σ(P) = 325.3°) as in the phospha-BCH-germylene 15a. The backbone consists of two slightly elongated C=C double bonds (C^1^=C^2^ = 138.3 pm) and a slightly shortened C–C single bond (C^2^–C^3^ = 145.2 pm), typical for localised butadiene groups. The localisation of the π-electrons and the pyramidalisation of the phosphorus atom correlate with the high s-character of the phosphorus lone pair. As shown for germylene 15a, the coordination sphere of the nitrogen atom of phosphole 23a is trigonal planar and the P–N single bond is shortened (P–N = 168.9 pm). In addition, the dihedral angle of the amino group to the phosphole ring is close to perpendicular (*α*(N) = 118.6°).

**Fig. 5:**
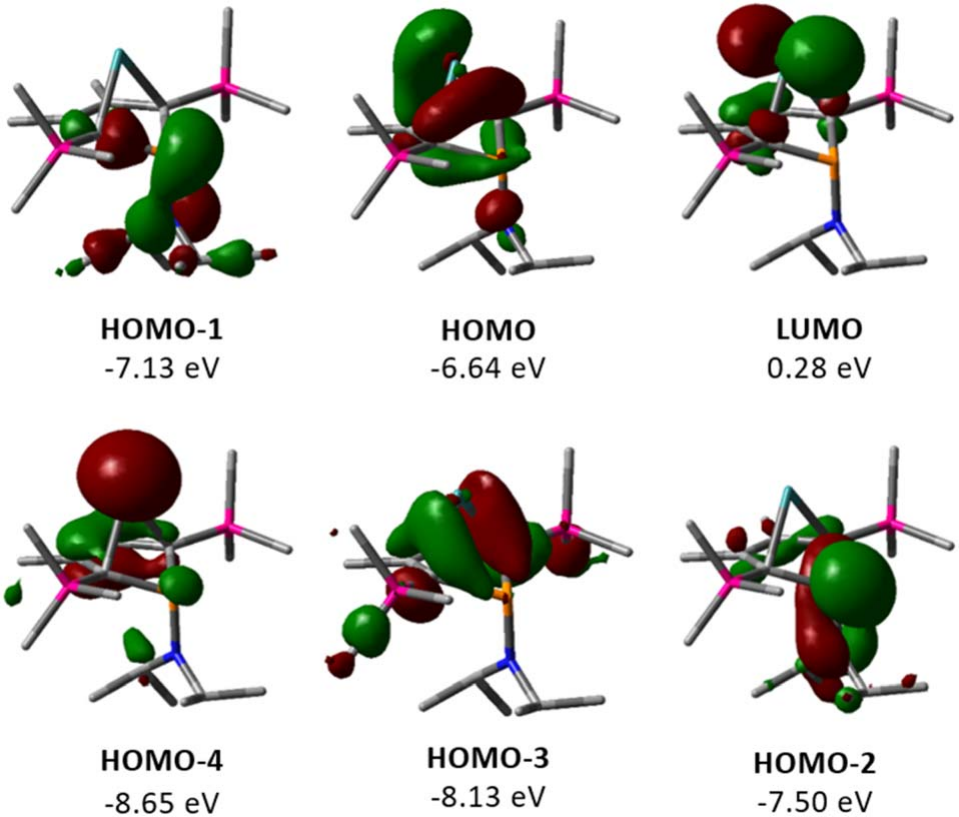
Selected frontier molecular orbitals of germylene 15a (M06-2X/6-311+G(d,p); isodensity value 0.04 a.u.).

The electronic structure of germylene 15a was further investigated using DFT calculations at the M06-2X/6-311+G(d,p) level of theory.^32^ The optimized molecular structure of germylene 15a differs only slightly from the experimental structures derived from the solution sc-XRD analysis. Fig. 5 displays selected molecular orbitals. The two frontier orbitals depict the interaction of the empty 4p(Ge) orbital with a filled π-orbital of the butadiene part of the molecule (homoconjugation). The HOMO shows the delocalisation of π-electrons from the butadiene system into the vacant germanium 4p-orbital. The LUMO is mainly the antibonding combination of these orbitals with large contribution from the 4p(Ge) orbital. HOMO−2 and HOMO−1 show contributions from the phosphorus and the nitrogen lone pairs, and from the antibonding P–C^1/4^ σ-orbitals, which indicates negative hyperconjugation.^35^ HOMO−3 displays the interaction of the two occupied σ-Ge–C^1/4^ bonds with the π*-orbital of the C^2^=C^3^ bond (σ–π*-hyperconjugation). HOMO−4 represents the lone pair at germanium as it shows large contributions from atomic orbitals of the germanium atom. This analysis of the molecular orbitals of germylene 15a identifies it as a carbene analogue. HOMO−4 and the LUMO are the orthogonal occupied and empty orbitals, typical for this class of compounds (Fig. 5).

**Fig. 6:**
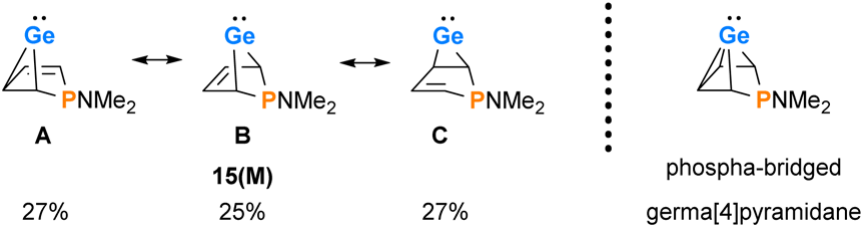
Dominant resonance structures A–C of 15(M) according to NRT computations (M06-2X/6-311+G(d,p)) and alternative representation of 15(M) as the *arachno*-type cluster.

The delocalized electronic structure of germylene 15a is also supported by the results of a natural bond orbital (NBO) analysis of the smaller model compound 15(M).^38–40^ The analysis reveals significant electron delocalisation from the π(C^2^C^3^) bond into an empty 4p Ge orbital (2nd order perturbation energy, Δ*E*^2nd^ = 4.80 eV) and from the σ-GeC^1^ and σ-GeC^4^ bonds into the π*(C^2^C^3^) bond (Δ*E*^2nd^ = 2.54 eV) (see ESI, Fig. S118†). These delocalisations lead to significant covalent bonding between the germanium atom and all four carbon atoms of the butadiene moiety. This is expressed by significant Wiberg bond indices (WBIs)^41^ between these atoms (WBI(GeC^1^) = 0.58), WBI(GeC^2^) = 0.34 *vs.* WBI(GeC(GeMe_4_) = 0.83) and by the three dominant resonance structures 15(M)A–C predicted by natural resonance theory (NRT) calculations (see Fig. 6).^42^

**Fig. 7:**
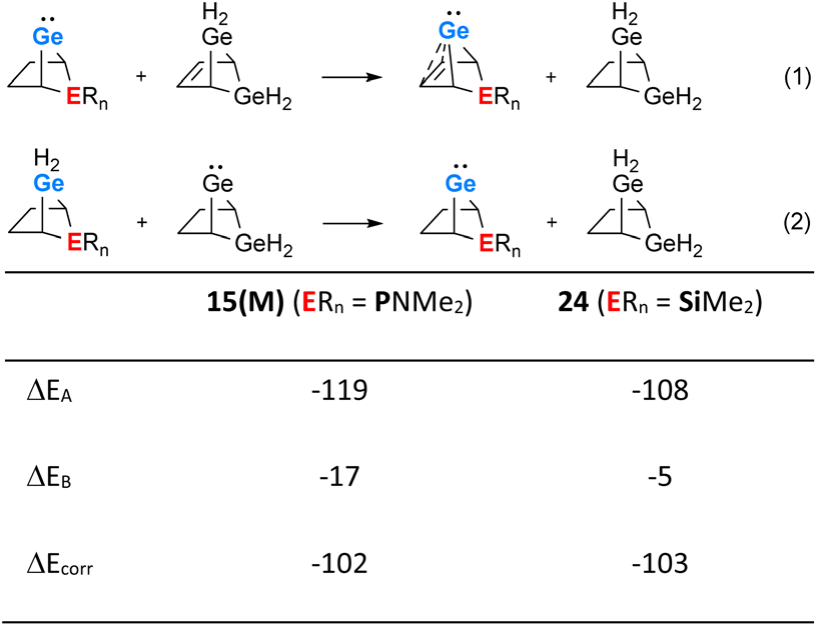
Isodesmic eqn (1) and (2) and values calculated for phospha-BCH-germylene 15(M) (ER_*n*_ = PNMe_2_) and sila-BCH-germylene 24 (ER_*n*_ = SiMe_2_) (M06-2X/6-311+G(d,p)).

The use of isodesmic reactions allows us to quantify the stabilisation of phospha-BCH-germylene 15(M) by homoconjugation between the germanium atom and the remote π-bond of the bicyclic cyclohexene skeleton (Fig. 7, eqn (1) and (2)). The results for eqn (1) indicate that germylene 15(M) is stabilized through the homoconjugation between the remote C=C double bond and the germanium centre by −119 kJ mol^−1^. The sila-BCH-germylene 24 that was calculated for comparison is slightly less stabilized (−108 kJ mol^−1^). Eqn (2) takes into account possible interactions of the heteroatom with the germanium(ii) centre. These interactions are for both compounds, 15(M) and 24, much smaller. The correction of the stabilisation energy by homoconjugation (eqn (1)) by the effect of the heteroatoms (eqn (2)) leads to both compounds having very similar and large stabilization energies (−102 and −103 kJ mol^−1^, Fig. 7) due to the cyclohexene cage.

The delocalised electronic structure suggests for 15(M), and similarly for phospha-BCHGe's 15, also the alternative description as as a phospha-bridged *arachno*-cluster (4 CH groups and 1 germanium atom with 16 electrons)^43–45^ with a bridged germa[4]pyramidane structure. Lee and Gapurenko recently suggested this type of structure for the hafnocene derivative 1 (see Fig. 6).^46^

### Reactivity studies

For the reactivity studies of phospha-BCHGe's, we used preferentially the N^*i*^Pr_2_- and NCy_2_-substituted germylenes 15b and 16b as they were formed with only small contamination of the phospholes 20b and 21b (below 5%, see Scheme 1). For comparison purposes, also the trimethylsilyl-substituted derivatives 15a and 16a were tested. In these cases, the reaction time for the preparation was shortened to 5–10 min to minimise the amount of phosphole byproducts 20a and 21a.

**Scheme 2:**
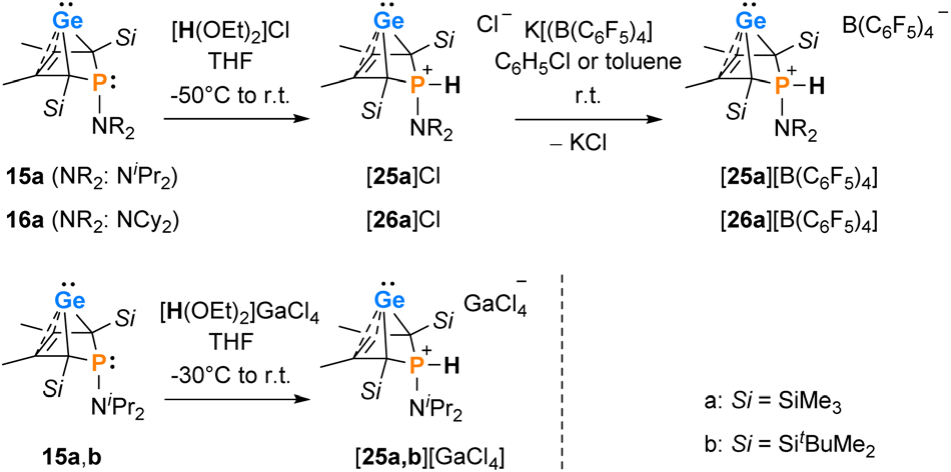
Protonation of phospha-BCHGe's 15, 16.

Interestingly, protonation of germylenes 15a/15b and 16a with protonated diethylether exclusively gave the bicyclic phosphonium germylenes [25a/b]^+^ and [26a]^+^ in isolated yields up to 80% (Scheme 2). The expected N-protonation did not occur to a sizeable amount.^47^ The ^13^C NMR parameters of the products of protonation are very close to those of the starting germylenes, which suggests a bicyclic structure also for the product (Table 2). The NMR spectra displayed characteristic large ^1^*J*_P,H_ coupling constants of 566–580 Hz in both the ^1^H and the hydrogen coupled ^31^P NMR spectrum. ^1^H NMR and ^31^P NMR chemical shifts of the P–H unit depend on the solvent (*i.e.* for [25a][B(C_6_F_5_)_4_]: *δ*^1^H = 6.86 (C_6_D_5_Cl) and 7.88 (THF-d_8_) and *δ*^31^P = 14.5 (C_6_D_5_Cl) and 28.4 (THF-d_8_)) as well as on the anion (*i.e.* for [25a][GaCl_4_] in C_6_D_6_: *δ*^1^H = 7.97 and *δ*^31^P = 31.0) (Table 2). Interestingly, the trimethylsilyl-substituted phosphonium germylene [25a]^+^, which was synthesised from *in situ* prepared germylene 15a, did not undergo the elimination reaction of germanium. It was isolated in 80% yield. In addition, there was no indication for the formation of the corresponding phospholium ions or their follow-up products.^48^

**Table 2:** Selected NMR spectroscopic data of phospha-BCHGe's 15a and 15b and phosphonium-BCHGe's [25a]^+^, [25b]^+^ and [26a]^+^

	Counterion	Solvent	*δ* ^13^C (C^1/4^)	*δ* ^13^C (C^2/3^)	*δ* ^31^P	*δ* ^1^H (P–H)
15a	—	C_6_D_6_	74.5	129.9	35.4	—
	—	THF-d_8_	74.6	130.7	33.3	—
15b	—	C_6_D_6_	72.8	131.1	53.8	—
[25a]^+^	GaCl_4_^−^	C_6_D_6_	59.7	124.5	31.0	7.97
	[B(C_6_F_5_)_4_]^−^	C_6_D_5_Cl	70.2	127.7	14.5	6.86
	[B(C_6_F_5_)_4_]^−^	THF-d_8_	60.6	125.6	28.4	7.89
[25b]^+^	GaCl_4_^−^	THF-d_8_	67.8	129.3	25.7	7.78
[26a]^+^	[B(C_6_F_5_)_4_]^−^	C_6_D_5_Cl	69.9	127.6	13.4	6.96

**Fig. 8:**
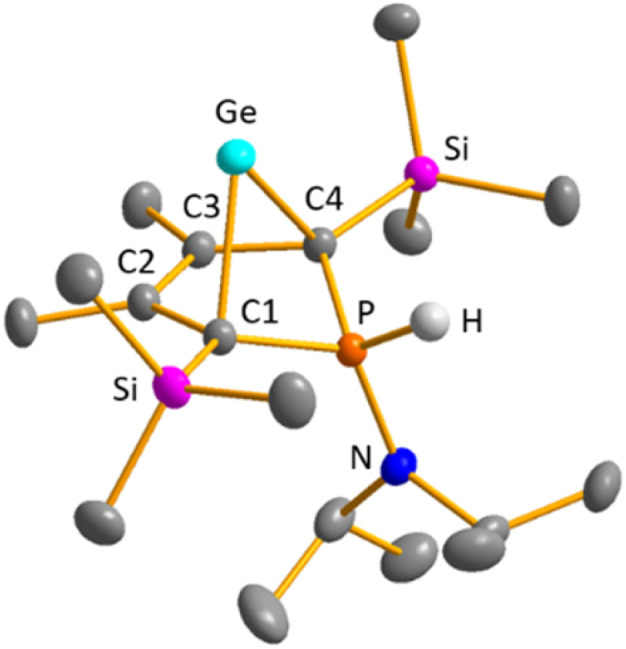
Molecular structure of phosphonium-BCH-germylene [25a]^+^ in the crystal of [25a][GaCl_4_]. Thermal ellipsoids at 50% probability. Hydrogen atoms (except P–H) and the counterion [GaCl_4_]^−^ are omitted for clarity. Selected atomic distances [pm] and angles [°]: C^1^–C^2^ 147.69 (18), C^2^–C^3^ 141.40 (17), Ge–C^1^ 220.51 (13), Ge–C^2^ 222.41 (12), P–C^1^ 177.91 (14), P–N 163.98 (13), C^1^–Ge–C^4^ 81.919 (11).

Single crystals of phosphonium gallate [25a][GaCl_4_], suitable for sc-XRD analysis, were obtained upon layering of a benzene solution with pentane (Fig. 8). Comparison of the molecular structure to that of the precursor germylene 15a displays that upon protonation and quarternisation, the s-character of the orbitals involved in the bonding of the phosphorus atom towards the other atoms is enlarged. This results in shortening of the P–C^1^ and P–N bonds in the phosphonium ion [25a]^+^ compared to germylene 15a: The C^1^–P bonds in the phosphonium salt are almost 7 pm (177.4 pm) shorter than in germylene 15a. The P–N bond is shortened by 5 pm (163.9 pm) and almost equals a formal P=N double bond (162 pm). The metrics of the but-2-ene backbone of the molecule do not significantly change upon protonation. Both the C^1^–C^2^ and the C^2^–C^3^ bonds are shortened by less than 1 pm compared to the precursor 15a. The germanium–carbon distances, however, are slightly larger by about 4 pm (C^1^–Ge) and about 2 pm (C^2^–Ge), respectively.

**Fig. 9:**
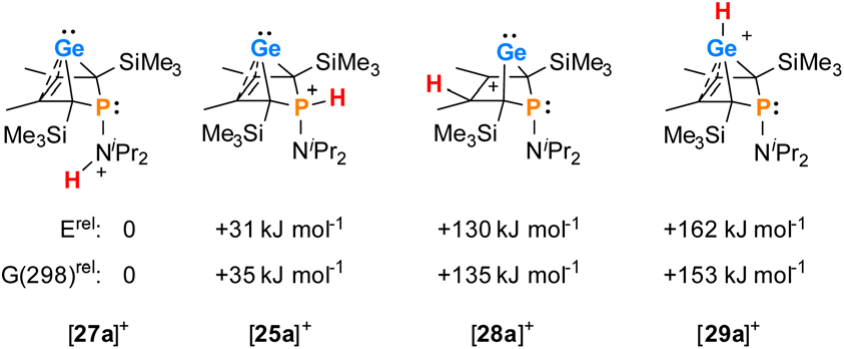
Relative energies of isomers of phosphonium-BCHGe [25a]^+^ at (ICPCM(solvent = THF)/M062X/6-311+G(d,p)//M062X/6-311+G(d,p)).

**Fig. 10:**
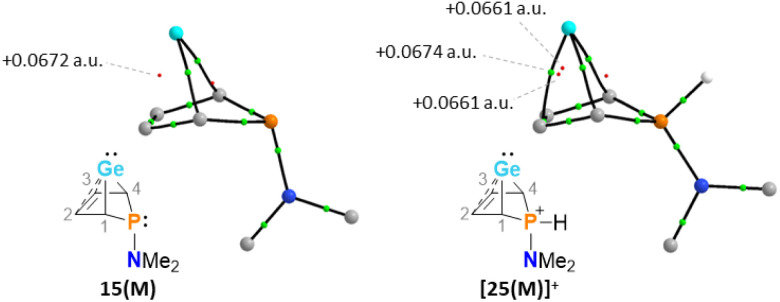
Topological graphs of phospha-BCH-germylene 15(M) and phosphonium-BCH-germylene [25(M)]^+^ according to QTAIM analyses (black lines are bond paths, small green spheres designate the corresponding bond critical points (bcp), which are minima of the electron density along the bond path. Small red spheres indicate ring critical points which represent local minima in rings defined by bond paths; calculated electron densities at critical points are given in black (M06-2X/def2-tzvp//M06-2X/6-311+G(d,p))).

The selective protonation of BCHGe's 15 and 16 at the phosphorus atom is surprising as aminophosphanes are usually protonated at the nitrogen atom.^47^ The results of quantum mechanical calculations for the different protonation sites of BCHGe 15a reveal that the bicyclic phosphonium ion [25a]^+^ is by 31 kJ mol^−1^ less stable than the isomeric ammonium ion [27a]^+^ (Fig. 9). This suggests that the observed selective protonation at phosphorus is of kinetic origin. The close similarity of the molecular structures of BCHGe 15a and of phosphonium-BCHGe [25a]^+^ indicates very similar stabilization mechanisms for both types of germylenes. Indeed, the calculated stabilization by homoconjugation (Fig. 7, eqn (1)) of the model compound [25(M)]^+^ is almost exactly as high as predicted for 15(M) (Δ*E*_A_ = 119 kJ mol^−1^).^49^ Notable are the results of the analysis of the computed electron density of both model compounds, 15(M) and [25(M)]^+^, based on the quantum theory of atoms in molecules (QTAIM) (Fig. 10).^50^ For phospha-BCHGe 15(M), the calculated molecular graph displays a dicoordinated germylene with bonds between the germanium and the C^1^ and C^4^ carbon atoms. The ring critical point (rcp) of the five-membered C_4_–Ge ring is located close to a plane spanned by the C^2^–C^3^ bond and the germanium atom, indicating the C^2^=C^3^ → Ge interaction (Fig. 10, left). The calculated molecular graph of phosphonium-BCHGe [25a(M)]^+^ is different as it displays an additional bonding path between the midpoint of the C^2^=C^3^ bond and the germanium atom (Fig. 10, right). This T-shaped electron distribution is typical for π-complexes of alkenes with electron deficient centres^51–53^ and clearly indicates the electron delocalization from the C=C double bond to the germanium atom (homoconjugation).^22^ This obvious difference between the topology of 15(M) and [25(M)]^+^, however, vanishes during quantitative analysis of the data. The bond critical point (bcp) of the additional bond path of [25(M)]^+^ is located approximately at the same position where the rcp of the GeC_4_ ring in phospha-BCH-germylene 15(M) is found (Fig. 10). The rcps of the two thereby emerging rings are located close to this bcp (Fig. 10, right). Additionally, the absolute values of the electron densities at the rcps and the bcp in [25(M)]^+^ are very similar and furthermore very close to the electron density at the rcp in 15(M) (see Fig. 10). This indicates great similarity of the electron density distribution in both compounds, 15(M) and [25(M)]^+^, and provides conclusive evidence from the QTAIM analysis for the homoconjugative interaction between the C^2^=C^3^ double bond and the germanium atom in both compounds.

**Scheme 3:**
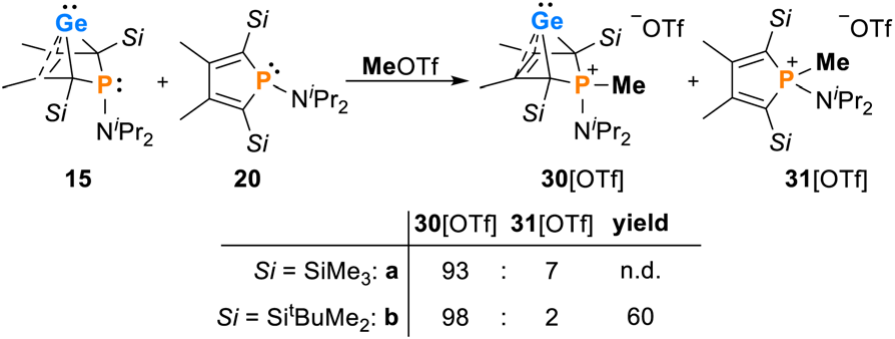
Reactivity of phospha-BCHGe's 15 towards MeOTf.

The addition of methyl triflate to solutions of germylenes 15 in pentane resulted in the formation of the methylphosphonium salts [30]OTf which were isolated in up to 60% yield. The product was contaminated with small amounts of the methylphospholium triflates 31, resulting from the methylation of the phosphole byproducts 20 (2–7%, Scheme 3, see the ESI† for identification of [20][OTf]). The methylphosphonium salts [30]OTf were characterised by NMR spectroscopy. In the ^1^H NMR spectrum, the new P–Me groups feature a doublet signal at *δ*^1^H = 2.35 (^2^*J*_P,H_ = 12.6 Hz [30a]^+^) and 2.30 (^2^*J*_P,H_ = 11.6 Hz [30b]^+^). The ^13^C NMR chemical shifts of the C^1/4^ bridgehead carbon atoms (*δ*^13^C(C^1/4^) = 67.2 [30a]^+^, 68.4 [30b]^+^) are in the typical range for BCHGe's (Tables 1 and 2). The ^31^P NMR signals were shifted to higher frequency compared to their precursors (*δ*^31^P = 54.5 [30a]^+^, 57.5 [30b]^+^). Treating the methylphosphonium triflate [30b][OTf] with a second equivalent of methyl triflate did not result in the methylation of the germylene unit. Only the precursor was isolated.

**Scheme 4:**
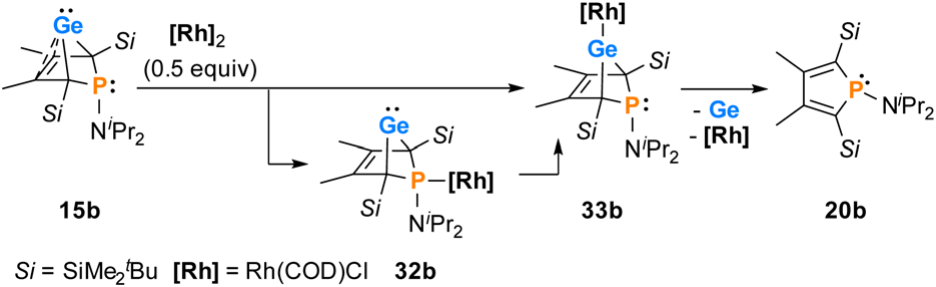
Reactivity of germylene 15b towards a Rh(i) complex.

**Fig. 11:**
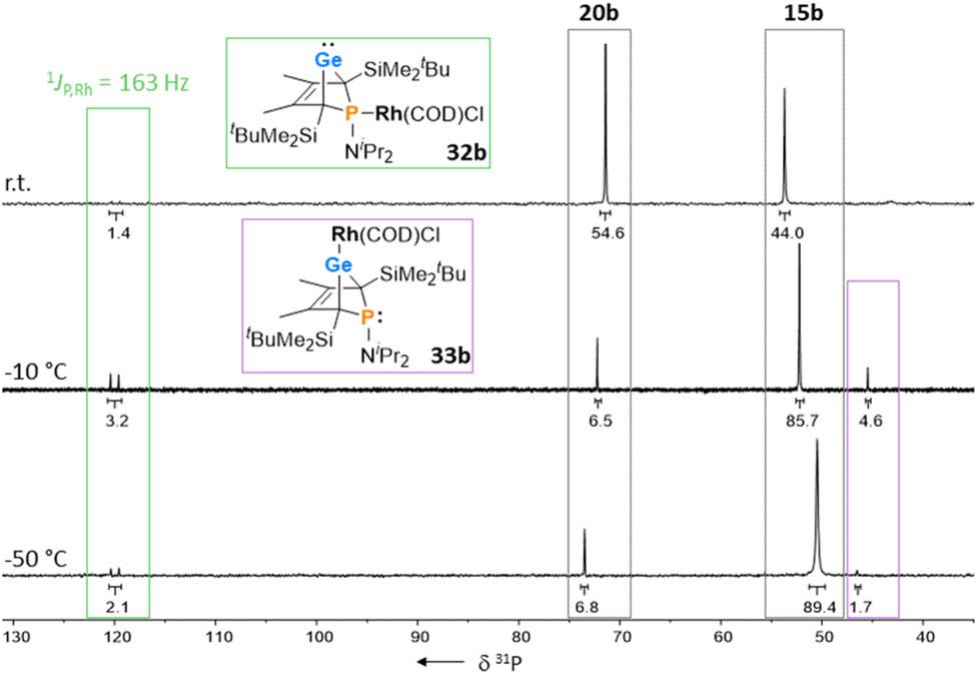
^31^P{^1^H} VT NMR spectra of the reaction of BCHGe 15b with [(COD)RhCl]_2_, recorded in toluene-d_8_.

The reactions of phospha-BCHGe's 15 towards various late transition metal complexes were studied to examine their suitability as ligands as well as their preferred coordination mode. In most cases, reductive elimination of germanium occurred and phospholes 20 or their corresponding metal complexes were obtained as final products. The NMR monitoring of the reaction of BCH-germylene 15b with the dimeric cyclooctadiene-rhodium(i)chloride complex [(COD)RhCl]_2_, [Rh]_2_, allows a more detailed understanding of the observed reaction sequence. At room temperature, the reaction mixture of germylene 15b and [Rh]_2_ turned green within five minutes. After an additional five minutes of stirring, the solution turned dark, and a black precipitate was formed. The elimination of germanium and rhodium metal was confirmed by analysis of NMR spectra, recorded from the crude reaction mixture. Almost pure phosphole 20b (*δ*^31^P = 71.7) was obtained (Scheme 4). Furthermore, signals of non-coordinated cyclooctadiene (*δ*^1^H = 5.53 (=C**H**–), 2.23 (C**H**_2_)) were displayed in the ^1^H NMR spectrum, showing that the rhodium complex decomposed as well during the reaction. To monitor the reaction and detect possible intermediate complexes, the reaction was carried out at room temperature for 5 min and then cooled to *T* = −60 °C. The reaction was followed by NMR spectroscopy at stepwise increasing temperatures (Fig. 11). Already the first ^31^P{^1^H} NMR spectrum, recorded at *T* = −50 °C, indicated the formation of the phosphole 20b from the germylene 15b (Fig. 11). Additional signals were detected at *δ*^31^P = 46.5 and 120.0. The doublet signal at *δ*^31^P = 120.0 shows a coupling constant of ^1^*J*_P,Rh_ = |163| Hz which is in the reported range of direct ^1^*J*_P,Rh_ coupling constants in quadratic planar Rh(i) complexes (^1^*J*_P,Rh_ = |110–210| Hz).^54,55^ Using 2D NMR spectroscopy (see ESI, Fig. S95 and S96†), the signals were assigned to complexes 32b and 33b, both exhibiting a bicyclic backbone structure with the characteristic ^13^C NMR chemical shift pattern (*δ*^13^C(C^1/4^) = 60.1, *δ*^13^C(C^2/3^) = 129.0 (32b) and *δ*^13^C(C^1/4^) = 57.3, *δ*^13^C(C^2/3^) = 125.3 (33b)). At *T* = −10 °C, the relative intensity of these two ^31^P NMR signals slightly increased. At room temperature, they almost vanished, suggesting a short lifetime for these two complexes (Fig. 11).

The reactivity studies suggest that germylene 15b coordinates *via* both the phosphorus (complex 32b) and the germanium atom (complex 33b) to the rhodium centre. In the latter case, irreversible elimination to form phosphole 20b occurs. The phosphane complex 32b isomerises to the germylene complex 33b which then decomposes to give phosphole 20b (Scheme 4). Additional experiments show that phosphole 20b does not form complexes with [(COD)RhCl]_2_. The results of DFT calculations indicate that the formation of the trimethylsilyl-substituted phosphanyl rhodium complex 32a is more stable than the germylene rhodium complex 33a by Δ*E* = 9 kJ mol^−1^ (M062X/6-311+G(d,p)). This small energy difference suggests the possibility for the proposed 32b → 33b isomerisation.

**Scheme 5:**
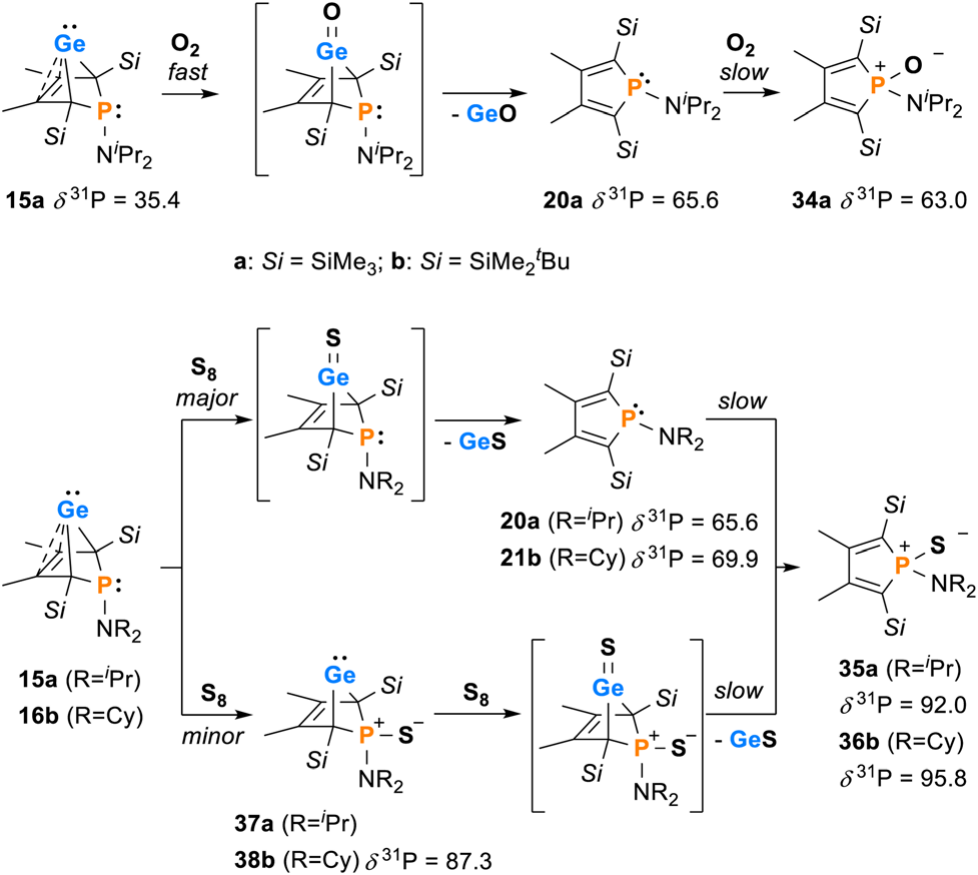
Reactivity of BCHGe's 15, 16 towards elemental oxygen and sulphur.

**Fig. 12:**
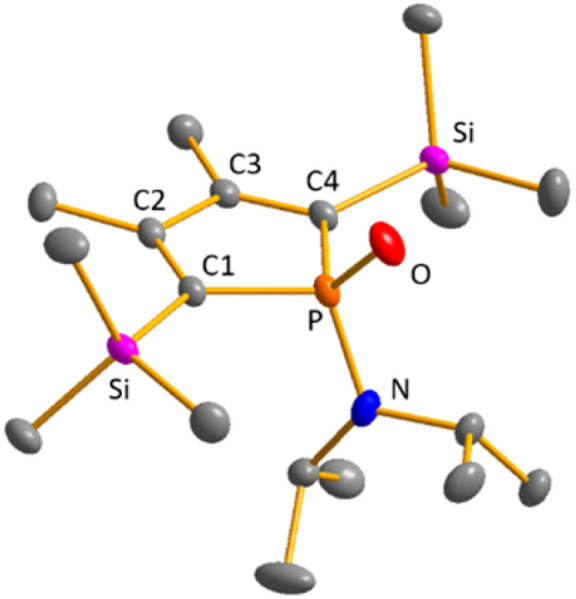
Molecular structure of phosphole oxide 34a in the crystal. Thermal ellipsoids at 50% probability. Hydrogen atoms are omitted for clarity. Selected atomic distances [pm] and angles [°]: C^1^–C^2^ 135.42 (10), C^2^–C^3^ 151.31 (11), P–C^1^ 180.46 (8), P–O 148.78 (6).

Next, the reactivity of phospha-BCHGe's 15 and 16 towards elemental oxygen and sulphur was examined (Scheme 4). The related hafnocena-BCHGe 1 formed 1,3-digermetanes upon treatment with elemental chalcogens, featuring four-membered Ge–Ch–Ge–Ch rings (Ch = S, Se and Te).^56^ Exposure to dioxygen led to decomposition, giving the corresponding hafnocenacyclopentadiene and germanium monoxide. Phospha-BCHGe's 15 and 16 showed similar reactivity, preferably reacting with the germylene site towards oxygen and sulphur. Upon exposure of germylene 15a to oxygen, a two-step reaction was observed (Scheme 5, top). In the first, fast step (5 min), pale yellow germanium monoxide was eliminated, giving the phosphole 20a (*δ*^31^P = 65.6). The ^31^P NMR spectrum of the reaction mixture already showed an additional small signal, corresponding to the phosphole oxide 34a (*δ*^31^P = 63.0). In the second, slow step (16 h), the phosphorus atom of phosphole 20a was oxidised, quantitatively giving phosphole oxide 34a (Scheme 5, top; identification see ESI †). Phosphole oxide 34a is stable *versus* Diels–Alder dimerization, a follow-up reaction that is frequently observed for phosphole oxides.^30,31^ Obviously, the large substituents prevent dimerization in this case. Phosphole oxide 34a was fully analysed by NMR spectroscopy (see ESI †). Additionally, colourless crystals, obtained upon recrystallization from pentane at −30 °C, were analysed by sc-XRD (Fig. 12). The molecular structure of 34a shows the expected localized butadiene group (C^1^–C^2^ = 135.42 pm; C^2^–C^3^ = 151.37 pm) and a tetracoordinated phosphorus atom with a very short P–O bond (P–O = 148.78 pm). The reaction of BCHGe 15a with elemental sulphur proceeds similarly. Elimination of GeS and formation of the phosphole 20a was observed. Subsequent oxidation by a second equivalent of sulphur gave, after 16 h reaction time, quantitatively phosphole sulphide 35a. The product is characterized by a ^31^P NMR resonance at *δ*^31^P = 92.0 (full characterisation of sulphide 35a, see ESI†). The reaction of the NCy_2_-substituted phospha-BCHGe 16b with elemental sulphur proceeded slower and revealed a second reaction pathway (Scheme 5, bottom). The predominant pathway was found to be the initial oxidation of the germanium atom and the subsequent elimination of GeS, giving phosphole 21b and finally phosphole sulphide 36b. Additionally, phospha-BCHGe sulphide 38b was detected in the reaction mixture after 20 min at r.t. (ratio 38b : 21b = 1 : 2.5). This suggests that in this case, the oxidation at the phosphorus atom competes with the reaction at germanium (see ESI Fig. S109–S112†). The reaction was completed after 5 days at r.t., giving pure phosphole sulfide 36b.

**Fig. 13:**
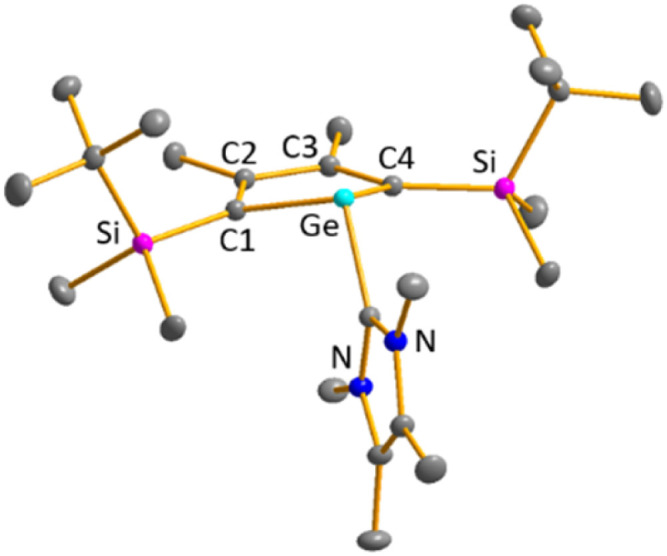
Molecular structure of NHC-stabilised germylene 40b in the crystal. Thermal ellipsoids at 50% probability. Hydrogen atoms are omitted for clarity. Selected atomic distances [pm] and angles [°]: C1–C2 137.11 (7), C2–C3 148.60 (9), Ge–C(NHC) 203.93 (5), ΣGe 292.8.

**Scheme 6:**
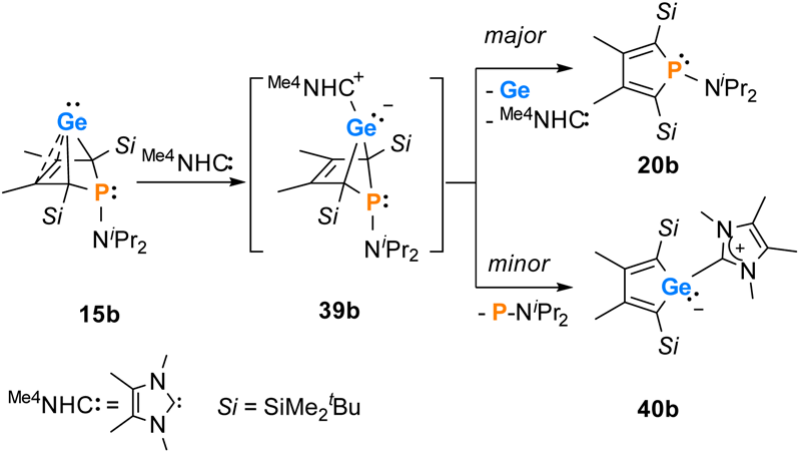
Reaction of ^Me4^NHC with germylene 15b.

Finally, the reactivity towards nucleophiles was studied. Addition of tetramethylimidazol-2-ylidene (^Me4^NHC) to a solution of germylene 15b at room temperature induced a colour change from yellow-orange to brownish red as well as the precipitation of a red solid.^57,58^ Control NMR spectra after 24 h displayed a mixture of germylene 15b, non-coordinated ^Me4^NHC and, as the main product, an increased amount of phosphole 20b. Additionally, we identified the germolylidene-^Me^NHC complex 40b as the minor byproduct by NMR spectroscopy (see ESI† for NMR data). A small batch of yellow crystals of the stabilized germylene 40b, suitable for sc-XRD analysis, was isolated from the red precipitate and secured the identification of the NHC-stabilized germylene 40b (Fig. 13, Scheme 6). The ratio of phosphole 20b and germolylidene 40b was determined to be 9 : 1 from ^1^H NMR spectroscopy. This suggests the following mechanistic scenario: nucleophilic attack of the ^Me4^NHC at the germanium atom forms the bicyclic intermediate 39b. This intermediate eliminates one of the two isolobal fragments which is either the germolylidene NHC-Ge or the phosphinidene ^*i*^Pr_2_N–P.^57,58^ Completion of the reaction took seven days with an equimolar amount of ^Me4^NHC and three days with two equivalents of ^Me4^NHC. Weaker nucleophiles (4-dimethylaminopyridine (DMAP) or THF) as well as strong, but sterically more demanding ones (^Dipp^NHC, Dipp: 2,6-diisopropylphenyl) did not react with germylene 15b. These results clearly indicate the importance of steric factors for the germanium elimination from the germylenes 15, induced by nucleophiles. According to calculations of the buried volume,^59–61^^Me4^NHC is significantly smaller than ^Dipp^NHC (% *V*_bur_ = 25.8 (^Me4^NHC) *vs.* 44.0 (^Dipp^NHC), see ESI† for details). Interestingly, the size of germylene 15a (% *V*_bur_ = 33.5) is comparable to that of ^Me4^NHC, while 15b (% *V*_bur_ = 41.9) is almost as large as ^Dipp^NHC. This comparison supports the above postulated self-induced germanium elimination of SiMe_3_-substituted phospha-BCHGe's 15a/16a/18a and furthermore gives hint on why this process is not observed for the sterically more encumbered SiMe_2_^*t*^Bu substituted phospha-BCHGe's 15b/16b/17b/18b.

## Conclusions

Phospha-BCHGe's 15–18 are synthesized by salt metathesis reaction between potassium salts of germole dianions K2[8] and dichloroaminophosphanes 10–13. The size of the flanking silyl groups is of importance for the long-term stability of germylenes. At room temperature in solution, the small SiMe_3_-substituted germylenes 15a–18a undergo a self-induced elimination of elemental germanium and form the corresponding phospholes 19–23. In contrast, germylenes 15b–18b with larger SiMe_2_^*t*^Bu-groups are stable at room temperature. The germylenes 15–18 are stabilized by homoconjugation between the empty 4p orbital at the dicoordinated germanium atom and the remote C^2^=C^3^ double bond. Therefore, reactivity studies show reduced electrophilicity of the germylenes. Small and strong nucleophiles add to the germanium atom and after elimination of germanium, the corresponding phospholes are formed. Small electrophiles add to the phosphorus atom, forming cationic phosphonium-BCHGe's [25]^+^, [26]^+^ and [30]^+^. The reaction of phospha-BCHGe's with electrophilic transition metal complexes leads to the elimination of germanium and the formation of the corresponding phospholes. Low temperature NMR studies of the reaction of 15b with (COD)RhCl dimer revealed the subsequent formation of a Rh–phosphane 32b and Rh–germylene complex 33b prior to elimination of germanium and generation of the phosphole 20b. Oxidation of phospha-BCHGe's 15 with elemental oxygen and sulphur leads to elimination of GeCh (Ch = O, S) and intermediate formation of the phospholes 20. The final products of these oxidation reactions are the corresponding phosphole chalcogenides 34, 35 and 36.

Overall, the herein introduced phospha-BCHGe's 15–18 exhibit strong nucleophilic but also non-neglectable electrophilic properties. This allows ranking of the reactivity of these germylenes, stabilized by homoconjugation with the remote C=C double bond, in between the silyl-substituted germylene III and NHGe IV (Fig. 1).

## Author contributions

Investigation, data curation, formal analysis, validation: MSW, SK (supporting), TB (supporting); writing: MSW, TM; conceptualization, funding acquisition, project administration, supervision, methodology, resources: TM; XRD: MS.

## Conflicts of interest

There are no conflicts to declare.

## Supplementary Material

SC-015-D4SC04034A-s001

SC-015-D4SC04034A-s002

SC-015-D4SC04034A-s003

## Data Availability

All experimental procedures along with the analytical data are available in the ESI.† The XRD data are deposited in the CCSD database. Original analytical data (source data) are available on request from the corresponding author. Computated molecular structures are given in the ESI† in XYZ coordinates, readable with the CCSD software “Mercury”.
