# Supplementary material for: Phospha-bicyclohexene-germylenes exhibiting unexpected reactivity
Source: Chem Sci. 2024 Jul 30;15(35):14161–70. doi: 10.1039/d4sc04034a (PMC11310892; doi:10.1039/d4sc04034a)
Supplement: SC-015-D4SC04034A-s002 [file SC-015-D4SC04034A-s002.pdf]

Supplementary Information (SI) for Chemical Science.
This journal is © The Royal Society of Chemistry 2024

Supplementary Information

Phospha-Bicyclohexene-Germylenes Exhibiting Unexpected Reactivity

Marie Sophie Würdemann,[a] Steffen Kühn,[a] Tobias Bötel,[a] Marc Schmidtmann[a] and Thomas Müller*[a]

a. Institute of Chemistry, Carl von Ossietzky Universität Oldenburg, Carl von Ossietzky-Str. 9-11, D-26129 Oldenburg, Federal Republic of Germany.

Table of Contents

Experimental Part 2
Starting Material 3
Germylenes and Phospholes 5
Reactivity Studies of the germylenes. 39
Crystallographic Data 73
Computational Details 81
References 88

Experimental Part

General. All operations were carried out under inert atmosphere using an MBraun glovebox filled with nitrogen and standard Schlenk technique. Glassware was stored in an oven at 160°C. It was evacuated and flushed with argon (dried over phosphoruspentoxide) before use.

Solvents and Chemicals. THF, diethyl ether, n-hexane and n-pentane were dried over sodium-potassium alloy. Benzene, benzene-d_6, toluene, toluene-d_8 and tetrahydrofurane-d_8 were dried over sodium. Chlorobenzene was dried over calcium chloride. All solvents were distilled prior to use and stored over molecular sieve (4Å). Chlorobenzene-d_5, chloroform-d_3 and dichloromethane-d_2 were dried and stored over molecular sieve (4Å). $GaCl_3$ was purified by sublimation prior to use. PCl_3 was distilled prior to use. Methyl triflate was stored over molecular sieve (4Å). All other chemicals are commercially available and were used without purification.

NMR spectroscopy. NMR spectra were recorded on a *Bruker Avance III RMN 1Bay* spectrometer (500 MHz), a *Bruker Avance DRX* spectrometer (500 MHz), a *Bruker Avance III WB500* spectrometer (500 MHz), a *JEOL JNM-ECZL* (500 MHz) and a *Bruker Furier 300* spectrometer (300 MHz). Chemical shifts δ are given in ppm relative to tetramethylsilane as standard. Coupling constants *J* are given in Hertz (Hz). ^{1}H NMR spectra were referenced internally using the residual proton signal of the deuterated solvent (Table S1). $^{13}C\{^{1}H\}$ NMR spectra were also referenced internally using the central line of the solvent signal. In case of more than one solvent signal, the most low-field shifted one was used for referencing. The spectra of all other nuclei were referenced to external standards which are given in Table S2. Additional to the one-dimensional experiments, the following two-dimensional NMR spectra were recorded to precisely assign all compound signals: $^{1}H^{13}C$ HMQC, $^{1}H^{13}C$ HMBC and $^{1}H^{31}P$ HMBC. Furthermore, $^{29}Si\{^{1}H\}$ INEPT experiments were performed using the parameters derived from $J_{Si,H}$ = 8 Hz for nine protons (for Bruker spectrometer: d3 = 0.0068 s and d4 = 0.0313 s. The multiplicity of NMR signals was entitled as follows: s (singlet), d (doublet), t (triplet), q (quartet), sept (septet), dt (doublet of triplets) and dsept (doublet of septets).

Table S1. Chemical shifts of the deuterated solvents (residual proton signals).

solvent	δ ^{1}H		δ ^{13}C
toluene-d_8	7.09	($C_6D_3\underline{H}(CD_3)$)	137.5
tetrahydrofuran-d_8	3.58	($C_4D_7\underline{H}O$)	67.2
benzene-d_6	7.16	($C_6D_5\underline{H}$)	128.1
chlorobenzene-d_5	7.14	($C_6D_4\underline{H}Cl$)	134.2
chloroform-d_3	7.26	($C\underline{H}Cl_3$)	77.2
dichloromethane-d_2	5.35	($CD\underline{H}Cl_2$)	53.8
D_2O capillary	4.79	($\underline{H}DO$)	–

Table S2. External NMR standards.

nucleus	δ	external standard
^{11}B	0.0	$BF_3{\cdot}Et_2O$
^{19}F	0.0	$CFCl_3$
^{29}Si	11.1	Me_2SiHCl
^{31}P	0.0	H_3PO_4 (85% in D_2O)
^{71}Ga	0.0	$Ga(NO_3)_3$ in C_6D_6

High resolution mass spectra. High resolution mass spectra (HR-MS) were recorded on a *Finnigan-LCG* or *Finnigan-MAT95* spectrometer.

Starting Material

Germoles. 1,1-Dichloro-2,5-bis(trimethylsilyl)-3,4-dimethylgermole **8a** and 2,5-bis(*tert*-butyldimethylsilyl)-1,1-dichloro-3,4-dimethylgermole **8b** as well as their dipotassium salts **K_2[8]** were synthesised according to literature procedures. The NMR spectroscopic data, given here for reference, matches the literature.[1-3]

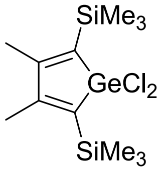

^{1}H NMR (500.1 MHz, 305 K, C_6D_6) δ = 0.31 (s, 18H, 2 x Si$\underline{Me_3}$), 1.65 (s, 6H, 2 x $C^{2/3}$–$\underline{Me}$).
^{13}C{^{1}H} NMR (125.7 MHz, 305 K, C_6D_6) δ = 0.2 (Si$\underline{Me_3}$), 19.4 ($C^{2/3}$–$\underline{Me}$), 132.9 ($C^{1/4}$), 160.9 ($C^{2/3}$).
^{29}Si{^{1}H} INEPT NMR (99.3 MHz, 305 K, C_6D_6) δ = -7.7.

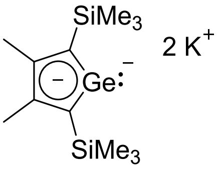

^{1}H NMR (500.1 MHz, 305 K, THF/D_2O capillary) δ = 0.55 (s, 18H, 2 x Si$\underline{Me_3}$), 2.66 (s, 6H, 2 x $C^{2/3}$–$\underline{Me}$).
^{13}C{^{1}H} NMR (125.7 MHz, 305 K, THF/D_2O capillary) δ = 4.8 (Si$\underline{Me_3}$), 20.9 ($C^{2/3}$–$\underline{Me}$), 130.9 ($C^{2/3}$), 156.1 ($C^{1/4}$).
^{29}Si{^{1}H} INEPT NMR (99.3 MHz, 305 K, THF/D_2O capillary) δ = -15.9.

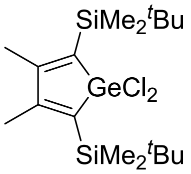

^{1}H NMR (500.1 MHz, 305 K, C_6D_6) δ = 0.36 (s, 12H, 2 x Si$\underline{Me_2}$^{t}Bu), 0.96 (s, 18H, 2 x SiMe$_2$$\underline{^tBu}$), 1.82 (s, 6H, 2 x $C^{2/3}$–Me).
^{13}C{^{1}H} NMR (125.7 MHz, 305 K, C_6D_6) δ = -3.2 (Si$\underline{Me_2}$^{t}Bu), 19.1 (SiMe$_2$$\underline{^tBu}$, C^4), 21.3 ($C^{2/3}$–Me), 27.6 (SiMe$_2$$\underline{^tBu}$), 132.2 ($C^{1/4}$), 161.7 ($C^{2/3}$).
^{29}Si{^{1}H} INEPT NMR (99.3 MHz, 305 K, C_6D_6) δ = 0.4.

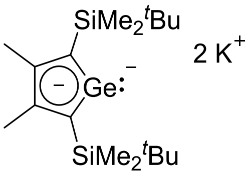

^{1}H NMR (500.1 MHz, 305 K, THF/D_2O capillary) δ = 0.15 (s, 12H, 2 x Si$\underline{Me_2}$^{t}Bu), 0.95 (s, 18H, 2 x SiMe$_2$$\underline{^tBu}$), 2.21 (s, 6H, 2 x $C^{2/3}$–Me).
^{13}C{^{1}H} NMR (125.7 MHz, 305 K, THF/D_2O capillary) δ = 0.6 (Si$\underline{Me_2}$^{t}Bu), 18.1 (SiMe$_2$$\underline{^tBu}$, C^4), 22.2 ($C^{2/3}$–Me), 28.9 (SiMe$_2$$\underline{^tBu}$), 130.1 ($C^{2/3}$), 151.4 ($C^{1/4}$).
^{29}Si{^{1}H} INEPT NMR (99.3 MHz, 305 K, THF/D_2O capillary) δ = -5.9.

Phosphanes. The amino(dichloro)phosphanes **10**-**13** were synthesised according to literature procedures. If not otherwise stated, the ^{31}P NMR chemical shift is in good agreement with the literature. Full NMR spectroscopic analysis is given below.[4-6]

Dichloro(di*iso*propylamino)phosphane **10**

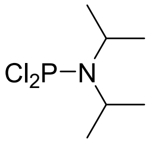

^{1}H NMR (500.1 MHz, 305 K, C_6D_6): δ = 0.96 (d, $^3J_{H,H}$ = 6.9 Hz, 12H, $N(CH\underline{Me_2})_2$), 3.62 (m, 2H, $N(C\underline{H}Me_2)_2$).
^{13}C{^{1}H} NMR (125.7 MHz, 305 K, C_6D_6): δ = 23.2 (d, $^3J_{C,P}$ = 8 Hz, $N(CH\underline{Me_2})_2$), 48.3 (d, $^2J_{C,P}$ = 14 Hz, $N(\underline{C}HMe_2)_2$).
^{31}P{^{1}H} NMR (202.4 MHz, 305 K, C_6D_6): δ = 169.0.

Dichloro(dicyclohexylamino)phosphane **11**

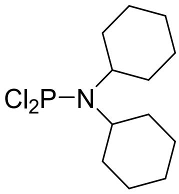

^{1}H NMR (500.1 MHz, 305 K, C_6D_6): δ = 0.82 (qt, $J_{H,H}$ = 13.1 Hz, $J_{H,H}$ = 3.7 Hz, 2H), 1.09 (q, $J_{H,H}$ = 13.1 Hz, 4H), 1.30 (qd, $J_{H,H}$ = 12.5 Hz, $J_{H,H}$ = 3.6 Hz, 4H), 1.36-1.43 (m, 2H), 1.50-1.57 (m, 4H), 1.70-1.77 (m, 4H), 3.38 (br, 2H, $\underline{H}C^{ipso}$).
^{13}C{^{1}H} NMR (125.7 MHz, 305 K, C_6D_6): δ = 25.5, 26.4, 34.5, 57.4 (d, $^2J_{C,P}$ = 11.6 Hz, C^{ipso}).
^{31}P{^{1}H} NMR (202.4 MHz, 305 K, C_6D_6): δ = 169.3.

Dichloro(piperidino)phosphane **12**

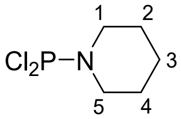

^{1}H NMR (500.1 MHz, 305 K, C_6D_6): δ = 1.06-1.16 (m, 6H, $C^{2/3/4}$-$\underline{H}$), 2.82-2.90 (m, 4H, $C^{1/5}$-$\underline{H}$).
^{13}C{^{1}H} NMR (125.7 MHz, 305 K, C_6D_6): δ = 24.0 ($\underline{C}^3$), 26.3 (d, $^3J_{C,P}$ = 5 Hz, $\underline{C}^{2/4}$), 47.4 (d, $^2J_{C,P}$ = 20 Hz, $\underline{C}^{1/5}$).
^{31}P{^{1}H} NMR (202.4 MHz, 305 K, C_6D_6): δ = 157.2.

Dichloro(2,2,6,6-tetramethylpiperidino)phosphane **13**

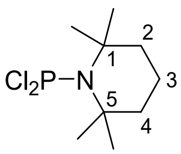

^{1}H NMR (500.1 MHz, 305 K, C_6D_6): δ = 1.16-1.26 (m, 6H, $C^{2/3/4}$-$\underline{H}$), 1.42 (br s, 12H, 4 x $\underline{Me}$).
^{13}C{^{1}H} NMR (125.7 MHz, 305 K, C_6D_6): δ = 16.5 ($\underline{C}^3$), 31.8 (br m, 4 x $\underline{Me}$), 41.1 ($\underline{C}^{2/4}$), 61.7 (m, $\underline{C}^{1/5}$).
^{31}P{^{1}H} NMR (202.4 MHz, 305 K, C_6D_6): δ = 163.2.

Germylenes and Phospholes

General procedure: Synthesis of aminophospha-BCH-germylenes 15-18

The THF solution (6 mL) of dipotassium germolediide, synthesised by the reduction of dichlorogermole (0.20 mmol) with potassium, was cooled to -50°C. The aminophosphane **9**-**13** (0.20 mmol), dissolved in THF (3 mL), was added. The reaction mixture was stirred for 30 minutes whilst it was allowed to warm to room temperature. The solvent was removed and the residue dissolved in diethyl ether and filtered over a PTFE syringe filter. The solvent was removed and the overall yield determined. NMR spectra were recorded without time delay.

Phospholes 19a and 19b

The THF solution (6 mL) of dipotassium germolediide **K_2[8]**, synthesised by the reduction of dichlorogermole (74 mg / 90 mg, 0.20 mmol) with potassium, was cooled to -50°C. Dichloro(dimethylamino)-phosphane **9** (29 mg, 0.20 mmol) was added directly to the cooled solution. The reaction mixture was stirred for 30 minutes whilst it was allowed to warm to room temperature. The solvent was removed and the residue redissolved in pentane. It was filtered over silica gel. Removal of the solvent gave phosphole **19a** (29 mg, 0.10 mmol, 50%) and phosphole **19b** (52 mg, 0.14 mmol, 70%) as yellow-orange oils containing minor impurities that could not be removed.
At room temperature, the NMR signals of the dimethylamino moiety cannot be observed. Therefore, low temperature NMR measurements (-40°C) were performed.

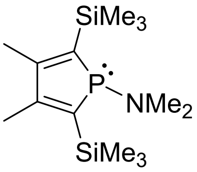

^{1}H NMR (499.9 MHz, 305 K, C_6D_6): δ = 0.34 (s, 18H, Si<u>Me</u>$_3$), 1.94 (d, $^4J_{H,P}$ = 5.6 Hz, 6H, $C^{2/3}$-<u>Me</u>).
^{13}C{^{1}H} NMR (125.7 MHz, 305 K, C_6D_6): δ = 0.5 (d, $^3J_{C,P}$ = 3 Hz, Si<u>Me</u>$_3$), 17.8 (d, $^3J_{C,P}$ = 6 Hz, $C^{2/3}$-<u>Me</u>), 143.6 (d, $^1J_{C,P}$ = 31 Hz, $C^{1/4}$), 153.4 (d, $^2J_{C,P}$ = 18 Hz, $C^{2/3}$).
^{29}Si{^{1}H} INEPT NMR (99.3 MHz, 305 K, C_6D_6): δ = -10.9 (d, $^2J_{Si,P}$ = 24 Hz).
^{31}P{^{1}H} NMR (202.4 MHz, 305 K, C_6D_6): δ = 94.3.
MS (EI): m/z (%) = 73 (86), 98 (43), 211 (52), 226 (29), 241 (30), 299 (100) [M^+].

Spectra recorded at -40°C:
^{1}H NMR (499.9 MHz, 233 K, toluene-d_8): δ = 0.33 (s, 18H, Si<u>Me</u>$_3$), 1.85 (d, $^4J_{H,P}$ = 5.6 Hz, 6H, $C^{2/3}$-<u>Me</u>), 1.98 (d, $^3J_{H,P}$ = 2.8 Hz, 3H, N<u>Me</u>$_2$), 2.68 (d, $^3J_{H,P}$ = 12.3 Hz, 3H, N<u>Me</u>$_2$).
^{13}C{^{1}H} NMR (125.7 MHz, 233 K, toluene-d_8): δ = 0.8 (d, $^3J_{C,P}$ = 3 Hz, Si<u>Me</u>$_3$), 18.3 (d, $^3J_{C,P}$ = 6 Hz, $C^{2/3}$-<u>Me</u>), 40.5 (d, $^2J_{C,P}$ = 12 Hz, N<u>Me</u>$_2$), 44.5 (d, $^2J_{C,P}$ = 39 Hz, N<u>Me</u>$_2$), 143.6 (d, $^1J_{C,P}$ = 30 Hz, $C^{1/4}$), 153.8 (d, $^2J_{C,P}$ = 18 Hz, $C^{2/3}$).

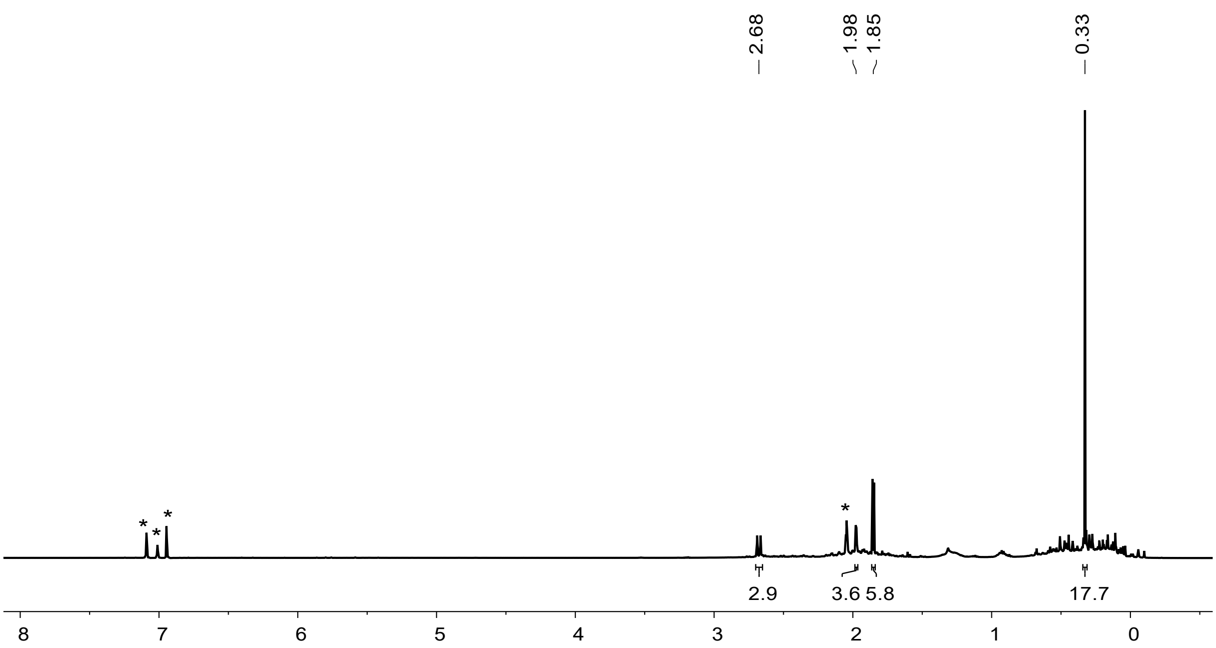

Figure S1. ^{1}H NMR spectrum (499.9 MHz, 233 K, toluene-d_8) of phosphole **19a** (*toluene-d_8).

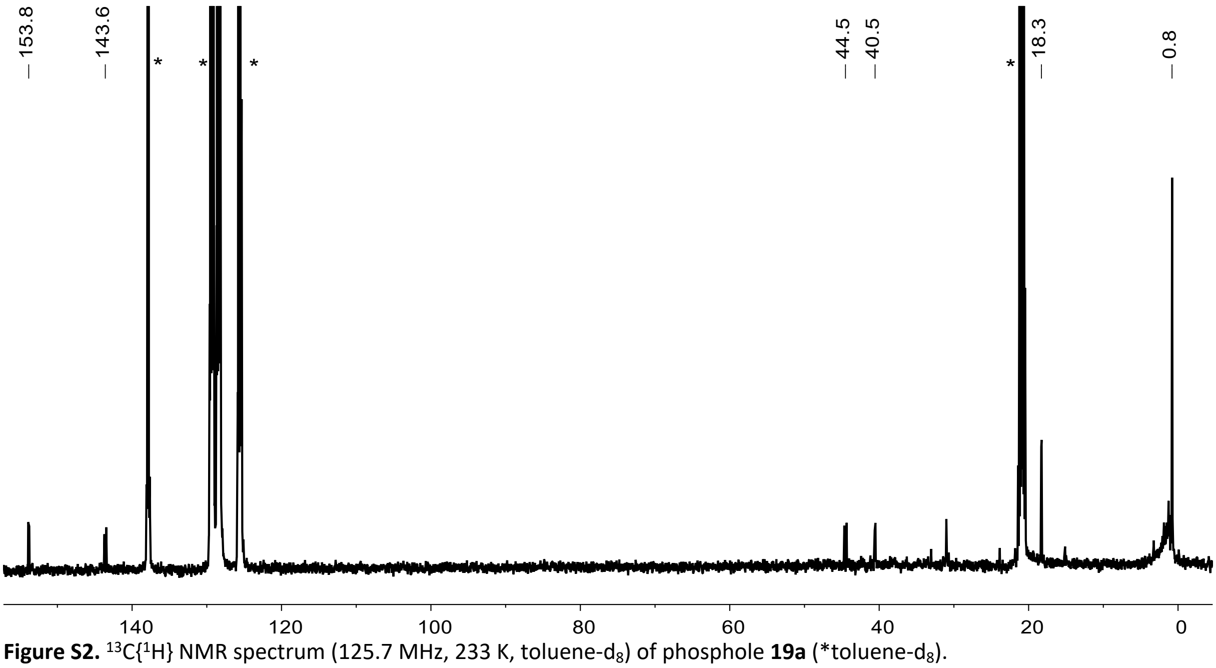

Figure S2. $^{13}C\{^{1}H\}$ NMR spectrum (125.7 MHz, 233 K, toluene-d_8) of phosphole **19a** (*toluene-d_8).

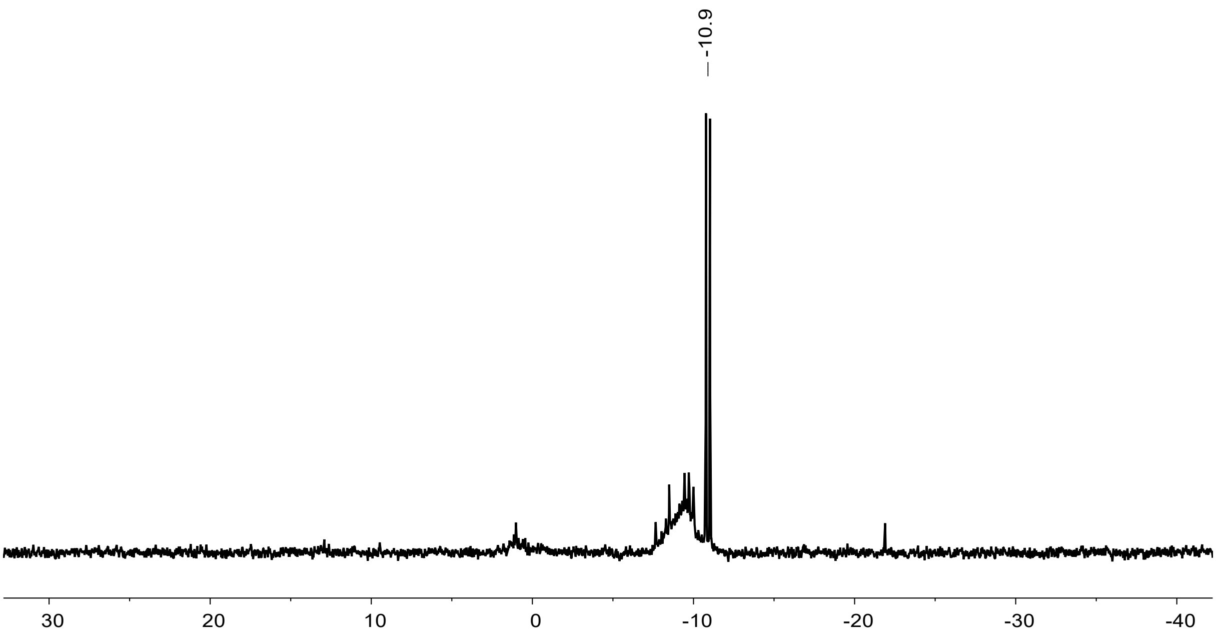

Figure S3. $^{29}Si\{^1H\}$ INEPT NMR spectrum (99.3 MHz, 305 K, benzene-d_6) of phosphole **19a**.

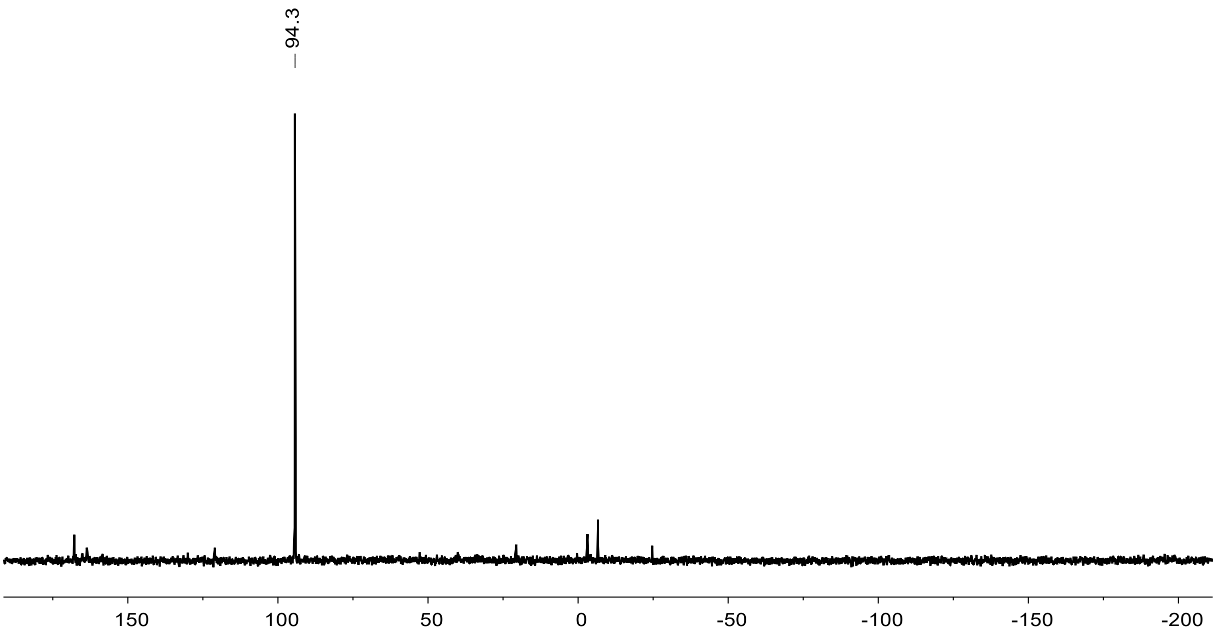

Figure S4. $^{31}P\{^1H\}$ NMR spectrum (202.4 MHz, 305 K, benzene-d_6) of phosphole **19a**.

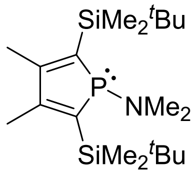

^{1}H NMR (499.9 MHz, 305 K, C_6D_6): δ = 0.32 (s, 12H, Si<u>Me</u>$_2$^{t}Bu), 1.04 (s, 18H, SiMe$_2$<u>^{t}Bu</u>), 2.04 (d, $^4J_{H,P}$ = 5.6 Hz, 6H, C$^{2/3}$-<u>Me</u>).

^{13}C{^{1}H} NMR (125.7 MHz, 305 K, C_6D_6): δ = -3.4 (m, Si<u>Me</u>$_2$^{t}Bu), 18.8 (SiMe$_2$<u>^{t}Bu</u>, C^4), 19.4 (d, $^3J_{C,P}$ = 6 Hz, C$^{2/3}$-<u>Me</u>), 27.8 (m, SiMe$_2$<u>^{t}Bu</u>), 141.2 (d, $^1J_{C,P}$ = 33 Hz, C$^{1/4}$), 154.2 (d, $^2J_{C,P}$ = 17 Hz, C$^{2/3}$).

^{29}Si{^{1}H} INEPT NMR (99.3 MHz, 305 K, C_6D_6): δ = -2.5 (d, $^2J_{Si,P}$ = 22 Hz).

^{31}P{^{1}H} NMR (202.4 MHz, 305 K, C_6D_6): δ = 98.5.

MS (EI): m/z (%) = 73 (32), 98 (100), 282 (20), 327 (16), 383 (8) [M$^+$].

Spectra recorded at -40°C:

^{1}H NMR (499.9 MHz, 233 K, toluene-d$_8$): δ = 0.21 (s, 6H, Si<u>Me$_2$</u>^{t}Bu), 0.36 (s, 6H, Si<u>Me$_2$</u>^{t}Bu), 1.00 (s, 18H, SiMe$_2$<u>^{t}Bu</u>), 1.96 (d, $^3J_{H,P}$ = 2.2 Hz, 3H, N<u>Me$_2$</u>), 1.98 (d, $^4J_{H,P}$ = 5.6 Hz, 6H, C$^{2/3}$-<u>Me</u>), 2.67 (d, $^3J_{H,P}$ = 11.8 Hz, 3H, N<u>Me$_2$</u>).

^{13}C{^{1}H} NMR (125.7 MHz, 233 K, toluene-d$_8$): δ = -3.9 (Si<u>Me$_2$</u>^{t}Bu), -2.6 (d, $^3J_{C,P}$ = 7 Hz, Si<u>Me$_2$</u>^{t}Bu), 19.3 (SiMe$_2$<u>^{t}Bu</u>, C^4), 19.9 (d, $^3J_{C,P}$ = 6 Hz, C$^{2/3}$-<u>Me</u>), 28.0 (SiMe$_2$<u>^{t}Bu</u>), 40.7 (d, $^2J_{C,P}$ = 12 Hz, N<u>Me$_2$</u>), 44.6 (d, $^2J_{C,P}$ = 40 Hz, N<u>Me$_2$</u>), 141.4 (d, $^1J_{C,P}$ = 33 Hz, C$^{1/4}$), 154.6 (d, $^2J_{C,P}$ = 17 Hz, C$^{2/3}$).

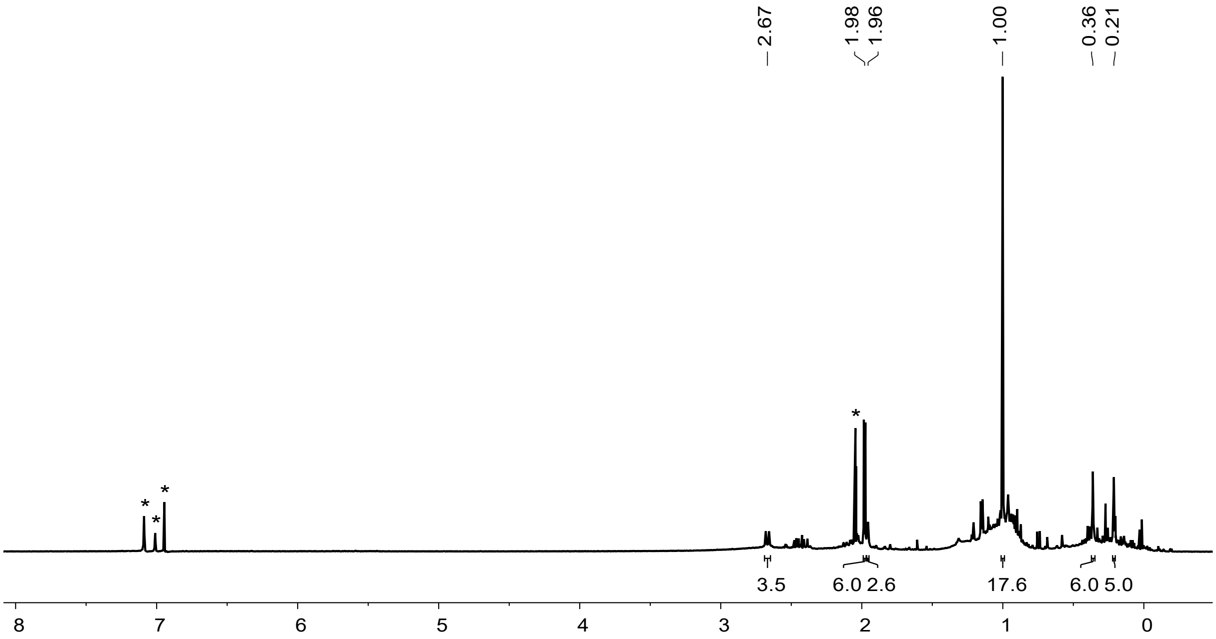

Figure S5. ^{1}H NMR spectrum (499.9 MHz, 233 K, toluene-d$_8$) of phosphole **19b** (*toluene-d$_8$).

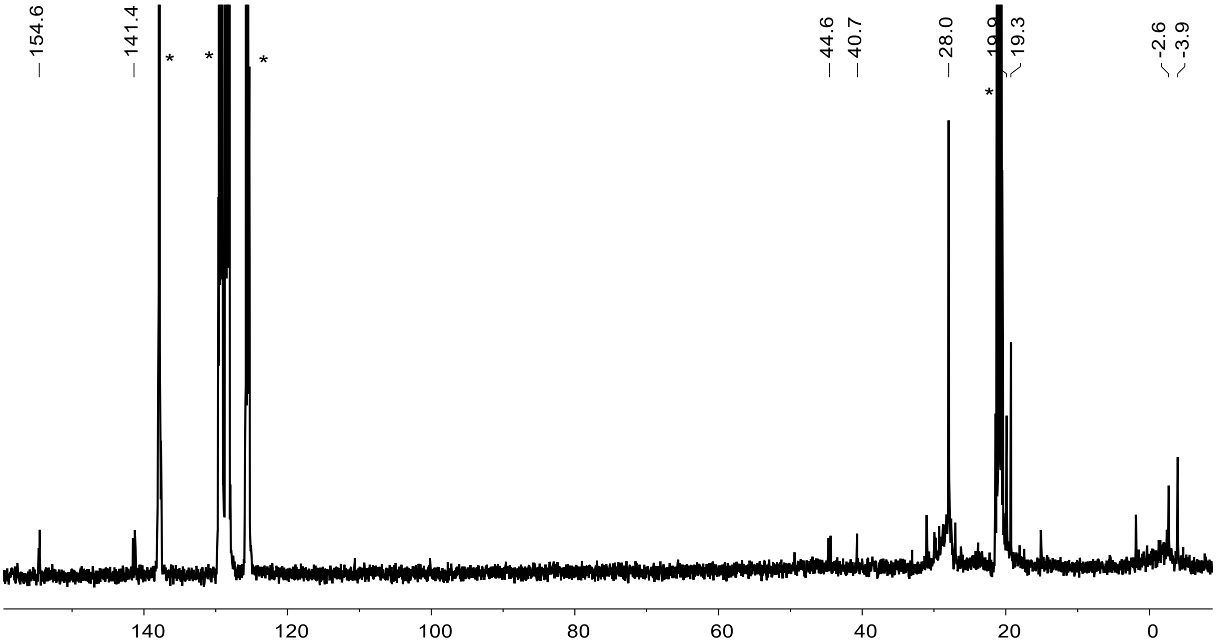

Figure S6. ^{13}C{^{1}H} NMR spectrum (125.7 MHz, 233 K, toluene-d$_8$) of phosphole **19b** (*toluene-d$_8$).

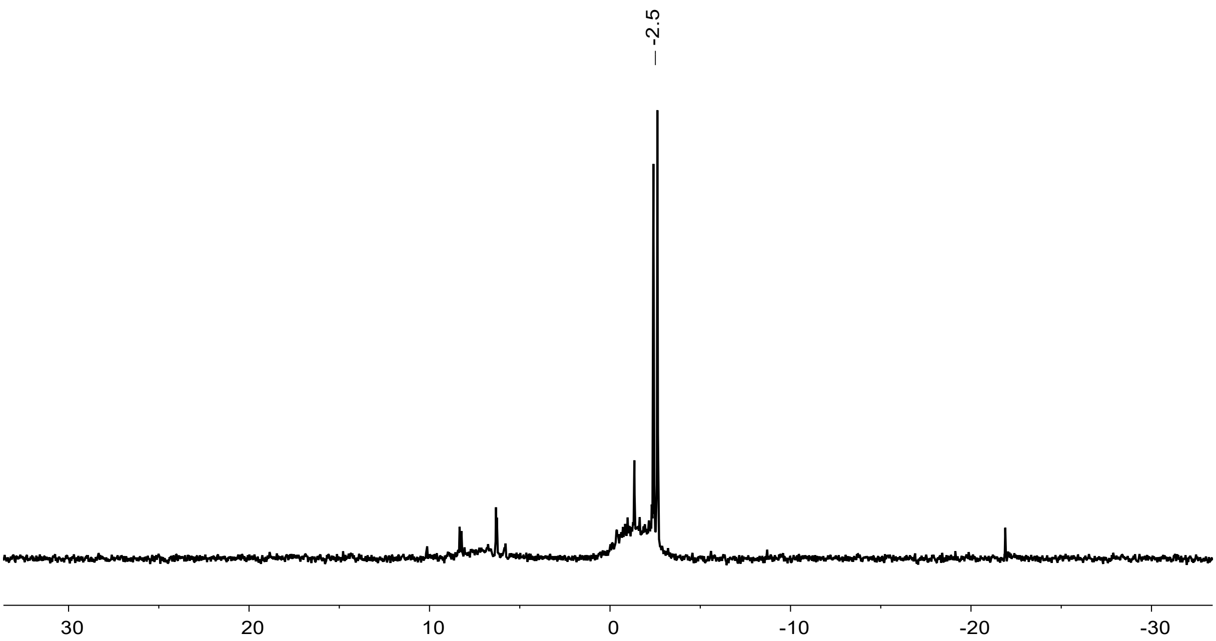

Figure S7. $^{29}Si\{^1H\}$ INEPT NMR spectrum (99.3 MHz, 305 K, benzene-d$_6$) of phosphole **19b**.

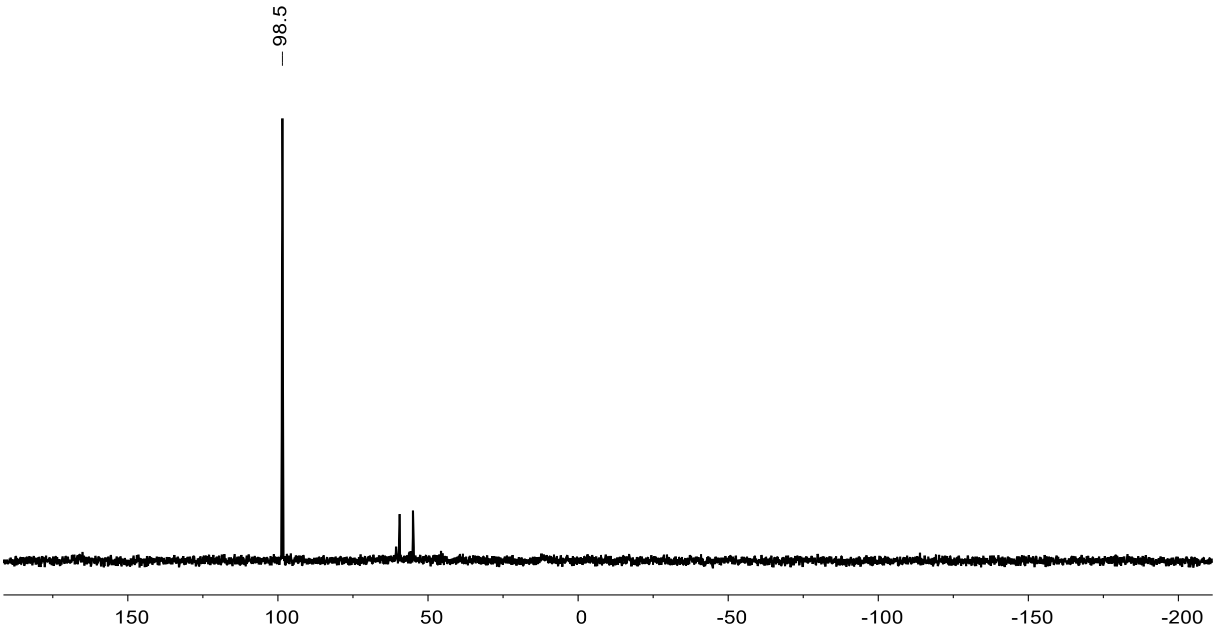

Figure S8. $^{31}P\{^1H\}$ NMR spectrum (202.4 MHz, 305 K, benzene-d$_6$) of phosphole **19b**.

Germylene 15a and Phosphole 20a

The product mixture contained phosphole **20a** and germylene **15a** in a mixture of 30 : 70, in an overall yield of 100%. The residue was redissolved in pentane. Storing the solution at -20°C for several days gave colourless crystals of germylene **15a** suitable for XRD analysis. Spectra recorded immediately after dissolving the crystals already contained trace amounts (3%) of phosphole **20a**.

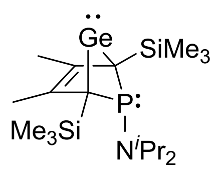

^{1}H NMR (500.1 MHz, 305 K, C_6D_6): δ = 0.29 (s, 18H, 2 x Si$\underline{Me}_3$), 1.13 (d, $^{3}J_{H,H}$ = 6.5 Hz, 12H, N(CH$\underline{Me}_2$)$_2$), 2.19 (s, 6H, 2 x $C^{2/3}$–$\underline{Me}$), 2.83-2.91 (m, 2H, N(C$\underline{H}$Me$_2$)$_2$).
^{13}C{^{1}H} NMR (125.7 MHz, 305 K, C_6D_6): δ = 1.6 (d, $^{3}J_{C,P}$ = 7 Hz, Si$\underline{Me}_3$), 15.7 (d, $^{3}J_{C,P}$ = 4 Hz, $C^{2/3}$–$\underline{Me}$), 25.2 (m, N(CH$\underline{Me}_2$)$_2$), 46.8 (m, N($\underline{C}$HMe$_2$)$_2$), 74.4 (d, $^{1}J_{C,P}$ = 36 Hz, $C^{1/4}$), 129.9 (d, $^{2}J_{C,P}$ = 8 Hz, $C^{2/3}$).
^{29}Si{^{1}H} INEPT NMR (99.3 MHz, 305 K, C_6D_6): δ = -7.6 (d, $^{2}J_{Si,P}$ = 21 Hz).
^{31}P{^{1}H} NMR (202.4 MHz, 305 K, C_6D_6): δ = 35.4.

^{1}H NMR (500.1 MHz, 305 K, THF-d_8): δ = 0.11 (s, 18H, 2 x Si$\underline{Me}_3$), 1.01 (d, $^{3}J_{H,H}$ = 6.6 Hz, 12H, N(CH$\underline{Me}_2$)$_2$), 2.39 (s, 6H, 2 x $C^{2/3}$–$\underline{Me}$), 2.83-2.93 (m, 2H, N(C$\underline{H}$Me$_2$)$_2$).
^{13}C{^{1}H} NMR (125.7 MHz, 305 K, THF-d_8): δ = 1.5 (d, $^{3}J_{C,P}$ = 6 Hz, Si$\underline{Me}_3$), 15.9 (d, $^{3}J_{C,P}$ = 5 Hz, $C^{2/3}$–$\underline{Me}$), 47.3 (m, N($\underline{C}$HMe$_2$)$_2$), 74.6 (d, $^{1}J_{C,P}$ = 36 Hz, $C^{1/4}$), 130.7 (d, $^{2}J_{C,P}$ = 8 Hz, $C^{2/3}$). N(CH$\underline{Me}_2$)$_2$ signal hidden by the solvent signal.
^{29}Si{^{1}H} INEPT NMR (99.3 MHz, 305 K, THF-d_8): δ = -9.3 (d, $^{2}J_{Si,P}$ = 21 Hz).
^{31}P{^{1}H} NMR (202.4 MHz, 305 K, THF-d_8): δ = 33.3.

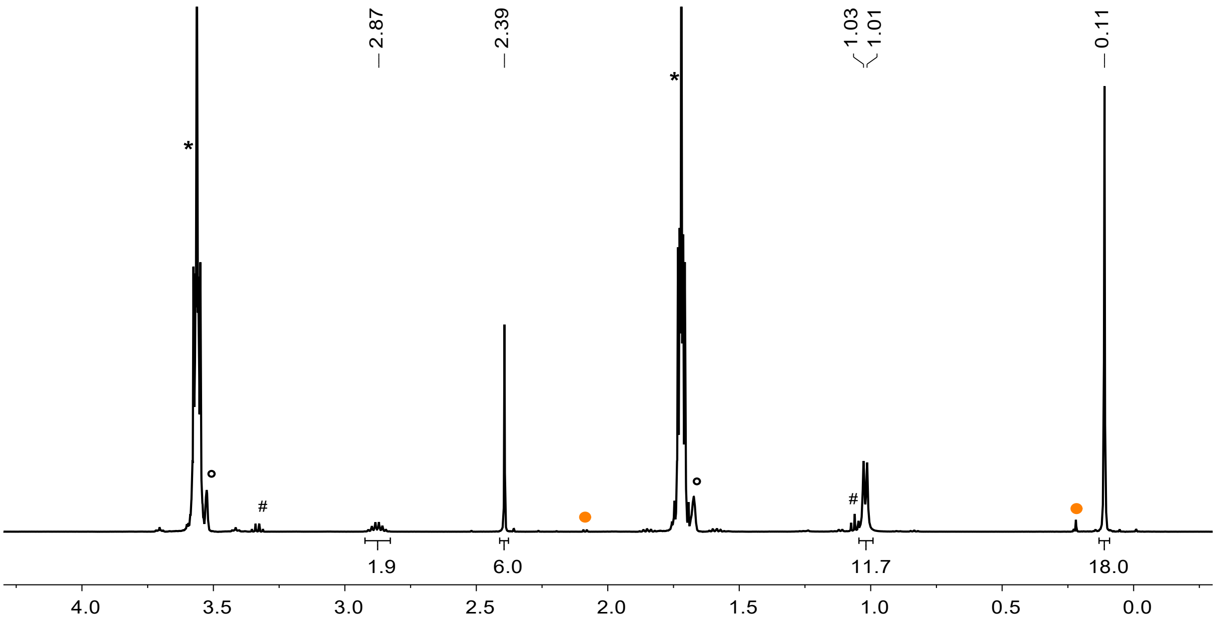

Figure S9. ^{1}H NMR spectrum (500.1 MHz, 305 K, THF-d_8) of dissolved crystals of germylene **15a** (*THF-d_8, °THF, #pentane, •phosphole **20a**).

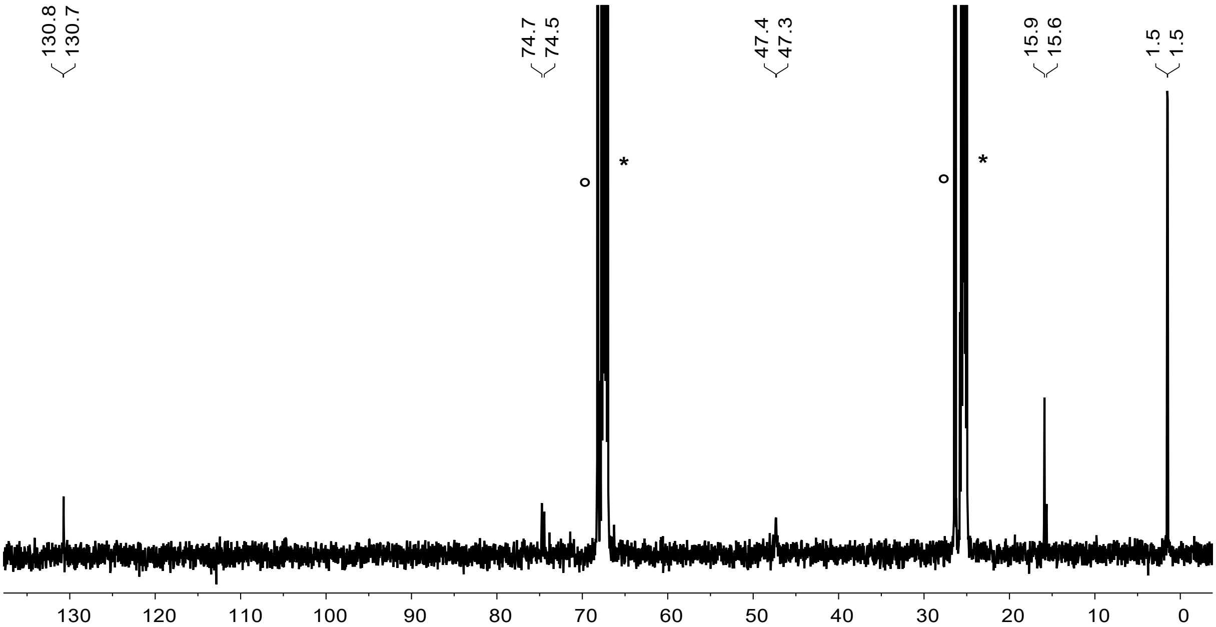

Figure S10. $^{13}C\{^{1}H\}$ NMR spectrum (125.7 MHz, 305 K, THF-d_8) of dissolved crystals of germylene **15a** (*THF-d_8, °THF).

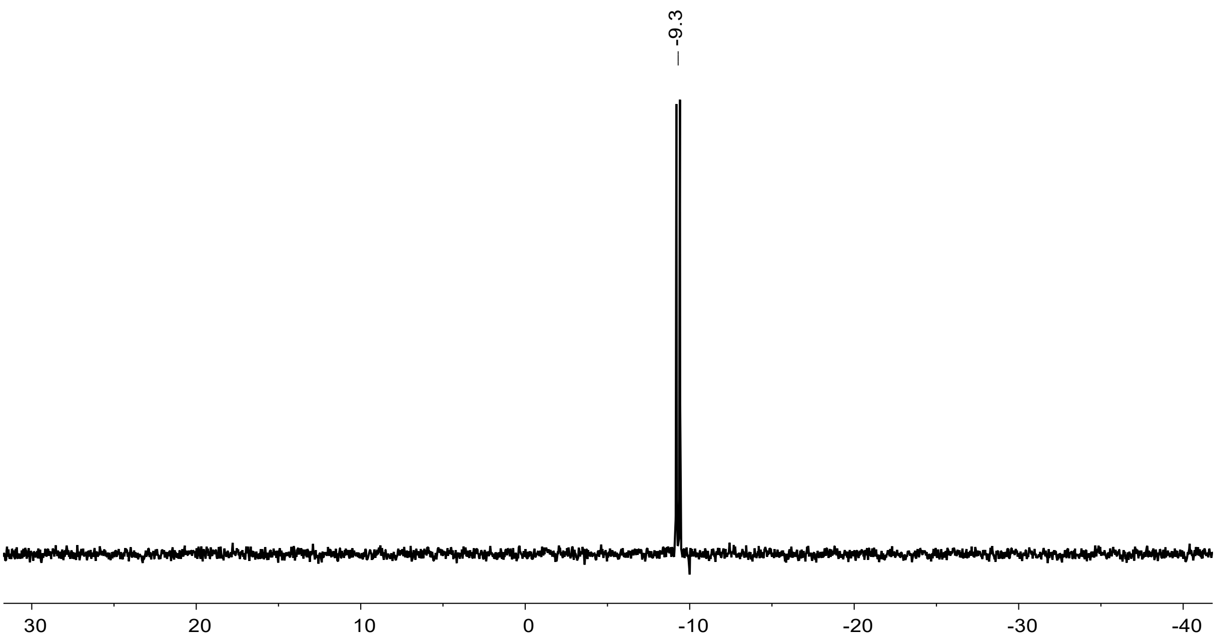

Figure S11. $^{29}Si\{^{1}H\}$ INEPT NMR spectrum (99.3 MHz, 305 K, THF-d_8) of dissolved crystals of germylene **15a**.

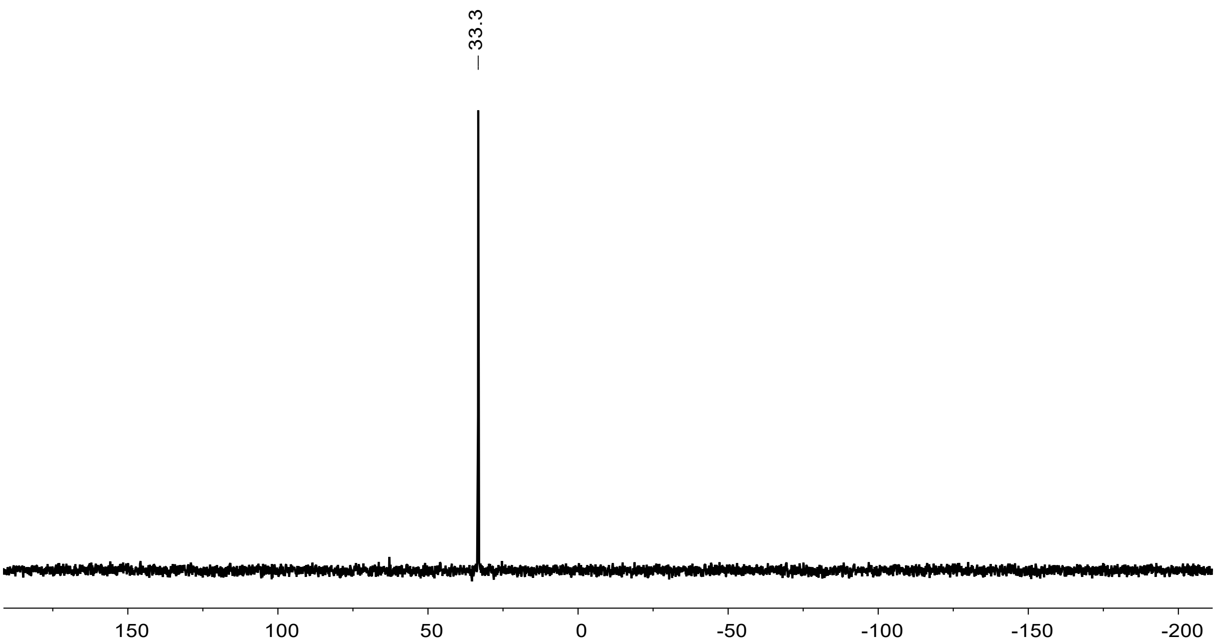

Figure S12. $^{31}P\{^1H\}$ NMR spectrum (202.4 MHz, 305 K, THF-d_8) of dissolved crystals of germylene **15a**.

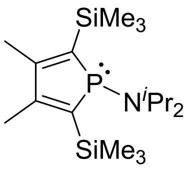

^{1}H NMR (500.1 MHz, 305 K, C_6D_6): δ = 0.40 (s, 18 H, 2 x Si<u>Me</u>$_3$), 1.18 (d, $^3J_{H,H}$ = 6.6 Hz, 12 H, N(CH<u>Me</u>$_2$)$_2$), 2.02 (d, $^4J_{H,H}$ = 6.5 Hz, 6 H, 2 x $C^{2/3}$–<u>Me</u>), 3.05-3.15 (m, 2 H, N(C<u>H</u>Me$_2$)$_2$).

^{13}C{^{1}H} NMR (125.7 MHz, 305 K, C_6D_6): δ = 1.4 (d, $^3J_{C,P}$ = 3 Hz, Si<u>Me</u>$_3$), 18.1 (d, $^3J_{C,P}$ = 6 Hz, $C^{2/3}$–<u>Me</u>), 24.4 (d, $^3J_{C,P}$ = 8 Hz, N(CH<u>Me</u>$_2$)$_2$), 51.1 (d, $^2J_{C,P}$ = 8 Hz, N(<u>C</u>HMe$_2$)$_2$), 143.0 (d, $^1J_{C,P}$ = 33 Hz, $C^{1/4}$), 151.9 (d, $^2J_{C,P}$ = 19 Hz, $C^{2/3}$).

^{29}Si{^{1}H} INEPT NMR (99.3 MHz, 305 K, C_6D_6): δ = -10.8 (d, $^2J_{Si,P}$ = 25 Hz).

^{31}P{^{1}H} NMR (202.4 MHz, 305 K, C_6D_6): δ = 65.6.

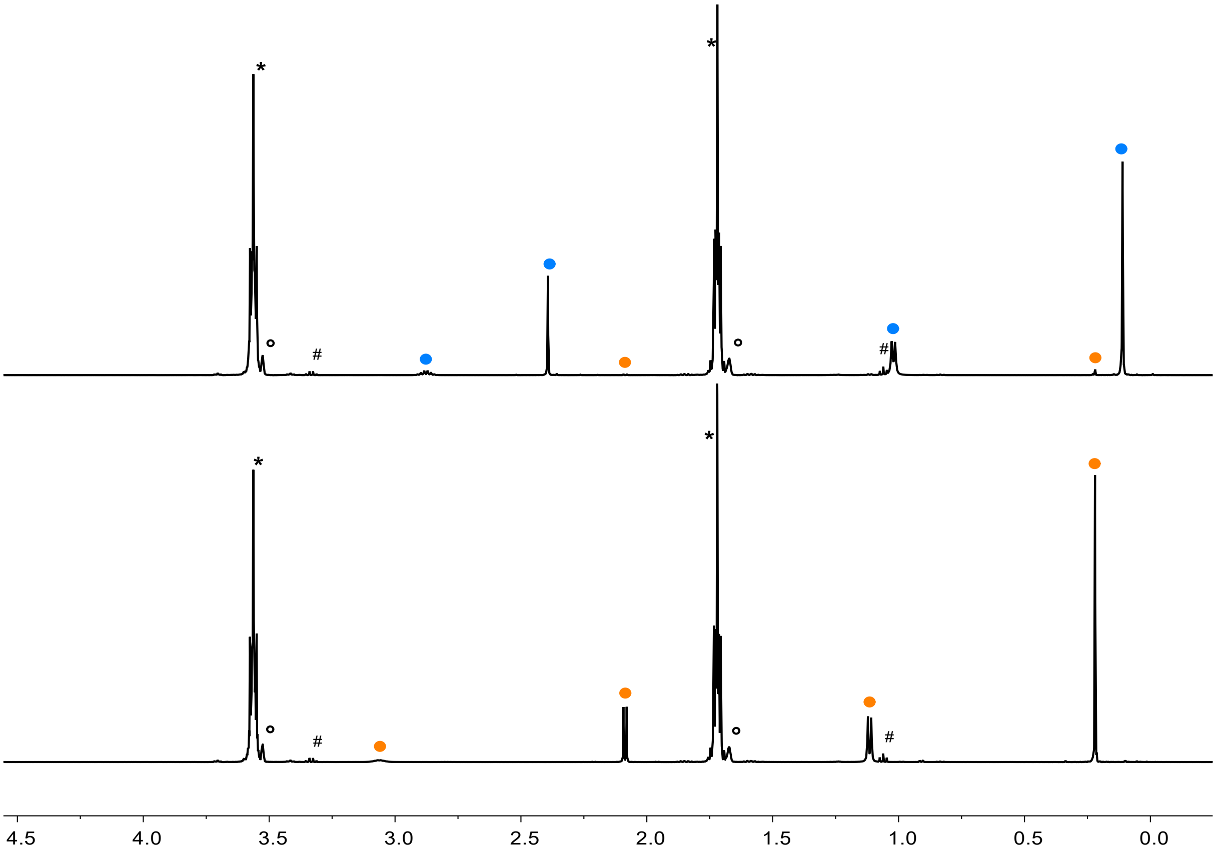

Figure S13. ^{1}H NMR spectra (500.1 MHz, 305 K, THF-d_8) of dissolved crystals of germylene **15a** (top). Bottom: phosphole **20a**, after precipitation of elemental germanium (*THF-d_8, °THF, #pentane).

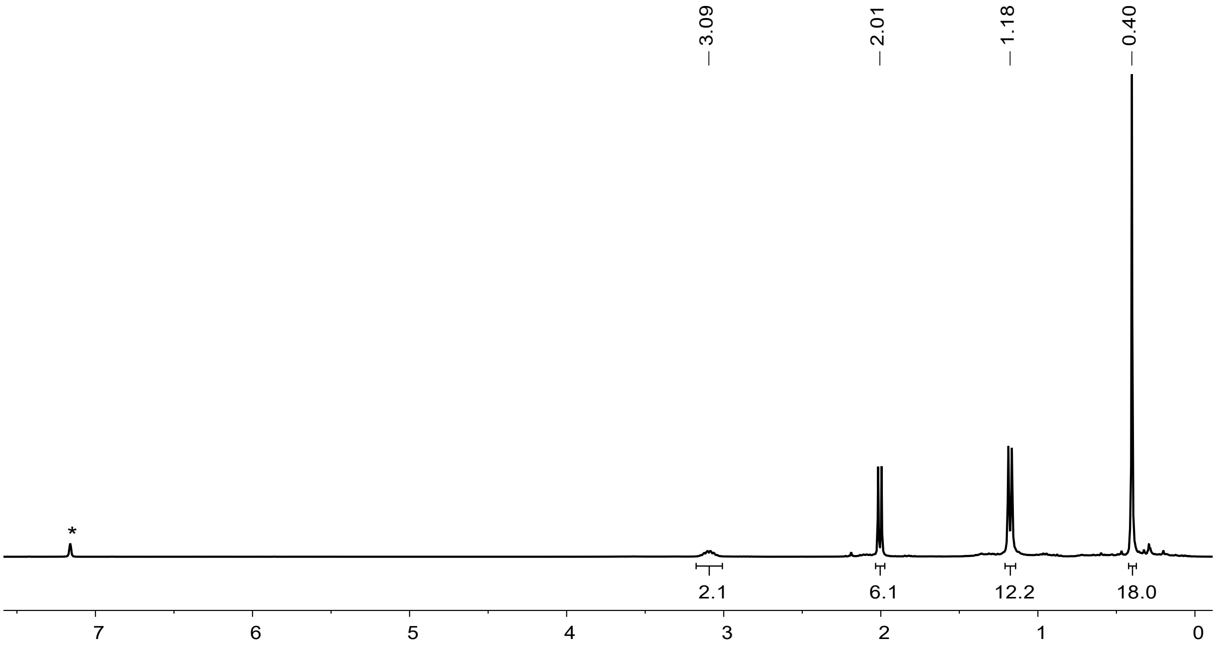

Figure S14. ^{1}H NMR spectrum (500.1 MHz, 305 K, benzene-d_6) of phosphole **20a** (*C_6D_5H).

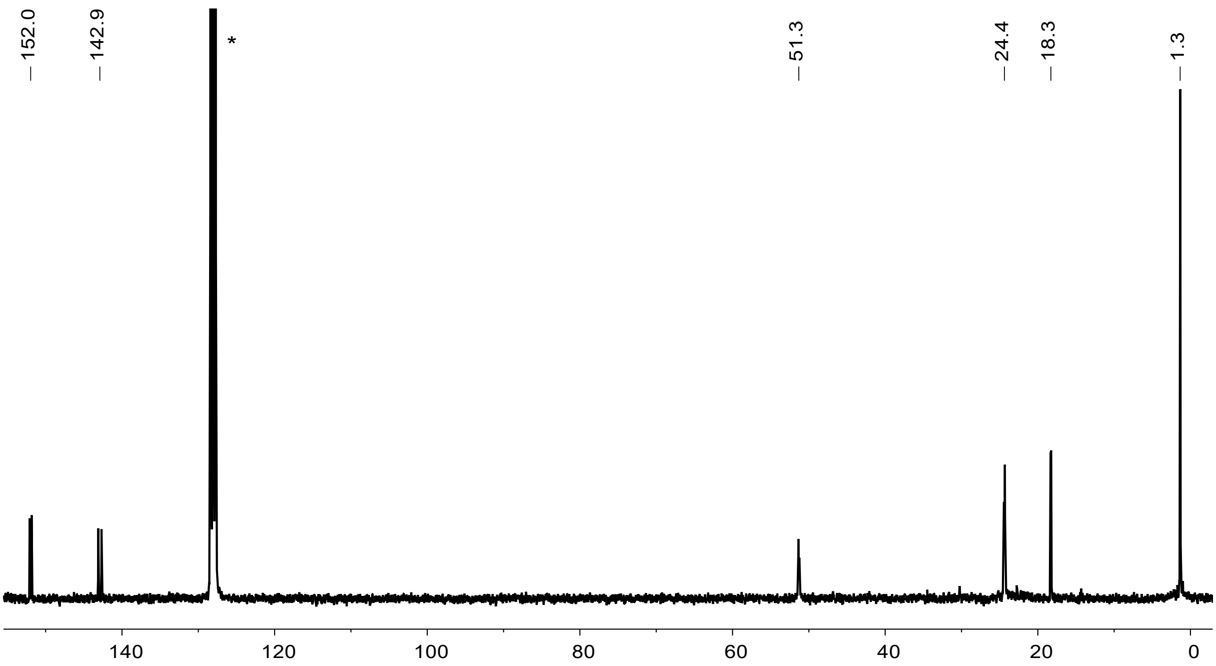

Figure S15. $^{13}C\{^1H\}$ NMR spectrum (125.7 MHz, 305 K, benzene-d_6) of phosphole **20a** (*C_6D_6).

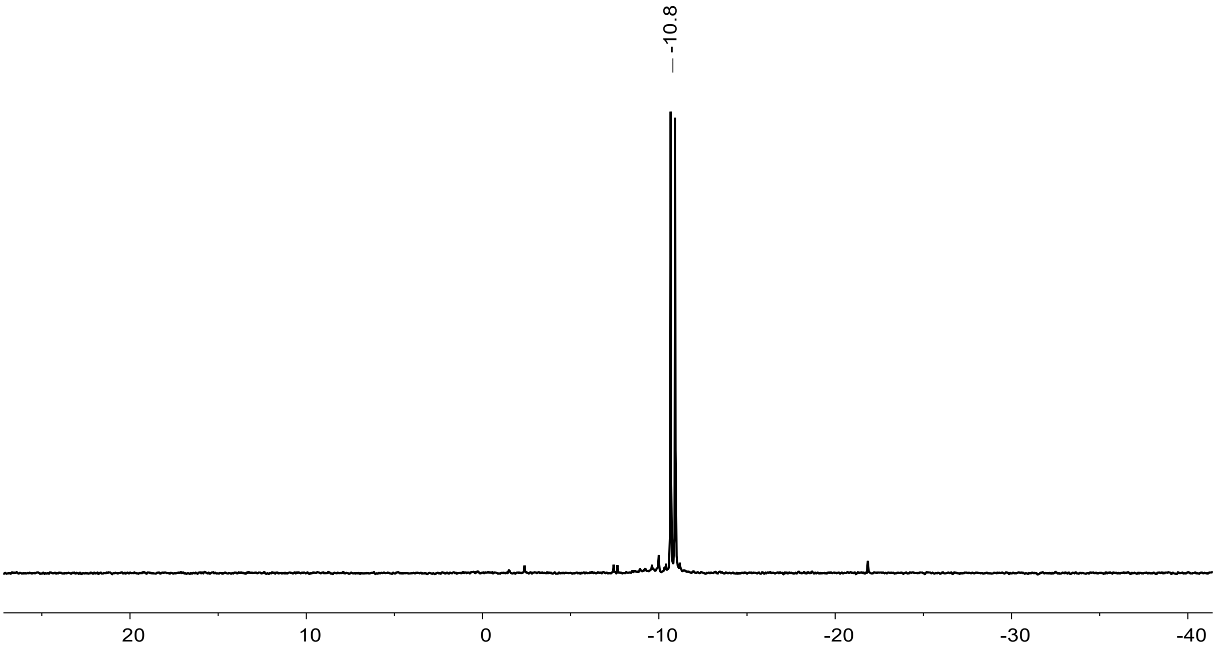

Figure S16. $^{29}Si\{^1H\}$ INEPT NMR spectrum (99.3 MHz, 305 K, benzene-d_6) of phosphole **20a**.

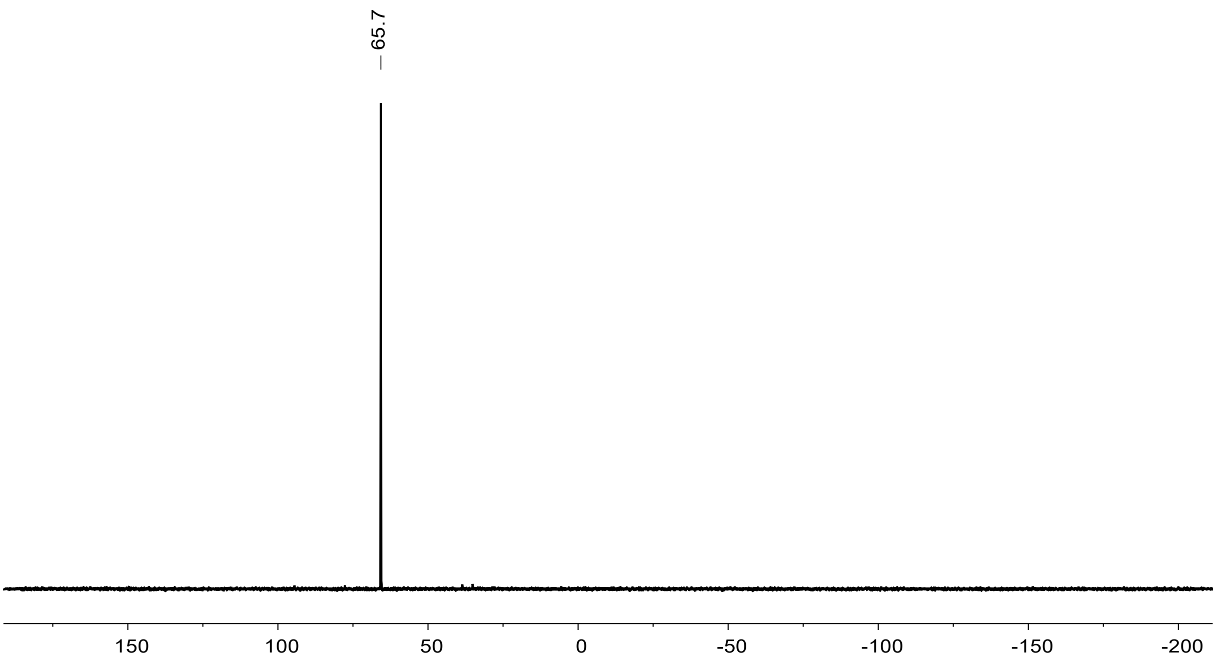

Figure S17. $^{31}P\{^1H\}$ NMR spectrum (202.4 MHz, 305 K, benzene-d_6) of phosphole **20a**.

Germylene 15b and Phosphole 20b

The product mixture contained phosphole **20b** and germylene **15b** in a mixture of 3 : 97, in an overall yield of 79%.

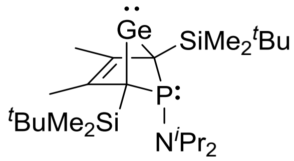

^{1}H NMR (500.1 MHz, 305 K, C_6D_6): δ = 0.21 (s, 6H, 2 x Si<u>Me</u>$_2$^{t}Bu), 0.32–0.34 (m, 6H, 2 x Si<u>Me</u>$_2$^{t}Bu), 1.11 (s, 18H, 2 x SiMe$_2$<u>^{t}Bu</u>), 1.13 (d, $^3J_{H,H}$ = 6.8 Hz, 12H, N(CH<u>Me</u>$_2$)$_2$), 2.23 (s, 6H, 2 x C<u>H</u>$_3$), 3.14–3.25 (m, 2H, N(C<u>H</u>Me$_2$)$_2$).

$^{13}C\{^1H\}$ NMR (125.7 MHz, 305 K, C_6D_6): δ = -2.5 (d, $^3J_{C,P}$ = 5 Hz, Si(<u>Me</u>$_2$)^{t}Bu), -2.1 (d, $^3J_{C,P}$ = 5 Hz, Si(<u>Me</u>$_2$)^{t}Bu), 17.1 (d, $^3J_{C,P}$ = 4 Hz, $C^{2/3}$–<u>Me</u>), 19.0 (d, $^4J_{C,P}$ = 2 Hz, Si(Me$_2$)<u>^{t}Bu</u>), 25.4 (d, $^3J_{C,P}$ = 5 Hz, N(CH<u>Me</u>$_2$)$_2$), 28.4 (d, $^3J_{C,P}$ = 3 Hz, Si(Me$_2$)<u>^{t}Bu</u>), 48.7 (d, $^2J_{C,P}$ = 10 Hz, N(<u>C</u>HMe$_2$)$_2$), 72.8 (d, $^1J_{C,P}$ = 41 Hz, $C^{1/4}$), 131.1 (d, $^2J_{C,P}$ = 7 Hz, $C^{2/3}$).

$^{29}Si\{^1H\}$ INEPT NMR (99.3 MHz, 305 K, C_6D_6): δ = -0.2 (d, $^2J_{Si,P}$ = 18 Hz).

$^{31}P\{^1H\}$ NMR (202.4 MHz, 305 K, C_6D_6): δ = 53.8.

MS (EI): m/z (%) = 73 (100), 283 (21), 383 (10), 413 (10), 514 (2) [M+H].

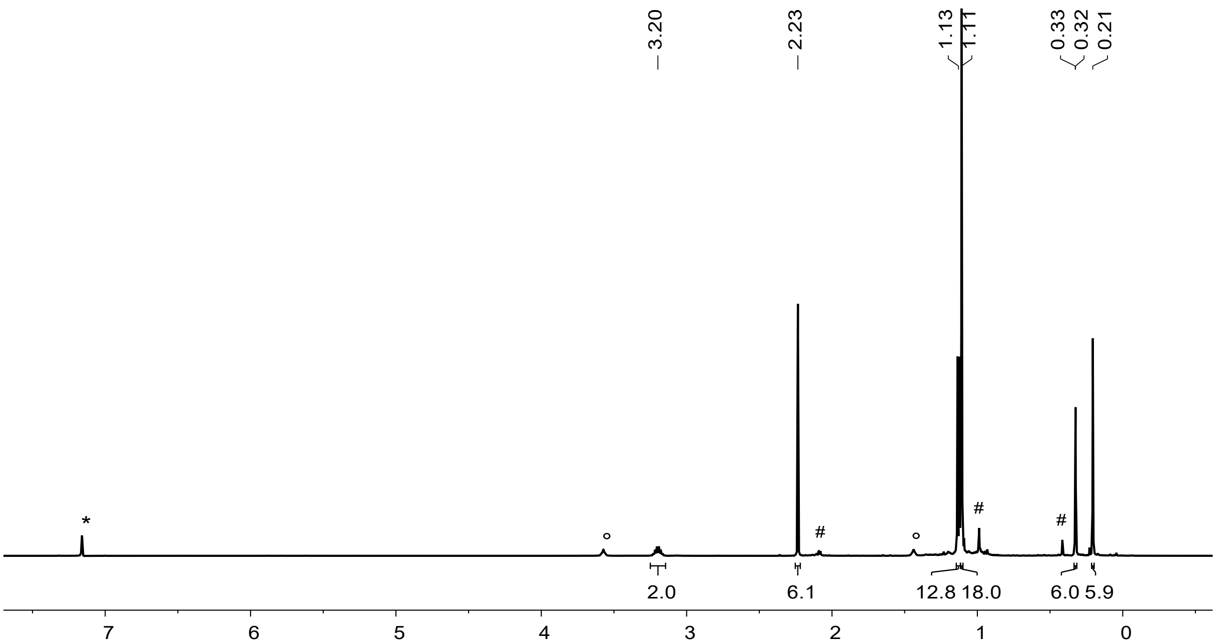

Figure S18. ^{1}H NMR spectrum (500.1 MHz, 305 K, benzene-d_6) of germylene **15b** (*C_6D_5H, °THF, #phosphole **20b**).

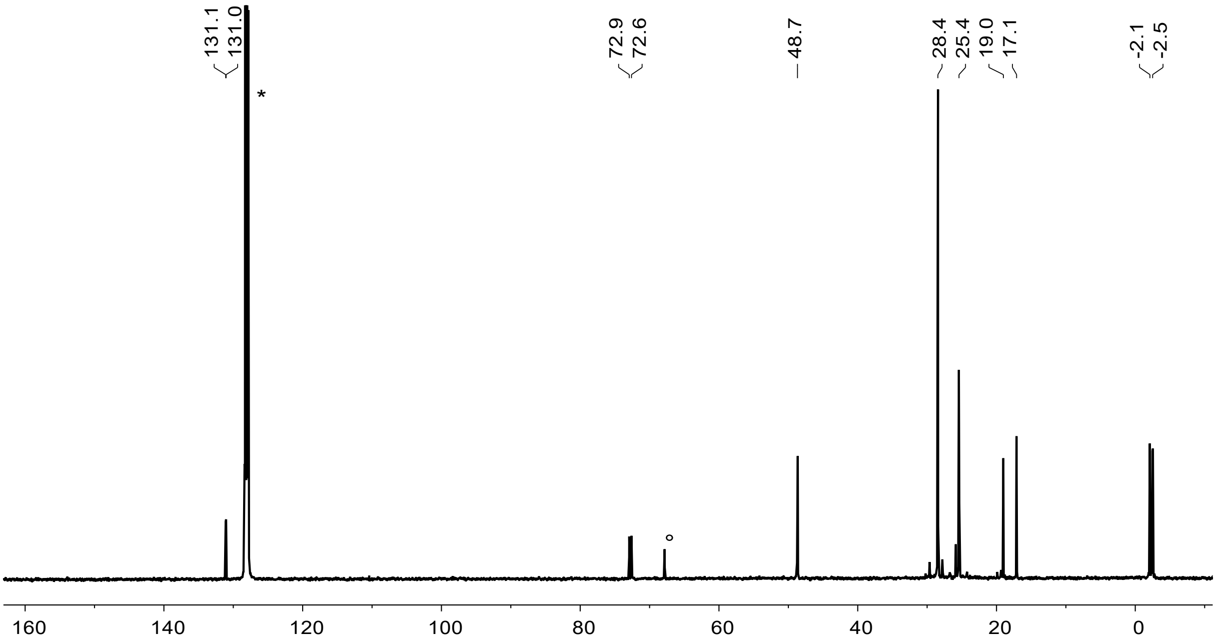

Figure S19. $^{13}C\{^{1}H\}$ NMR spectrum (125.7 MHz, 305 K, benzene-d_6) of germylene **15b** (*C_6D_6, °impurity).

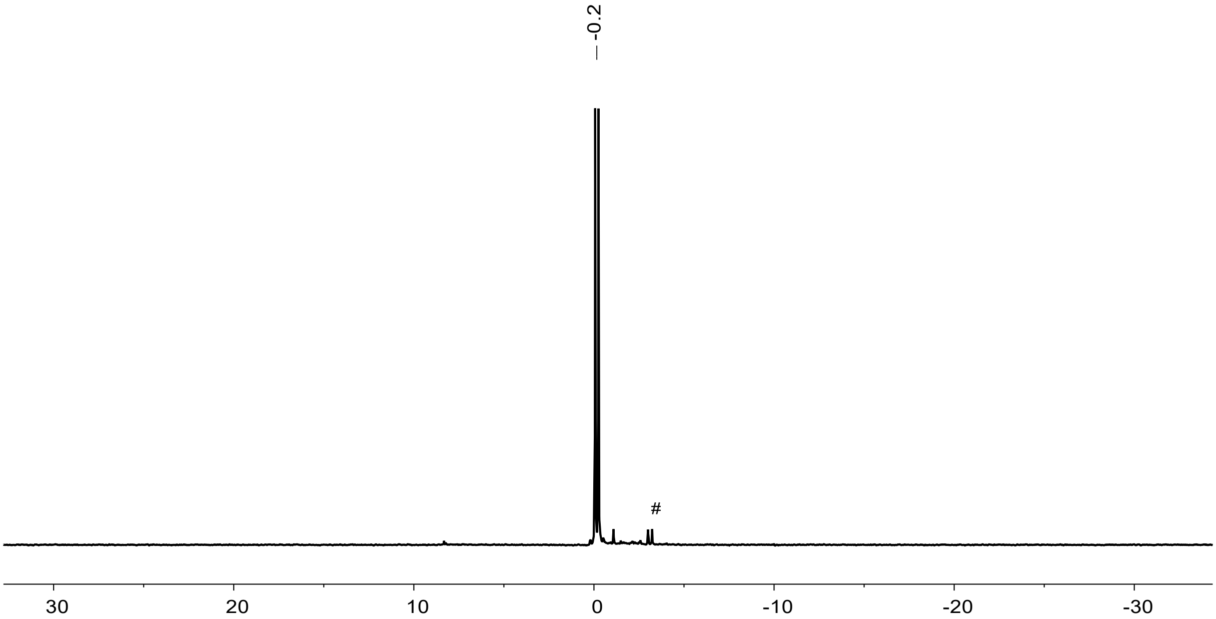

Figure S20. ^{29}Si{^{1}H} INEPT NMR spectrum (99.3 MHz, 305 K, benzene-d$_6$) of germylene **15b** ($^{\#}$phosphole **20b**).

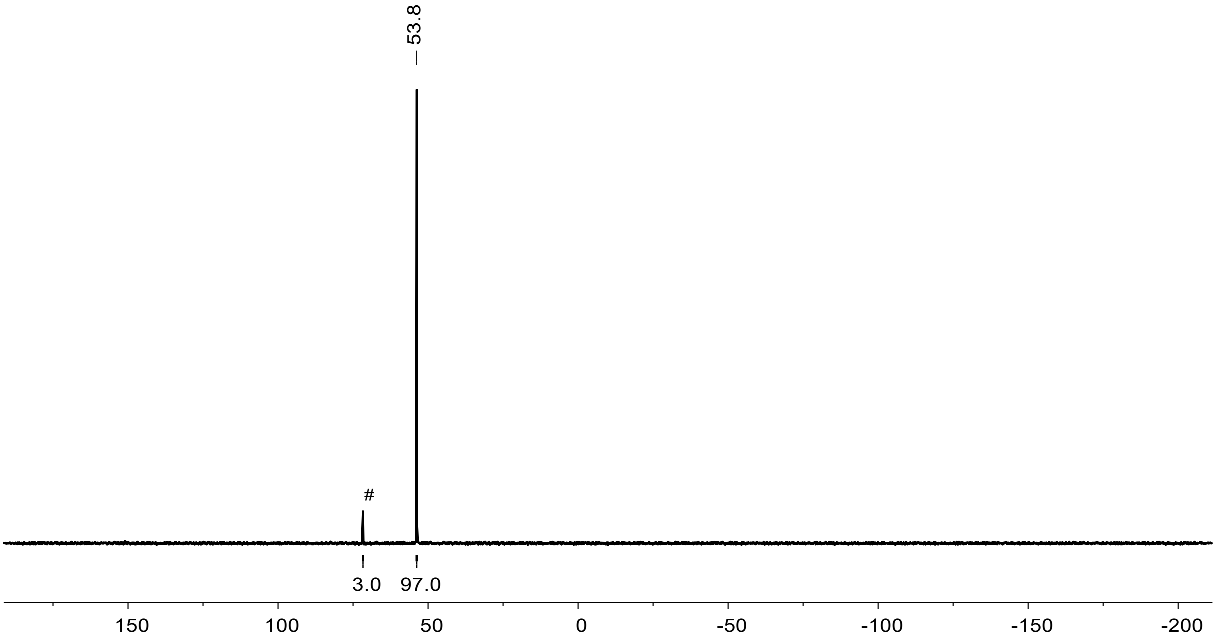

Figure S21. ^{31}P{^{1}H} NMR spectrum (202.4 MHz, 305 K, benzene-d$_6$) of germylene **15b** ($^{\#}$phosphole **20b**).

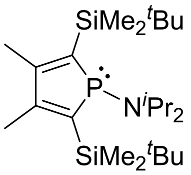

^{1}H NMR (500.1 MHz, 305 K, C$_6$D$_6$): δ = 0.42 (s, 12H, 2 x Si$\underline{Me_2}$^{t}Bu), 0.98 (s, 18H, 2 x SiMe$_2$$\underline{^tBu}$), 1.19 (d, $^3J_{H,H}$ = 6.4 Hz, 12H, N(CH$\underline{Me_2}$)$_2$), 2.09 (d, $^4J_{H,P}$ = 6.3 Hz, 6H, 2 x C$^{2/3}$–$\underline{Me}$), 3.02-3.13 (m, 2H, N(C$\underline{H}$Me$_2$)$_2$).
^{13}C{^{1}H} NMR (125.7 MHz, 305 K, C$_6$D$_6$): δ = -2.6 (d, $^3J_{C,P}$ = 4 Hz, Si$\underline{Me_2}$^{t}Bu), 19.3 (SiMe$_2$$\underline{^tBu}$), 19.9 (d, $^3J_{C,P}$ = 6 Hz, C$^{2/3}$–$\underline{Me}$), 24.1–24.5 (m, N(CH$\underline{Me_2}$)$_2$), 27.8 (d, $^3J_{C,P}$ = 3 Hz, SiMe$_2$$\underline{^tBu}$), 50.8 (br, N($\underline{C}HMe_2$)$_2$), 140.7 (d, $^1J_{C,P}$ = 36 Hz, C$^{1/4}$), 154.0 (d, $^2J_{C,P}$ = 16 Hz, C$^{2/3}$).
^{29}Si{^{1}H} INEPT NMR (99.3 MHz, 305 K, C$_6$D$_6$): δ = -3.1 (d, $^2J_{Si,P}$ = 22 Hz).
^{31}P{^{1}H} NMR (202.4 MHz, 305 K, C$_6$D$_6$): δ = 71.7 ($^2J_{P,Si}$ = 22 Hz).

MS (EI): m/z (%) = 73 (100), 149 (40), 283 (84), 413 (75), 439 (10) [M+].

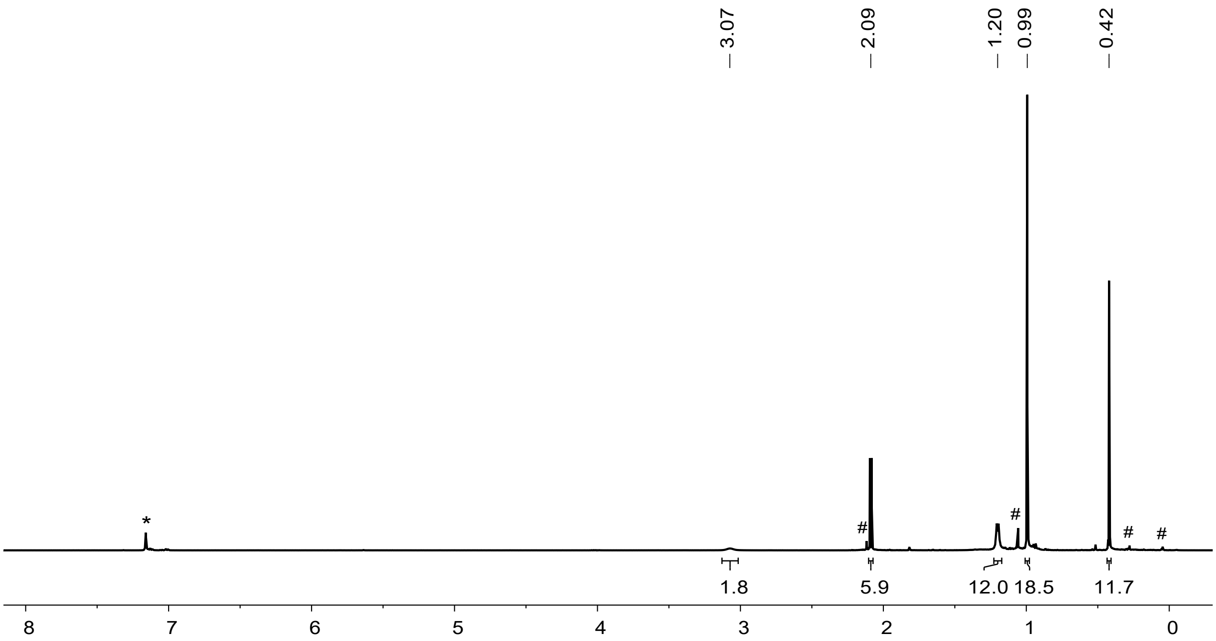

Figure S22. ^{1}H NMR spectrum (500.1 MHz, 305 K, benzene-d_6) of phosphole **20b** (*C_6D_5H, #germylene **15b**).

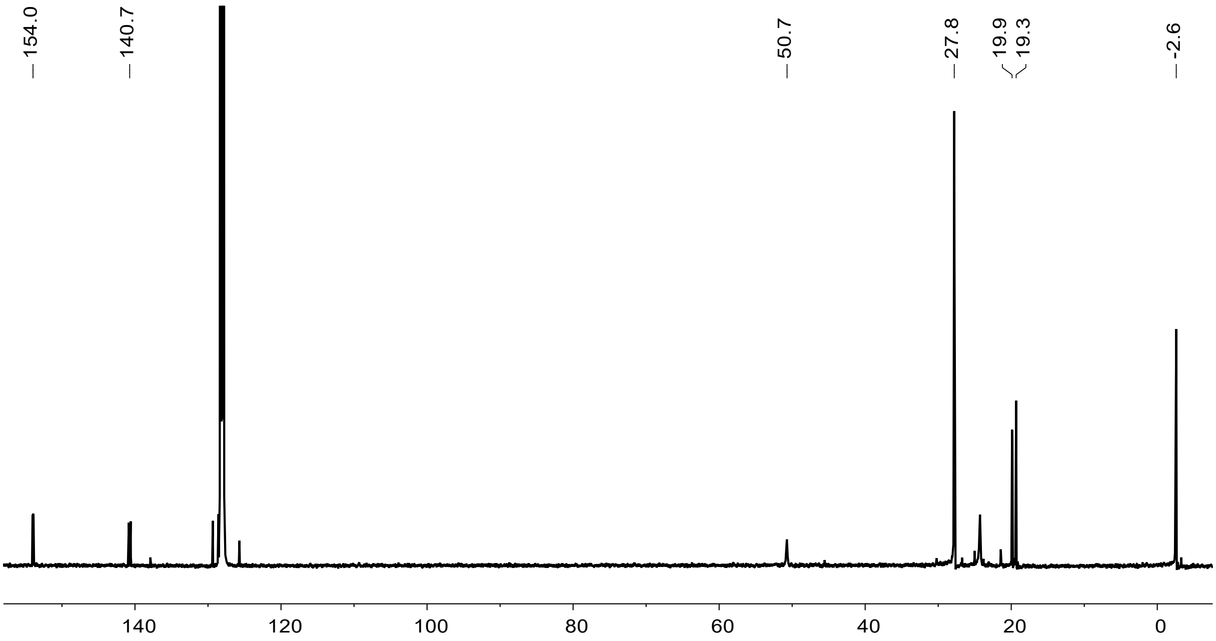

Figure S23. $^{13}C\{^{1}H\}$ NMR spectrum (125.7 MHz, 305 K, benzene-d_6) of phosphole **20b** (*C_6D_6).

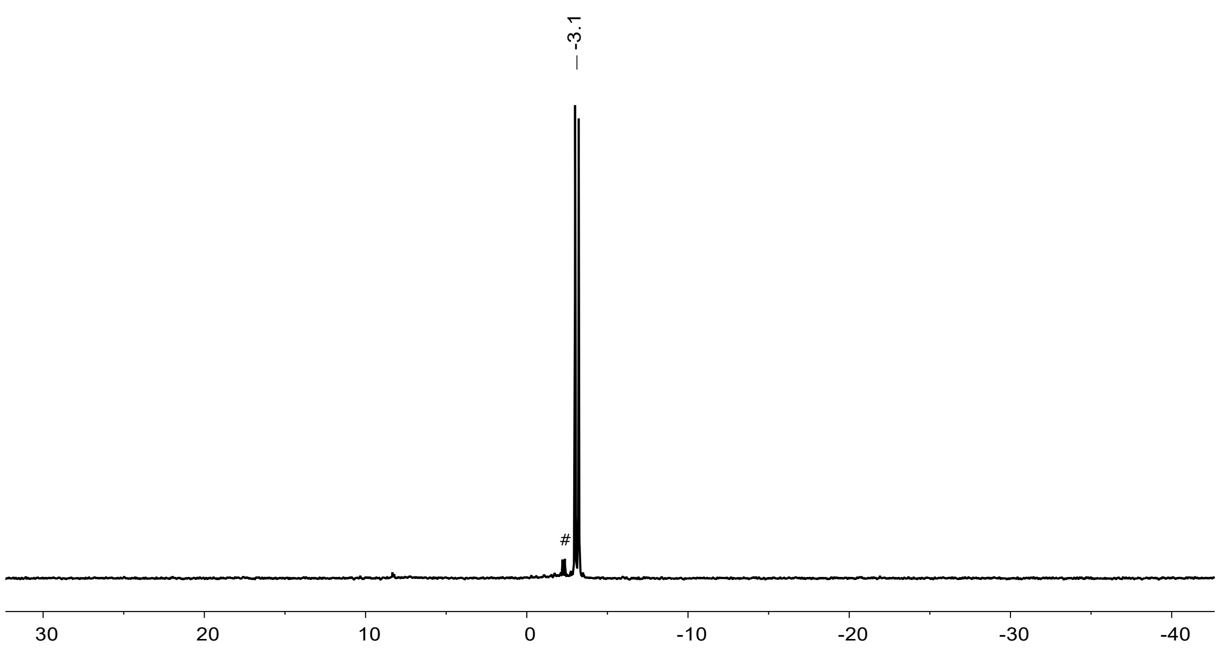

Figure S24. $^{29}Si\{^1H\}$ INEPT NMR spectrum (99.3 MHz, 305 K, benzene-d$_6$) of phosphole **20b** ($^{\#}$germylene **15b**).

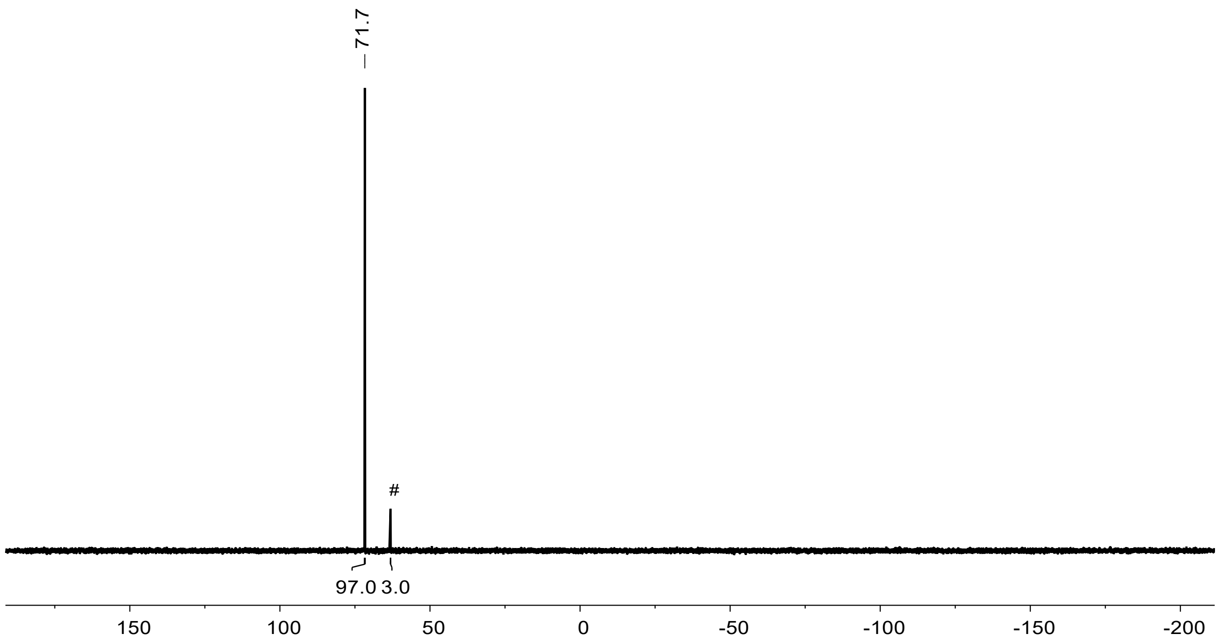

Figure S25. $^{31}P\{^1H\}$ NMR spectrum (202.4 MHz, 305 K, benzene-d$_6$) of phosphole **20b** ($^{\#}$germylene **15b**).

Germylene 16a and Phosphole 21a

The product mixture contained phosphole **21a** and germylene **16a** in a mixture of 25 : 75, in an overall yield of 95%. Colourless crystals, suitable for X-ray diffraction, of phosphole **21a** were obtained upon keeping a pentane solution of the reaction mixture at -30°C.

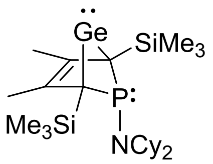

^{1}H NMR (500.1 MHz, 305 K, C_6D_6): δ = 0.39 (s, 18H, 2 x Si<u>Me</u>$_3$), 2.34 (s, 6H, 2 x $C^{2/3}$–<u>Me</u>), 2.49 (br, 2H, NCy_2-C^{ipso}-<u>H</u>). Cyclohexyl signals cannot be assigned due to overlap with the phosphole signals.

^{13}C{^{1}H} NMR (125.7 MHz, 305 K, C_6D_6): δ = 1.6 (d, $^{3}J_{C,P}$ = 6 Hz, Si<u>Me</u>$_3$), 15.7 (d, $^{3}J_{C,P}$ = 4 Hz, $C^{2/3}$–<u>Me</u>), 25.5(N<u>Cy</u>$_2$), 26.2(N<u>Cy</u>$_2$), 35.0 (N<u>Cy</u>$_2$), 53.2 (NCy_2, <u>C</u>^{ipso}), 74.8 (d, $^{1}J_{C,P}$ = 37 Hz, $C^{1/4}$), 129.9 (d, $^{2}J_{C,P}$ = 8 Hz, $C^{2/3}$).

^{29}Si{^{1}H} NMR (99.3 MHz, 305 K, C_6D_6): δ = -7.6 (d, $^{2}J_{Si,P}$ = 21 Hz).

^{31}P{^{1}H} NMR (202.4 MHz, 305 K, C_6D_6): δ = 39.4.

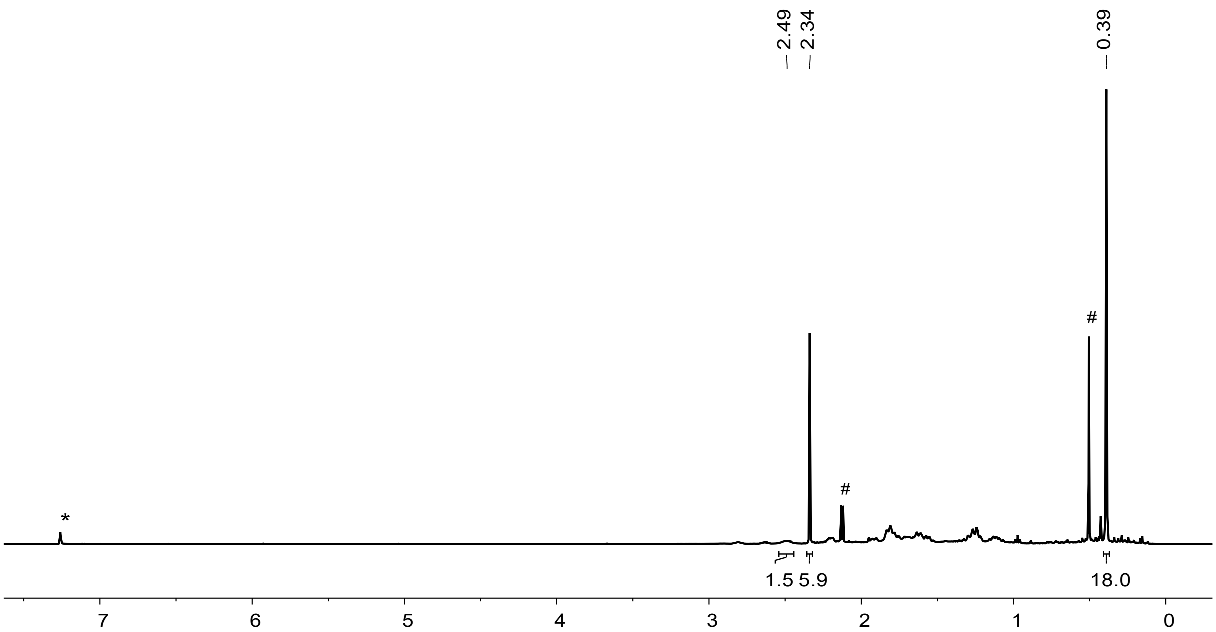

Figure S26. ^{1}H NMR spectrum (500.1 MHz, 305 K, benzene-d_6) of germylene **16a** (*C_6D_5H, #phosphole **21a**).

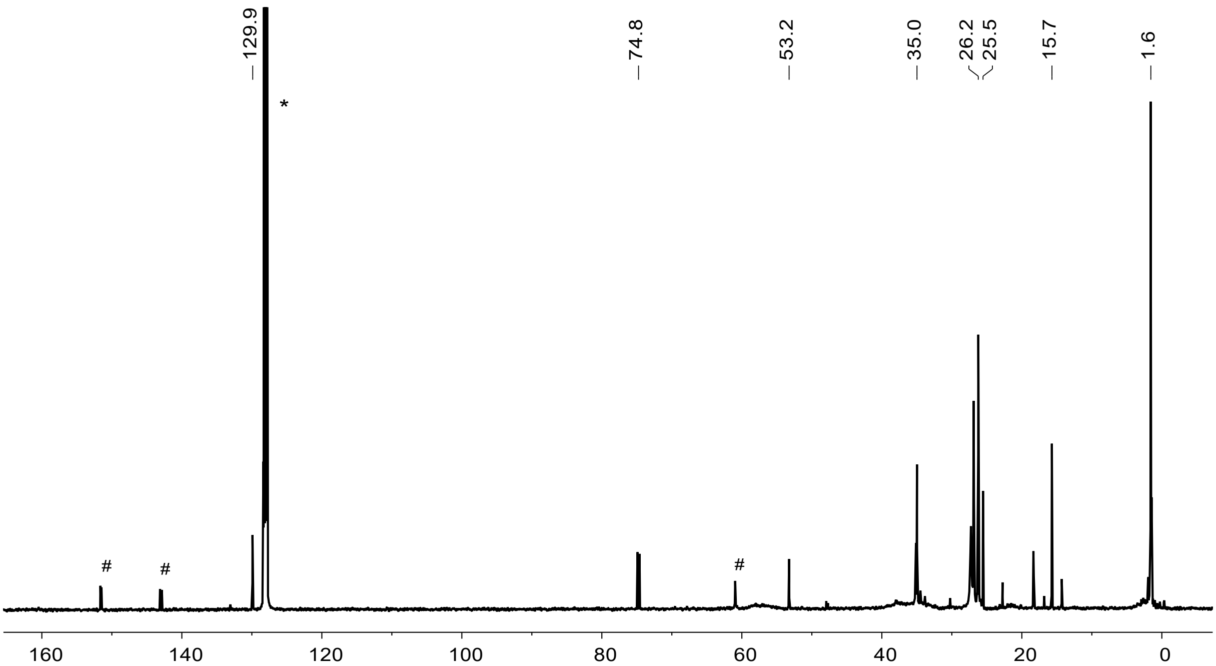

Figure S27. ^{13}C{^{1}H} NMR spectrum (125.7 MHz, 305 K, benzene-d_6) of germylene **16a** (*C_6D_6, # phosphole **21a**).

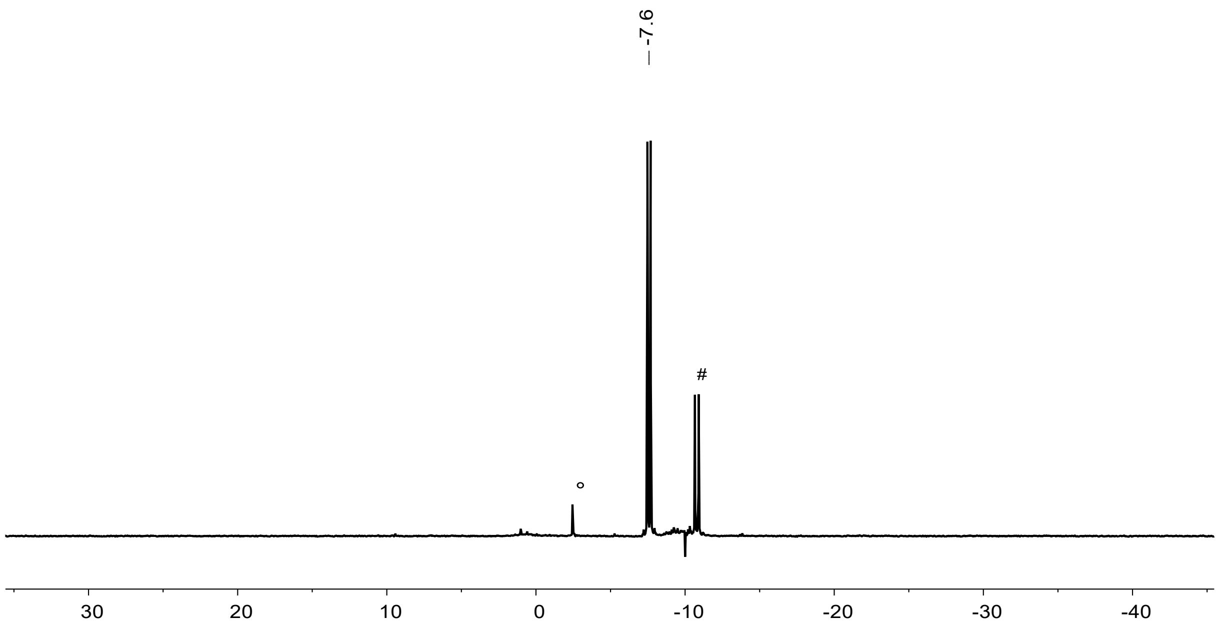

Figure S28. $^{29}Si\{^{1}H\}$ INEPT NMR spectrum (99.3 MHz, 305 K, benzene-d_6) of germylene **16a** ($^{\#}$phosphole **21a**, ° impurity).

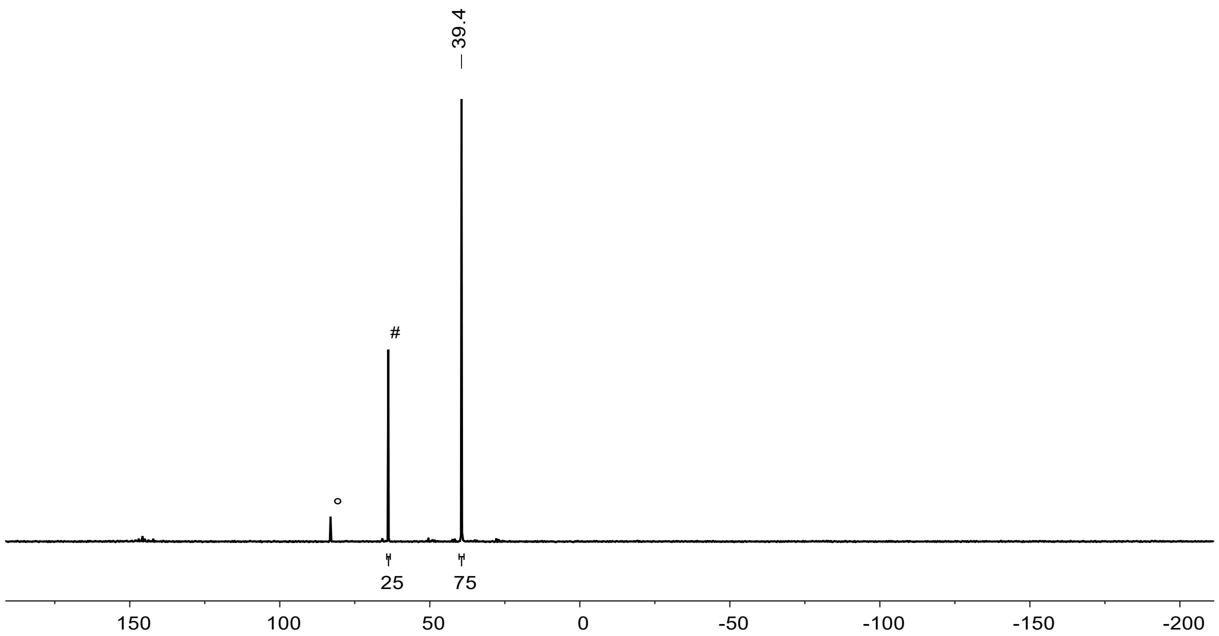

Figure S29. $^{31}P\{^{1}H\}$ NMR spectrum (202.4 MHz, 305 K, benzene-d_6) of germylene **16a** ($^{\#}$phosphole **21a**, ° impurity).

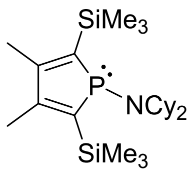

^{1}H NMR (500.1 MHz, 305 K, C_6D_6): δ = 0.43 (s, 18H, 2 x Si<u>Me</u>$_3$), 0.99 (qt, $J_{H,H}$ = 13.1 Hz, $J_{H,H}$ = 3.6 Hz, 2 H, Cy), 1.16 (qt, $J_{H,H}$ = 13.1 Hz, $J_{H,H}$ = 3.3 Hz, 4 H, Cy), 1.43-1.54 (m, 6 H, Cy), 1.66-1.73 (m, 4 H, Cy), 2.04 (d, $^{4}J_{H,P}$ = 6.6 Hz, 6H, 2 x $C^{2/3}$–<u>Me</u>), 2.10-2.16 (m, 4 H, Cy), 2.69-2.78 (m, 2 H, Cy, 2 x <u>H</u>C^{ipso}).

$^{13}C\{^{1}H\}$ NMR (125.7 MHz, 305 K, C_6D_6): δ = 1.5 (d, $^{3}J_{C,P}$ = 3 Hz, Si<u>Me</u>$_3$), 18.3 (d, $^{3}J_{C,P}$ = 6 Hz, $C^{2/3}$–<u>Me</u>), 26.3 (Cy), 26.9 (Cy), 35.2 (d, 8 Hz, Cy), 60.9 (d, $^{2}J_{C,P}$ = 6 Hz, Cy, C^{ipso}), 143.0 (d, $^{2}J_{C,P}$ = 33 Hz, $C^{1/4}$), 151.6 (d, $^{2}J_{C,P}$ = 20 Hz, $C^{2/3}$).

$^{29}Si\{^{1}H\}$ INEPT NMR (99.3 MHz, 305 K, C_6D_6): δ = -10.8 (d, $^{2}J_{Si,P}$ = 25 Hz).

^{31}P{^{1}H} NMR (202.4 MHz, 305 K, C_6D_6): δ = 63.8.
MS (EI): m/z (%) = 73 (100), 168 (70), 180 (52), 256 (55), 362 (65), 435 (100) [M^{+}].

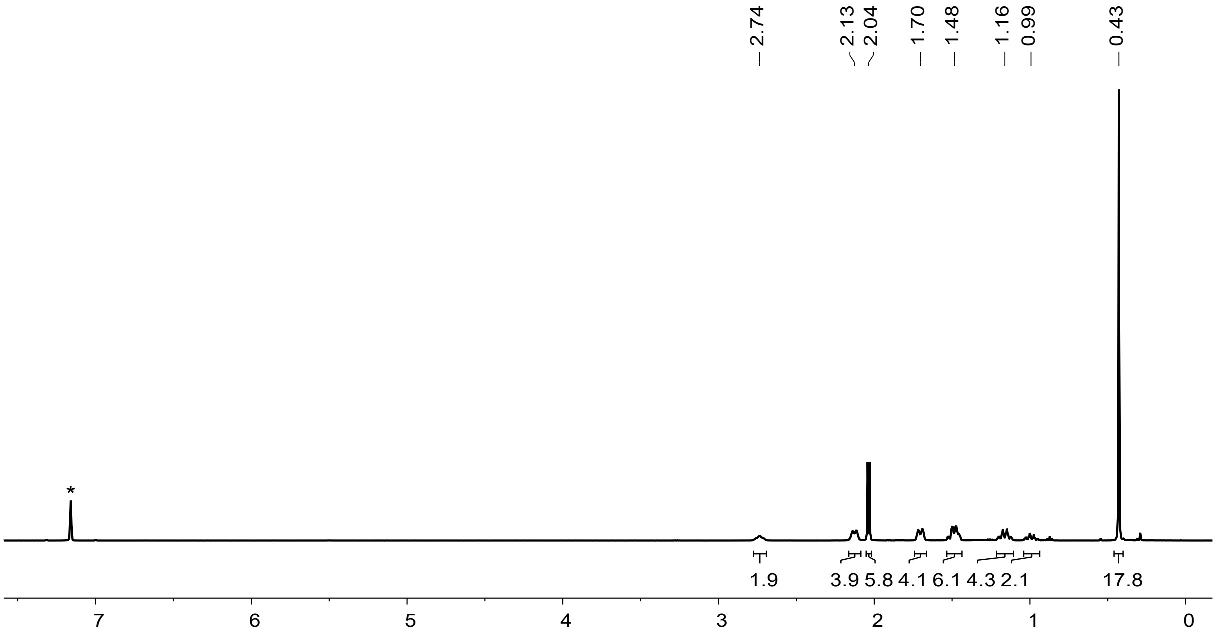

Figure S30. ^{1}H NMR spectrum (500.1 MHz, 305 K, benzene-d_6) of phosphole **21a** (*C_6D_5H).

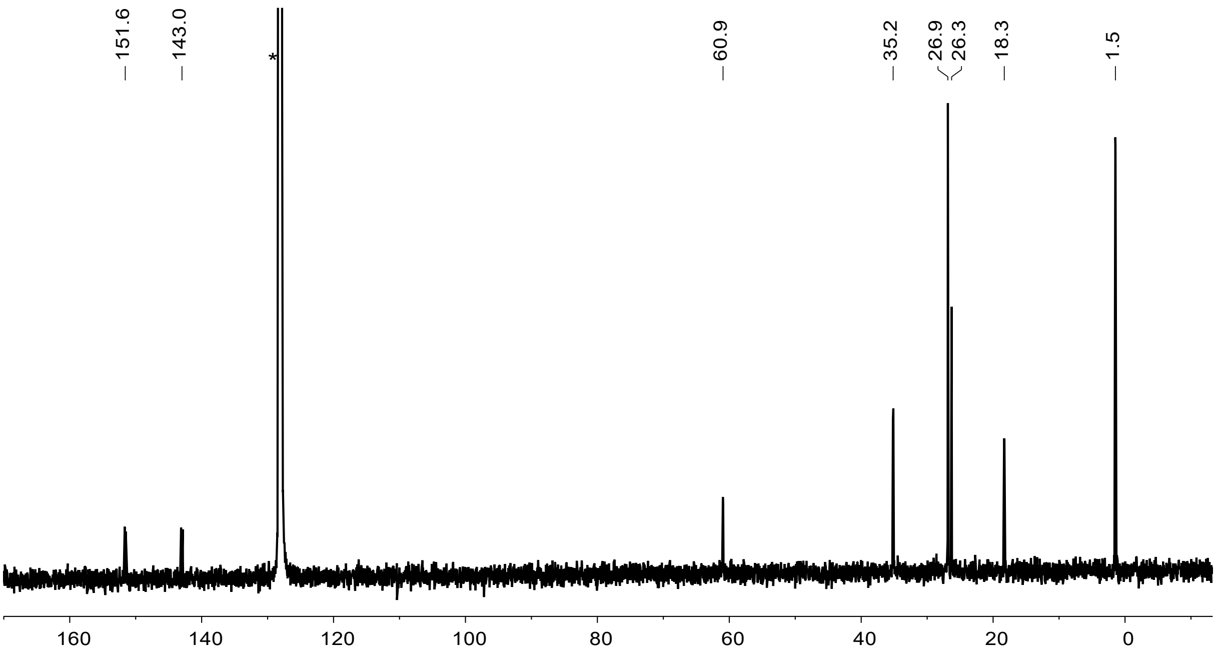

Figure S31. ^{13}C{^{1}H} NMR spectrum (125.7 MHz, 305 K, benzene-d_6) of phosphole **21a** (*C_6D_6).

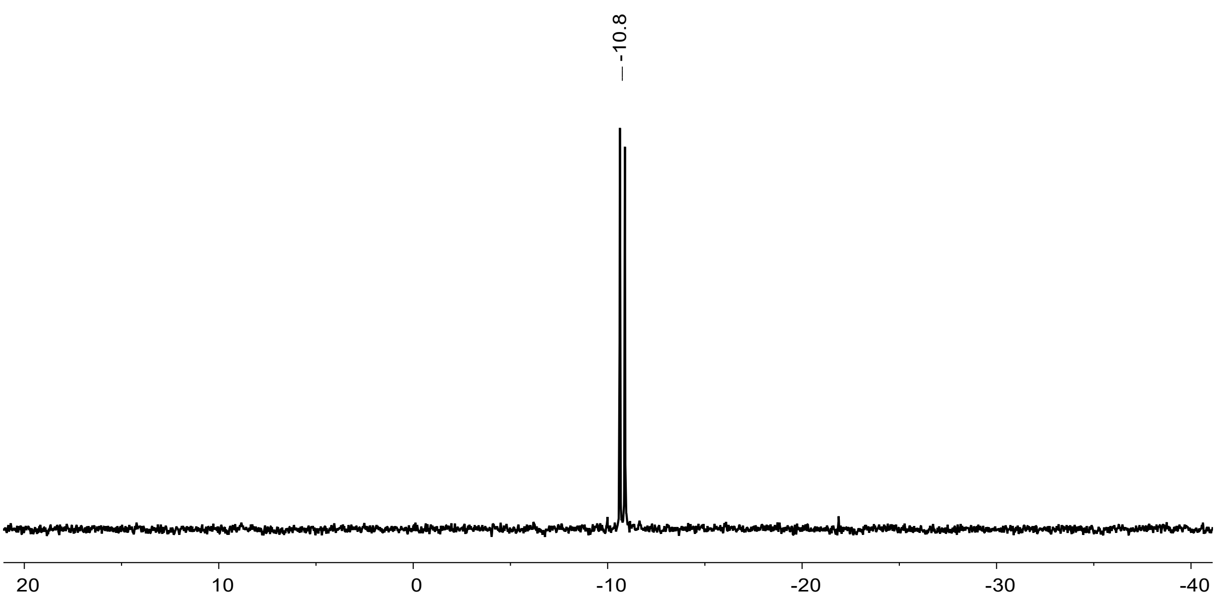

Figure S32. $^{29}Si\{^1H\}$ INEPT NMR spectrum (99.3 MHz, 305 K, benzene-d_6) of phosphole **21a**.

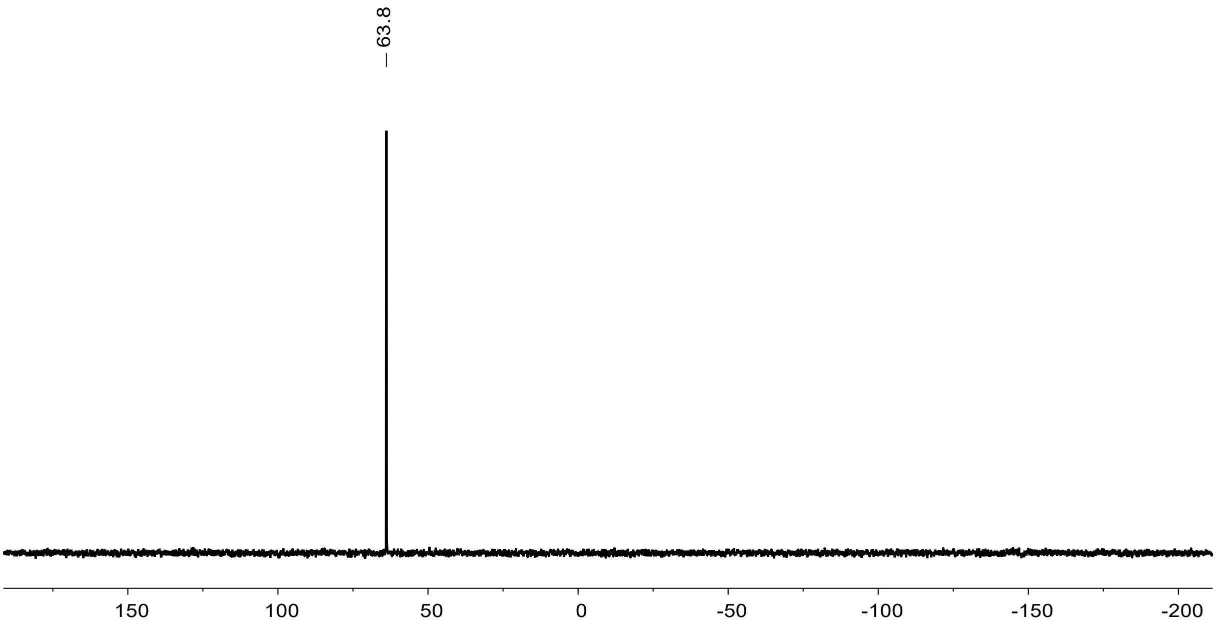

Figure S33. $^{31}P\{^1H\}$ NMR spectrum (202.4 MHz, 305 K, benzene-d_6) of phosphole **21a**.

Germylene 16b and Phosphole 21b

The product mixture contained phosphole **21b** and germylene **16b** in a mixture of 2 : 98, in an overall yield of 78%. Phosphole **21b** was analysed NMR spectroscopically from the mixture.

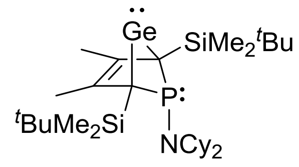

^{1}H NMR (500.1 MHz, 305 K, C_6D_6) δ = 0.21 (s, 6H, 2 x Si<u>Me</u>$_2$^{t}Bu), 0.34–0.36 (m, 6H, 2 x Si<u>Me</u>$_2$^{t}Bu), 1.11 (s, 18H, 2 x SiMe$_2$<u>^{t}Bu</u>), 2.29 (s, 6H, 2 x C<u>H</u>$_3$). The N<u>Cy</u>$_2$ signals are broad and overlay with other signals. **^{13}C{^{1}H} NMR** (125.7 MHz, 305 K, C_6D_6) δ = -2.5 (d, $^3J_{C,P}$ = 9 Hz, Si(<u>Me</u>$_2$)^{t}Bu), -2.2 (d, $^3J_{C,P}$ = 5 Hz, Si(<u>Me</u>$_2$)^{t}Bu), 17.0 (d, $^3J_{C,P}$ = 4 Hz, C$^{2/3}$–<u>Me</u>), 19.1 (d, $^4J_{C,P}$ = 3 Hz, Si(Me$_2$)<u>^{t}Bu</u>), 26.5 (N<u>Cy</u>$_2$), 27.5 (N<u>Cy</u>$_2$), 36.3 (br, N<u>Cy</u>$_2$), 28.5 (d, $^3J_{C,P}$ = 3 Hz, Si(Me$_2$)<u>^{t}Bu</u>), 59.0 (NCy$_2$, <u>C</u>^{ipso}), 73.0 (d, $^1J_{C,P}$ = 41 Hz, C$^{1/4}$), 131.1 (d, $^2J_{C,P}$ = 7 Hz, C$^{2/3}$).

^{29}**Si{^{1}H} INEPT NMR** (99.3 MHz, 305 K, C_6D_6) δ = -0.2 (d, $^2J_{Si,P}$ = 18 Hz).
^{31}**P{^{1}H} NMR** (202.4 MHz, 305 K, C_6D_6) δ = 57.7.
MS (EI): m/z (%) = 73 (77), 180 (38), 225 (34), 283 (100), 348 (32), 404 (26), 462 (48), 519 (32), 593 (6) [M$^+$].

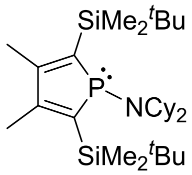

^{1}**H NMR** (500.1 MHz, 305 K, C_6D_6): δ = 0.38 (Si<u>Me</u>$_2$^{t}Bu), 2.14 (d, $^4J_{H,P}$ = 6.2 Hz, C$^{2/3}$-<u>Me</u>). Due to low concentration, assignment of the NCy_2 signals was not possible.
^{13}**C{^{1}H} NMR** (125.7 MHz, 305 K, C_6D_6): δ = 141 (C$^{1/4}$), 154 (C$^{2/3}$). Data retrieved from the ^{1}H^{13}C HMBC NMR spectrum. Assignment of the remaining signals was not possible due to low concentration of the phosphole.
^{29}**Si{^{1}H} INEPT NMR** (99.3 MHz, 305 K, C_6D_6): δ = -3.1 (d, $^2J_{Si,P}$ = 23 Hz).
^{31}**P{^{1}H} NMR** (202.4 MHz, 305 K, C_6D_6): δ = 69.7.

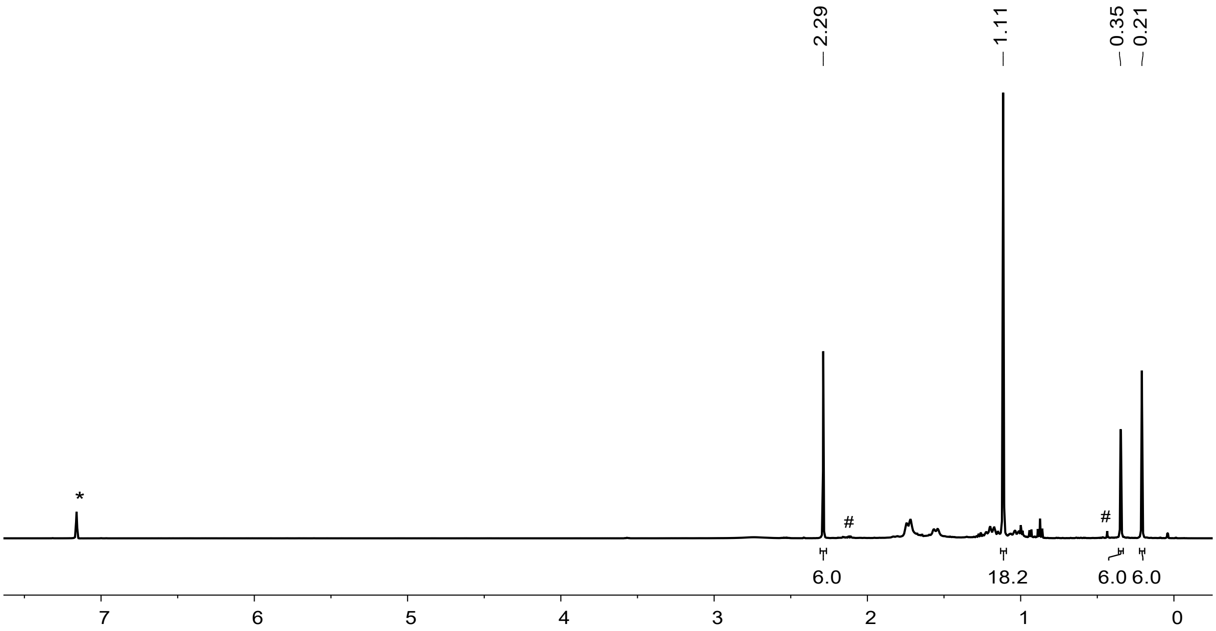

Figure S34. ^{1}H NMR spectrum (500.1 MHz, 305 K, benzene-d$_6$) of germylene **16b** (*C_6D_5H, #phosphole **21b**).

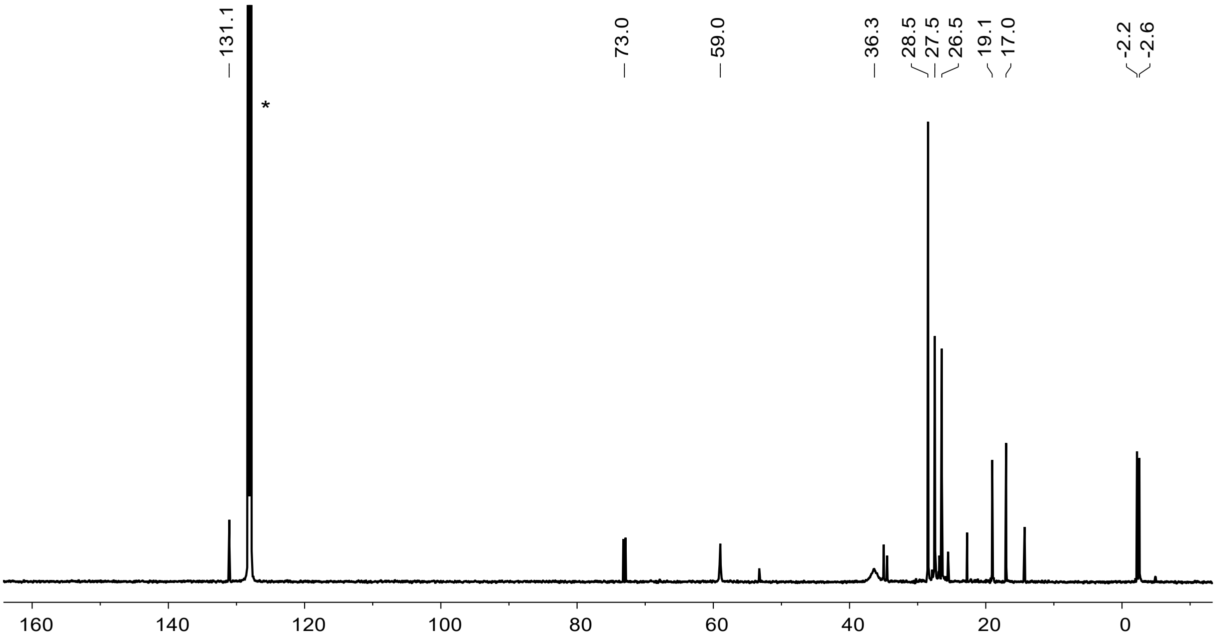

Figure S35. $^{13}C\{^{1}H\}$ NMR spectrum (125.7 MHz, 305 K, benzene-d$_6$) of germylene **16b** (*C_6D_6).

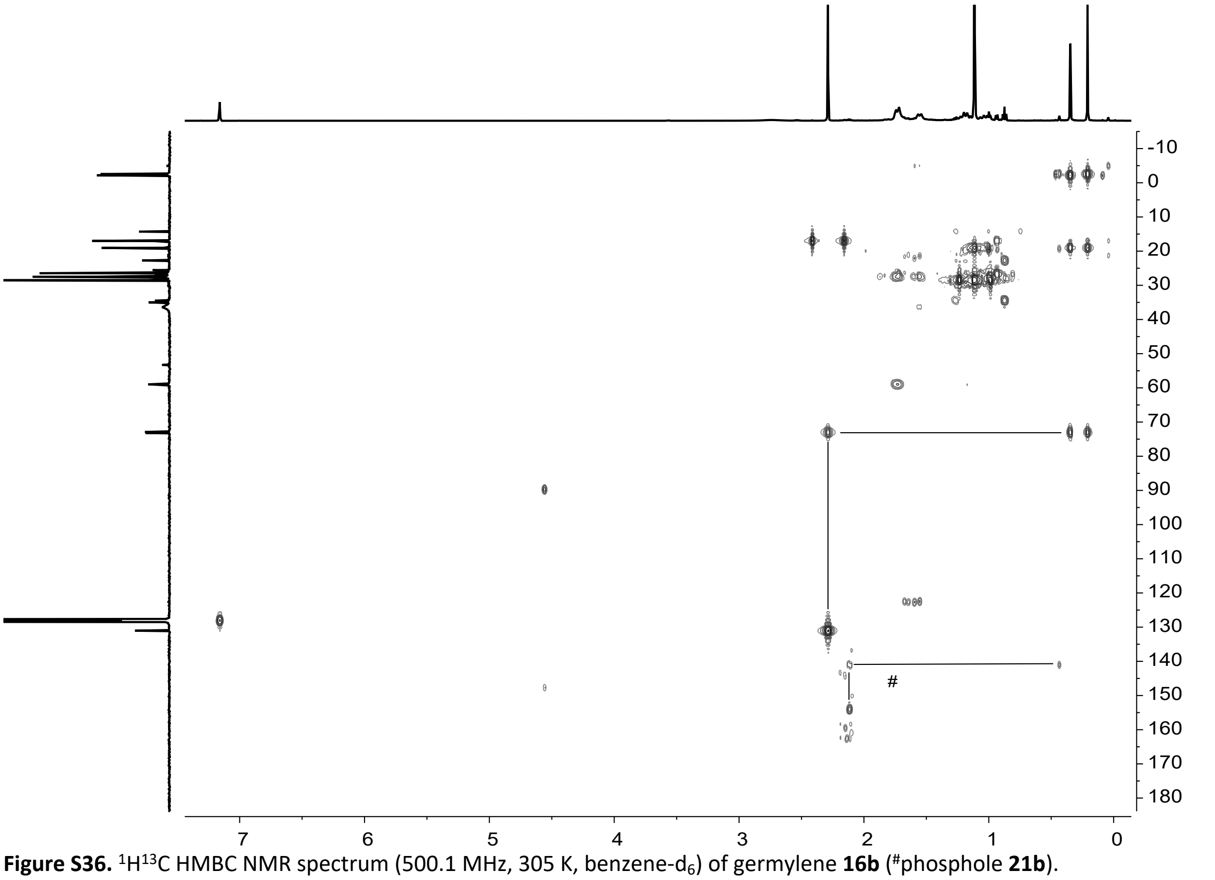

Figure S36. $^{1}H^{13}C$ HMBC NMR spectrum (500.1 MHz, 305 K, benzene-d$_6$) of germylene **16b** ($^{\#}$phosphole **21b**).

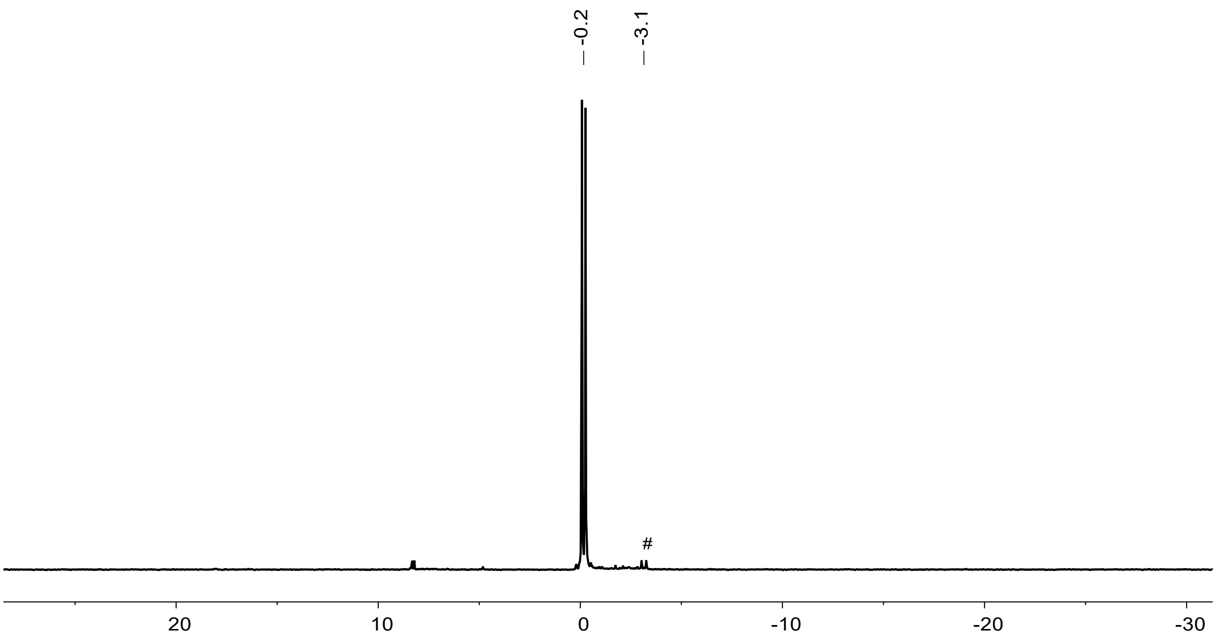

Figure S37. $^{29}Si\{^1H\}$ INEPT NMR spectrum (99.3 MHz, 305 K, benzene-d$_6$) of germylene **16b** ($^{\#}$phosphole **21b**).

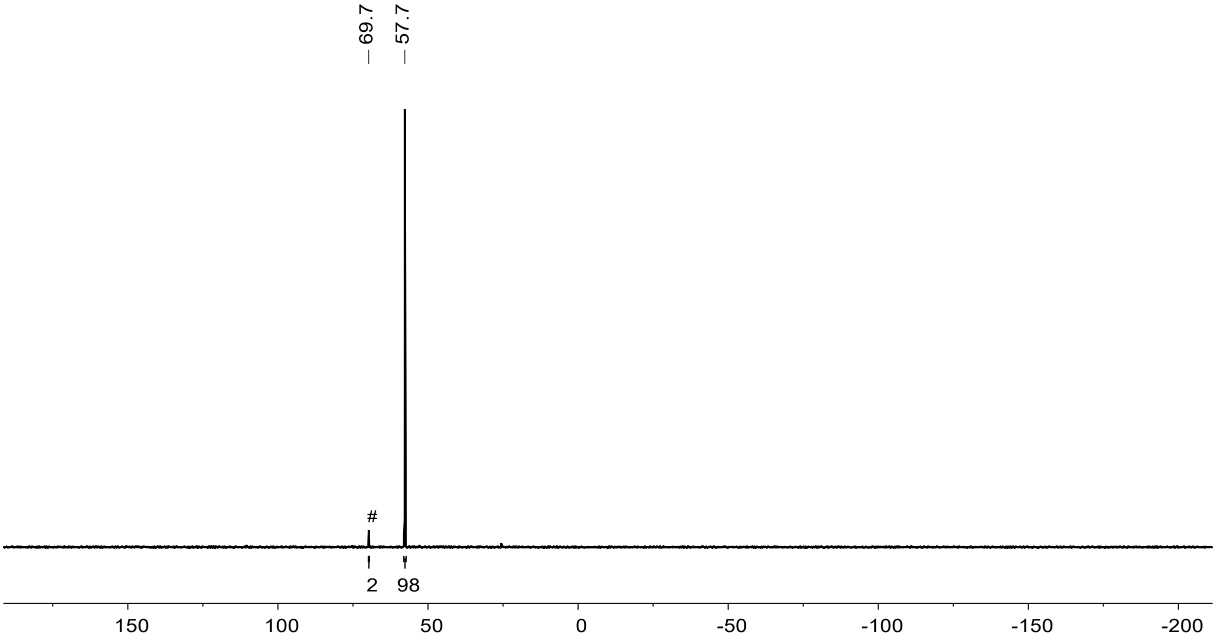

Figure S38. $^{31}P\{^1H\}$ NMR spectrum (202.4 MHz, 305 K, benzene-d$_6$) of germylene **16b** ($^{\#}$phosphole **21b**).

Phosphole 22a

Dipotassium germolediide **K$_2$[8a]** was synthesised by the reduction of dichlorogermole **8a** (0.20 mmol) with potassium in THF (6 mL). Afterwards, the solvent was removed and the germolediide salt was dissolved in diethyl ether (4 mL). It was cooled to -50°C and dichloro(piperidino)phosphane **12** (37 mg, 0.20 mmol), dissolved in THF (3 mL), was added. The reaction mixture was stirred for 30 minutes before the cold bath was removed and the solution stirred at room temperature overnight. The solvent was removed under reduced pressure. The crude product was redissolved in pentane and filtered over a PTFE syringe filter. Removal of the solvent gave phosphole **22a** (66 mg, 0.19 mmol, 95%) as orange oil. Low temperature NMR measurements (-40°C) were performed to detect the signals of the piperidino substituent.

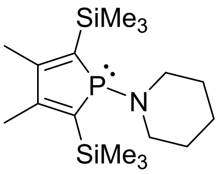

^{1}H NMR (499.9 MHz, 305 K, C_6D_6): δ = 0.39 (s, 18H, 2 x Si$\underline{Me_3}$), 1.28-1.33 (m, 4H, $NC_5\underline{H_{10}}$), 1.95 (d, $^{4}J_{H,H}$ = 5.4 Hz, 6H, 2 x $C^{2/3}$–$\underline{Me}$).

^{13}C{^{1}H} NMR (125.7 MHz, 305 K, C_6D_6): δ = 0.9 (d, $^{3}J_{C,P}$ = 3 Hz, Si$\underline{Me_3}$), 18.0 (d, $^{3}J_{C,P}$ = 6 Hz, $C^{2/3}$–$\underline{Me}$), 27.4 ($NC_5\underline{H_{10}}$), 142.9 (d, $^{1}J_{C,P}$ = 32 Hz, $C^{1/4}$), 154.3 (d, $^{2}J_{C,P}$ = 16 Hz, $C^{2/3}$).

^{29}Si{^{1}H} INEPT NMR (99.3 MHz, 305 K, C_6D_6): δ = -11.0 (d, $^{2}J_{Si,P}$ = 25 Hz).

^{31}P{^{1}H} NMR (202.4 MHz, 305 K, C_6D_6): δ = 92.2.

MS (EI): m/z (%) = 73 (100), 84 (35), 167 (50), 265 (60), 339 (100) [M$^+$].

Spectra recorded at -40°C:

^{1}H NMR (499.9 MHz, 233 K, toluene-d_8): δ = 0.38 (s, 18H, Si$\underline{Me_3}$), 1.41-1.47 (m, 4H, $NC_5\underline{H_{10}}$), 1.87 (d, $^{4}J_{H,P}$ = 5.4 Hz, 6H, $C^{2/3}$-$\underline{Me}$), 2.22-2.28 (m, 3H, $NC_5\underline{H_{10}}$), 3.06-3.14 (m, 3H, $NC_5\underline{H_{10}}$).

^{13}C{^{1}H} NMR (125.7 MHz, 233 K, toluene-d_8): δ = 0.6 (d, $^{3}J_{C,P}$ = 3 Hz, $SiMe_3$), 17.8 (d, $^{3}J_{C,P}$ = 6 Hz, $C^{2/3}$-$\underline{Me}$), 27.4 (NC_5H_{10}, *meta*-$\underline{C}H_2$, *para*-$\underline{C}H_2$), 49.2 (d, $^{2}J_{C,P}$ = 10 Hz, NC_5H_{10}, *ortho*-$\underline{C}H_2$), 53.1 (d, $^{2}J_{C,P}$ = 33 Hz, NC_5H_{10}, *ortho*-$\underline{C}H_2$), 142.3 (d, $^{1}J_{C,P}$ = 30 Hz, $C^{1/4}$), 154.1 (d, $^{2}J_{C,P}$ = 17 Hz, $C^{2/3}$).

^{31}P{^{1}H} NMR (202.4 MHz, 233 K, toluene-d_8): δ = 91.5.

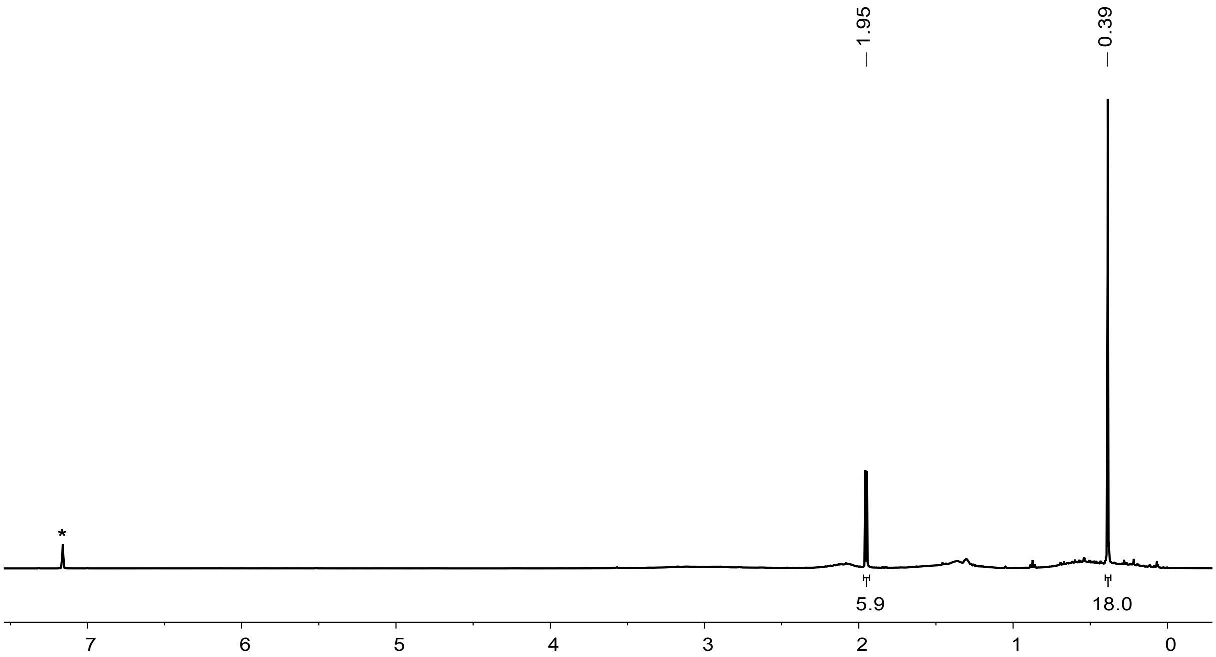

Figure S39. ^{1}H NMR spectrum (500.1 MHz, 305 K, benzene-d_6) of phosphole **22a** (*C_6D_5H).

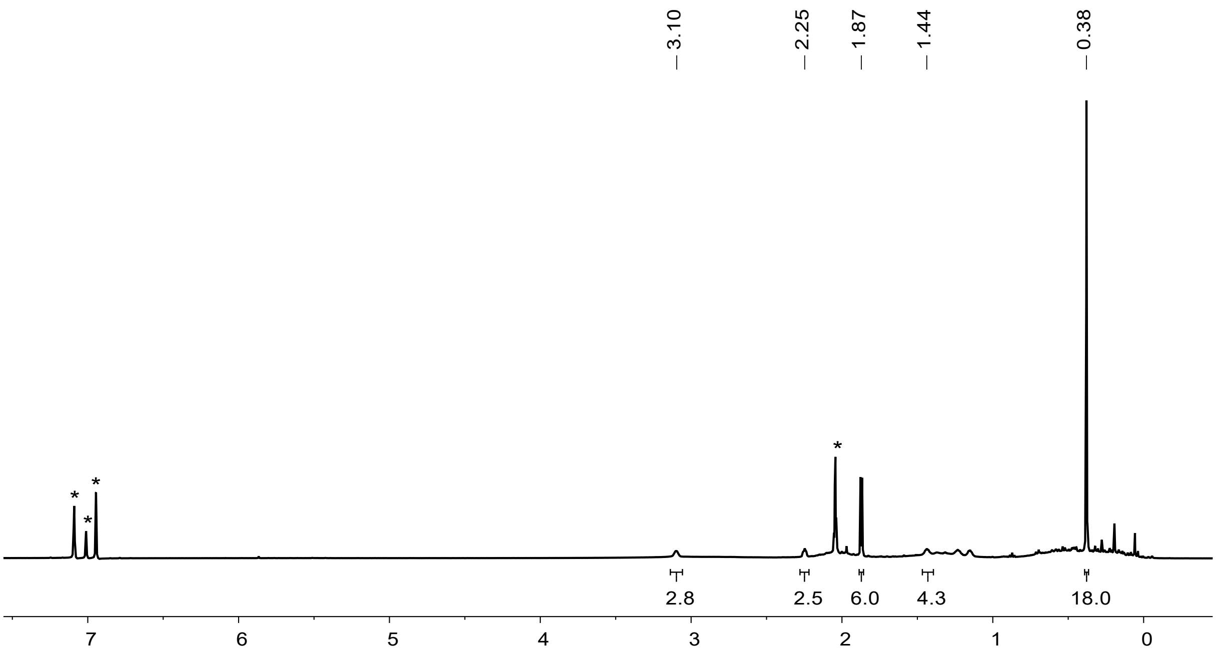

Figure S40. ^{1}H NMR spectrum (500.1 MHz, 233 K, toluene-d_8) of phosphole **22a** (*toluene-d_8).

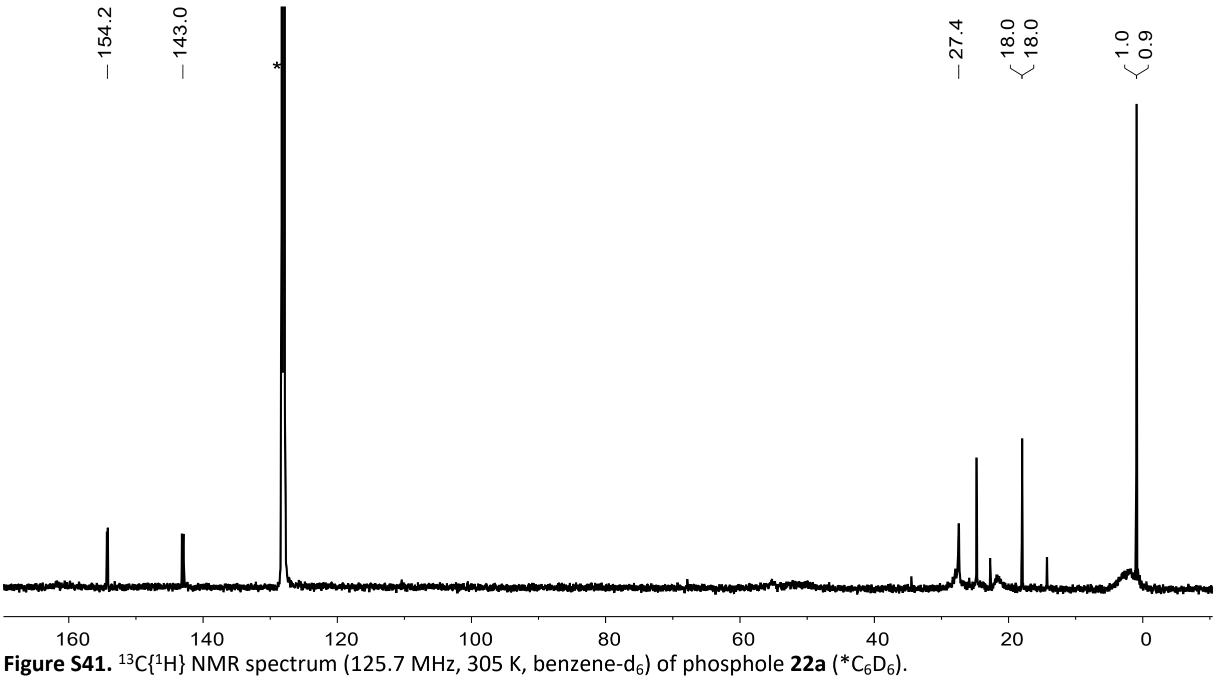

Figure S41. $^{13}C\{^{1}H\}$ NMR spectrum (125.7 MHz, 305 K, benzene-d_6) of phosphole **22a** (*C_6D_6).

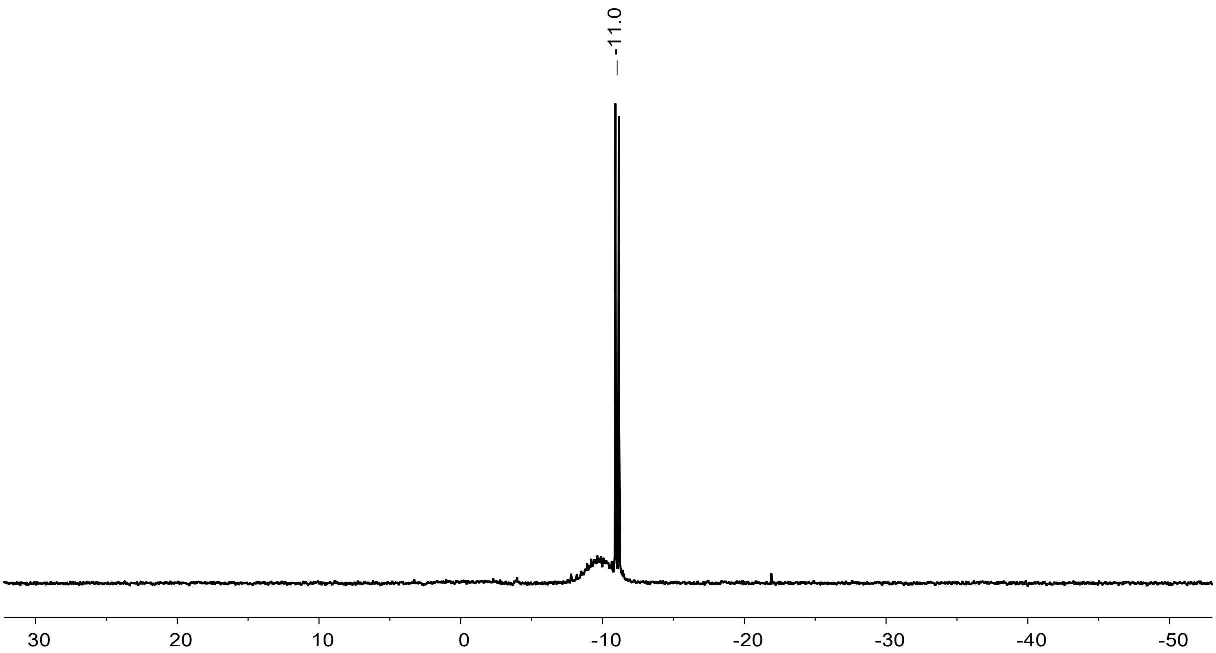

Figure S42. $^{29}Si\{^1H\}$ INEPT NMR spectrum (99.3 MHz, 305 K, benzene-d_6) of phosphole **22a**.

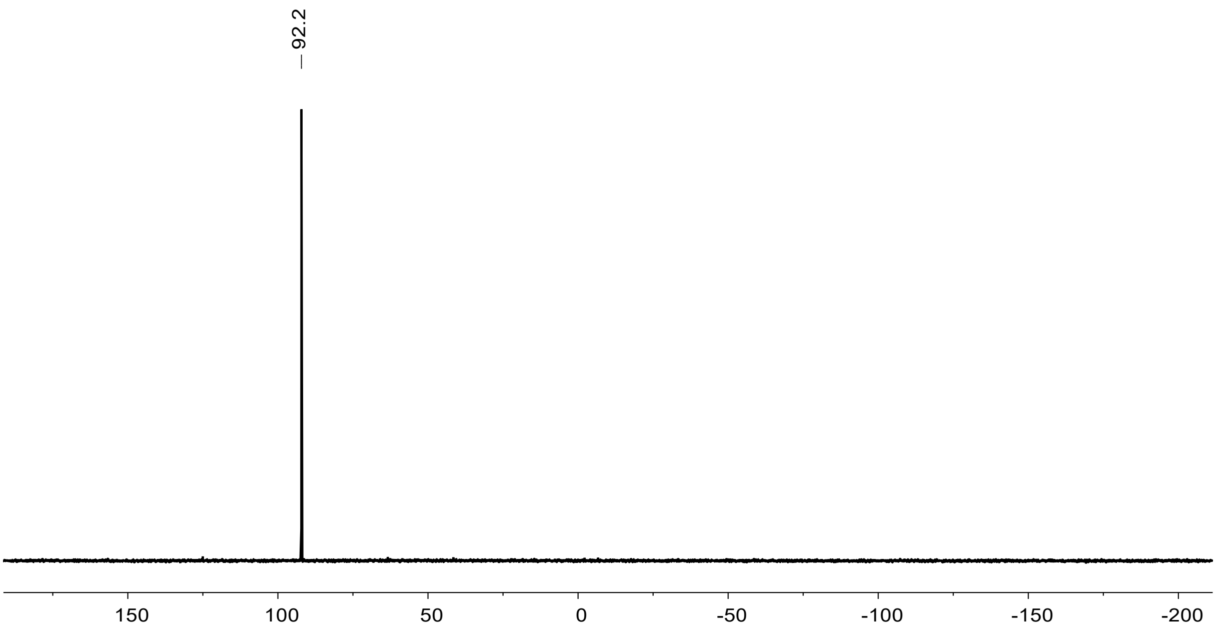

Figure S43. $^{31}P\{^1H\}$ NMR spectrum (202.4 MHz, 305 K, benzene-d_6) of phosphole **22a**.

Germylene 17b and Phosphole 22b

The mixture contained phosphole **22b** and germylene **17b** in a ratio of 7 : 93, in an overall yield of 71%. Phosphole **22b** was analysed NMR spectroscopically from the mixture.

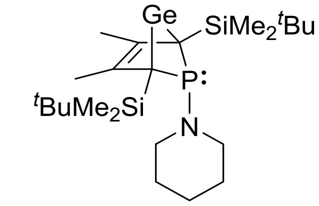

^{1}H NMR (500.1 MHz, 305 K, C_6D_6) δ = 0.23 (s, 6H, 2 x Si<u>Me</u>$_2$^{t}Bu), 0.24-0.25 (m, 6H, 2 x Si<u>Me</u>$_2$^{t}Bu), 1.12 (s, 18H, 2 x SiMe$_2$<u>^{t}Bu</u>), 1.29–1.34 (m, 6H, NC_5H_{10}), 2.22 (s, 6H, 2 x C<u>H</u>$_3$), 2.69–2.74 (m, 4H, NC_5H_{10}).
$^{13}C\{^1H\}$ NMR (125.7 MHz, 305 K, C_6D_6) δ = -3.0 (d, $^3J_{C,P}$ = 9 Hz, Si(<u>Me</u>$_2$)^{t}Bu), -2.1 (d, $^3J_{C,P}$ = 7 Hz, Si(<u>Me</u>$_2$)^{t}Bu), 16.2 (d, $^3J_{C,P}$ = 4 Hz, $C^{2/3}$–<u>Me</u>), 18.6–18.7 (m, Si(Me$_2$)<u>^{t}Bu</u>), 25.5 (NC_5H_{10}), 28.0 (d, $^3J_{C,P}$ = 3 Hz, Si(Me$_2$)<u>^{t}Bu</u>), 49.3 (d, $^2J_{C,P}$ = 11 Hz, NC_5H_{10}), 73.4 (d, $^1J_{C,P}$ = 37 Hz, $C^{1/4}$), 130.4 (d, $^2J_{C,P}$ = 8 Hz, $C^{2/3}$).
$^{29}Si\{^1H\}$ INEPT NMR (99.3 MHz, 305 K, C_6D_6) δ = 0.4 (d, $^2J_{Si,P}$ = 17 Hz).
$^{31}P\{^1H\}$ NMR (202.4 MHz, 305 K, C_6D_6) δ = 59.8.

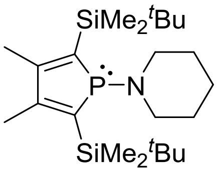

^{1}H NMR (499.9 MHz, 305 K, C_6D_6): δ = 0.39 (s, 12H, 2 x Si<u>Me</u>$_2$^{t}Bu), 1.04 (s, 18H, SiMe$_2$<u>^{t}Bu</u>), 2.06 (d, $^4J_{H,P}$ = 5.3 Hz, $C^{2/3}$-Me). Signals of the piperidino substituent could not be assigned due to low concentration.
$^{13}C\{^1H\}$ NMR (125.7 MHz, 305 K, C_6D_6): δ = 140 ($C^{1/4}$), 155 ($C^{2/3}$). Data retrieved from the $^1H^{13}C$ HMBC NMR spectrum. Assignment of the remaining signals was not possible due to low concentration of the phosphole.
$^{29}Si\{^1H\}$ INEPT NMR (99.3 MHz, 305 K, C_6D_6): δ = -2.9 (d, $^2J_{Si,P}$ =22 Hz).
$^{31}P\{^1H\}$ NMR (202.4 MHz, 305 K, C_6D_6): δ = 95.9.
MS (EI): m/z (%) = 73 (100), 169 (26), 252 (33), 283 (81), 310 (25), 367 (34), 413 (70), 423 (32) [M^+].

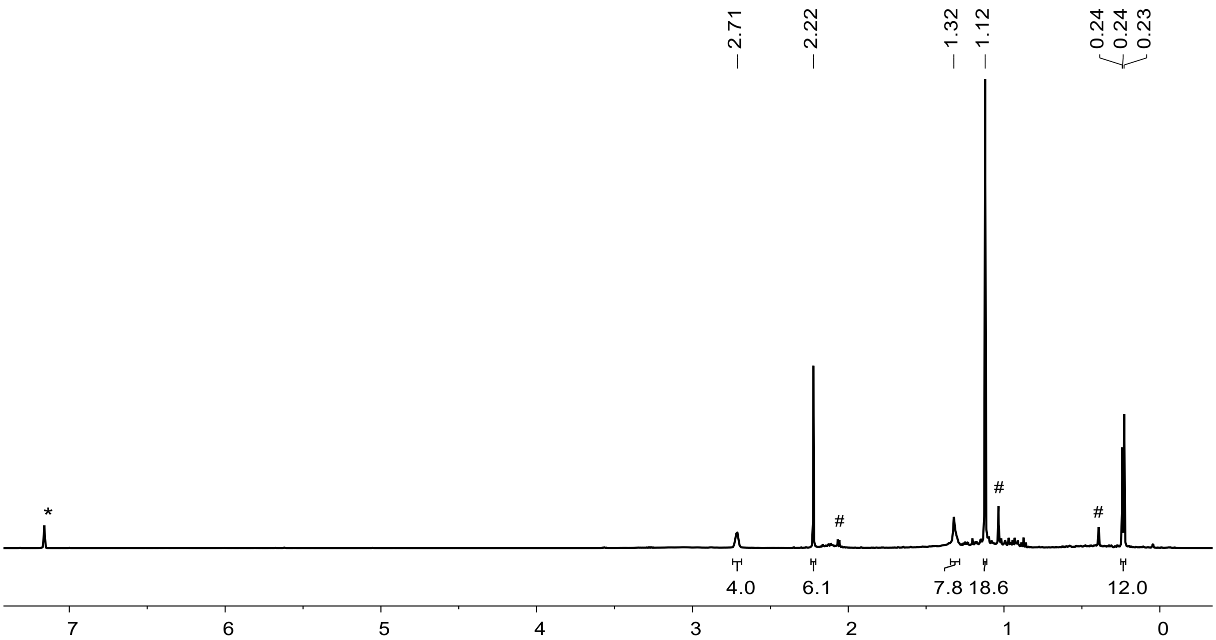

Figure S44. ^1H NMR spectrum (500.1 MHz, 305 K, benzene-d_6) of germylene **17b** (*C_6D_5H, #phosphole **22b**).

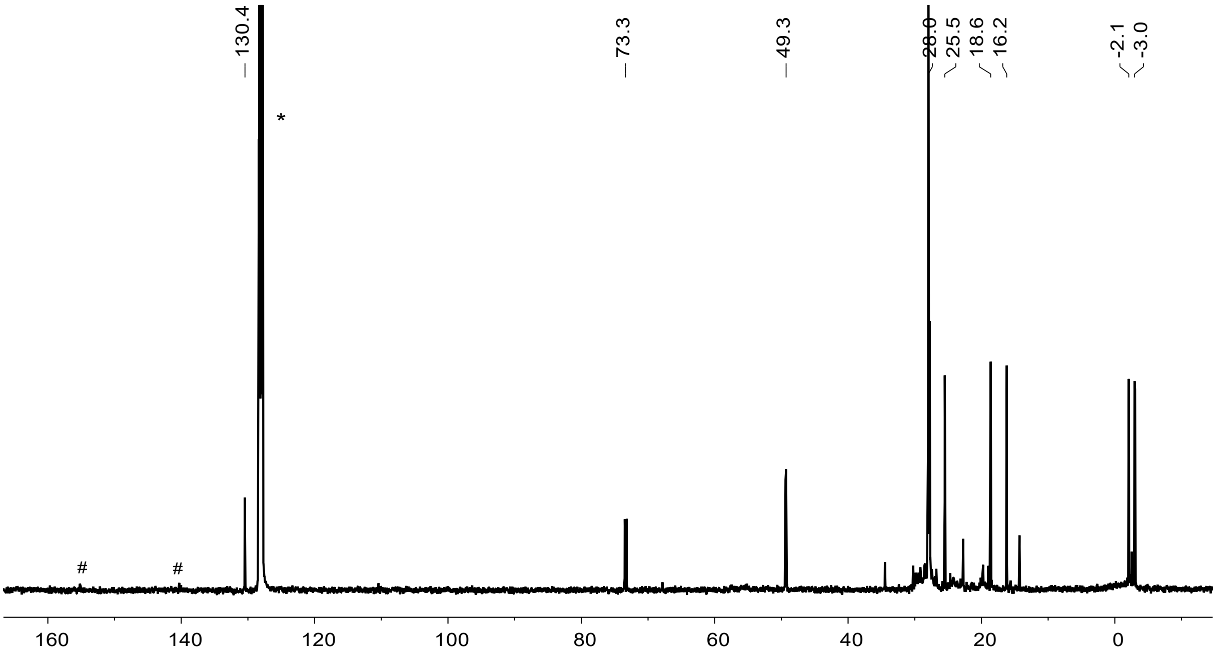

Figure S45. $^{13}C\{^1H\}$ NMR spectrum (125.7 MHz, 305 K, benzene-d$_6$) of germylene **17b** (*C_6D_6, #phosphole **22b**).

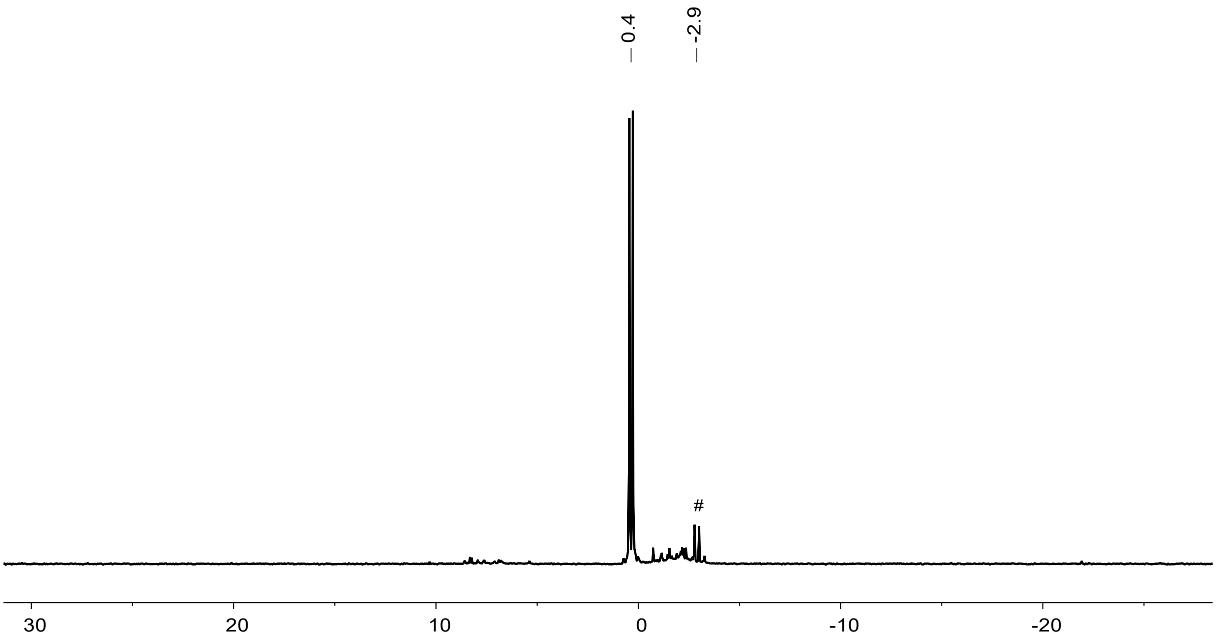

Figure S46. $^{29}Si\{^1H\}$ INEPT NMR spectrum (99.3 MHz, 305 K, benzene-d$_6$) of germylene **17b** (# phosphole **22b**).

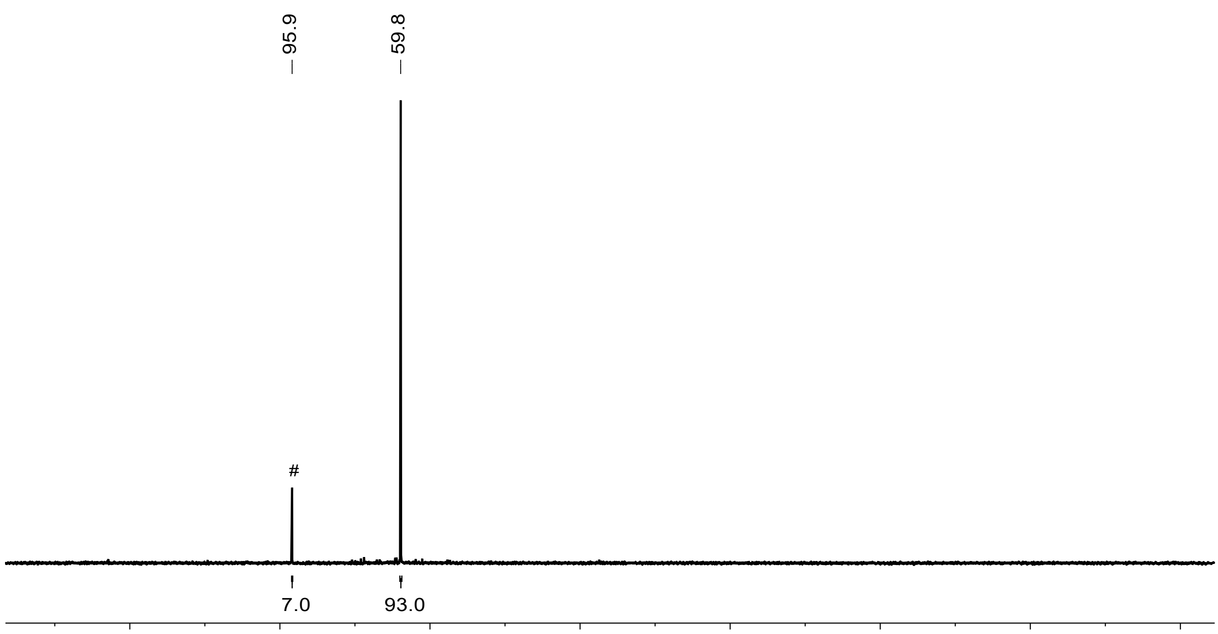

Figure S47. $^{31}P\{^{1}H\}$ NMR spectrum (202.4 MHz, 305 K, benzene-d_6) of germylene **17b** ($^{\#}$phosphole **22b**).

Germylene 18a and Phosphole 23a

The product mixture contained phosphole **23a** and germylene **18a** in a mixture of 58 : 42.

To isolate the phosphole **23a**, the solution that had already changed colour from orange-brown to black, was diluted in pentane and filtered over silica gel. Removal of the solvent gave phosphole **23a** (15 mg, 0.04 mmol, 20%) as light orange solid. Keeping a pentane solution of the compound at -30°C overnight afforded colourless crystals suitable for XRD analysis.

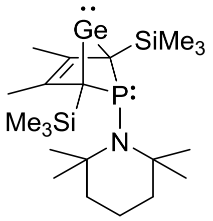

^{1}H NMR (500.1 MHz, 305 K, C_6D_6): δ = 0.32 (s, 18H, 2 x SiMe$_3$), 1.04 (br s, 6H, 2 x TMP-Me), 1.34-1.39 (m, 6H, TMP), 1.73 (br s, 6H, 2 x TMP-Me), 2.19 (s, 6H, Me).

$^{13}C\{^{1}H\}$ NMR (125.7 MHz, 305 K, C_6D_6): δ = 2.2 (d, $^{3}J_{C,P}$ = 6 Hz, 2 x SiMe$_3$), 16.4 (d, $^{3}J_{C,P}$ = 5 Hz, $C^{2/3}$-Me), 18.1 (TMP, γ-C), 33.4 (2 x TMP-Me), 35.4 (d, $^{3}J_{C,P}$ = 27 Hz, 2 x TMP-Me), 43.0 (TMP, β-C), 43.7 (TMP, β-C), 55.5 (d, $^{2}J_{C,P}$ = 12 Hz , TMP, α-C), 57.3 (d, $^{2}J_{C,P}$ = 25 Hz, TMP, α-C), 81.9 (d, $^{1}J_{C,P}$= 48 Hz, $C^{1/4}$), 134.1 (d, $^{2}J_{C,P}$= 8 Hz, $C^{2/3}$).

$^{29}Si\{^{1}H\}$ INEPT NMR (99.3 MHz, 305 K, C_6D_6): δ = -7.4 (d, $^{2}J_{Si,P}$ = 23 Hz).

$^{31}P\{^{1}H\}$ NMR (202.4 MHz, 305 K, C_6D_6): δ = 30.5.

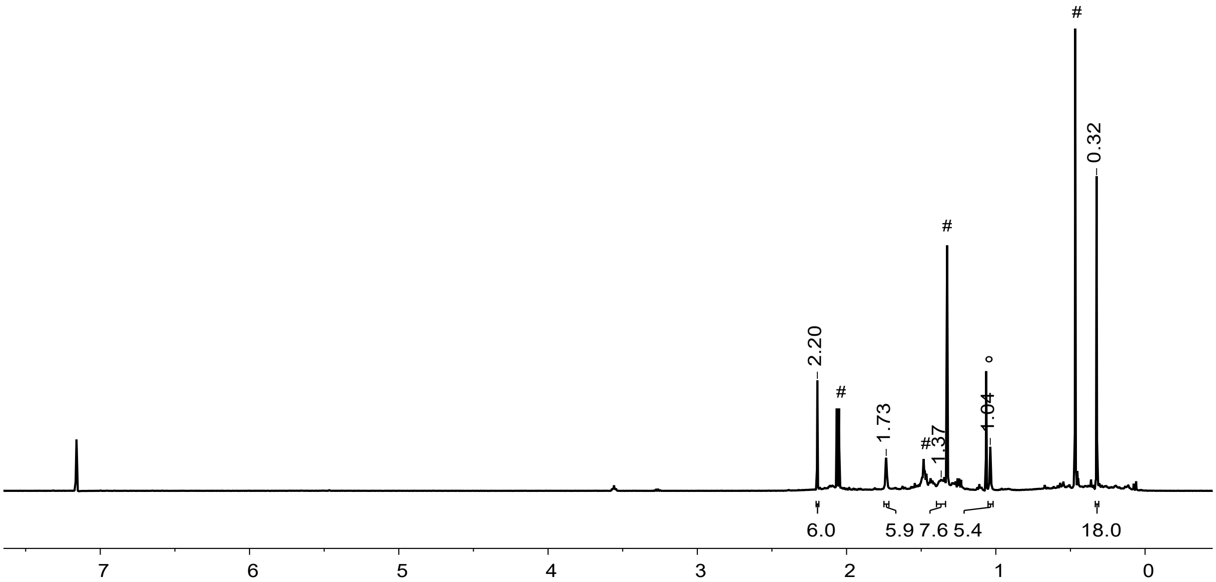

Figure S48. ^{1}H NMR spectrum (500.1 MHz, 305 K, benzene-d_6) of germylene **18a** (#phosphole **23a**, °impurity).

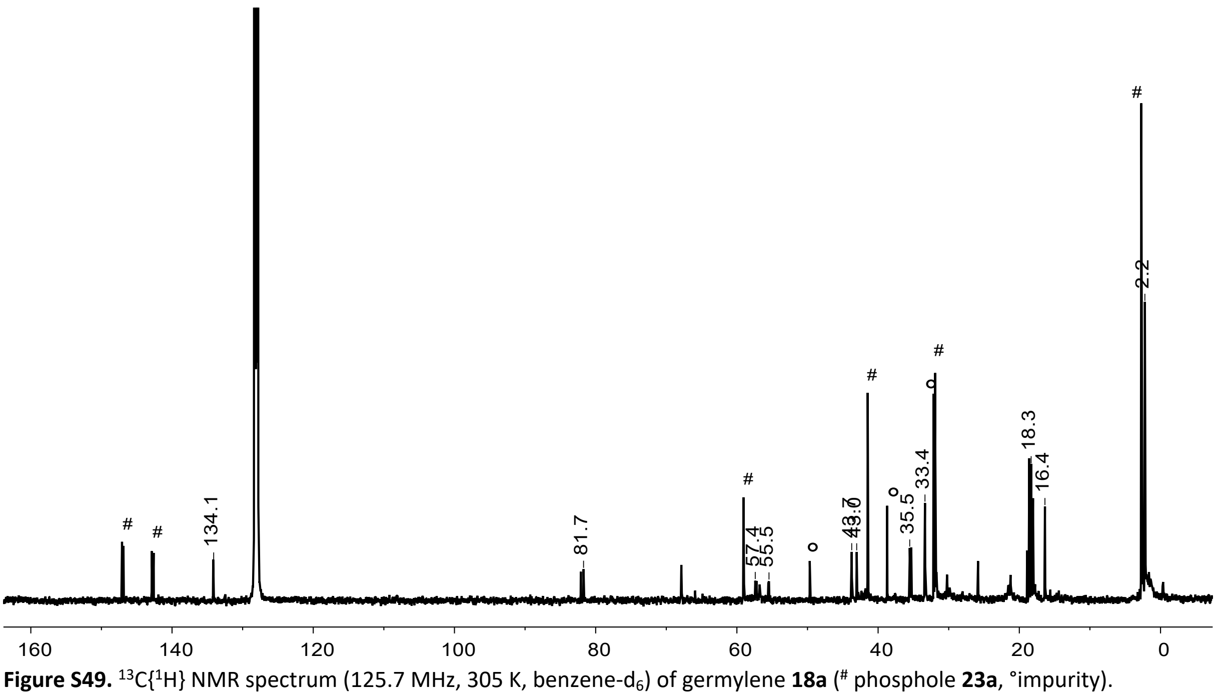

Figure S49. $^{13}C\{^{1}H\}$ NMR spectrum (125.7 MHz, 305 K, benzene-d_6) of germylene **18a** (# phosphole **23a**, °impurity).

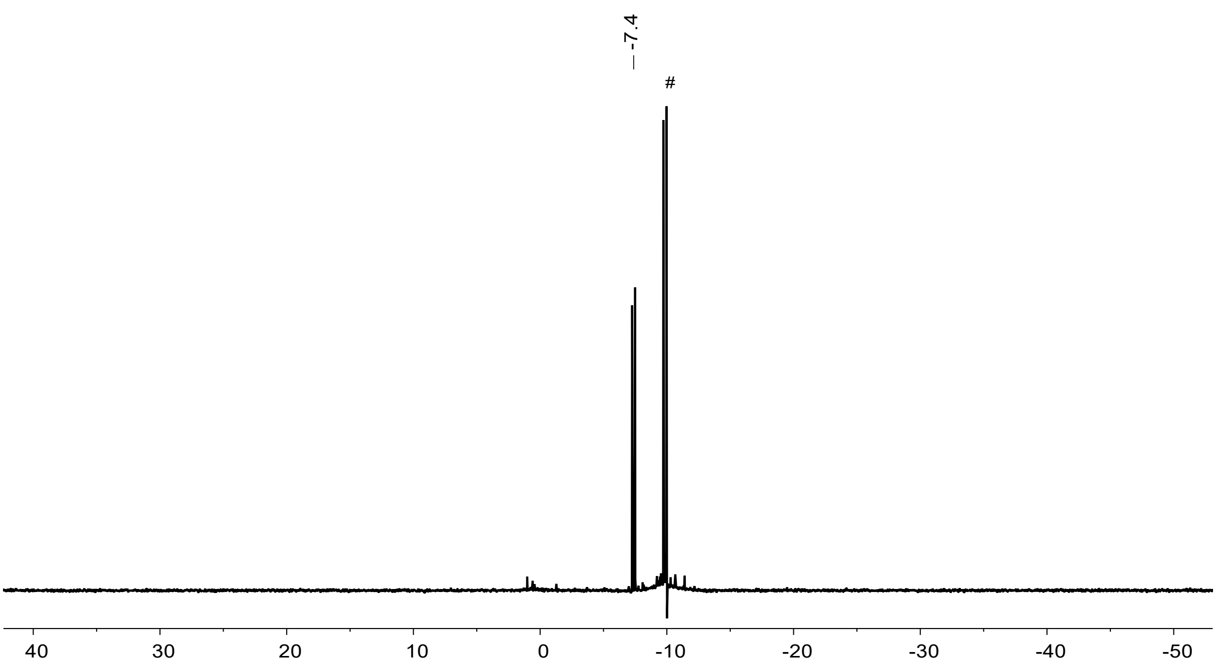

Figure S50. $^{29}Si\{^1H\}$ INEPT NMR spectrum (99.3 MHz, 305 K, benzene-d$_6$) of germylene **18a** (#phosphole **23a**).

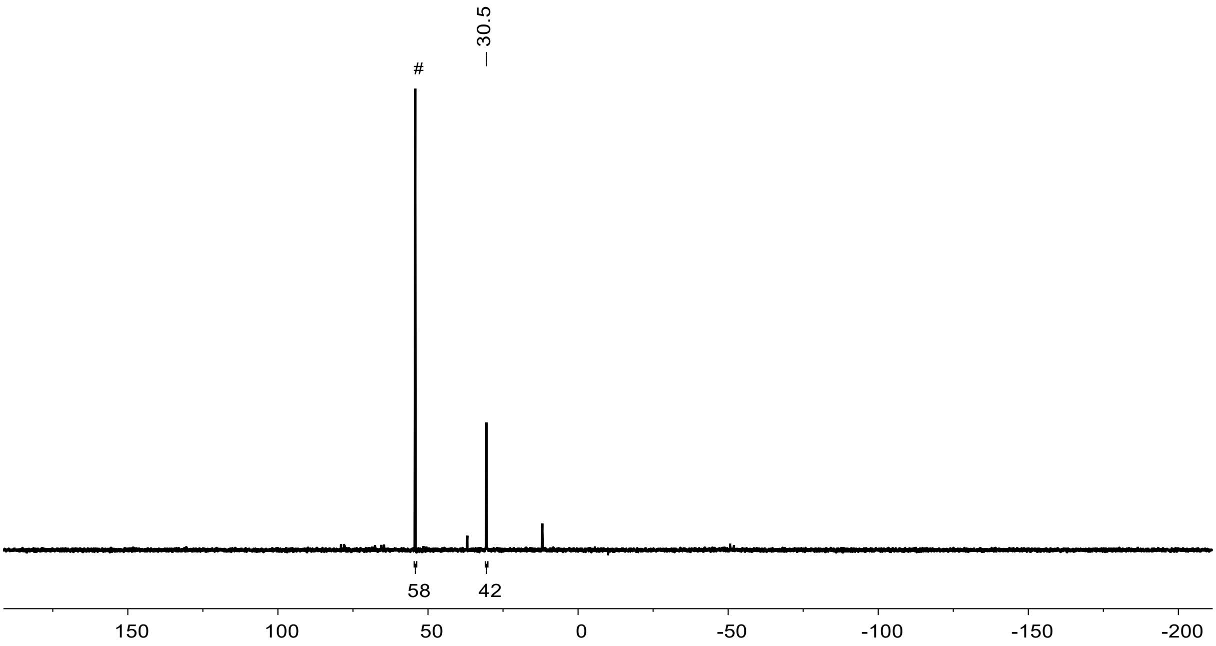

Figure S51. $^{31}P\{^1H\}$ NMR spectrum (202.4 MHz, 305 K, benzene-d$_6$) of germylene **18a** (#phosphole **23a**).

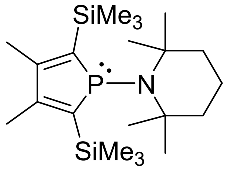

^{1}H NMR (499.9 MHz, 305 K, C_6D_6): δ = 0.47 (s, 18H, 2 x Si<u>Me</u>$_3$), 1.33 (s, 12H, TMP, 4 x <u>Me</u>), 1.45-1.52 (m, 6H, TMP, 3 x C<u>H</u>$_2$), 2.06 (d, $^4J_{H,P}$ = 7.6 Hz, 6H, 2 x C$^{2/3}$–<u>Me</u>).

$^{13}C\{^1H\}$ NMR (125.7 MHz, 305 K, C_6D_6): δ = 2.7 (d, $^3J_{C,P}$ = 2 Hz, 2 x Si<u>Me</u>$_3$), 18.3 (TMP, *para*-<u>C</u>), 18.6 (d, $^3J_{C,P}$ = 7 Hz, C$^{2/3}$–<u>Me</u>), 31.9 (d, $^3J_{C,P}$ = 8 Hz, TMP, 4 x <u>Me</u>), 41.5 (d, $^4J_{C,P}$ = 2 Hz, TMP, 2 x *meta*-<u>C</u>), 59.0 (d, $^2J_{C,P}$ = 6 Hz, TMP, 2 x *ortho*-<u>C</u>), 142.7 (d, $^1J_{C,P}$ = 34 Hz, C$^{1/4}$), 147.0 (d, $^2J_{C,P}$ = 26 Hz, C$^{2/3}$).

^{29}Si{^{1}H} INEPT NMR (99.3 MHz, 305 K, C_6D_6): δ = -9.8 (d, $^2J_{Si,P}$ = 25 Hz).
^{31}P{^{1}H} NMR (202.4 MHz, 305 K, C_6D_6): δ = 54.2.
MS (EI): m/z (%) = 73 (100), 114 (25), 312 (38), 322 (46), 380 (100), 395 (73) [M$^+$].

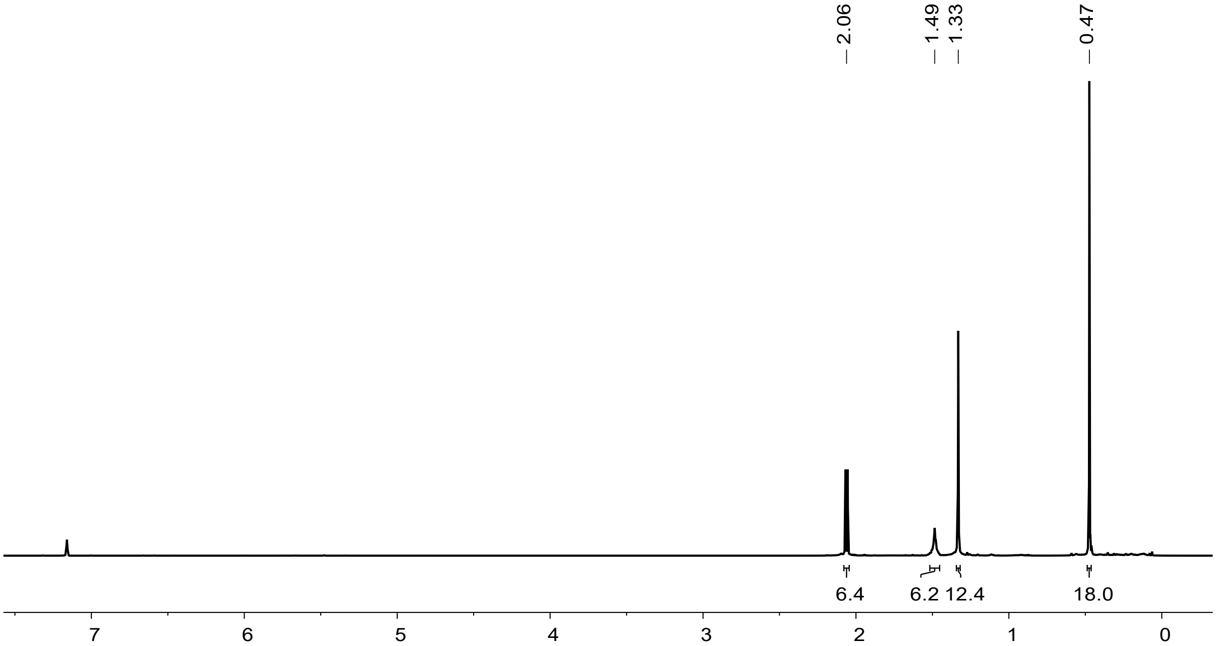

Figure S52. ^{1}H NMR spectrum (500.1 MHz, 305 K, benzene-d$_6$) of phosphole **23a**.

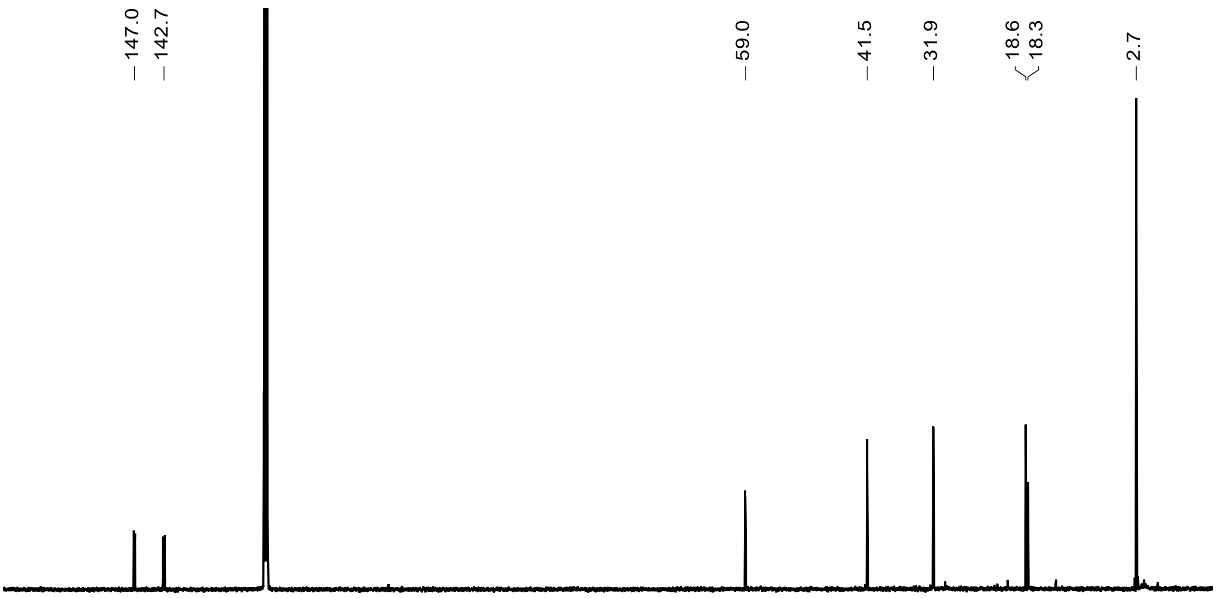

Figure S53. ^{13}C{^{1}H} NMR spectrum (125.7 MHz, 305 K, benzene-d$_6$) of phosphole **23a**.

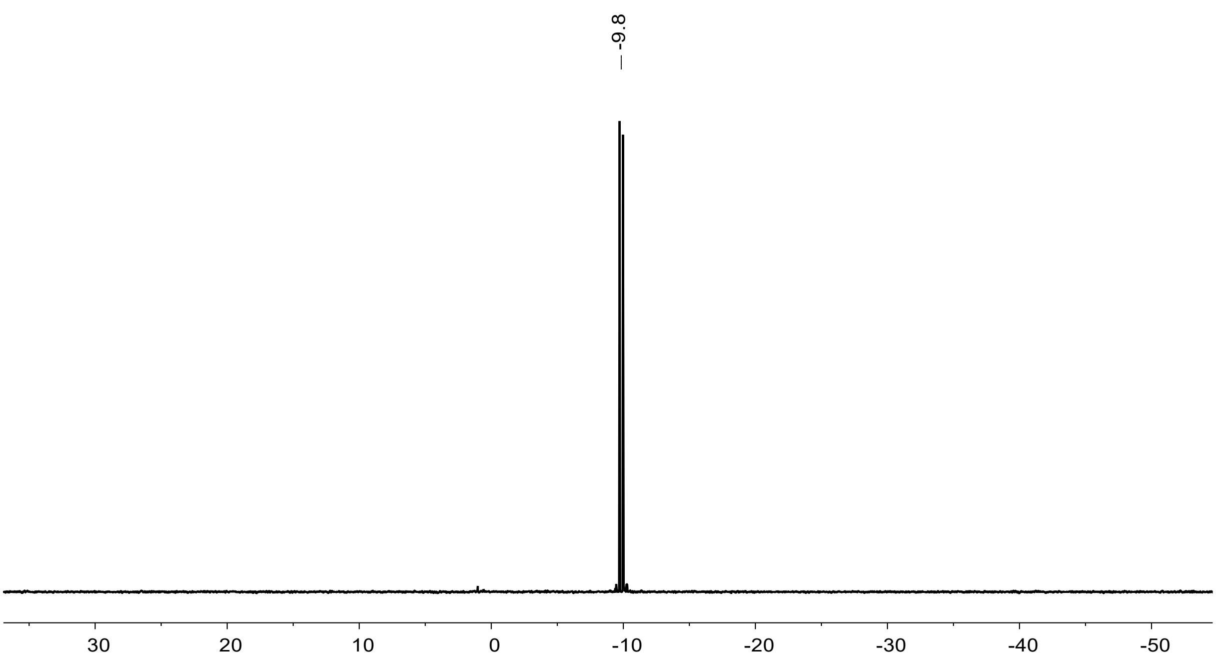

Figure S54. $^{29}Si\{^1H\}$ INEPT NMR spectrum (99.3 MHz, 305 K, benzene-d_6) of phosphole **23a**.

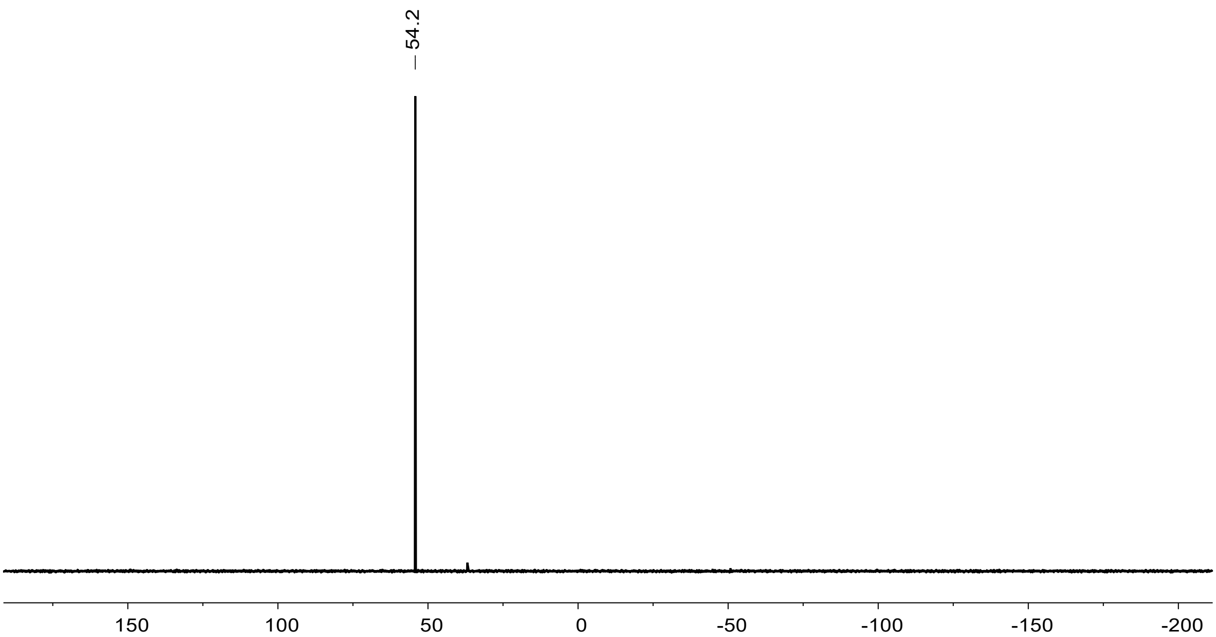

Figure S55. $^{31}P\{^1H\}$ NMR spectrum (202.4 MHz, 305 K, benzene-d_6) of phosphole **23a**.

Germylene 18b and Phosphole 23b

The product mixture contained phosphole **23b** and germylene **18b** (alongside smaller impurities) in a mixture of 14 : 86, in an overall yield of 73%.

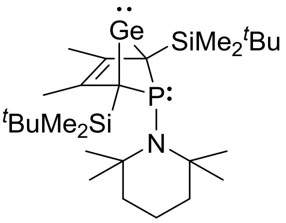

^{1}H NMR (499.9 MHz, 305 K, C_6D_6): δ = 0.26-0.27 (m, 6H, Si$\underline{Me_2}{}^t$Bu), 0.41 (s, 6H, Si$\underline{Me_2}{}^t$Bu), 1.01 (s, 3H, TMP-$\underline{Me}$), 1.06 (s, 3H, TMP-$\underline{Me}$), 1.10 (s, 18H, SiMe$_2\underline{^t Bu}$), 1.37-1.41 (m, 6H, TMP), 1.73 (s, 6H, 2 x TMP-$\underline{Me}$), 2.24 (s, 6H, Me).
^{13}C{^{1}H} NMR (125.7 MHz, 305 K, C_6D_6): δ = -1.9 (d, $^3J_{C,P}$ = 10 Hz, Si$\underline{Me_2}{}^t$Bu), -0.5 (d, $^3J_{C,P}$ = 5 Hz, Si$\underline{Me_2}{}^t$Bu), 18.0 (d, $^3J_{C,P}$ = 5 Hz, C$^{2/3}$-$\underline{Me}$), 18.2 (TMP, γ-$\underline{C}$), 19.5 (d, $^3J_{C,P}$ = 3 Hz, SiMe$_2\underline{^t Bu}$, ^{4}C), 28.7 (SiMe$_2\underline{^t Bu}$), 33.9 (2 x TMP-$\underline{Me}$), 35.1 (d, $^3J_{C,P}$ = 26 Hz, 2 x TMP-$\underline{Me}$), 42.3 (d, $^3J_{C,P}$ = 1 Hz, TMP, β-$\underline{C}$), 42.7 (d, $^3J_{C,P}$ = 2 Hz, TMP, β-$\underline{C}$), 55.6 (d, $^2J_{C,P}$ = 12 Hz, TMP, α-$\underline{C}$), 57.9 (d, $^2J_{C,P}$ = 25 Hz, TMP, α-$\underline{C}$), 81.6 (d, $^1J_{C,P}$ = 53 Hz, C$^{1/4}$), 135.1 (d, $^2J_{C,P}$ = 8 Hz, C$^{2/3}$).
^{29}Si{^{1}H} INEPT NMR (99.3 MHz, 305 K, C_6D_6): δ = 0.2 (d, $^2J_{Si,P}$ = 21 Hz).
^{31}P{^{1}H} NMR (202.4 MHz, 305 K, C_6D_6): δ = 39.7.

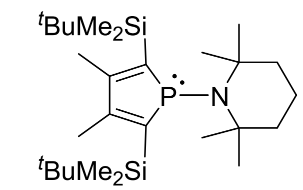

^{1}H NMR (499.9 MHz, 305 K, C_6D_6): δ = 0.33 (s, 3H, Si$\underline{Me_2}{}^t$Bu), 0.53 (s, 9H, Si$\underline{Me_2}{}^t$Bu), 1.09 (s, 18H, SiMe$_2\underline{^t Bu}$), 2.13 (d, $^4J_{H,P}$ = 7.5 Hz, 6H, C$^{2/3}$-$\underline{Me}$).
^{13}C{^{1}H} NMR (125.7 MHz, 305 K, C_6D_6): δ = 0.0 (d, $^3J_{C,P}$ = 3 Hz, Si$\underline{Me_2}{}^t$Bu), 19.6 (SiMe$_2\underline{^t Bu}$, ^{4}C), 20.1 (d, $^3J_{C,P}$ = 7 Hz, C$^{2/3}$-$\underline{Me}$), 28.7 (SiMe$_2\underline{^t Bu}$), 139.7 (d, $^1J_{C,P}$ = 39 Hz, C$^{1/4}$), 148.9 (d, $^2J_{C,P}$ = 23 Hz, C$^{2/3}$). Assignment of the TMP signals was not done due to the low concentration of the phosphole.
^{29}Si{^{1}H} INEPT NMR (99.3 MHz, 305 K, C_6D_6): δ = -1.3 (d, $^2J_{Si,P}$ = 22 Hz).
^{31}P{^{1}H} NMR (202.4 MHz, 305 K, C_6D_6): δ = 61.2.

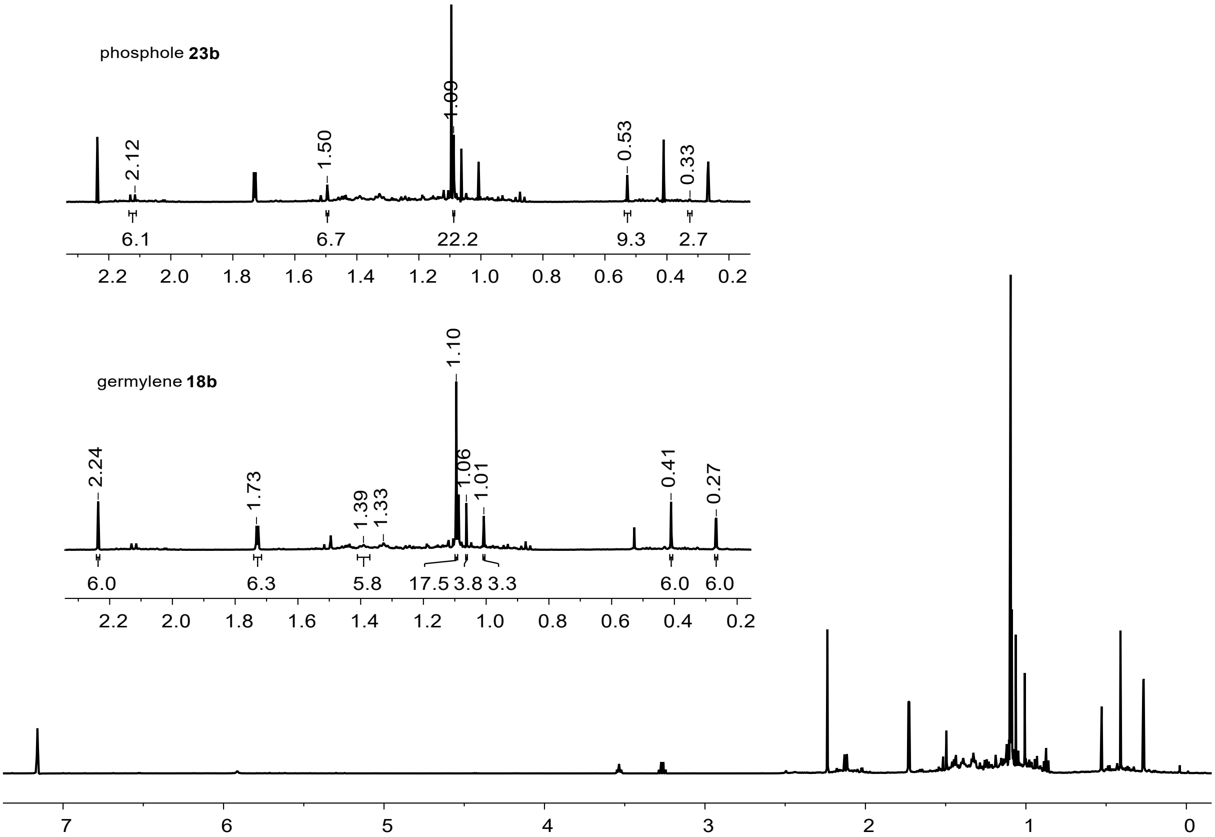

Figure S56. ^{1}H NMR spectrum (500.1 MHz, 305 K, benzene-d$_6$) of germylene **18b** (highlighted in lower zoom) and phosphole **23b** (highlighted in upper zoom).

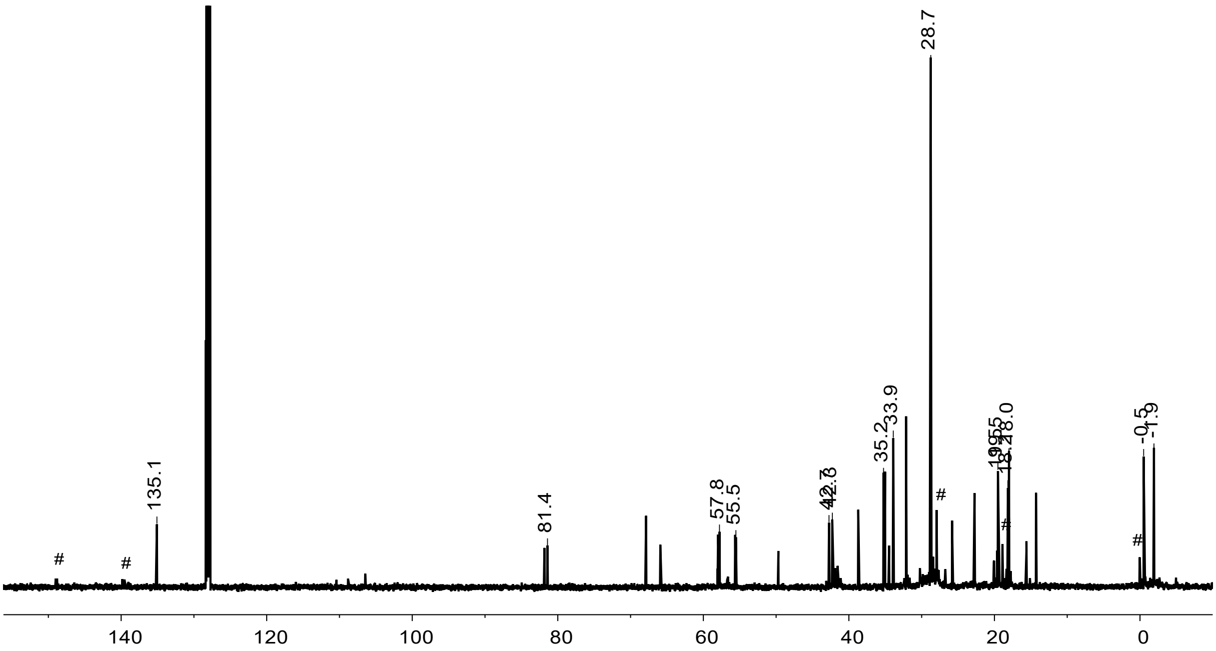

Figure S57. $^{13}C\{^{1}H\}$ NMR spectrum (125.7 MHz, 305 K, benzene-d$_6$) of germylene **18b** and phosphole **23b**(#).

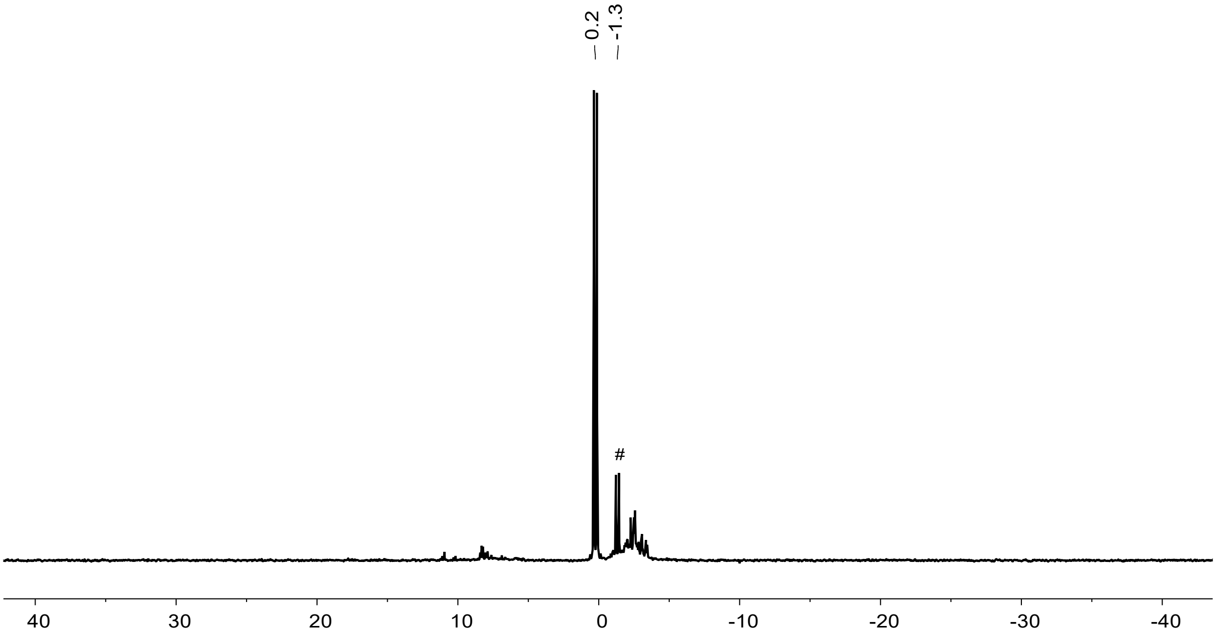

Figure S58. $^{29}Si\{^{1}H\}$ INEPT NMR spectrum (99.3 MHz, 305 K, benzene-d$_6$) of germylene **18b** and phosphole **23b**(#).

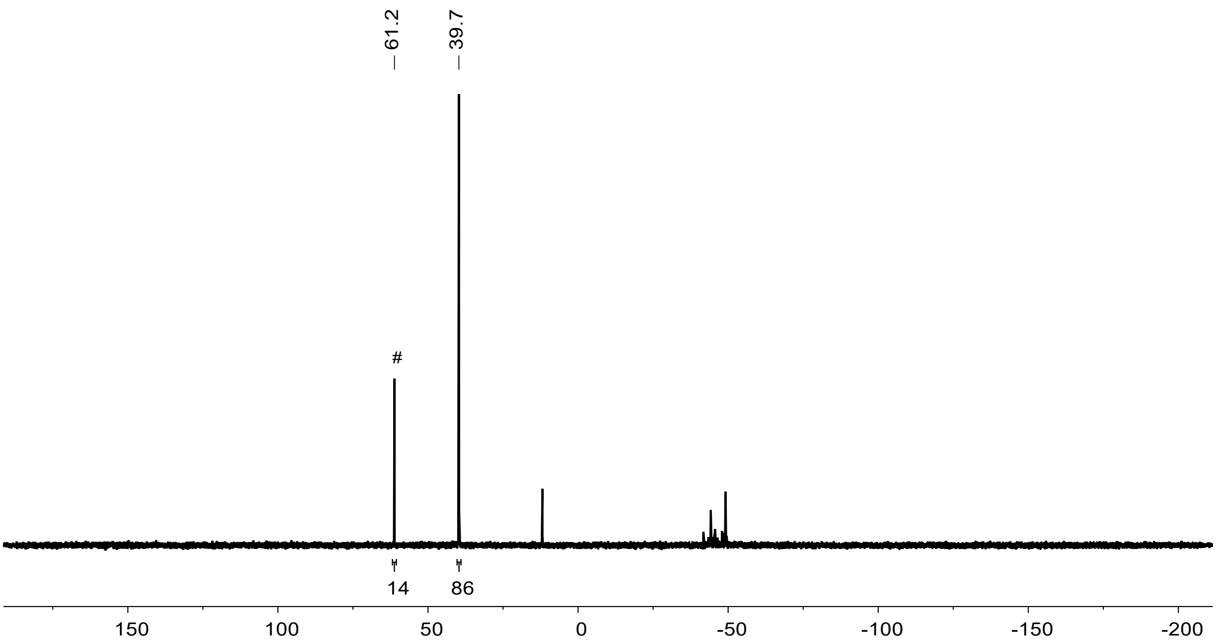

Figure S59. $^{31}P\{^1H\}$ NMR spectrum (202.4 MHz, 305 K, benzene-d$_6$) of germylene **18b** and phosphole **23b**(#).

Reactivity Studies of the germylenes.

General procedure for $SiMe_3$ – substituted germylenes: Germylenes **15a** and **16a** were reacted *in situ* with the different reagents. Therefore, the procedures described above were adapted but the reaction time was lowered to 10 minutes of stirring. The solvent was either removed and exchanged in the cold or the reagents were added directly to the THF solution.

General procedure $SiMe_2{}^tBu$– substituted germylenes: Germylenes **15b** and **16b** were synthesised as described above. Toluene solutions of the concentration c = 0.1 mol L^{-1} were prepared and stored at -20°C. They were used within 2 weeks after synthesis.

Phosphonium-BCH-germylene gallate [25a][$GaCl_4$]

A diethyl ether solution of H^+ $GaCl_4^-$, prepared from galliumtrichloride (35 mg, 0.20 mmol) and HCl (2M in Et_2O, 0.1 mL, 0.20 mmol), was added. The solution turned yellowish immediately. The cold bath was removed and the solution was stirred for 30 minutes at room temperature. Afterwards, the solvent was removed. The residue was dissolved in diethyl ether and filtered over a PTFE syringe filter. Recrystallisation from toluene yielded phosphonium gallate **[25a][$GaCl_4$]** (24 mg, 0.04 mmol, 20%) as colourless crystals.

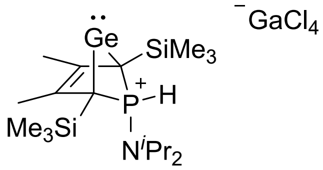

^{1}H NMR (500.1 MHz, 305 K, C_6D_6): δ = 0.35 (s, 18H, 2 x Si<u>Me</u>$_3$), 0.80 (d, $^3J_{H,H}$ = 6.8 Hz, 12 H, N(CH<u>Me</u>$_2$)$_2$), 1.91 (d, $^4J_{H,P}$ = 0.9 Hz, 6H, C$^{2/3}$-<u>Me</u>), 2.99 (dsept, $^3J_{H,P}$ = 15.0 Hz, $^3J_{H,H}$ = 6.8 Hz, 2H, N(C<u>H</u>Me$_2$)$_2$), 7.97 (d, $^1J_{H,P}$ = 569.1 Hz, 1H, P-<u>H</u>).

^{13}C{^{1}H} NMR (125.7 MHz, 305 K, C_6D_6): δ = 1.0 (d, $^{3}J_{C,P}$ = 4 Hz, Si<u>Me</u>$_3$), 15.0 (d, $^{3}J_{C,P}$ = 6 Hz, $C^{2/3}$-<u>Me</u>), 23.6 (d, $^{3}J_{C,P}$ = 2 Hz, N(CH<u>Me</u>$_2$)$_2$), 48.4 (d, $^{2}J_{C,P}$ = 2 Hz, N(<u>C</u>HMe$_2$)$_2$), 59.7 (d, $^{1}J_{C,P}$ = 48 Hz, $C^{1/4}$), 124.5 (d, $^{2}J_{C,P}$ = 9 Hz, $C^{2/3}$).
^{29}Si{^{1}H} INEPT NMR (99.3 MHz, 305 K, C_6D_6): δ = -8.1 (d, $^{2}J_{Si,P}$ = 3 Hz).
^{31}P NMR (202.4 MHz, 305 K, C_6D_6): δ = 31.0 (dt, $^{1}J_{P,H}$ = 569 Hz (P-<u>H</u>), $^{3}J_{P,H}$ = 15 Hz (P-N(C<u>H</u>Me$_2$)$_2$)).
^{71}Ga{^{1}H} NMR (152.5 MHz, 305 K, C_6D_6): δ = 249.9 (br).

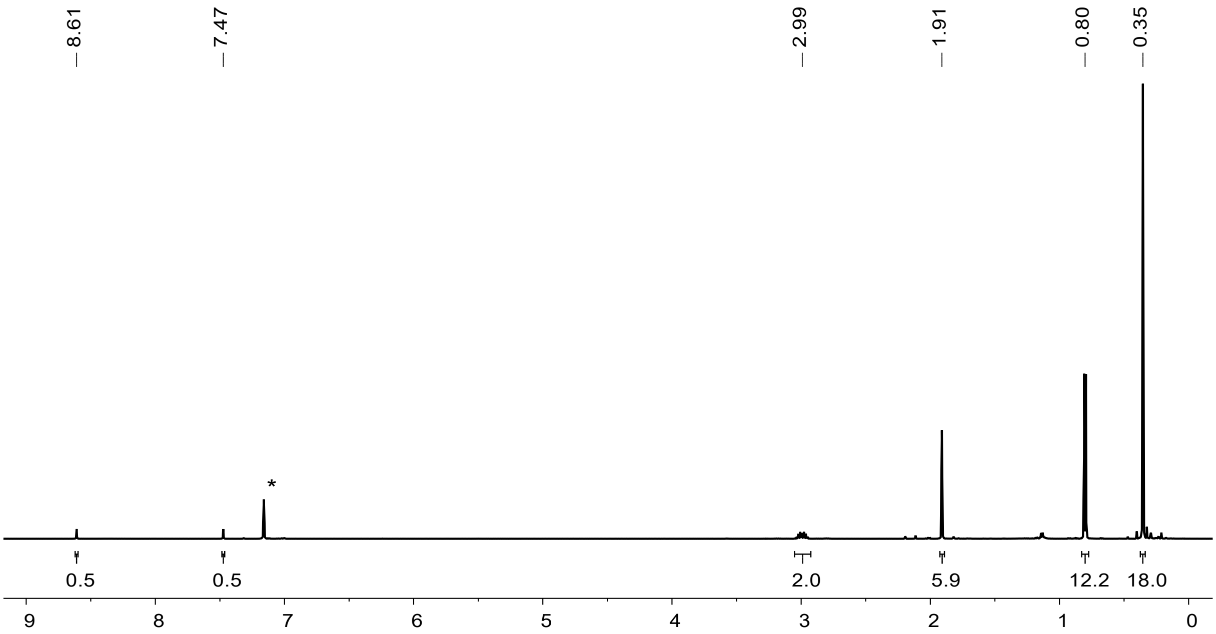

Figure S60. ^{1}H NMR spectrum (500.1 MHz, 305 K, benzene-d_6) of phosphonium gallate **[25a][GaCl$_4$]**.

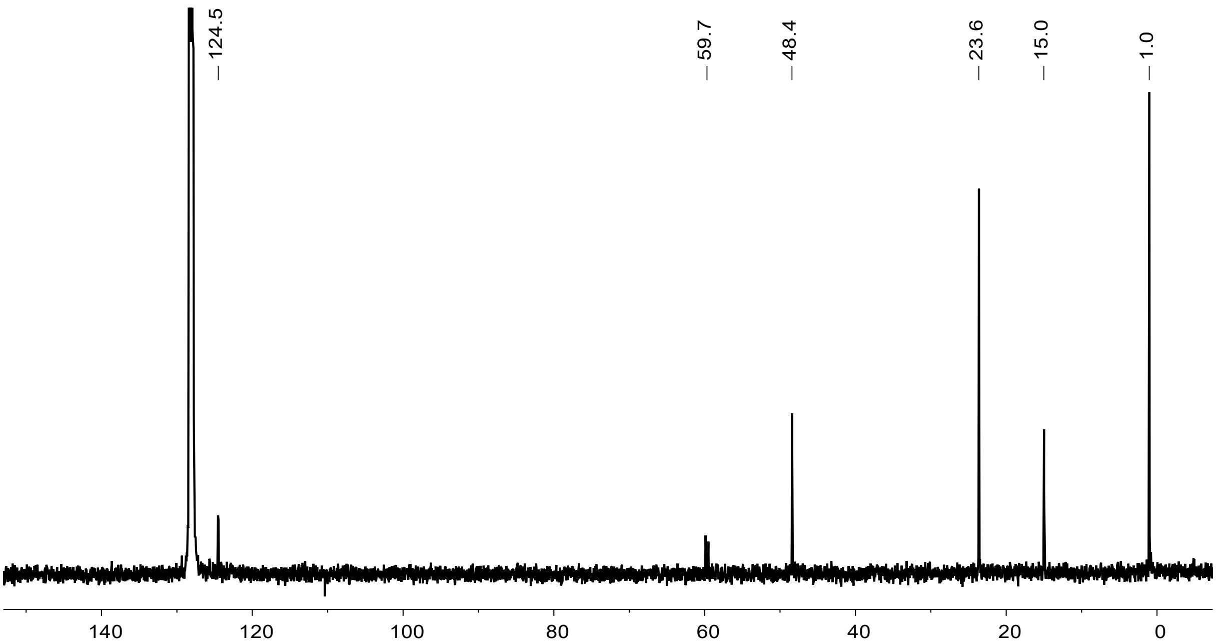

Figure S61. ^{13}C{^{1}H} NMR spectrum (125.7 MHz, 305 K, benzene-d_6) of phosphonium gallate **[25a][GaCl$_4$]**.

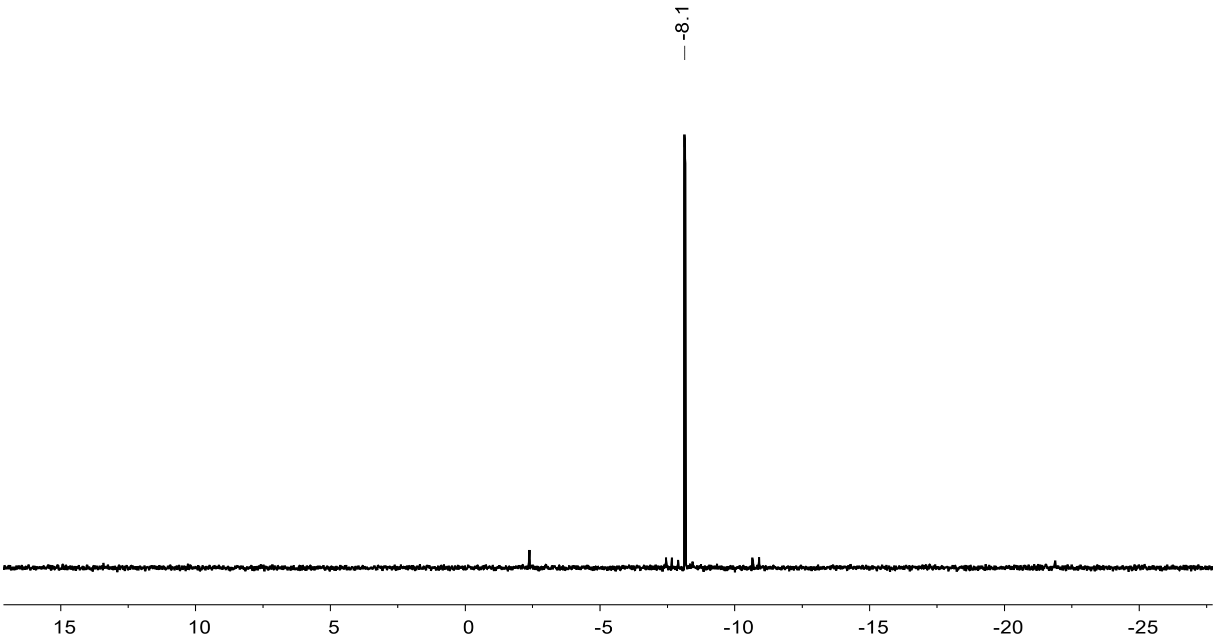

Figure S62. $^{29}Si\{^{1}H\}$ INEPT NMR spectrum (99.3 MHz, 305 K, benzene-d_6) of phosphonium gallate **[25a][GaCl$_4$]**.

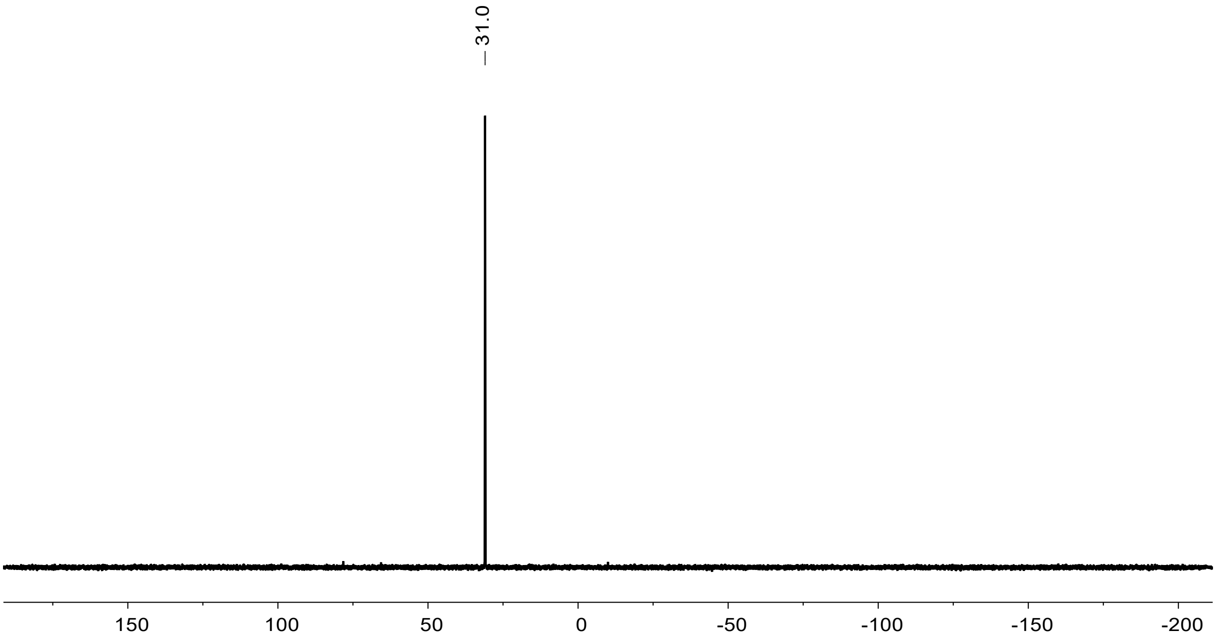

Figure S63. $^{31}P\{^{1}H\}$ NMR spectrum (202.4 MHz, 305 K, benzene-d_6) of phosphonium gallate **[25a][GaCl$_4$]**.

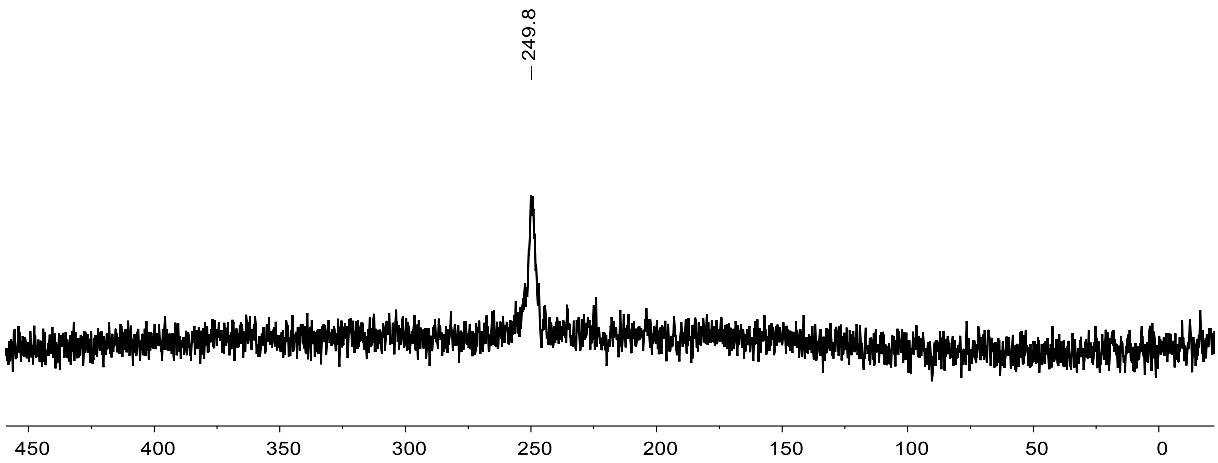

Figure S64. ^{71}Ga{^{1}H} NMR spectrum (152.5 MHz, 305 K, benzene-d$_6$) of phosphonium gallate **[25a][GaCl$_4$]**.

Phosphonium-BCH-germylene borate [25a][B(C_6F_5)$_4$]

Hydrochloric acid (2M in Et_2O, 0.1 mL) was added to the freshly prepared germylene **15a**. It was stirred for 10 minutes at that temperature. Afterwards, the solvent was removed. The residue was dissolved in chlorobenzene-d$_5$ (1.5 mL) and added to a Schlenk flask, equipped with potassium borate K[B(C_6F_5)$_4$] (144 mg, 0.20 mmol). The solution was stirred overnight at room temperature, before NMR spectra were recorded. Removal of the solvent gave phosphonium-BCH-germylene borate **[25a][B(C_6F_5)$_4$]** (177 mg, 0.16 mmol, 80%) as slightly yellow solid.

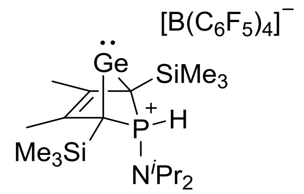

^{1}H NMR (500.1 MHz, 305 K, C_6D_5Cl): δ = 0.04 (s, 18H, 2 x Si$\underline{\text{Me}}_3$), 0.83 (d, $^3J_{H,H}$ = 6.8 Hz, 12H, N(CH$\underline{\text{Me}}_2$)$_2$), 2.03 (s, 6H, C$^{2/3}$–$\underline{\text{Me}}$), 2.83-3.05 (m, 2H, N(C$\underline{\text{H}}$Me$_2$)$_2$), 6.86 (d, $^1J_{H,P}$ = 568.8 Hz, 1H, P-H).
^{13}C{^{1}H} NMR (125.7 MHz, 305 K, C_6D_5Cl): δ = 0.0 (d, $^3J_{C,P}$ = 3 Hz, Si$\underline{\text{Me}}_3$), 14.4 (d, $^3J_{C,P}$ = 5 Hz, C$^{2/3}$–$\underline{\text{Me}}$), 23.5 (N(CH$\underline{\text{Me}}_2$)$_2$), 49.1-49.2 (m, N($\underline{\text{C}}$HMe$_2$)$_2$), 70.2 (d, $^1J_{C,P}$ = 52 Hz, C$^{1/4}$), 127.7 (d, $^2J_{C,P}$ = 14 Hz, C$^{2/3}$).
^{29}Si{^{1}H} INEPT NMR (99.3 MHz, 305 K, C_6D_5Cl): δ = -6.3 (d, $^2J_{Si,P}$ = 4 Hz).
^{31}P{^{1}H} NMR (202.4 MHz, 305 K, C_6D_5Cl): δ = 14.5.
^{11}B{^{1}H} NMR (160.4 MHz, 305 K, C_6D_5Cl): δ = -16.0.

^{1}H NMR (500.1 MHz, 305 K, THF-d$_8$): δ = 0.30 (s, 18H, 2 x Si$\underline{\text{Me}}_3$), 1.21 (d, $^3J_{H,H}$ = 6.9 Hz, 12H, N(CH$\underline{\text{Me}}_2$)$_2$), 2.17 (s, 6H, C$^{2/3}$–$\underline{\text{Me}}$), 3.46-3.54 (m, 2H, N(C$\underline{\text{H}}$Me$_2$)$_2$), 7.88 (d, $^1J_{H,P}$ = 579.7 Hz, 1H, P-H).
^{13}C{^{1}H} NMR (125.7 MHz, 305 K, THF-d$_8$): δ = 1.0 (d, $^3J_{C,P}$ = 4 Hz, Si$\underline{\text{Me}}_3$), 15.1 (d, $^3J_{C,P}$ = 6 Hz, C$^{2/3}$–$\underline{\text{Me}}$), 24.2 (d, $^3J_{C,P}$ = 2 Hz, N(CH$\underline{\text{Me}}_2$)$_2$), 49.4 (d, $^2J_{C,P}$ = 2 Hz, N($\underline{\text{C}}$HMe$_2$)$_2$), 60.6 (d, $^1J_{C,P}$ = 49 Hz, C$^{1/4}$), 125.6 (d, $^2J_{C,P}$ = 9 Hz, C$^{2/3}$).
^{29}Si{^{1}H} INEPT NMR (99.3 MHz, 305 K, THF-d$_8$): δ = -7.6 (d, $^2J_{Si,P}$ = 3 Hz).
^{31}P{^{1}H} NMR (202.4 MHz, 305 K, THF-d$_8$): δ = 28.4.
^{11}B{^{1}H} NMR (160.4 MHz, 305 K, THF-d$_8$): δ = -16.6.

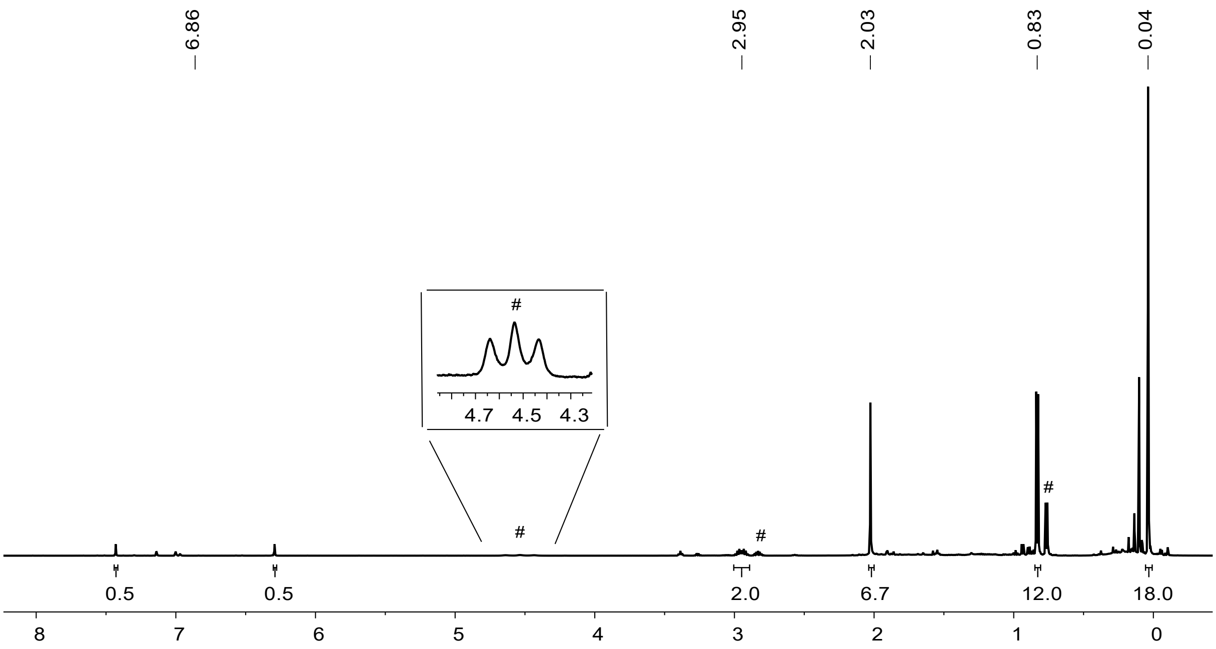

Figure S65. ^{1}H NMR spectrum (500.1 MHz, 305 K, chlorobenzene-d_5) of phosphonium borate **[25a][B(C$_6$F$_5$)$_4$]** ($^{\#}$[$H_2N^{i}Pr_2$]$^{+}$).

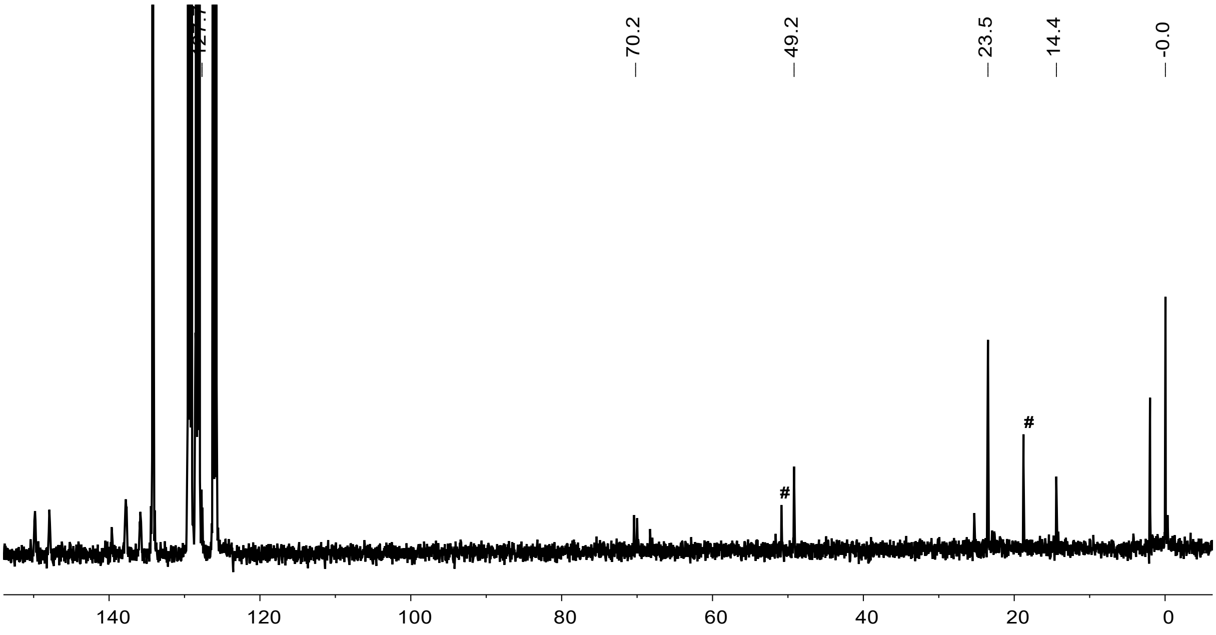

Figure S66. $^{13}C\{^{1}H\}$ NMR spectrum (125.7 MHz, 305 K, chlorobenzene-d_5) of phosphonium borate **[25a][B(C$_6$F$_5$)$_4$]** ($^{\#}$[$H_2N^{i}Pr_2$]$^{+}$).

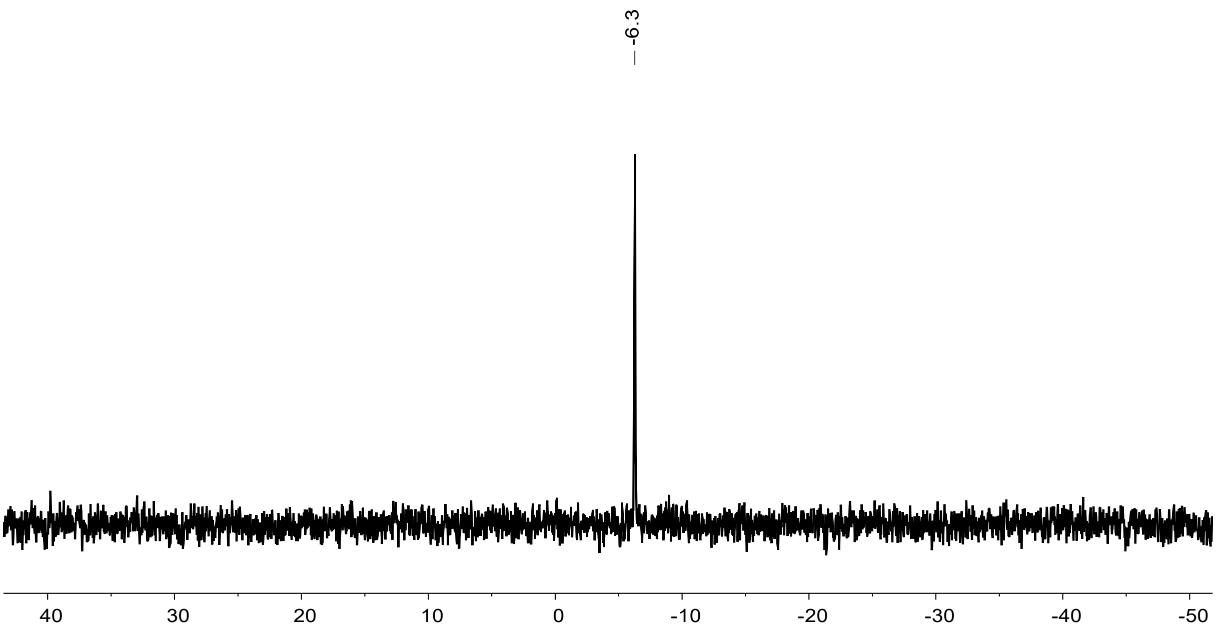

Figure S67. $^{29}Si\{^{1}H\}$ INEPT NMR spectrum (99.3 MHz, 305 K, chlorobenzene-d_5) of phosphonium borate **[25a][B(C_6F_5)$_4$]**.

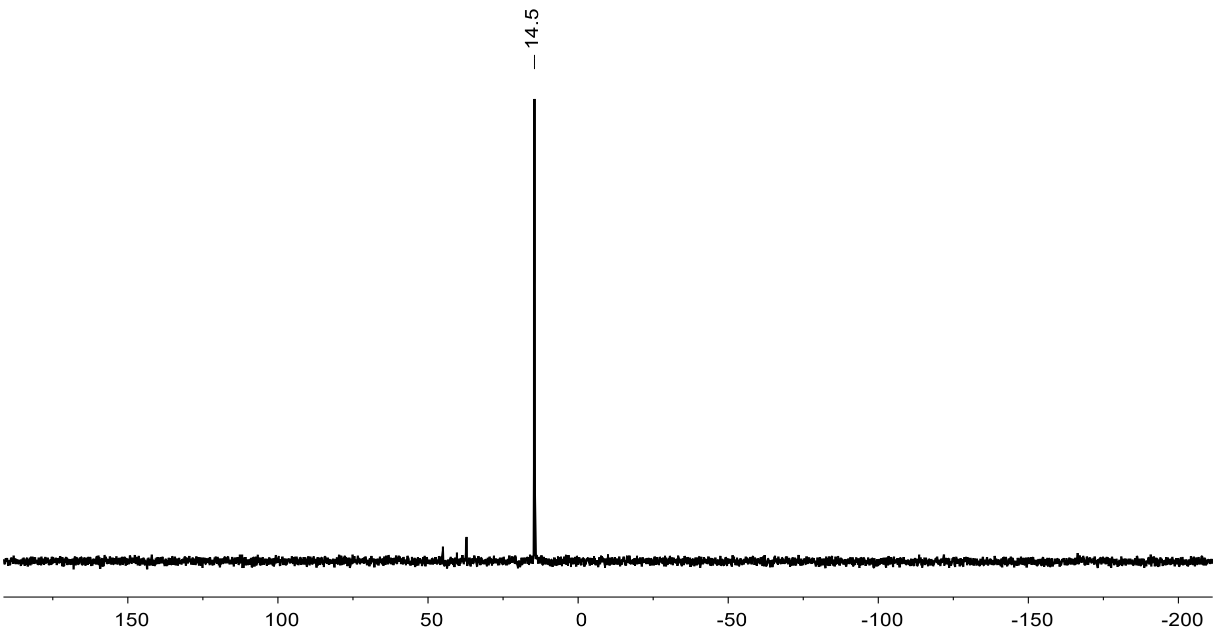

Figure S68. $^{31}P\{^{1}H\}$ NMR spectrum (202.4 MHz, 305 K, chlorobenzene-d_5) of phosphonium borate **[25a][B(C_6F_5)$_4$]**.

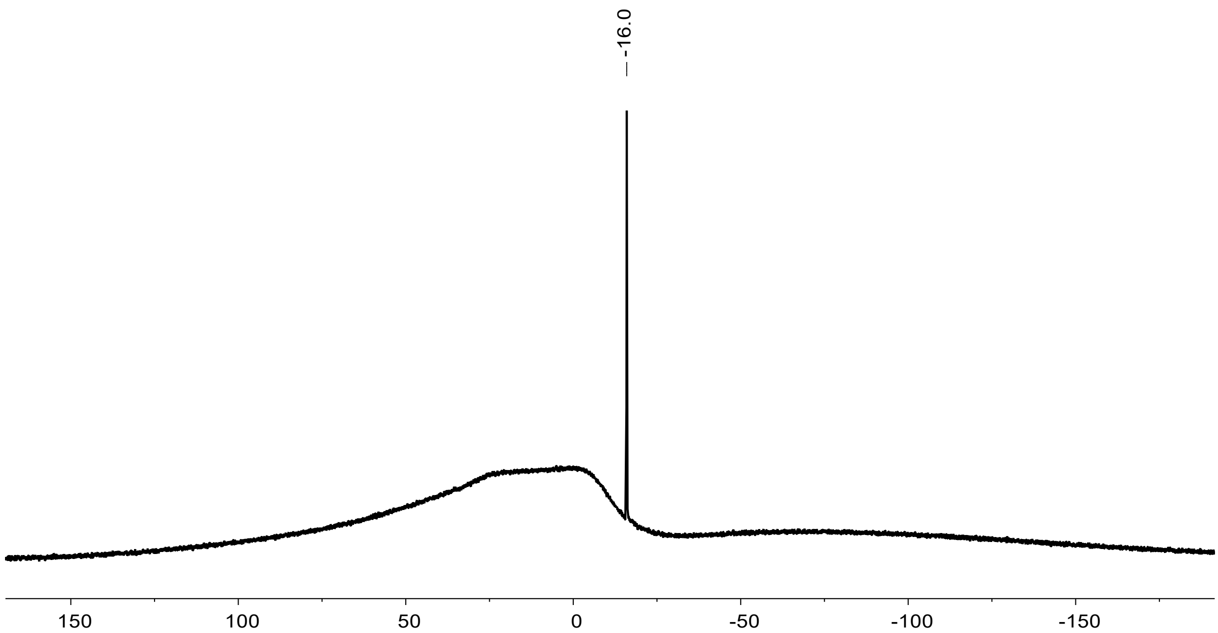

Figure S69. $^{11}B\{^1H\}$ NMR spectrum (160.4 MHz, 305 K, chlorobenzene-d$_5$) of phosphonium borate **[25a][B(C$_6$F$_5$)$_4$]**.

Phosphonium-BCH-germylene [26a][B(C$_6$F$_5$)$_4$]

Hydrochloric acid (2M in Et_2O, 0.2 mL) was added to the freshly prepared germylene **16a**. It was stirred for 10 minutes at that temperature. Afterwards, the solvent was removed. The residue was dissolved in toluene (5 mL) and added to a Schlenk flask equipped with potassium borate (288 mg, 0.40 mmol). The solution was stirred overnight at room temperature. The precipitates settled to the bottom and the solution was transferred into another flask. The solvent was removed and phosphonium-BCH-germylene borate **[26a][B(C$_6$F$_5$)$_4$]** (368 mg, 0.31 mmol, 78%) was obtained as slightly yellow solid.

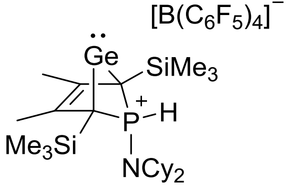

^{1}H NMR (500.1 MHz, 305 K, C_6D_5Cl): δ = 0.06 (s, 18H, 2 x Si<u>Me</u>$_3$), 0.77-0.88 (m, 2H, NCy$_2$), 0.98-1.09 (m, 4H, NCy$_2$), 1.09-1.19 (m, 4H, NCy$_2$), 1.38-1.45 (m, 4H, NCy$_2$), 1.45-1.51 (m, 2H, NCy$_2$), 1.60-1.67 (m, 4H, NCy$_2$), 2.05 (s, 6H, C$^{2/3}$–<u>Me</u>), 2.53-2.67 (m, 2H, NCy$_2$), 6.96 (d, $^1J_{H,P}$ = 567.0 Hz, 1H, P-H).
^{13}C{^{1}H} NMR (125.7 MHz, 305 K, C_6D_5Cl): δ = -0.1 (d, $^3J_{C,P}$ = 3 Hz, Si<u>Me</u>$_3$), 14.4 (d, $^3J_{C,P}$ = 5 Hz, C$^{2/3}$–<u>Me</u>), 25.1 (NCy$_2$), 26.2 (NCy$_2$), 35.0-35.1 (NCy$_2$), 58.5 (NCy$_2$), 69.9 (d, $^1J_{C,P}$ = 52 Hz, C$^{1/4}$), 127.6 (d, $^2J_{C,P}$ = 13 Hz, C$^{2/3}$).
^{29}Si{^{1}H} INEPT NMR (99.3 MHz, 305 K, C_6D_5Cl): δ = -6.4 (d, $^2J_{Si,P}$ = 4 Hz).
^{31}P{^{1}H} NMR (202.4 MHz, 305 K, C_6D_5Cl): δ = 13.4.

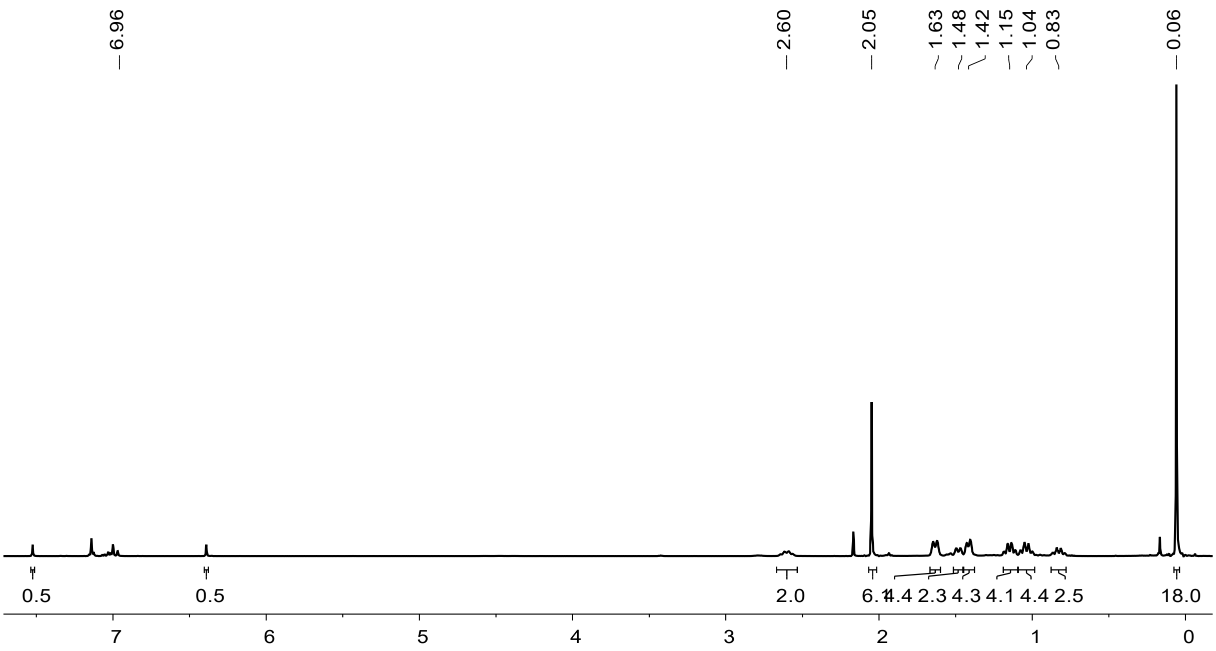

Figure S70. ^{1}H NMR spectrum (500.1 MHz, 305 K, chlorobenzene-d$_5$) of phosphonium borate **[26a][B(C$_6$F$_5$)$_4$]**.

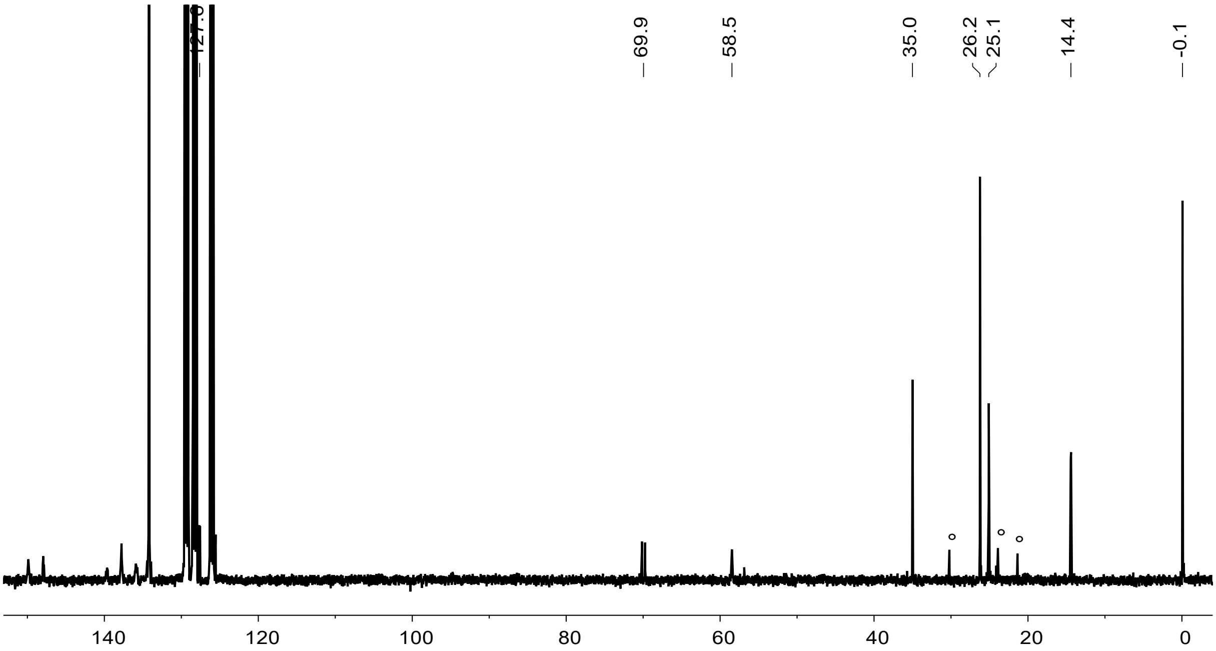

Figure S71. $^{13}C\{^{1}H\}$ NMR spectrum (125.7 MHz, 305 K, chlorobenzene-d$_5$) of phosphonium borate **[26a][B(C$_6$F$_5$)$_4$]** (°impurity).

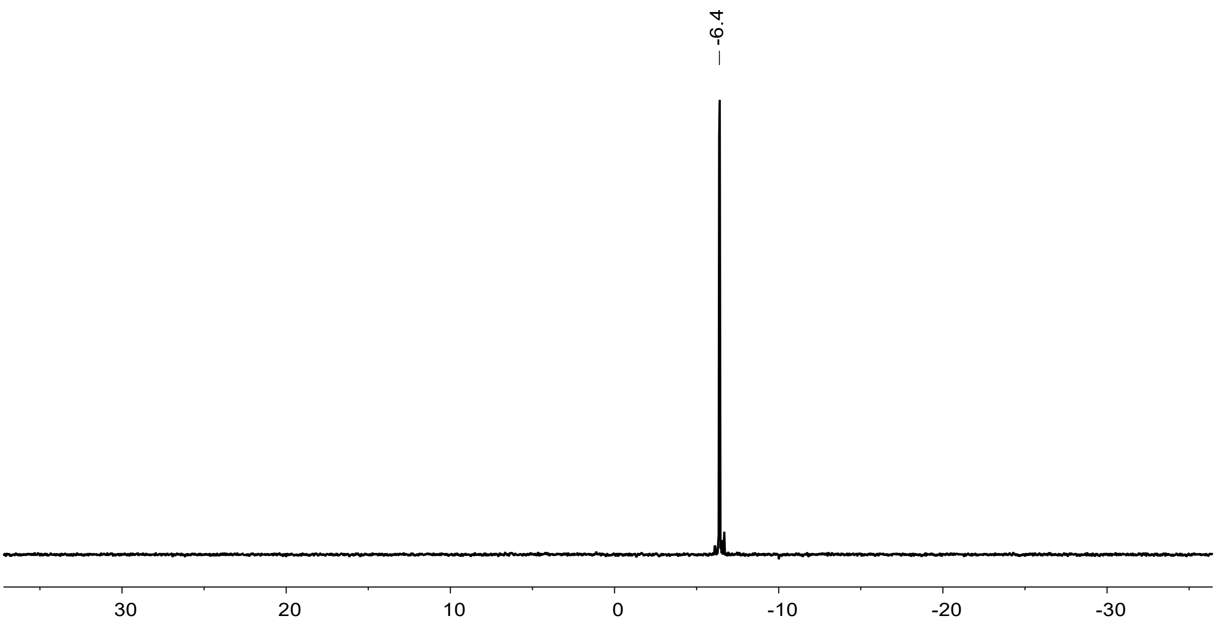

Figure S72. $^{29}Si\{^1H\}$ INEPT NMR spectrum (99.3 MHz, 305 K, chlorobenzene-d_5) of phosphonium borate **[26a][B(C_6F_5)$_4$]**.

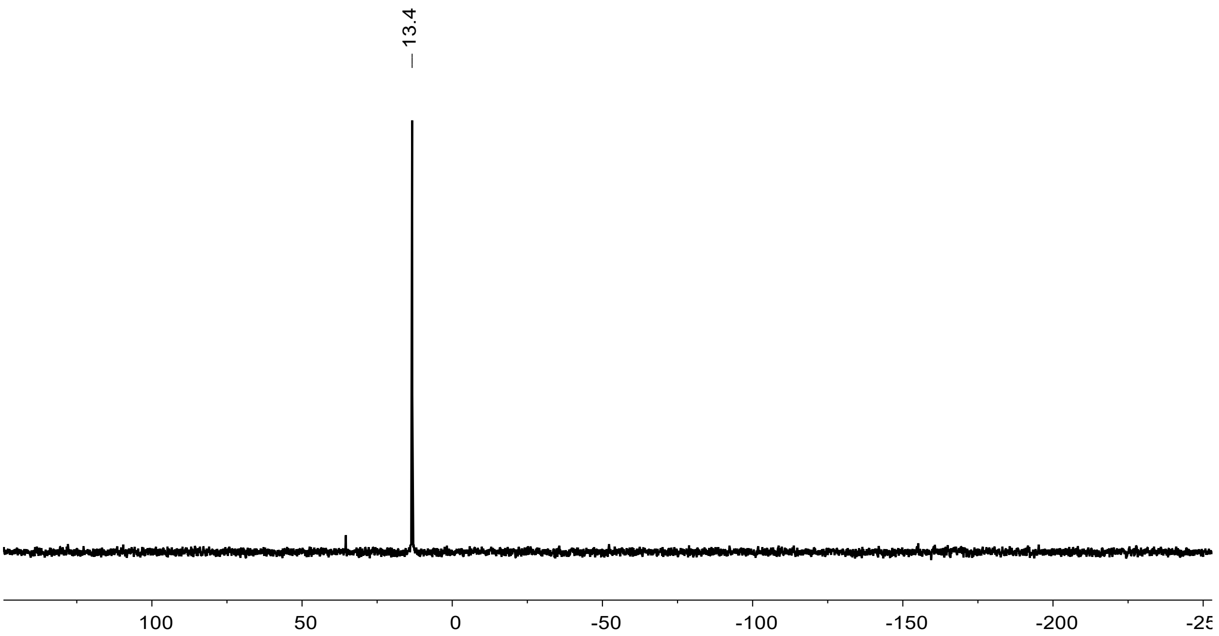

Figure S73. $^{31}P\{^1H\}$ NMR spectrum (202.4 MHz, 305 K, chlorobenzene-d_5) of phosphonium borate **[26a][B(C_6F_5)$_4$]**.

Phosphonium-BCH-germylene gallate [25b][$GaCl_4$]

To a toluene/diethyl ether (4 mL) solution of germylene **15b** (0.20 mmol), a diethyl ether solution of H^+ $GaCl_4^-$, prepared from galliumtrichloride (35 mg, 0.20 mmol) and HCl (2M in Et_2O, 0.1 mL, 0.20 mmol), was added. The solution turned yellow immediately. It was stirred for 5 minutes at -30°C and afterwards 30 minutes at room temperature. A colourless precipitate had formed during that time. The solvent was removed and the residue was washed with pentane and toluene. Phosphonium-BCH-germylene gallate **[25b][$GaCl_4$]** was isolated as colourless solid. After a couple of hours in THF solution, the compound started to decompose.

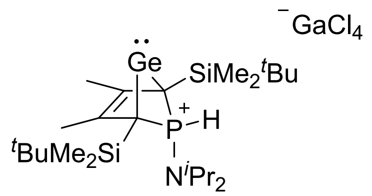

^{1}**H NMR** (500.1 MHz, 305 K, THF-d$_8$): δ = 0.39 (s, 6H, Si<u>Me$_2$</u>^{t}Bu), 0.42 (s, 6H, Si<u>Me$_2$</u>^{t}Bu), 1.03 (s, 18H, SiMe$_2$<u>^{t}Bu</u>), 1.28 (d, $^3J_{H,H}$ = 6.7 Hz, 12H, N(CH<u>Me$_2$</u>)$_2$), 2.53 (s, 6H, C$^{2/3}$-<u>Me</u>), 3.59-3.66 (m, 2H, N(C<u>H</u>Me$_2$)$_2$), 7.78 (d, $^1J_{H,P}$ = 580.3 Hz, 1H, P-<u>H</u>).

^{13}**C{^{1}H} NMR** (125.7 MHz, 305 K, THF-d$_8$): δ = -2.9 (d, $^3J_{C,P}$ = 1 Hz, Si<u>Me$_2$</u>^{t}Bu), -2.8 (d, $^3J_{C,P}$ = 4 Hz, Si<u>Me$_2$</u>^{t}Bu), 16.8 (d, $^3J_{C,P}$ = 5 Hz, C$^{2/3}$-<u>Me</u>), 19.4 (d, $^3J_{C,P}$ = 6 Hz, SiMe$_2$<u>^{t}Bu</u>, C^4), 24.4 (d, $^3J_{C,P}$ = 3 Hz, N(CH<u>Me$_2$</u>)$_2$), 28.3 (SiMe$_2$<u>^{t}Bu</u>), 50.5 (m, N(<u>C</u>HMe$_2$)$_2$), 67.8 (C$^{1/4}$, hidden under solvent signal), 129.3 (d, $^2J_{C,P}$ = 12 Hz, C$^{2/3}$).

^{29}**Si{^{1}H} INEPT NMR** (99.3 MHz, 305 K, THF-d$_8$): δ = 0.5 (d, $^2J_{Si,P}$ = 2 Hz).

^{31}**P NMR** (202.4 MHz, 305 K, THF-d$_8$): δ = 25.7 (dt, $^1J_{P,H}$ = 580 Hz (P-<u>H</u>), $^3J_{P,H}$ = 15 Hz (P-N(C<u>H</u>Me$_2$)$_2$)).

^{71}**Ga{^{1}H} NMR** (152.5 MHz, 305 K, THF-d$_8$): δ = 248.2.

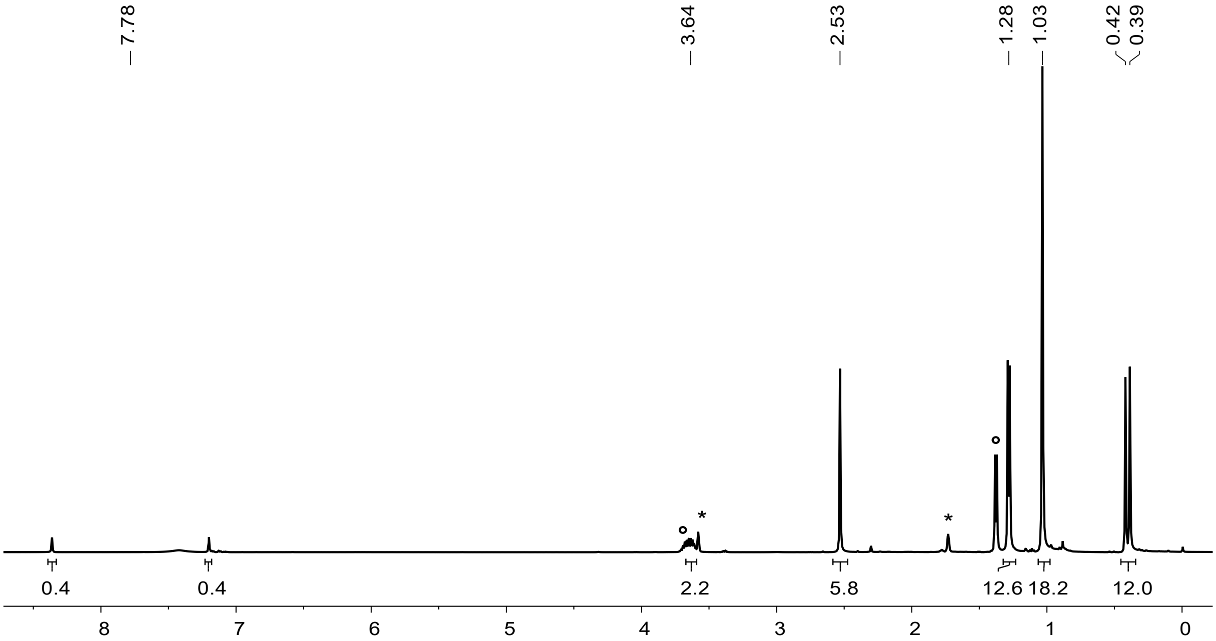

Figure S74. ^{1}H NMR spectrum (500.1 MHz, 305 K, THF-d$_8$) of phosphonium gallate **[25b][GaCl$_4$]** (*THF-d$_8$, ° impurity).

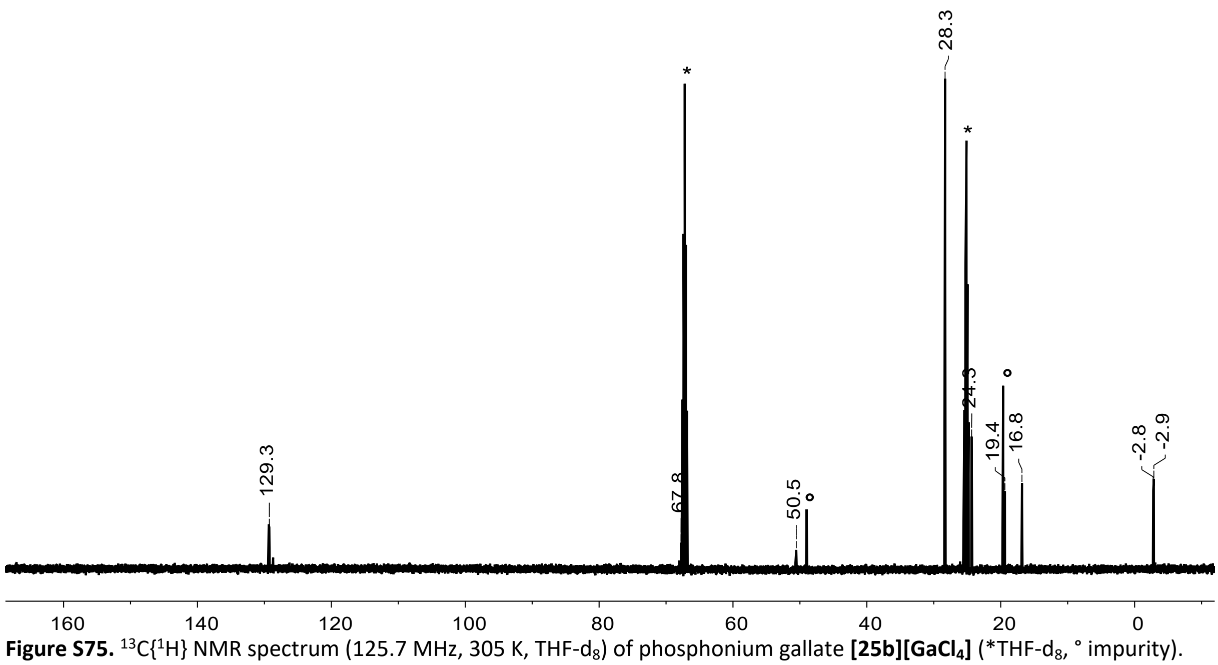

Figure S75. $^{13}C\{^{1}H\}$ NMR spectrum (125.7 MHz, 305 K, THF-d_8) of phosphonium gallate **[25b][GaCl$_4$]** (*THF-d_8, ° impurity).

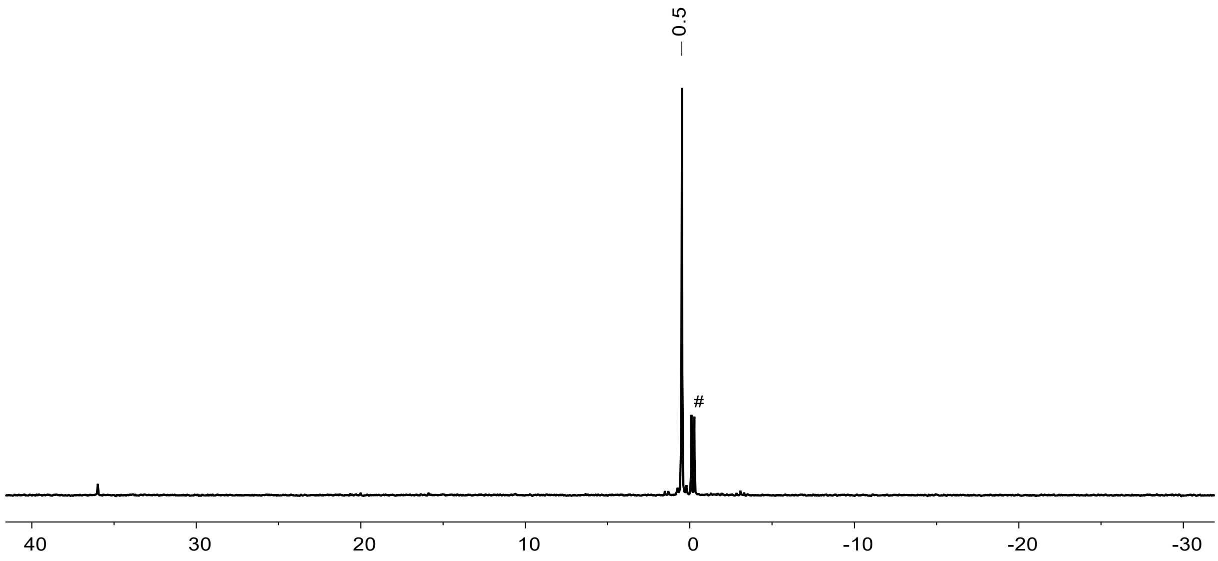

Figure S76. $^{29}Si\{^{1}H\}$ INEPT NMR spectrum (99.3 MHz, 305 K, THF-d_8) of phosphonium gallate **[25b][GaCl$_4$]** ($^{\#}$phosphole **20b**).

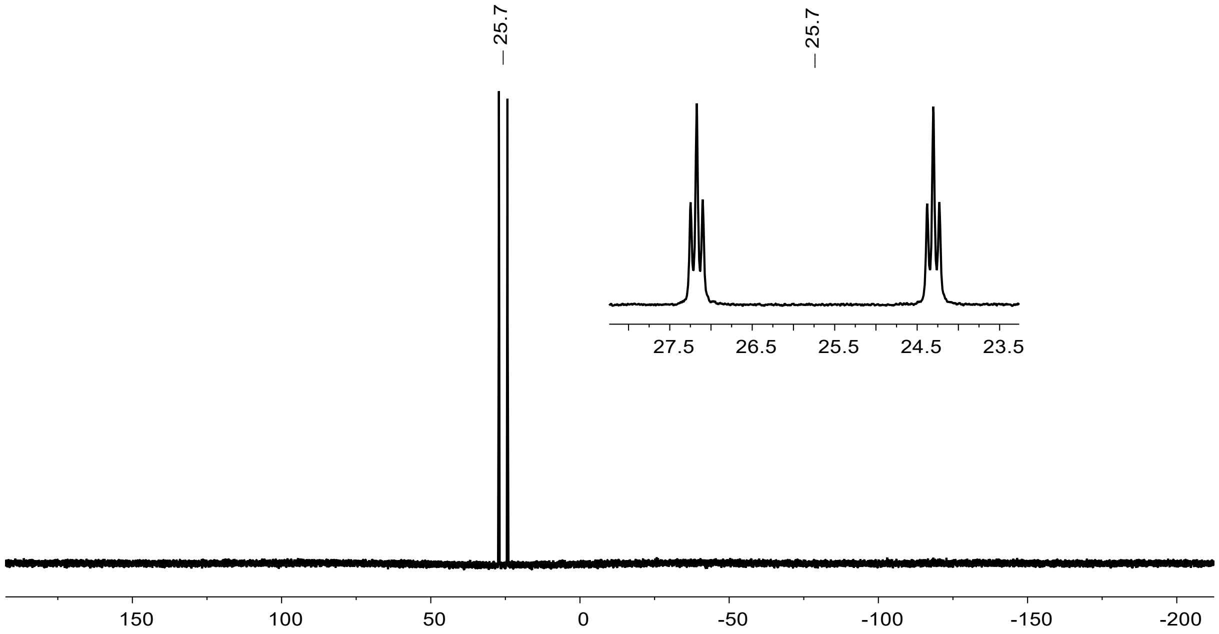

Figure S77. ^{31}P NMR spectrum (202.4 MHz, 305 K, THF-d_8) of phosphonium gallate **[25b][GaCl$_4$]**.

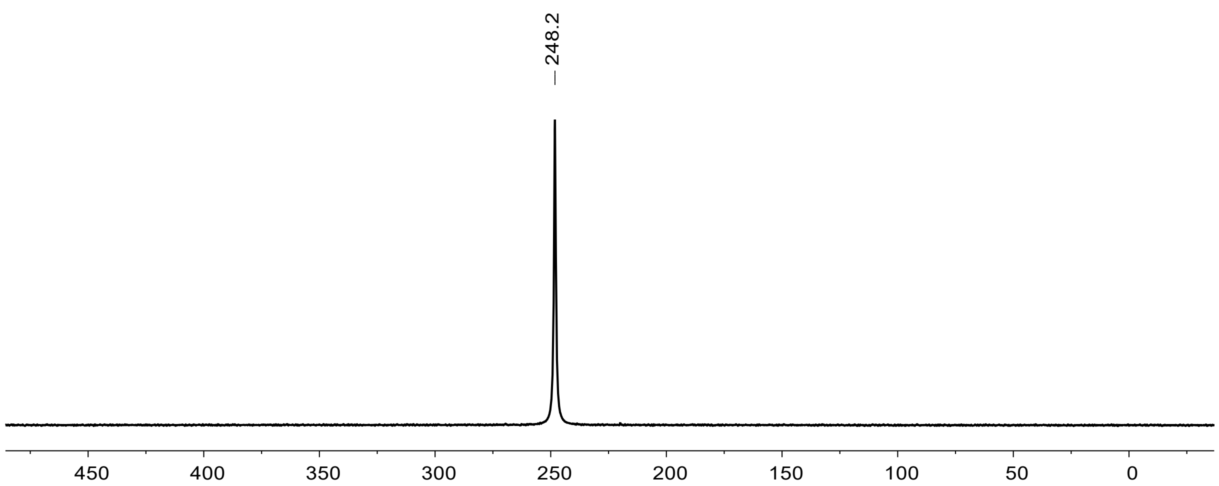

Figure S78. $^{71}Ga\{^1H\}$ NMR spectrum (152.5 MHz, 305 K, THF-d_8) of phosphonium gallate **[25b][GaCl$_4$]**.

Methylphosphonium-BCH-germylene [30a][OTf]

Germylene **15a** was prepared as described above. After addition of the dichlorophosphane, the mixture was only stirred for 5 minutes before the solvent was removed at -30°C. The residue was dissolved in pentane (6 mL) and methyl triflate (33 mg, 0.20 mmol) was added immediately after. It was stirred for two hours whilst the reaction was allowed to warm to room temperature. The methylphosphonium salt precipitates upon formation. The solution was removed via syringe and the residue was washed with pentane (2 x 3 mL). It was dried *in vacuo* to give methylphosphonium triflate **[30a][OTf]** as a colourless solid.

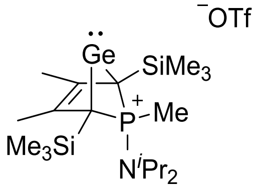

^{1}H NMR (500.1 MHz, 305 K, C_6D_5Cl): δ = 0.13 (s, 18H, 2 x Si<u>Me</u>$_3$), 0.90 (d, $^3J_{H,H}$ = 7.1 Hz, 12H, N(CH<u>Me</u>$_2$)$_2$), 2.07 (s, 6H, 2 x $C^{2/3}$–<u>Me</u>), 2.35 (d, $^2J_{H,P}$ = 12.6 Hz, 2H, P-<u>Me</u>), 3.03 (sept, $^3J_{H,H}$ = 7.1 Hz, 2H, N(C<u>H</u>Me$_2$)$_2$).
^{13}C{^{1}H} NMR (125.7 MHz, 305 K, C_6D_5Cl): δ = 0.2 (d, $^3J_{C,P}$ = 3 Hz, Si<u>Me</u>$_3$), 12.6 (d, $^1J_{C,P}$ = 92 Hz, P–<u>Me</u>), 14.3 (d, $^3J_{C,P}$ = 5 Hz, $C^{2/3}$–<u>Me</u>), 22.6 (N(CH<u>Me</u>$_2$)$_2$), 47.5 (N(<u>C</u>HMe$_2$)$_2$), 67.2 (d, $^1J_{C,P}$ = 57 Hz, $C^{1/4}$), 122.1 (q, $^1J_{C,F}$ = 322 Hz, <u>C</u>F$_3$), 125.4 (d, $^2J_{C,P}$ = 11 Hz, $C^{2/3}$).
^{29}Si{^{1}H} INEPT NMR (99.3 MHz, 305 K, C_6D_5Cl): δ = -5.9 (d, $^2J_{Si,P}$ = 5 Hz).
^{31}P{^{1}H} NMR (202.4 MHz, 305 K, C_6D_5Cl): δ = 54.5.

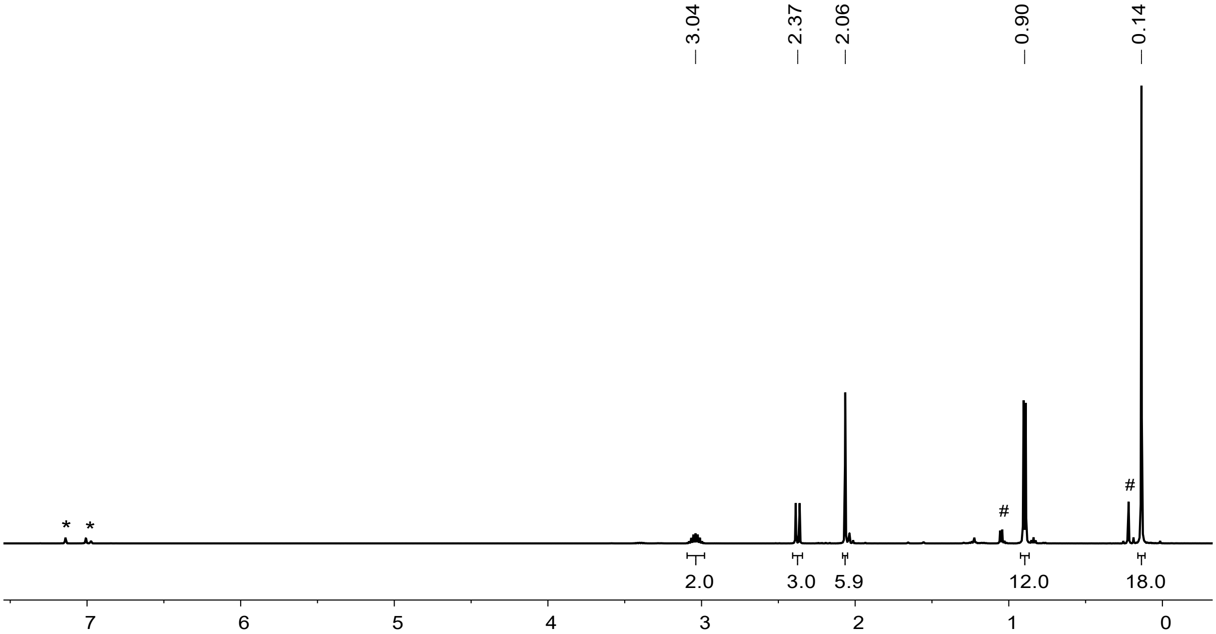

Figure S79. ^{1}H NMR spectrum (500.1 MHz, 305 K, chlorobenzene-d$_5$) of phosphonium triflate **[30a][OTf]** ($^\#$phospholium triflate **[31a][OTf]**).

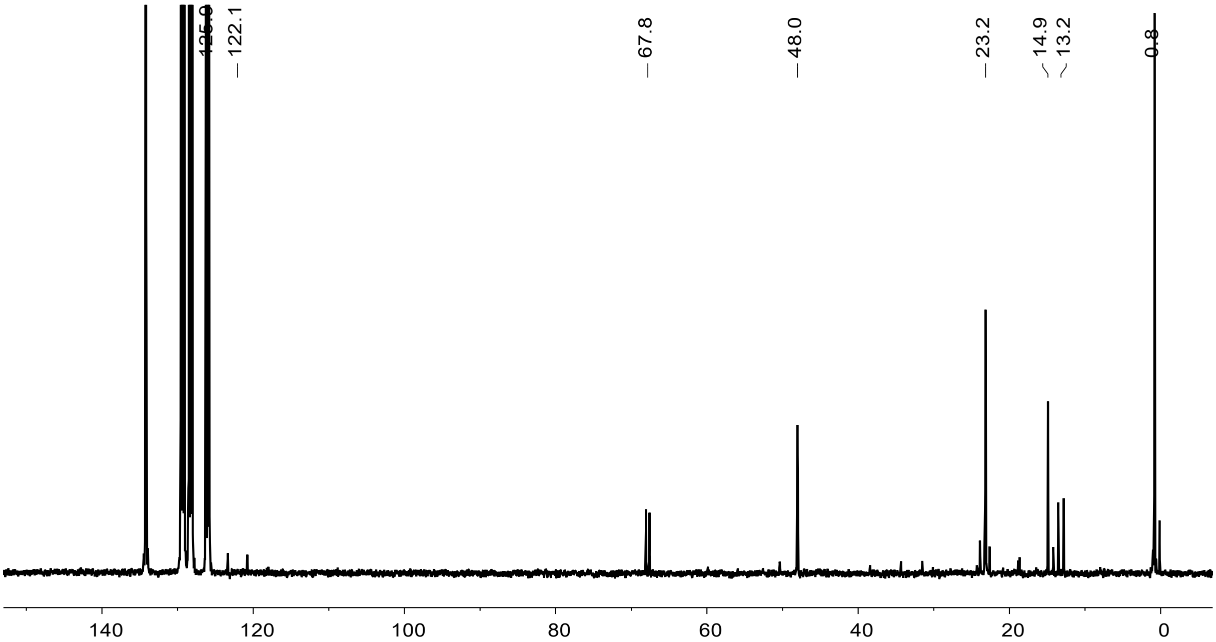

Figure S80. ^{13}C{^{1}H} NMR spectrum (125.7 MHz, 305 K, chlorobenzene-d$_5$) of phosphonium triflate **[30a][OTf]**.

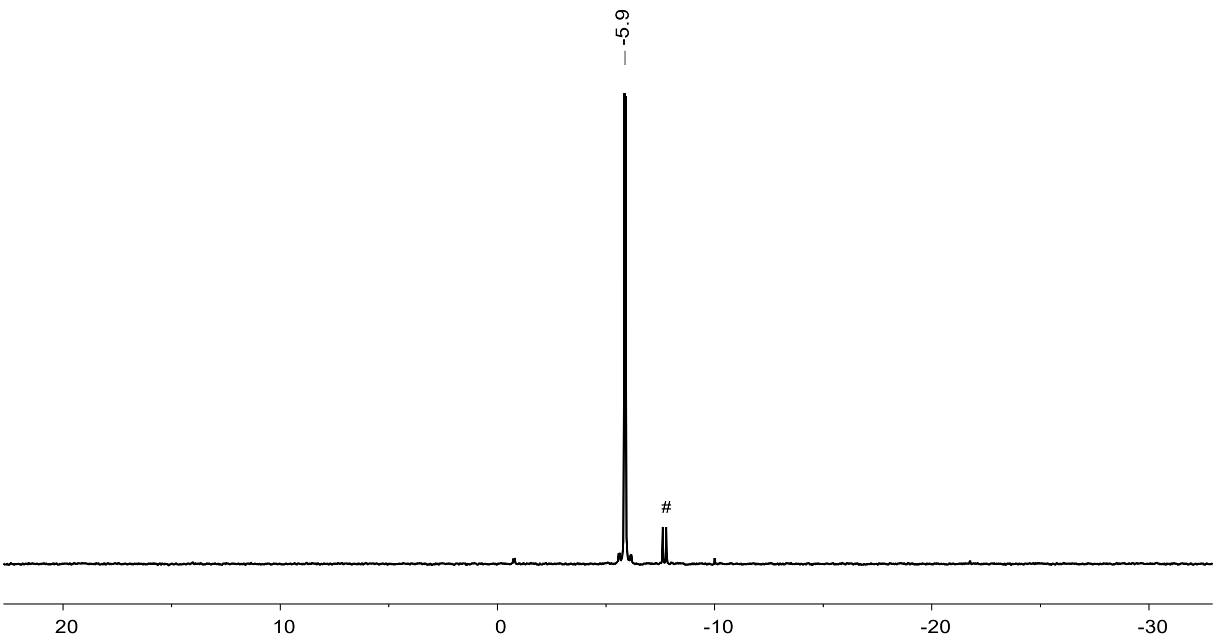

Figure S81. $^{29}Si\{^1H\}$ INEPT NMR spectrum (99.3 MHz, 305 K, chlorobenzene-d_5) of phosphonium triflate **[30a][OTf]** ([#]phospholium triflate **[31a][OTf]**).

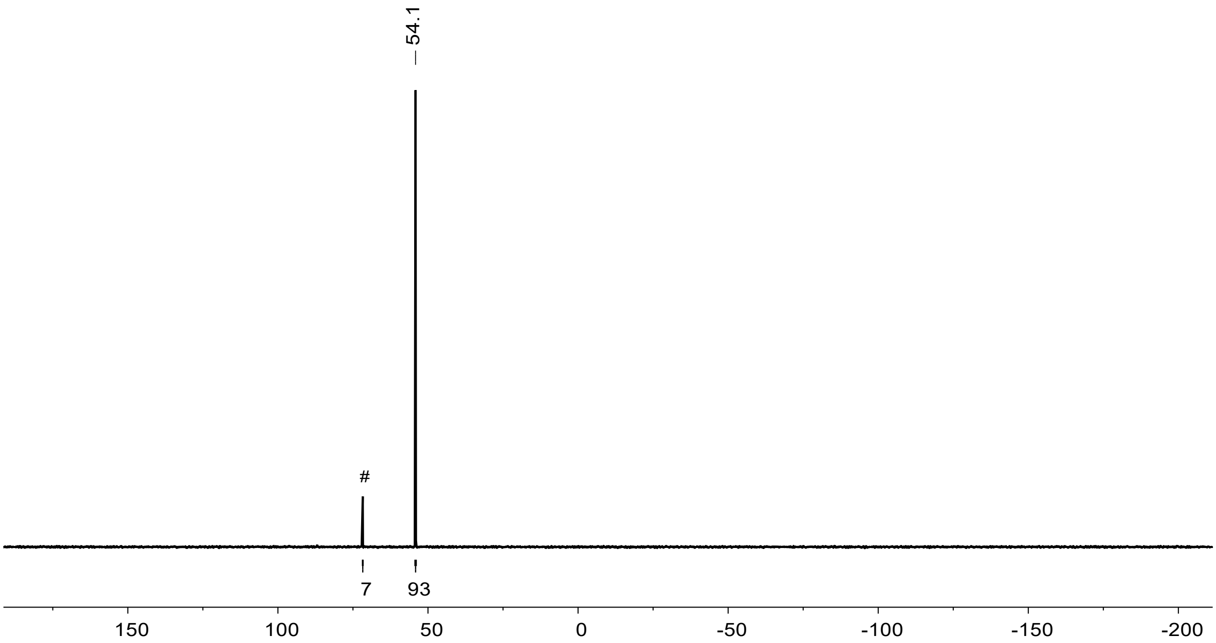

Figure S82. $^{31}P\{^1H\}$ NMR spectrum (202.4 MHz, 305 K, chlorobenzene-d_5) of phosphonium triflate **[30a][OTf]** ([#]phospholium triflate **[31a][OTf]**).

Methylphospholium triflate [31a][OTf]

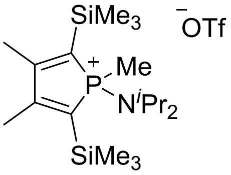

^{1}H NMR (500.1 MHz, 305 K, C_6D_5Cl): δ = 0.22 (s, 18H, 2 x Si<u>Me</u>$_3$), 1.05 (d, $^3J_{H,H}$ = 6.8 Hz, 12H, N(CH<u>Me</u>$_2$)$_2$), 2.03 (d, $^2J_{H,P}$ = 11.1 Hz, 3H, P–<u>Me</u>), 2.04 (d, $^4J_{H,P}$ = 2.5 Hz, 6H, 2 x $C^{2/3}$-Me), 3.41 (sept, $^3J_{H,H}$ = 6.8 Hz, 2H, N(C<u>H</u>Me$_2$)$_2$).

$^{13}C\{^1H\}$ NMR (125.7 MHz, 305 K, C_6D_5Cl): δ = 0.1 (d, $^3J_{C,P}$ = 2 Hz, Si<u>Me</u>$_3$), 7.8 (d, $^1J_{C,P}$ = 63 Hz, P-Me), 18.8 (d, $^3J_{C,P}$ = 25 Hz, $C^{2/3}$–<u>Me</u>), 23.9 (d, $^3J_{C,P}$ = 3 Hz, N(CH<u>Me$_2$</u>)$_2$), 50.4 (d, $^2J_{C,P}$ = 4 Hz, N(<u>C</u>HMe$_2$)$_2$), 122.2 (q, $^1J_{C,F}$ = 322 Hz, <u>C</u>F$_3$), 128.0 (d, $^1J_{C,P}$ = 47 Hz, $C^{1/4}$), 169.7 (d, $^2J_{C,P}$ = 22 Hz, $C^{2/3}$).
$^{29}Si\{^1H\}$ INEPT NMR (99.3 MHz, 305 K, C_6D_5Cl): δ = -7.7 (d, $^2J_{Si,P}$ = 16 Hz).
$^{31}P\{^1H\}$ NMR (202.4 MHz, 305 K, C_6D_5Cl): δ = 71.7.

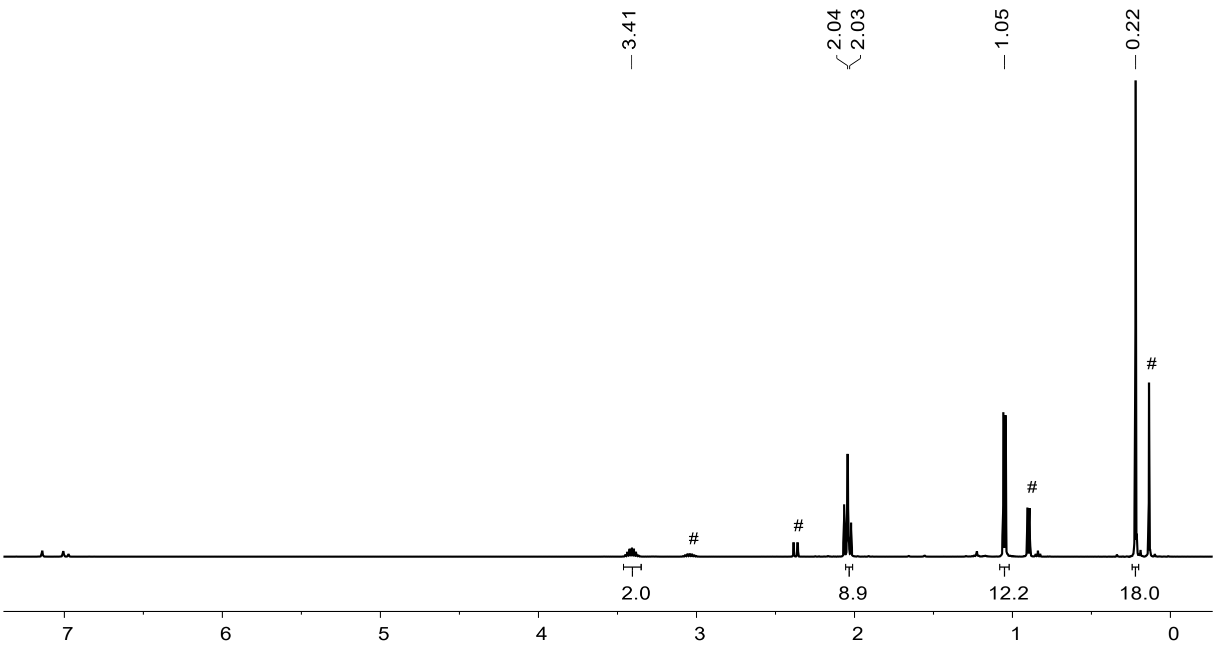

Figure S83. ^1H NMR spectrum (500.1 MHz, 305 K, chlorobenzene-d$_5$) of phospholium triflate **[31a][OTf]** (#phosphonium triflate **[30a][OTf]**).

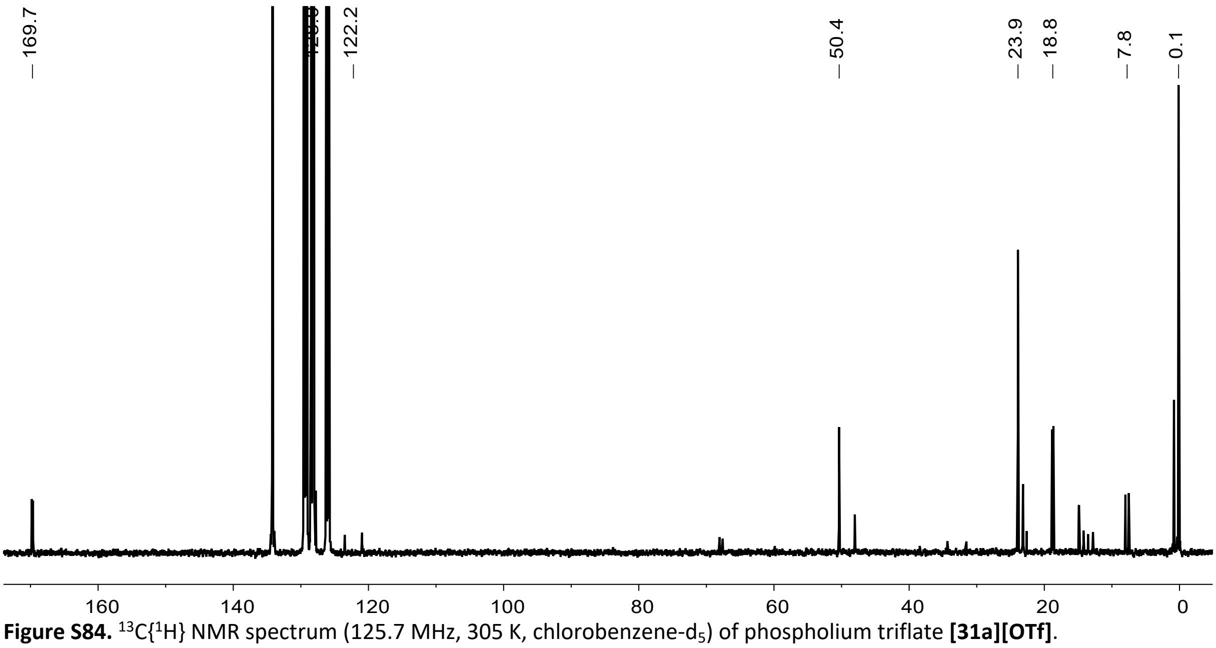

Figure S84. $^{13}C\{^1H\}$ NMR spectrum (125.7 MHz, 305 K, chlorobenzene-d$_5$) of phospholium triflate **[31a][OTf]**.

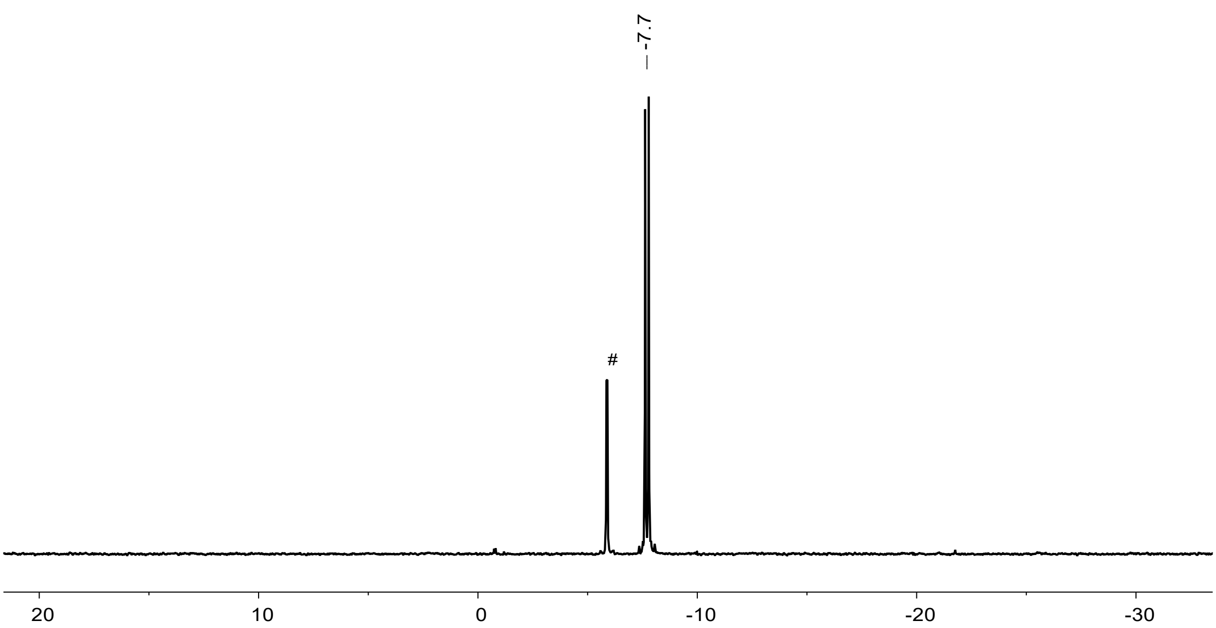

Figure S85. $^{29}Si\{^1H\}$ INEPT NMR spectrum (99.3 MHz, 305 K, chlorobenzene-d_5) of phospholium triflate **[31a][OTf]** ($^{\#}$phosphonium triflate **[30a][OTf]**).

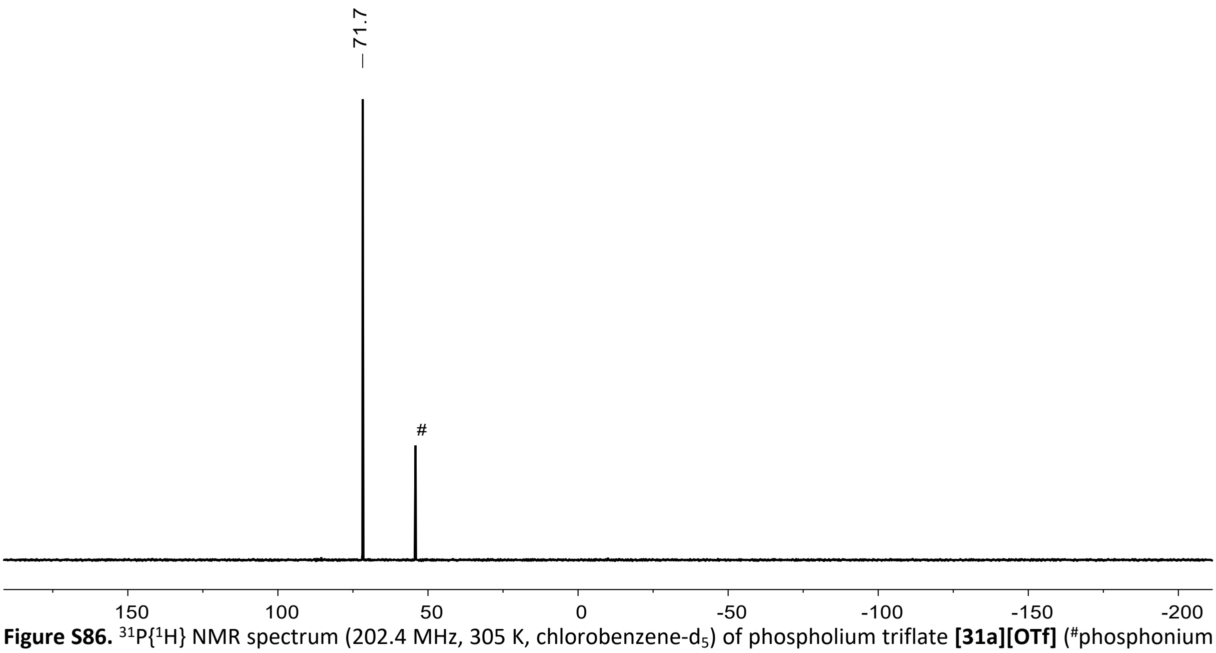

Figure S86. $^{31}P\{^1H\}$ NMR spectrum (202.4 MHz, 305 K, chlorobenzene-d_5) of phospholium triflate **[31a][OTf]** ($^{\#}$phosphonium triflate **[30a][OTf]**).

Methylphosphonium-BCH-germylene [30b][OTf]

To a toluene solution of germylene **15b** (2 mL, 0.20 mmol), pentane (3 mL) was added. The solution was cooled to -30°C and methyltriflate (33 mg, 0.20 mmol) was added. The solution turned yellow immediately. Over the period of 30 minutes the solution was allowed to warm to room temperature. During that time a colourless precipitate had formed. The solvent was removed via teflon tube. The yellow residue was washed with pentane (2 x 2 mL) before it was dried under vacuum to give methylphosphonium-BCH-germylene triflate **[30b][OTf]** (83 mg, 0.12 mmol, 60%) as a colourless solid. The recorded NMR spectra show that the product contained 2% of methylphospholium triflate **[31b][OTf]** which was presumably formed from the small amount of phosphole included in the starting material.

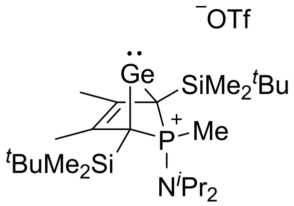

^{1}H NMR (500.1 MHz, 305 K, C_6D_5Cl): δ = 0.14 (s, 6H, Si<u>Me$_2$</u>^{t}Bu), 0.15 (s, 6H, Si<u>Me$_2$</u>^{t}Bu), 0.82 (s, 18H, SiMe$_2$<u>^{t}Bu</u>), 1.03 (d, $^3J_{H,H}$ = 6.9 Hz, 12H, N(CH<u>Me$_2$</u>)$_2$), 2.16 (s, 6H, 2 x C$^{2/3}$-<u>Me</u>), 2.30 (d, $^2J_{H,P}$ = 11.6 Hz, 3H, P-<u>Me</u>), 3.32 (d, sept, $^3J_{H,P}$ = 11.6 Hz, $^3J_{H,H}$ = 6.9 Hz ,2H, N(C<u>H</u>Me$_2$)$_2$).

^{13}C{^{1}H} NMR (125.7 MHz, 305 K, C_6D_5Cl): δ = -2.9 (d, $^3J_{C,P}$ = 3 Hz, Si<u>Me$_2$</u>^{t}Bu), -2.2 (Si<u>Me$_2$</u>^{t}Bu), 13.3 (d, $^1J_{C,P}$ = 98 Hz, P-<u>Me</u>), 16.5 (d, $^3J_{C,P}$ = 6 Hz, C$^{2/3}$-<u>Me</u>), 18.8 (d, $^3J_{C,P}$ = 6 Hz, SiMe$_2$<u>^{t}Bu</u>, C^4), 23.9 (d, $^3J_{C,P}$ = 3 Hz, N(CH<u>Me$_2$</u>)$_2$), 27.5 (SiMe$_2$<u>^{t}Bu</u>), 50.0 (N(<u>C</u>HMe$_2$)$_2$), 68.4 (d, $^1J_{C,P}$ = 51 Hz, <u>C</u>$^{1/4}$), 122.3 (q, $^1J_{C,F}$ = 322 Hz, <u>C</u>F$_3$), 128.0 (d, $^2J_{C,P}$ = 12 Hz, <u>C</u>$^{2/3}$).

^{29}Si{^{1}H} INEPT NMR (99.3 MHz, 305 K, C_6D_5Cl): δ = 0.5 (d, $^2J_{Si,P}$ = 2 Hz).

^{31}P NMR (202.4 MHz, 305 K, C_6D_5Cl): δ = 57.5 (sext, $^2J_{P,H}$ (P-C<u>H$_3$</u>) = $^3J_{P,H}$ (N(C<u>H</u>Me$_2$)$_2$) = 12 Hz).

MS (EI): m/z (%) = 73 (100), 183 (26), 242 (46), 297 (20), 354 (29), 410 (18), 454 (24), 528 (1) [M$^+$].

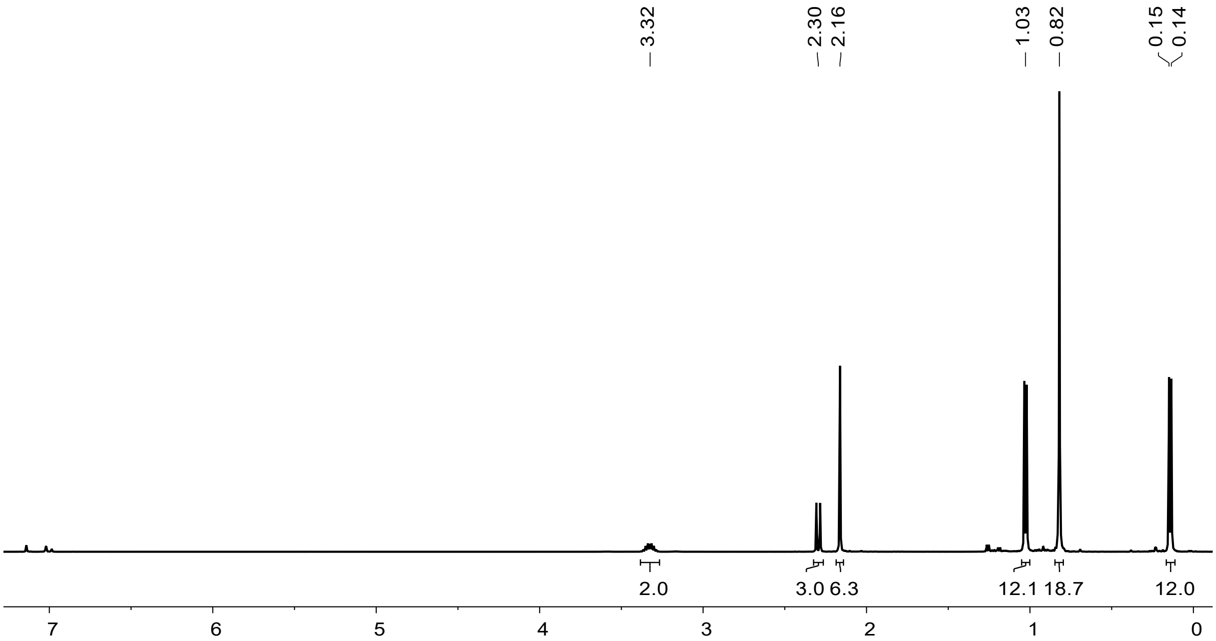

Figure S87. ^{1}H NMR spectrum (500.1 MHz, 305 K, chlorobenzene-d$_5$) of phosphonium triflate **[30b][OTf]**.

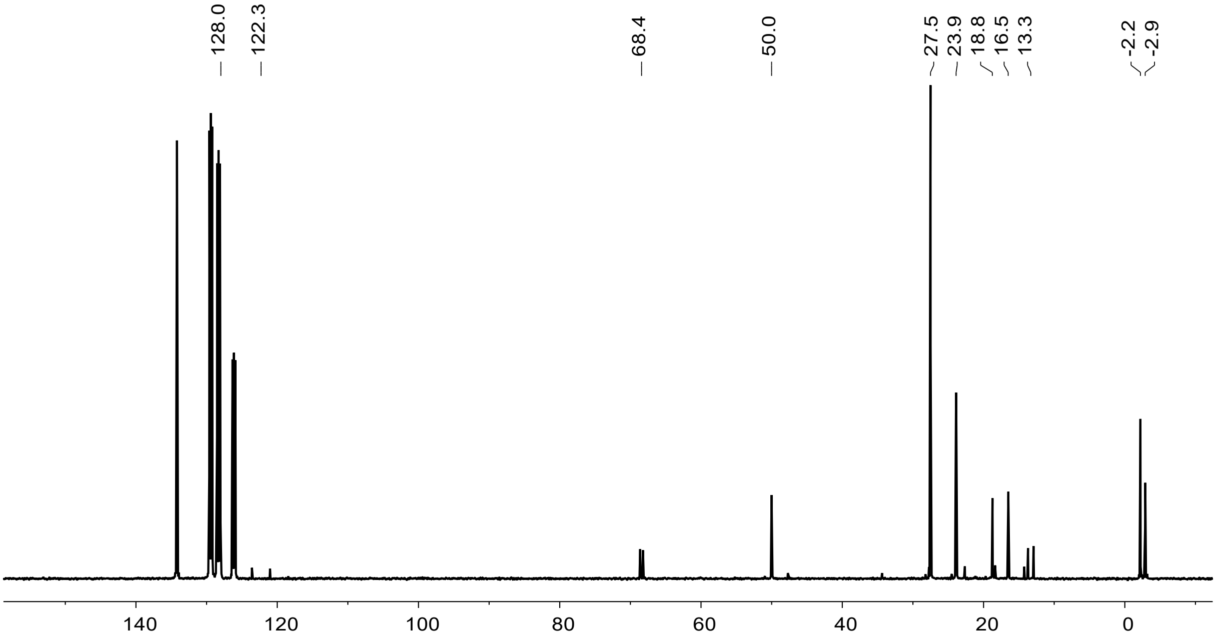

Figure S88. $^{13}C\{^{1}H\}$ NMR spectrum (125.7 MHz, 305 K, chlorobenzene-d_5) of phosphonium triflate **[30b][OTf]**.

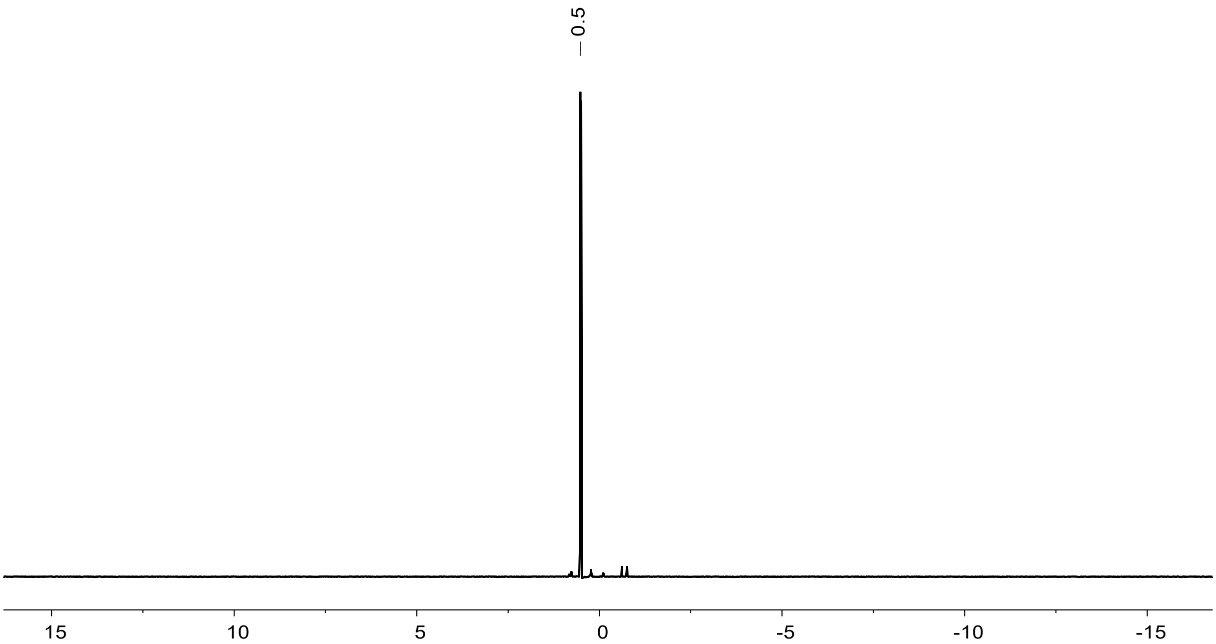

Figure S89. $^{29}Si\{^{1}H\}$ INEPT NMR spectrum (99.3 MHz, 305 K, chlorobenzene-d_5) of phosphonium triflate **[30b][OTf]**.

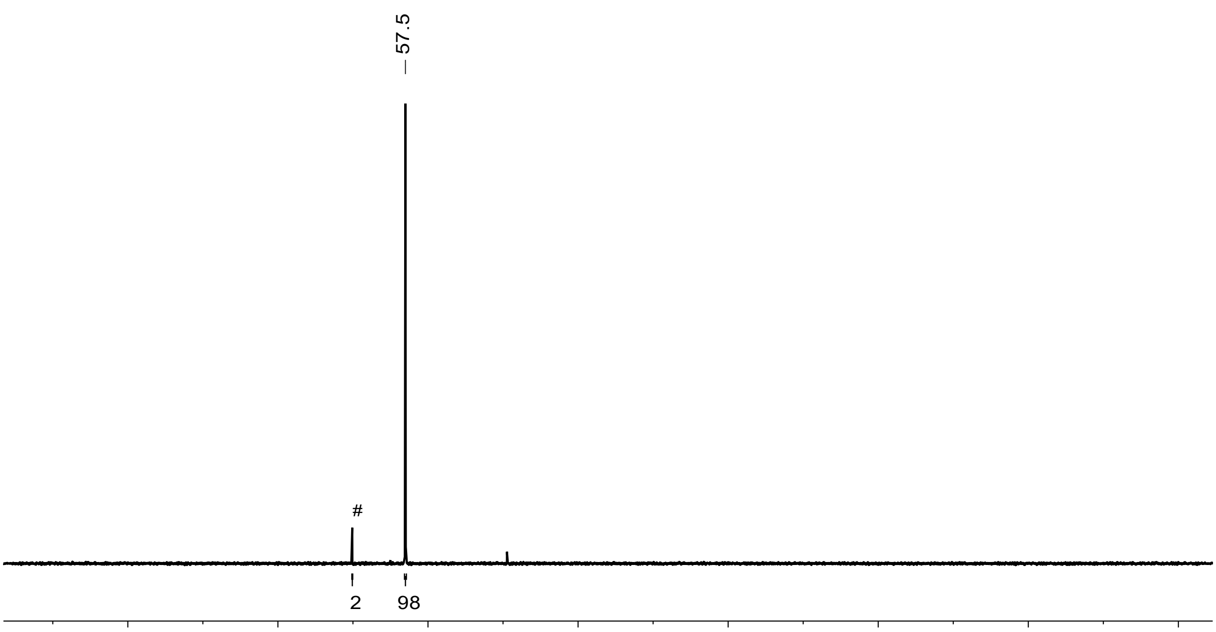

Figure S90. $^{31}P\{^1H\}$ NMR spectrum (202.4 MHz, 305 K, chlorobenzene-d$_5$) of phosphonium triflate **[30b][OTf]** ($^{\#}$methylphospholium triflate **[31b][OTf]**).

Methylphospholium triflate [31b][OTf]

Methylphospholium triflate **[31b][OTf]** was obtained upon reaction of methylphosphonium triflate **[30b][OTf]** with Pd(CH_2SiMe_3)$_2$(COD), carried out to access the ligating properties of the germylene moiety of **[30b]**$^+$. The reaction led to the elimination of germanium and decomposition of the palladium complex.

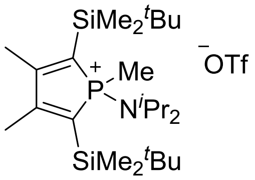

^{1}H NMR (500.1 MHz, 305 K, C_6D_5Cl): δ = 0.23 (s, 6H, Si<u>Me</u>$_2$^{t}Bu), 0.23 (s, 6H, Si<u>Me</u>$_2$^{t}Bu), 0.82 (s, 18H, SiMe$_2$^{t}<u>Bu</u>), 1.19 (d, $^3J_{H,H}$ = 6.8 Hz, 12H, N(CH<u>Me</u>$_2$)$_2$), 2.10 (d, $^2J_{P,H}$ = 11.5 Hz, 3H, P-<u>Me</u>), 2.15 (d, $^4J_{P,H}$ = 2.4 Hz, 6H, C$^{2/3}$-<u>Me</u>), 3.57 (dsept, $^3J_{P,H}$ = 12 Hz, $^3J_{H,H}$ = 6.8 Hz, 2H, N(C<u>H</u>Me$_2$)$_2$).

$^{13}C\{^1H\}$ NMR (125.7 MHz, 305 K, C_6D_5Cl): δ = -3.2 (Si<u>Me</u>$_2$^{t}Bu), -3.1 (d, $^3J_{C,P}$ = 4 Hz, Si<u>Me</u>$_2$^{t}Bu), 8.5 (d, $^1J_{C,P}$ = 62 Hz, P-<u>Me</u>), 21.0 (d, $^3J_{C,P}$ = 25 Hz, C$^{2/3}$-<u>Me</u>), 24.5 (d, $^3J_{C,P}$ = 3 Hz, N(CH<u>Me</u>$_2$)$_2$), 27.7 (SiMe$_2$^{t}<u>Bu</u>), 50.9 (d, $^2J_{C,P}$ = 4 Hz, N(<u>C</u>HMe$_2$)$_2$), 122.2 (q, $^1J_{C,F}$ = 322 Hz, <u>C</u>F$_3$), 127.1 (d, $^1J_{C,P}$ = 44 Hz, <u>C</u>$^{1/4}$), 171.4 (d, $^2J_{C,P}$ = 21 Hz, <u>C</u>$^{2/3}$).

$^{29}Si\{^1H\}$ INEPT NMR (99.3 MHz, 305 K, C_6D_5Cl): δ = -0.7 (d, $^2J_{Si,P}$ = 14 Hz).

^{31}P NMR (202.4 MHz, 305 K, C_6D_5Cl): δ = 75.2 (sextd, $^2J_{P,H}$ (P-C<u>H</u>$_3$) = $^3J_{P,H}$ (N(C<u>H</u>Me$_2$)$_2$) = 12 Hz, $^4J_{P,H}$ (P-C$^{2/3}$-<u>Me</u>) = 2 Hz).

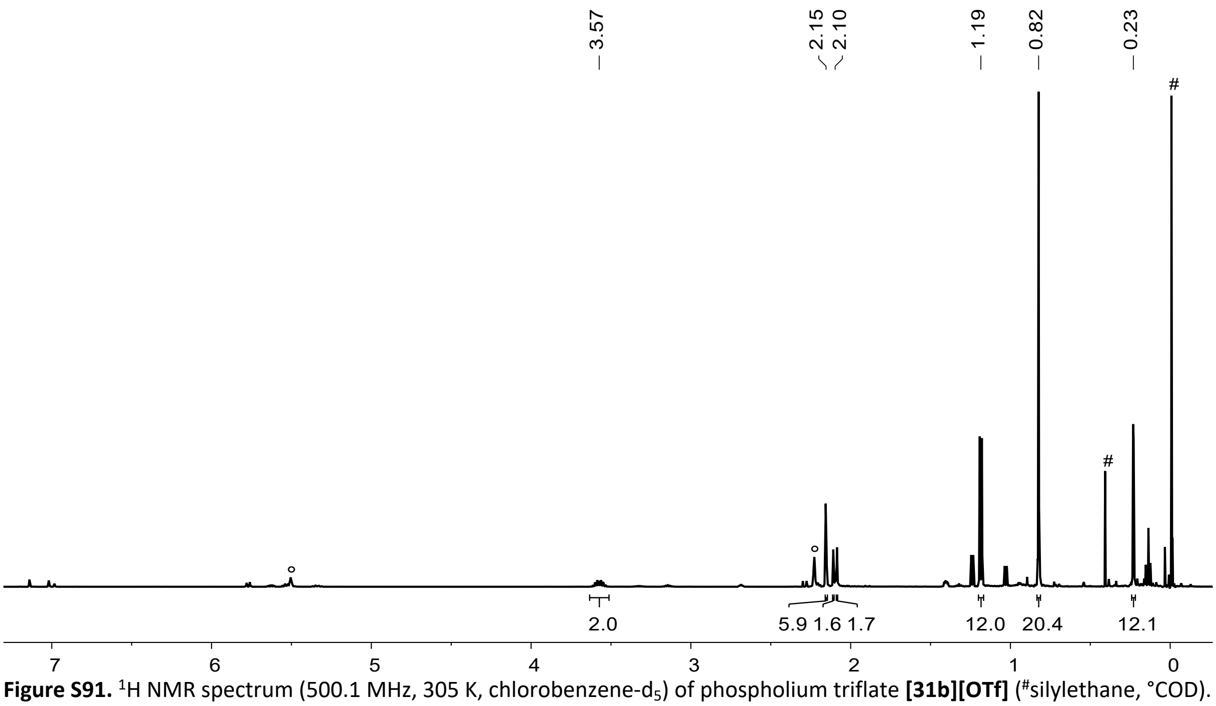

Figure S91. ^{1}H NMR spectrum (500.1 MHz, 305 K, chlorobenzene-d$_5$) of phospholium triflate **[31b][OTf]** ($^\#$silylethane, °COD).

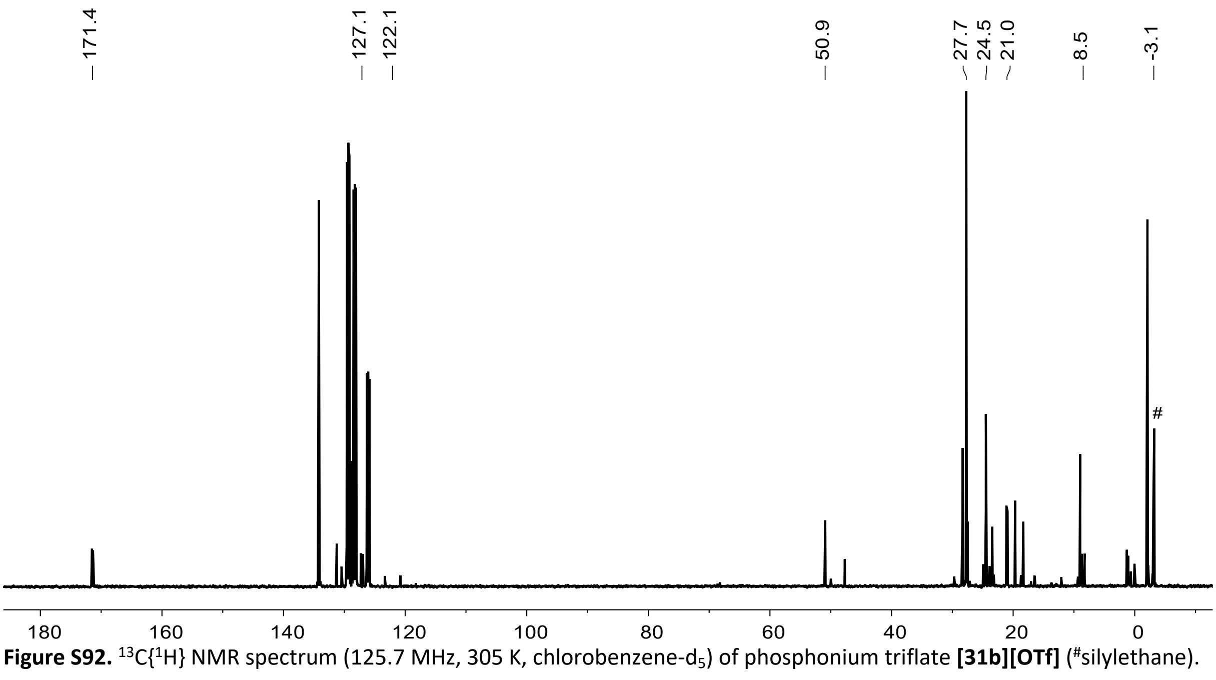

Figure S92. ^{13}C{^{1}H} NMR spectrum (125.7 MHz, 305 K, chlorobenzene-d$_5$) of phosphonium triflate **[31b][OTf]** ($^\#$silylethane).

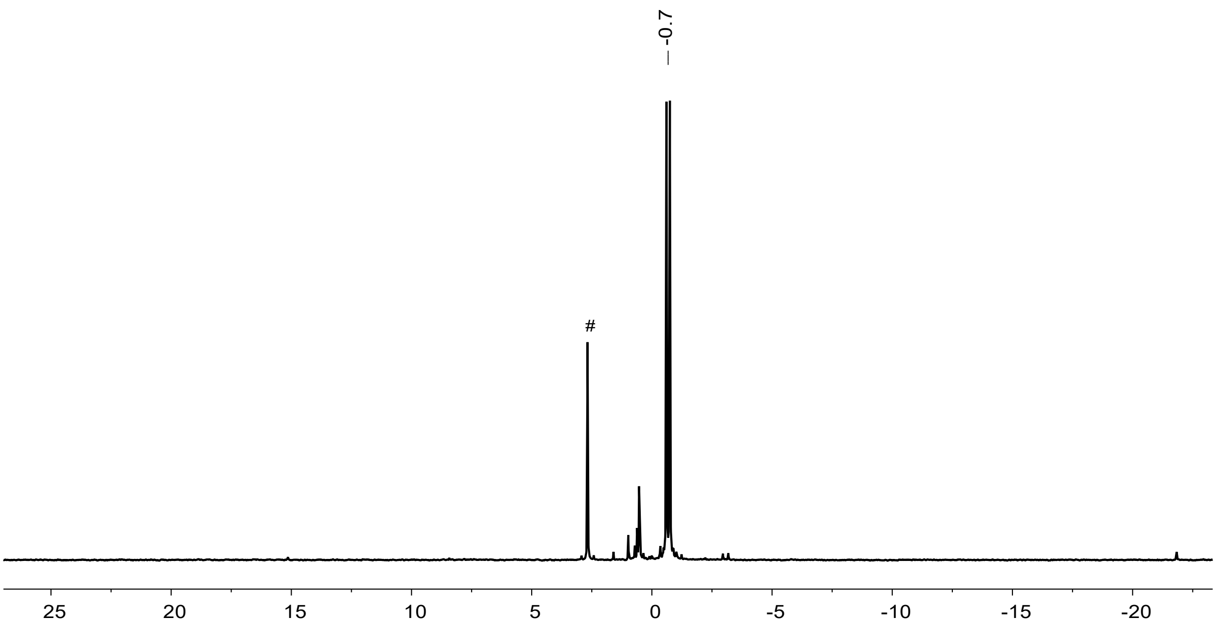

Figure S93. $^{29}Si\{^1H\}$ INEPT NMR spectrum (99.3 MHz, 305 K, chlorobenzene-d_5) of phosphonium triflate **[31b][OTf]** ($^{\#}$silylethane).

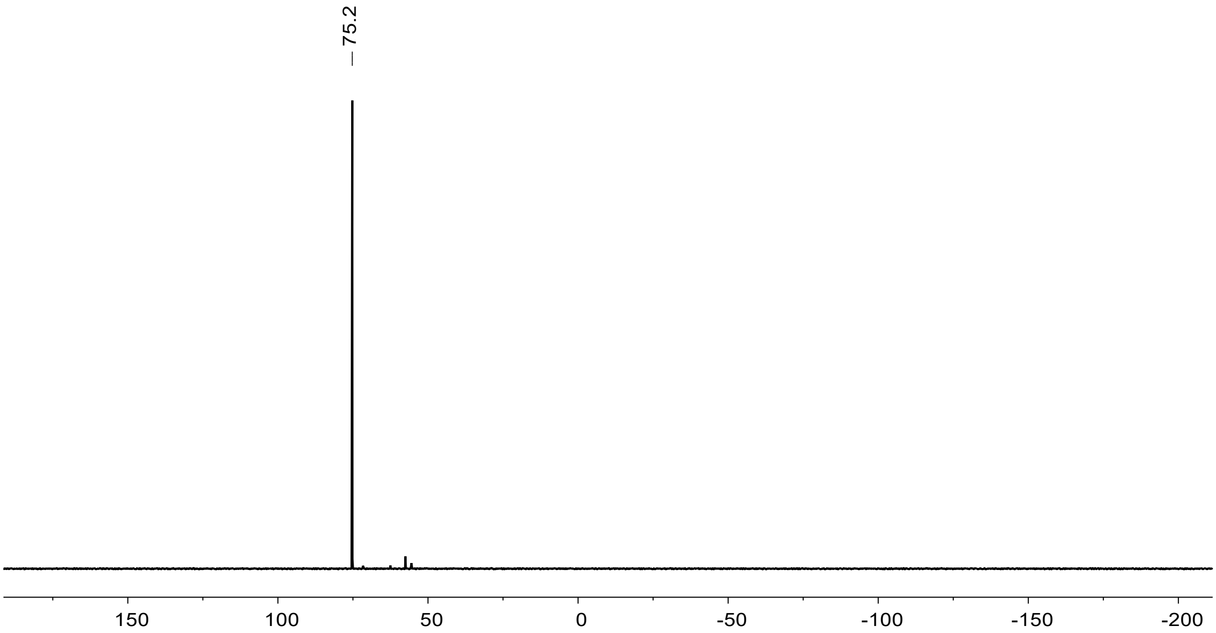

Figure S94. $^{31}P\{^1H\}$ NMR spectrum (202.4 MHz, 305 K, chlorobenzene-d_5) of phosphonium triflate **[31b][OTf]**.

Rhodium complexes 32b & 33b

Upon addition of a toluene solution of germylene **15b** (0.1 M, 1 mL) to a toluene solution of $[Rh(COD)Cl]_2$ (0.05 mmol, 25 mg) at room temperature, the mixture turned green within five minutes. After additional five minutes of stirring, the solution turned dark and a black precipitate was formed. The elimination of germanium and rhodium metal, as indicated by the formation of the black precipitate, was confirmed by analysis of NMR spectra, recorded from the crude reaction mixture: almost pure phosphole **20b** was obtained. To monitor the reaction and detect the intermediate complex, the reaction was carried out at -60°C. As no visible reaction took place at that temperature, the reaction mixture was stirred for 5 minutes at room temperature before a NMR sample was taken

and measured at -50°C immediately. Spectra at -10°C and at room temperature were measured afterwards with 1 hour in between each temperature variation. The sample was kept at -60°C in the meantime. The two rhodium complexes **32b** and **33b** were identified using 2D NMR spectra.

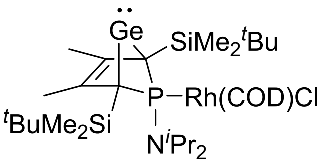

32b

^{1}H NMR (from ^{1}H^{13}C HMBC NMR: 500.1 MHz, 263 K, toluene-d$_8$): δ = 0.27 (Si<u>Me</u>$_2$^{t}Bu), 1.99 (C$^{2/3}$-<u>Me</u>).
^{13}C NMR (from ^{1}H^{13}C HMBC NMR: 500.1 MHz, 263 K, toluene-d$_8$): δ = 60.1 (C$^{1/4}$), 129.0 (C$^{2/3}$).
^{31}P{^{1}H} NMR (202.4 MHz, 263 K, toluene-d$_8$): δ = 120.1 (d, $^1J_{P,Rh}$ = 163 Hz).

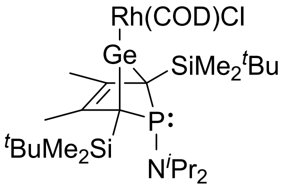

33b

^{1}H NMR (from ^{1}H^{13}C HMBC NMR: 500.1 MHz, 263 K, toluene-d$_8$): δ = 0.43 (Si<u>Me</u>$_2$^{t}Bu), 1.92 (C$^{2/3}$-<u>Me</u>).
^{13}C NMR (from ^{1}H^{13}C HMBC NMR: 500.1 MHz, 263 K, toluene-d$_8$): δ = 57.3 (C$^{1/4}$), 125.3 (C$^{2/3}$).
^{31}P{^{1}H} NMR (202.4 MHz, 263 K, toluene-d$_8$): δ = 45.6.

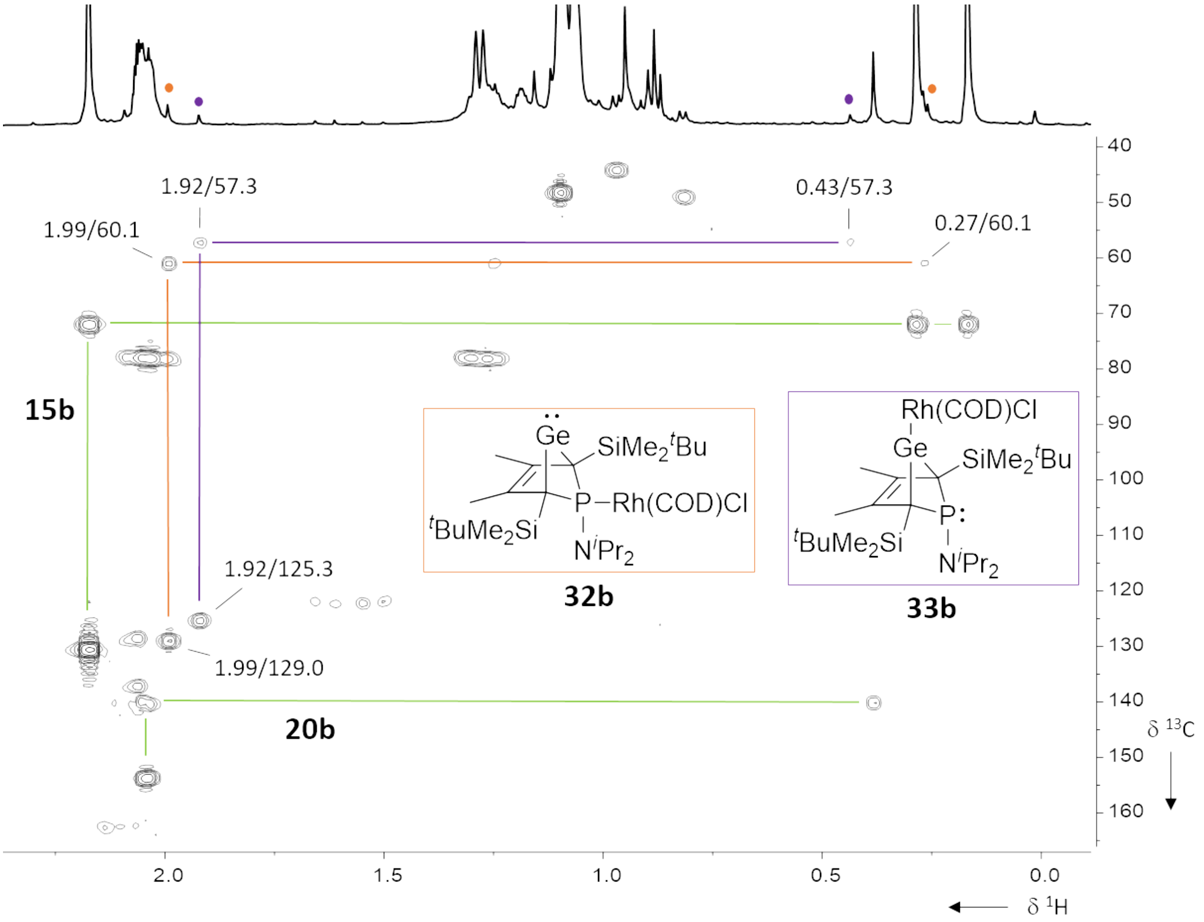

Figure S95. ^{1}H^{13}C HMBC NMR spectrum (500.1 MHz, 263 K, toluene-d$_8$) of the reaction mixture.

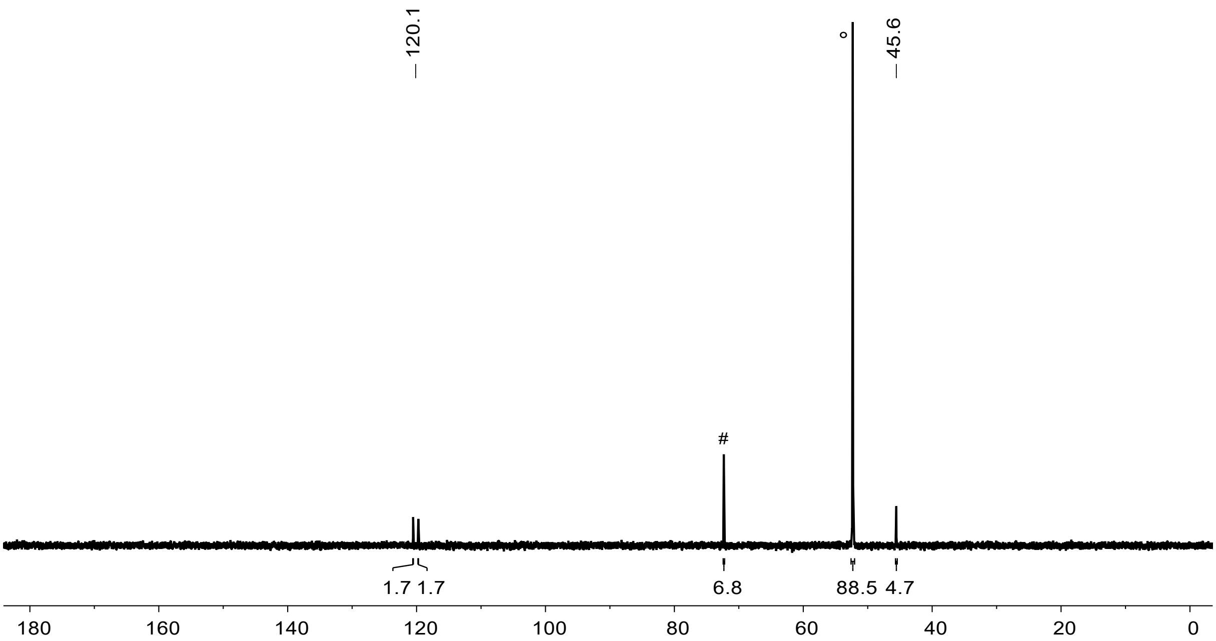

Figure S96. $^{31}P\{^1H\}$ NMR spectrum (202.4 MHz, 263 K, toluene-d_8) of the reaction mixture (#phosphole **20b**, °germylene **15b**).

Phosphole oxide 34a

Germylene **15a** (0.20 mmol) was freshly prepared. After 15 minutes of stirring, the cold bath was removed and the stopper of the Schlenk flask was exchanged for a tube filled with calciumdichloride as drying agent. The argon transfer was stopped and the solution was stirred overnight. A pale yellow precipitate was formed. The solvent was removed, the residue redissolved in pentane and filtered over a PTFE syringe filter. Removal of the solvent gave phosphole oxide **34a** (60 mg, 0.16 mmol, 80%) as oily yellow residue.

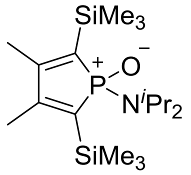

^{1}H NMR (500.1 MHz, 305 K, C_6D_5Cl): δ = 0.36 (s, 18H, 2 x Si$\underline{Me}_3$), 1.23 (d, $^3J_{H,H}$ = 6.9 Hz, 12H, N(CH$\underline{Me}_2$)$_2$), 1.70 (d, $^4J_{H,P}$ = 2.4 Hz, 6H, 2 x $C^{2/3}$–$\underline{Me}$), 3.76 (sept, $^3J_{H,H}$ = 6.9 Hz, 2H, N(C$\underline{H}$Me$_2$)$_2$).

$^{13}C\{^1H\}$ NMR (125.7 MHz, 305 K, C_6D_5Cl): δ = 0.5 (d, $^3J_{C,P}$ = 2 Hz, Si$\underline{Me}_3$), 17.6 (d, $^3J_{C,P}$ = 26 Hz, $C^{2/3}$–$\underline{Me}$), 24.7 (N(CH$\underline{Me}_2$)$_2$), 45.8 (d, $^2J_{C,P}$ = 5 Hz, N($\underline{C}$HMe$_2$)$_2$), 135.5 (d, $^1J_{C,P}$ = 71 Hz, $C^{1/4}$), 160.0 (d, $^2J_{C,P}$ = 26 Hz, $C^{2/3}$).

$^{29}Si\{^1H\}$ INEPT NMR (99.3 MHz, 305 K, C_6D_5Cl): δ = -9.9 (d, $^2J_{Si,P}$ = 16 Hz).

$^{31}P\{^1H\}$ NMR (202.4 MHz, 305 K, C_6D_5Cl): δ = 63.0.

MS (EI): m/z (%) = 91 (100), 356 (32), 371 (3) [M^+].

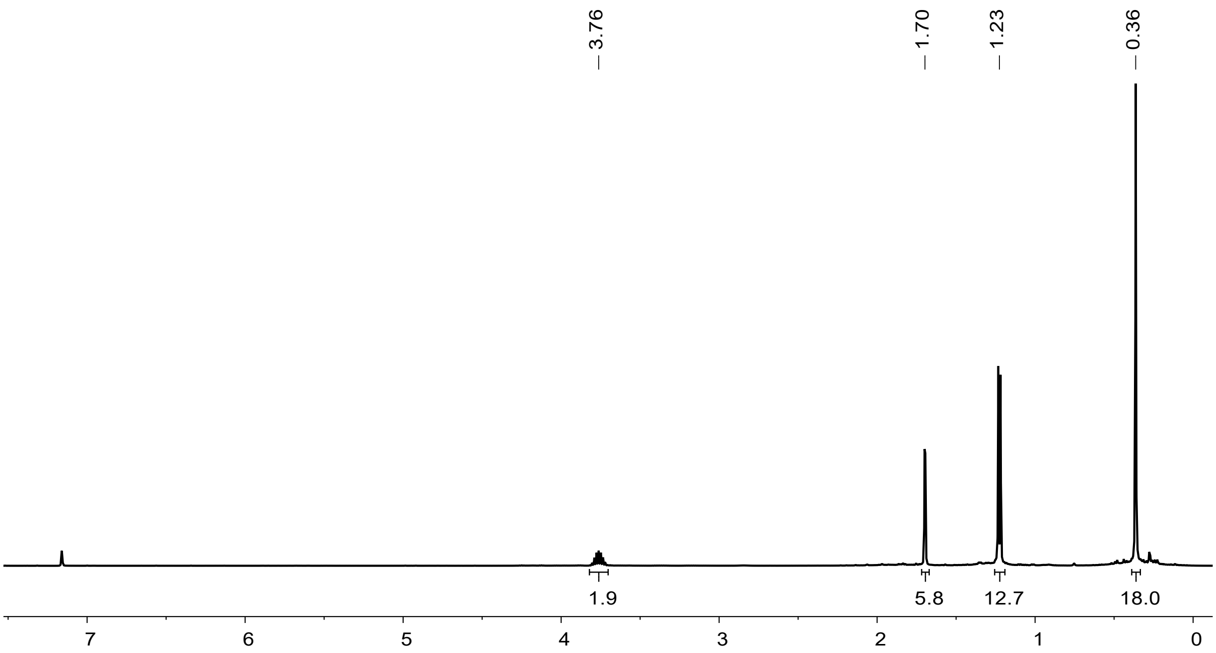

Figure S97. ^{1}H NMR spectrum (500.1 MHz, 305 K, benzene-d_6) of phosphole oxide **34a**.

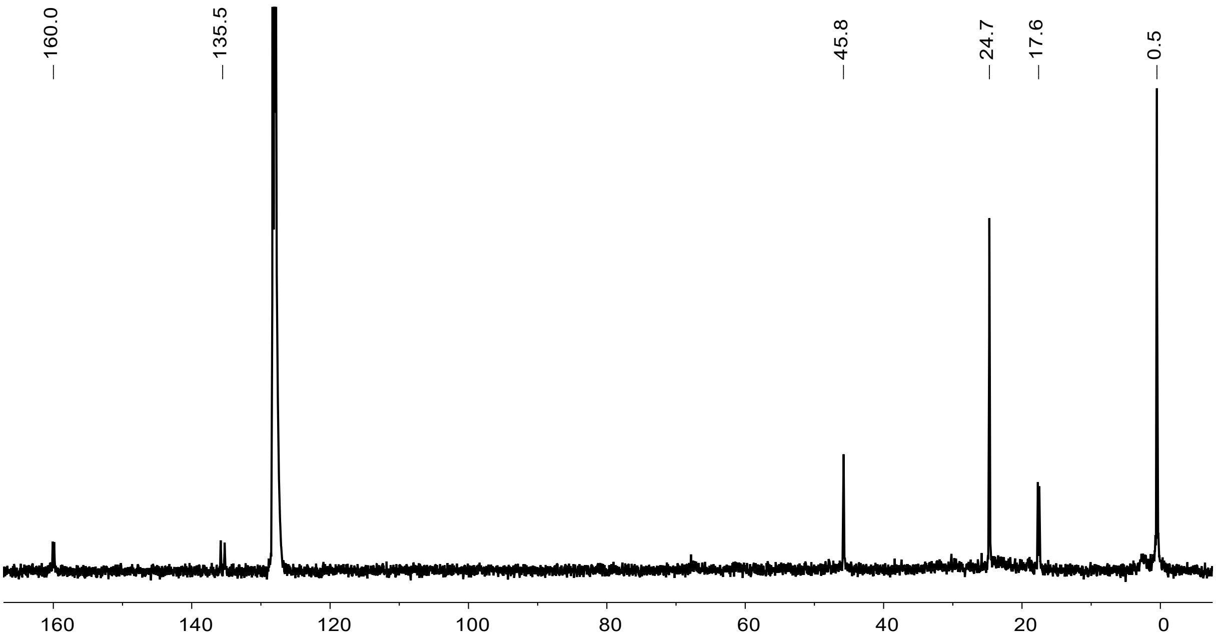

Figure S98. $^{13}C\{^{1}H\}$ NMR spectrum (125.7 MHz, 305 K, benzene-d_6) of phosphole oxide **34a**.

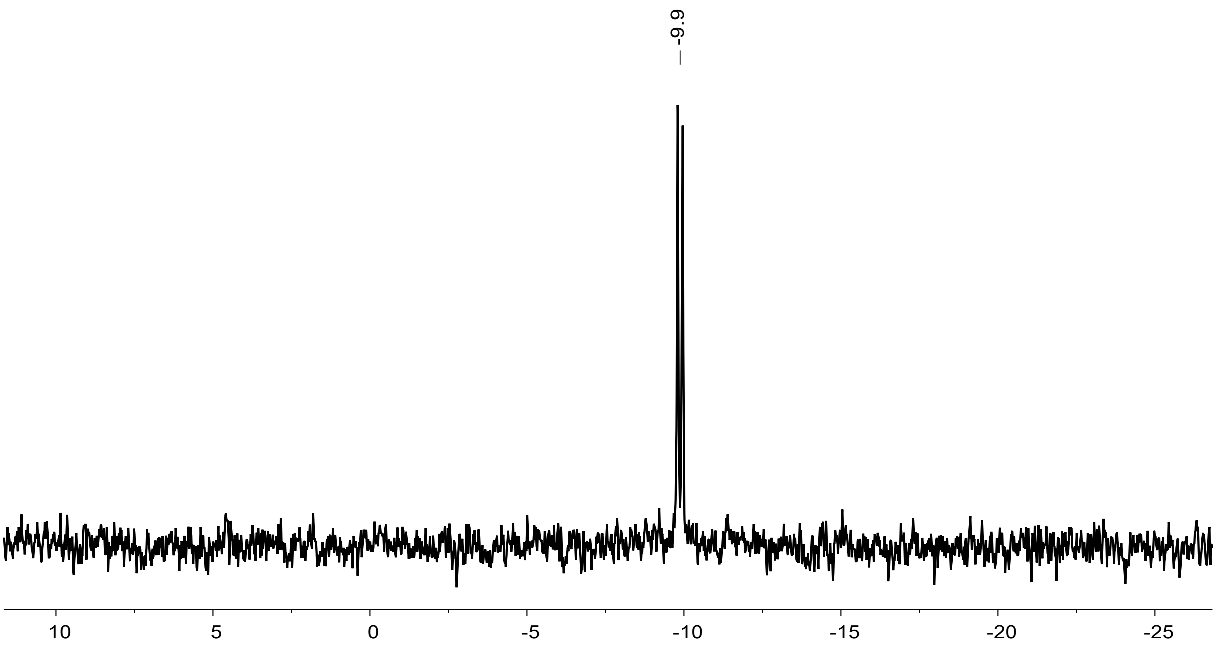

Figure S99. $^{29}Si\{^1H\}$ INEPT NMR spectrum (99.3 MHz, 305 K, benzene-d_6) of phosphole oxide **34a**.

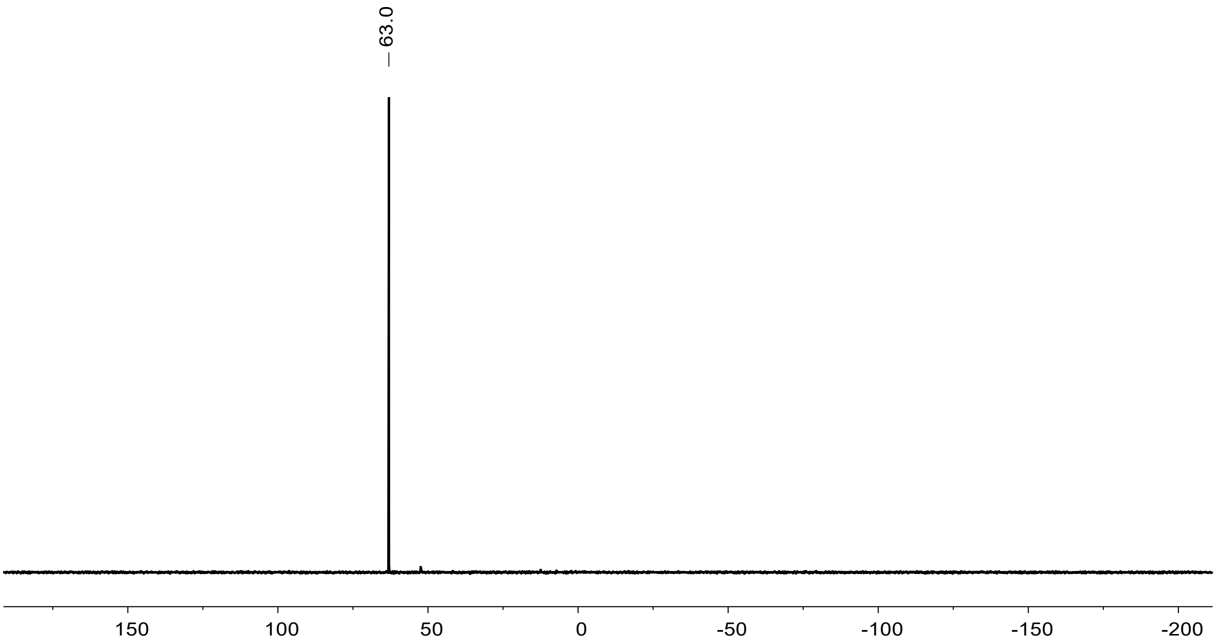

Figure S100. $^{31}P\{^1H\}$ NMR spectrum (202.4 MHz, 305 K, benzene-d_6) of phosphole oxide **34a**.

Phosphole sulfide 35a

Germylene **15a** (0.20 mmol) was freshly prepared and the mixture was stirred for 30 minutes before the solvent was removed. The residue was dissolved in toluene and filtered into a vial, equipped with cyclooctasulfur (13 mg, 0.05 mmol). Stirring overnight afforded a yellow solution with a colourless precipitate. Solid and solution were separated by centrifugation. The solvent was removed from the solution to give phosphole sulfide **35a** (68 mg, 0.18 mmol, 90%) as yellow solid.

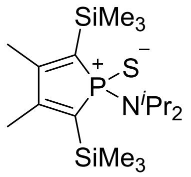

^{1}H NMR (500.1 MHz, 305 K, C_6D_5Cl): δ = 0.43 (s, 18H, 2 x Si<u>Me</u>$_3$), 1.18 (d, $^3J_{H,H}$ = 7.0 Hz, 12H, N(CH<u>Me</u>$_2$)$_2$), 1.69 (d, $^4J_{H,P}$ = 2.4 Hz, 6H, 2 x C$^{2/3}$–<u>Me</u>), 4.19 (sept, $^3J_{H,H}$ = 7.0 Hz, 2H, N(C<u>H</u>Me$_2$)$_2$).
^{13}C{^{1}H} NMR (125.7 MHz, 305 K, C_6D_5Cl): δ = 0.9 (d, $^3J_{C,P}$ = 2 Hz, Si<u>Me</u>$_3$), 17.4 (d, $^3J_{C,P}$ = 25 Hz, C$^{2/3}$–<u>Me</u>), 25.1 (d, $^3J_{C,P}$ = 3 Hz, N(CH<u>Me</u>$_2$)$_2$), 47.4 (d, $^2J_{C,P}$ = 7 Hz, N(<u>C</u>HMe$_2$)$_2$), 138.9 (d, $^1J_{C,P}$ = 52 Hz, C$^{1/4}$), 156.8 (d, $^2J_{C,P}$ = 22 Hz, C$^{2/3}$).
^{29}Si{^{1}H} INEPT NMR (99.3 MHz, 305 K, C_6D_5Cl): δ = -9.3 (d, $^2J_{Si,P}$ = 18 Hz).
^{31}P{^{1}H} NMR (202.4 MHz, 305 K, C_6D_5Cl): δ = 92.0.
MS (EI): m/z (%) = 73 (42), 100 (100), 200 (62), 271 (20), 288 (36), 387 (5) [M$^+$].

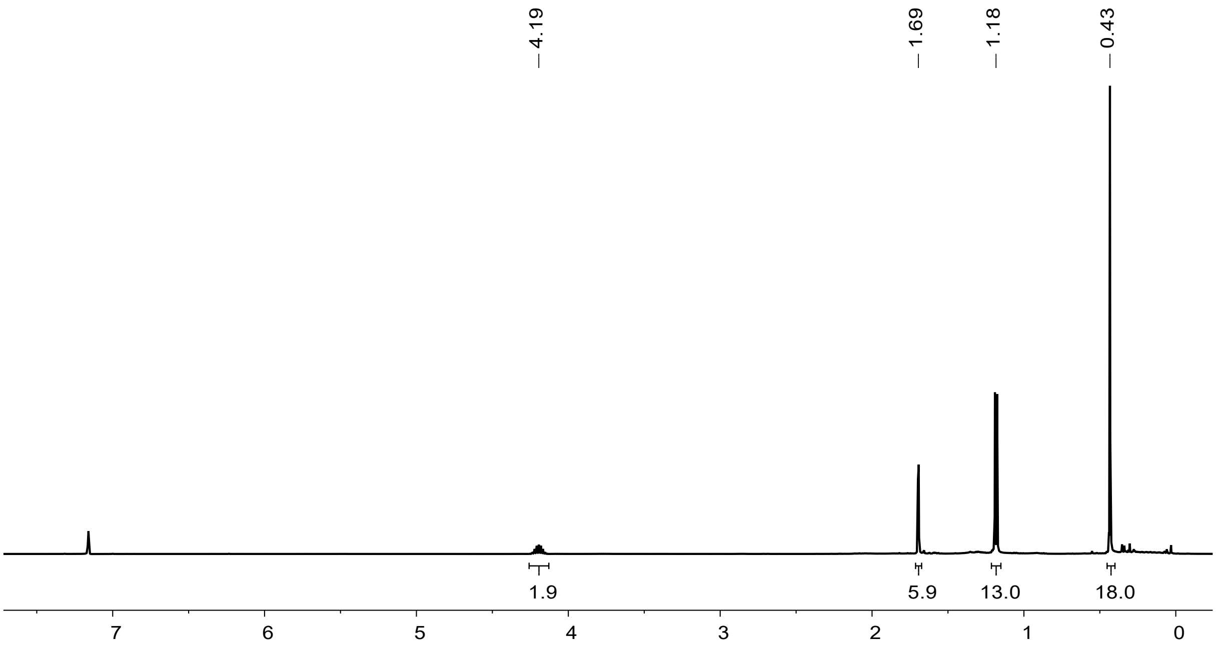

Figure S101. ^{1}H NMR spectrum (500.1 MHz, 305 K, benzene-d_6) of phosphole sulfide **35a**.

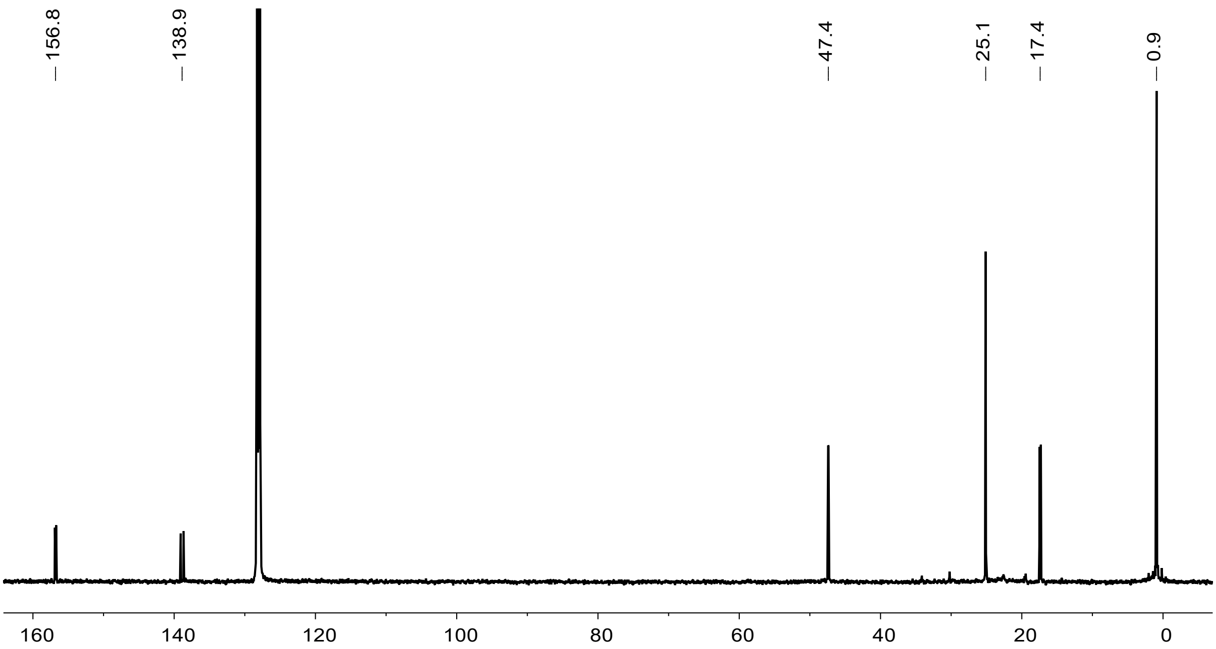

Figure S102. $^{13}C\{^{1}H\}$ NMR spectrum (125.7 MHz, 305 K, benzene-d_6) of phosphole sulfide **35a**.

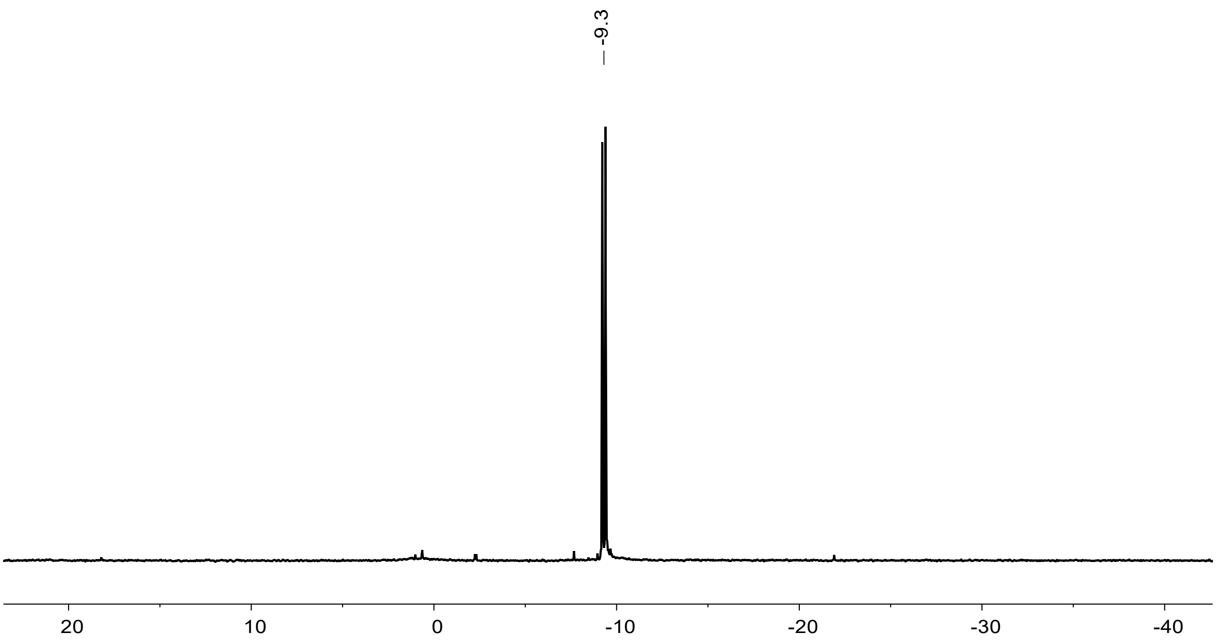

Figure S103. $^{29}Si\{^{1}H\}$ INEPT NMR spectrum (99.3 MHz, 305 K, benzene-d_6) of phosphole sulfide **35a**.

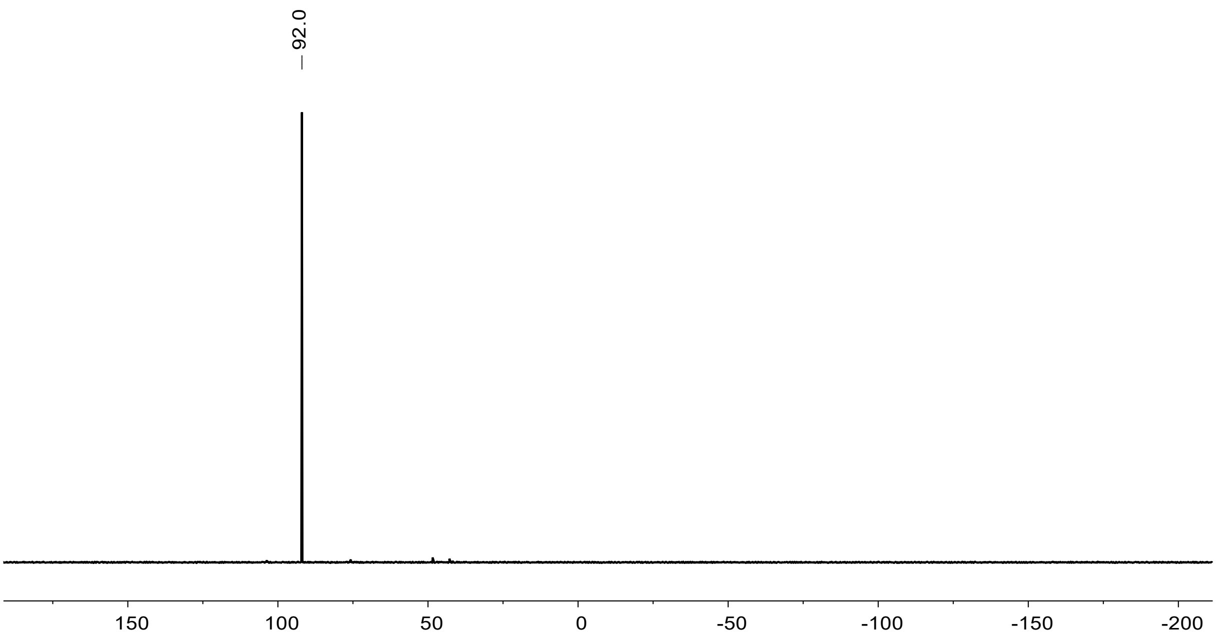

Figure S104. $^{31}P\{^1H\}$ NMR spectrum (202.4 MHz, 305 K, benzene-d_6) of phosphole sulfide **35a**.

Phosphole Sulfide 36b

To a toluene/pentane solution of germylene **16b** (215 mg, 0.36 mmol), cyclooctasulfur (12 mg, 0.045 mmol) was added at room temperature. It was stirred for 20 minutes. After that time, a precipitate was formed which was identified as germaniumsulfide after qualitative identification of sulfide ions. Monitoring the reaction revealed that it was not complete, however, phospha-BCH-germylene sulfide **38b** was identified in the mixture (Figure S109-S112). An excess amount of cyclooctasulfur was added and the mixture was stirred for 5 days. The precipitates were filtered off and the solvent was removed. Phosphole sulfide **36b** (145 mg, 0.26 mmol, 72%) was isolated as a yellow solid.

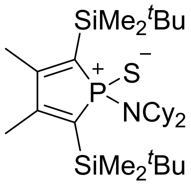

^{1}H NMR (500.1 MHz, 305 K, C_6D_6): δ = 0.28 (s, 6H, Si<u>Me</u>$_2{}^t$Bu), 0.60 (s, 6H, Si<u>Me</u>$_2{}^t$Bu), 0.94-1.04 (m, 5H, Cy), 1.08 (s, 18H, SiMe$_2{}^t$<u>Bu</u>), 1.17-1.27 (m, 5H, Cy), 1.50-1.60 (m, 6H, Cy), 1.68-1.74 (m, 4H, Cy), 1.88 (d, $^4J_{H,H}$ = 2.3 Hz, 6H, 2 x C<u>H</u>$_3$), 1.91-1.96 (m, 4H, Cy), 3.73-3.85 (m, 2H, Cy, 2 x <u>H</u>C^{ipso}).

$^{13}C\{^1H\}$ NMR (125.7 MHz, 305 K, C_6D_6): δ = -3.0 (Si<u>Me</u>$_2{}^t$Bu), -1.7 (Si<u>Me</u>$_2{}^t$Bu), 19.4 (SiMe$_2$<u>^{t}Bu</u>, C^4), 19.6 (C$^{2/3}$-Me), 26.3 (Cy), 27.9 (Cy), 28.7 (Si<u>Me</u>$_2{}^t$Bu), 36.7 (d, $^3J_{C,P}$ = 3 Hz, Cy), 56.9 (d, $^2J_{C,P}$ = 5 Hz, Cy, C^{ipso}), 137.4 (d, $^1J_{C,P}$ = 49 Hz, C$^{1/4}$), 158.6 (d, $^2J_{C,P}$ = 21 Hz, C$^{2/3}$).

$^{29}Si\{^1H\}$ INEPT NMR (99.3 MHz, 305 K, C_6D_6): δ = -2.1 (d, $^2J_{Si,P}$ = 16 Hz).

$^{31}P\{^1H\}$ NMR (202.4 MHz, 305 K, C_6D_6): δ = 95.8 ($^2J_{P,Si}$ = 16 Hz).

MS (EI): m/z (%) = 73 (15), 180 (100), 315 (28), 551 (36) [M$^+$].

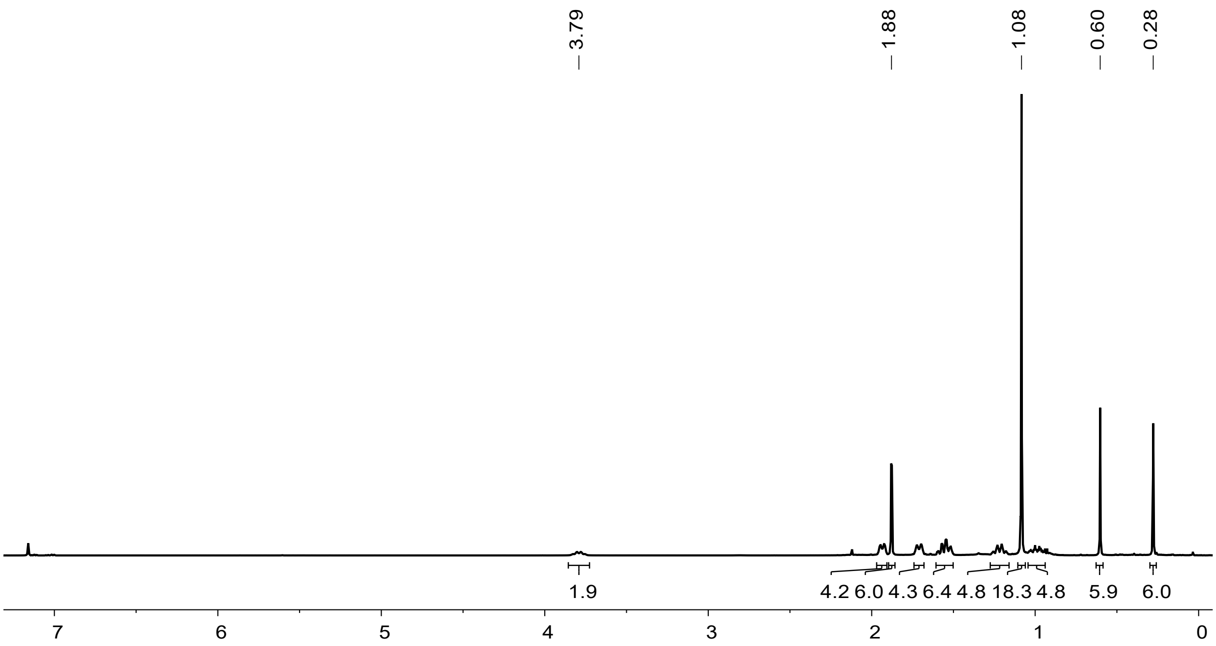

Figure S105. ^{1}H NMR spectrum (500.1 MHz, 305 K, benzene-d$_6$) of phosphole sulfide **36b**.

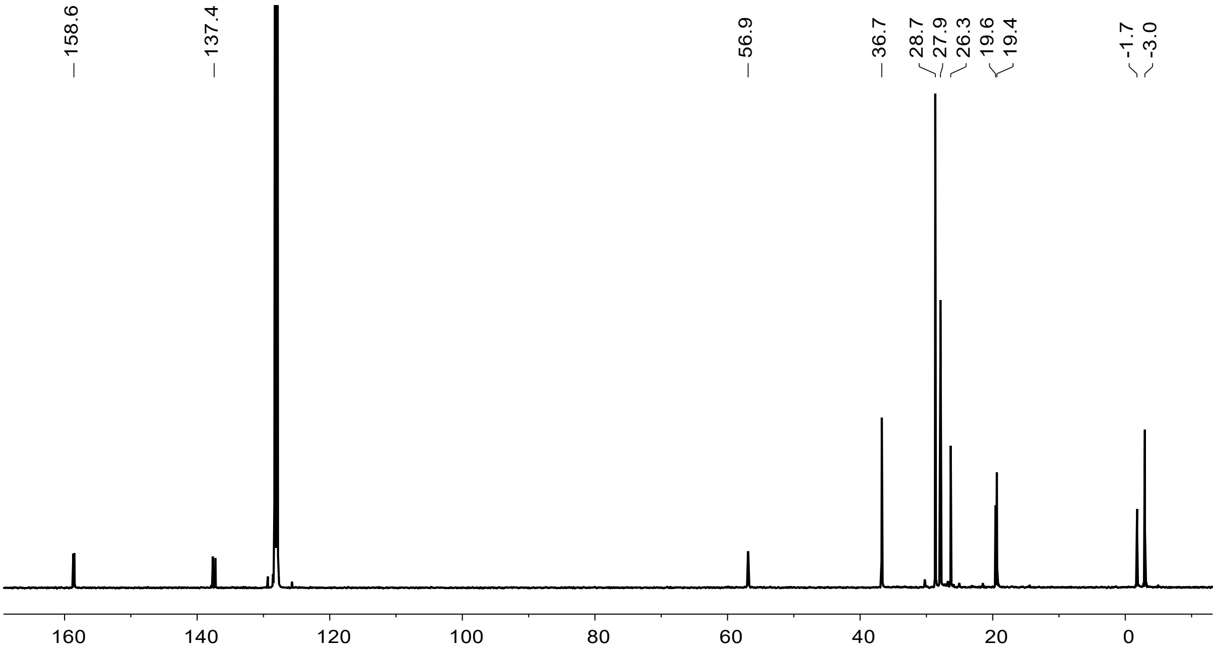

Figure S106. ^{13}C{^{1}H} NMR spectrum (125.7 MHz, 305 K, benzene-d$_6$) of phosphole sulfide **36b**.

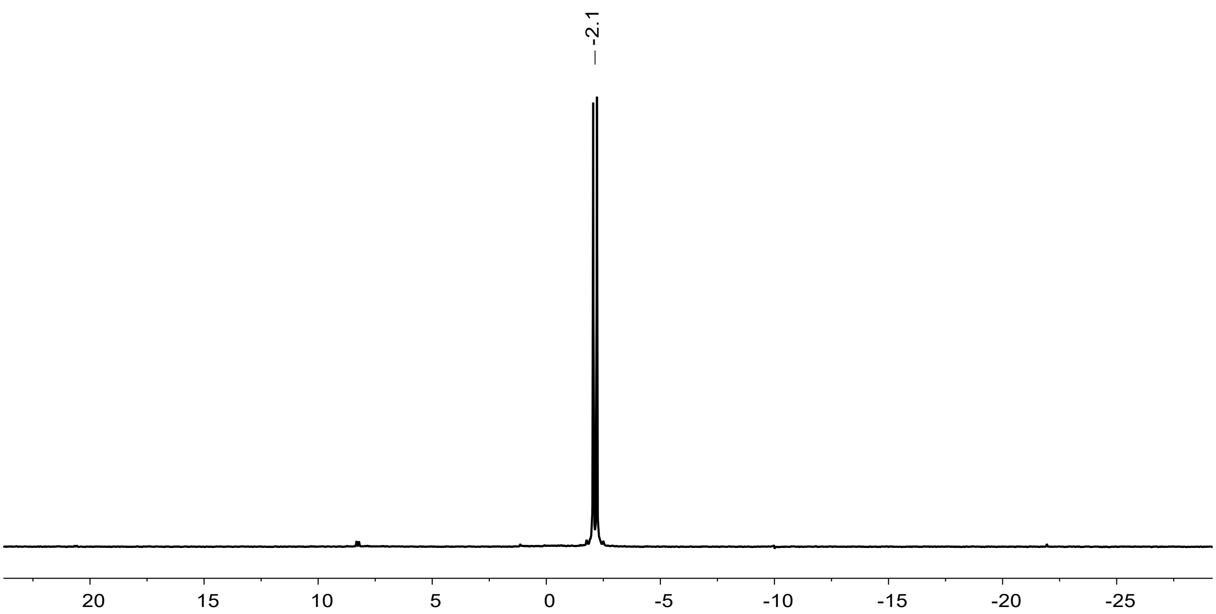

Figure S107. $^{29}Si\{^1H\}$ INEPT NMR spectrum (99.3 MHz, 305 K, benzene-d_6) of phosphole sulfide **36b**.

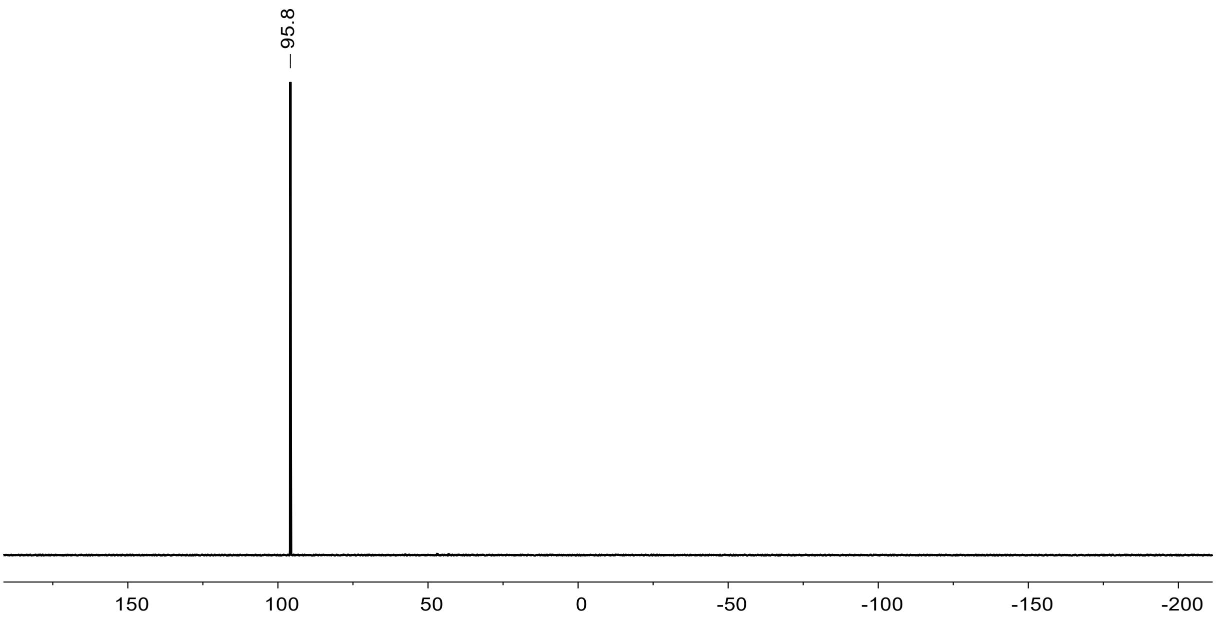

Figure S108. $^{31}P\{^1H\}$ NMR spectrum (202.4 MHz, 305 K, benzene-d_6) of phosphole sulfide **36b**.

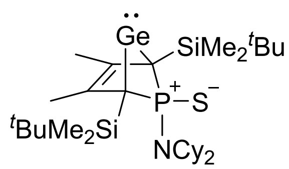

38b

^{1}H NMR (from $^1H^{13}C$ HMBC NMR: 500.1 MHz, 305 K, C_6D_5Cl): δ = 0.22 (Si<u>Me</u>$_2{}^t$Bu), 0.65 (Si<u>Me</u>$_2{}^t$Bu), 2.19 ($C^{2/3}$-<u>Me</u>).

^{13}C NMR (from $^1H^{13}C$ HMBC NMR: 500.1 MHz, 305 K, C_6D_5Cl): δ = 77 ($C^{1/4}$), 129 ($C^{2/3}$).

$^{29}Si\{^1H\}$ INEPT NMR (99.3 MHz, 305 K, C_6D_5Cl): δ = 0.1 (d, $^2J_{Si,P}$ = 3 Hz).

$^{31}P\{^1H\}$ NMR (202.4 MHz, 305 K, C_6D_5Cl): δ = 87.3.

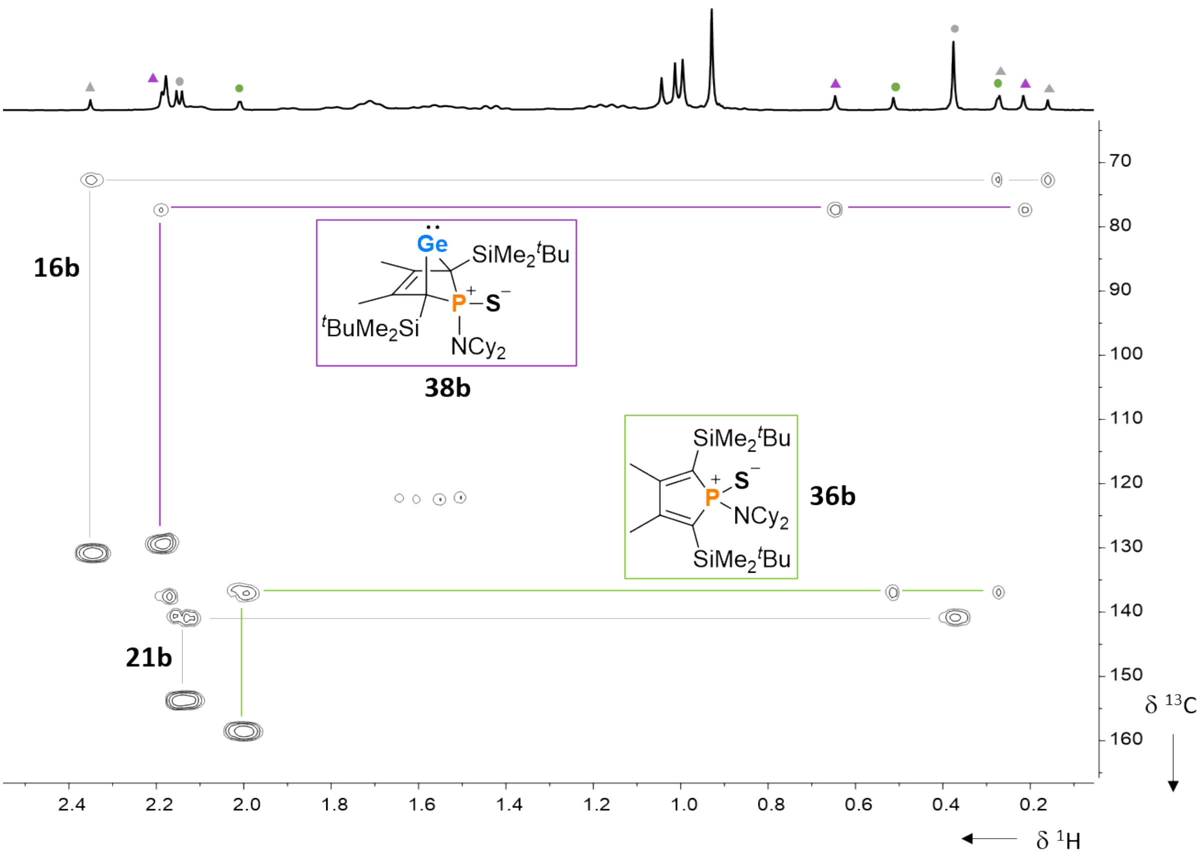

Figure S109. $^{1}H^{13}C$ HMBC NMR spectrum (500.1 MHz, 305 K, chlorobenzene-d_5) of the mixture of germylene **16b**, phosphole **21b**, germylene sulfide **38b** (purple) and phosphole sulfide **36b** (green).

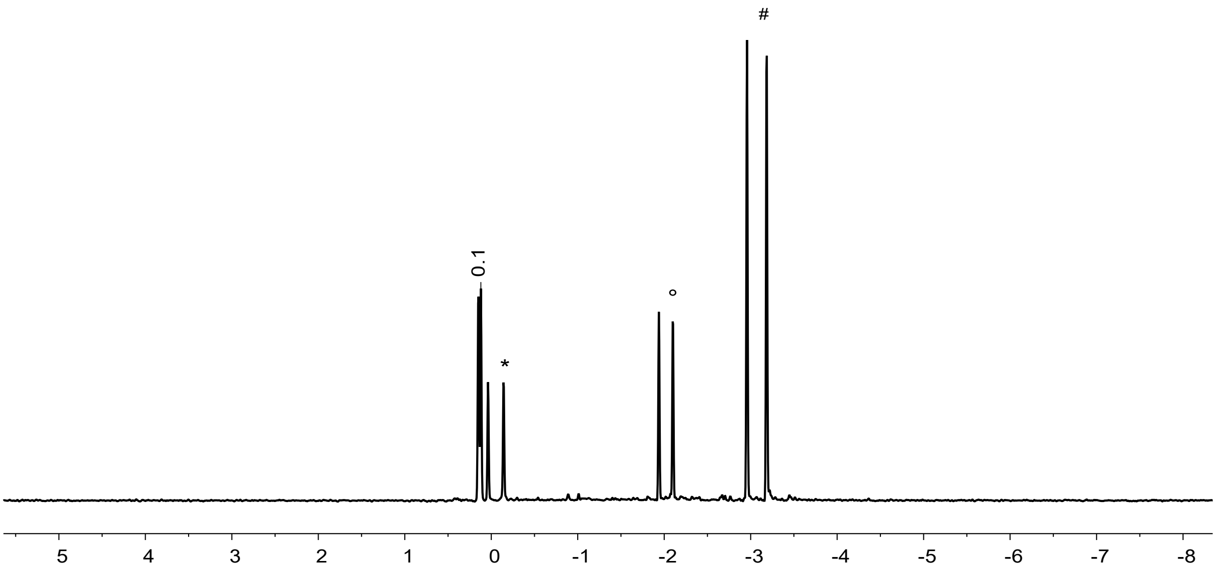

Figure S110. ^{29}Si INEPT NMR spectrum (99.3 MHz, 305 K, chlorobenzene-d_5) of the mixture of germylene **16b** (*), phosphole **21b** (#), germylene sulfide **38b** and phosphole sulfide **36b** (°).

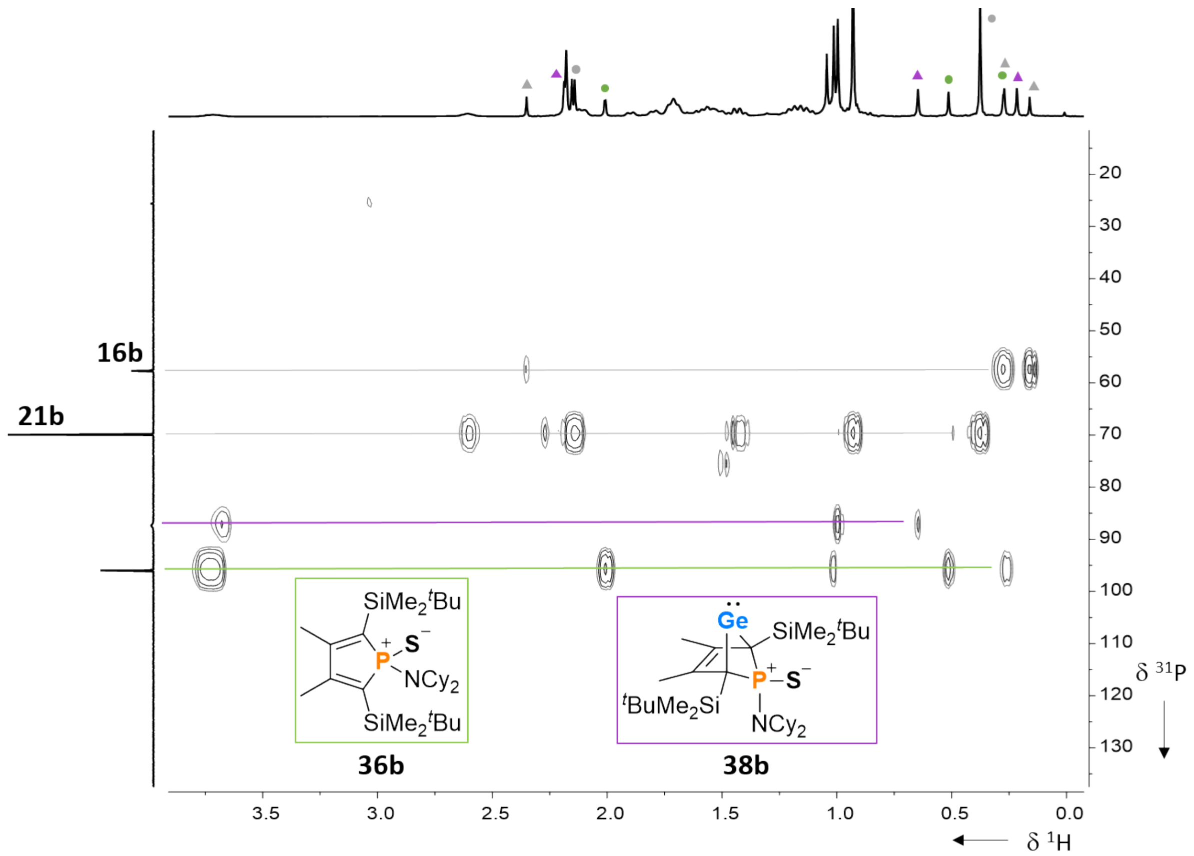

Figure S111. $^{1}H^{31}P$ HMBC NMR spectrum (500.1 MHz, 305 K, chlorobenzene-d_5) of the mixture of germylene **16b**, phosphole **21b**, germylene sulfide **38b** (purple) and phosphole sulfide **36b** (green).

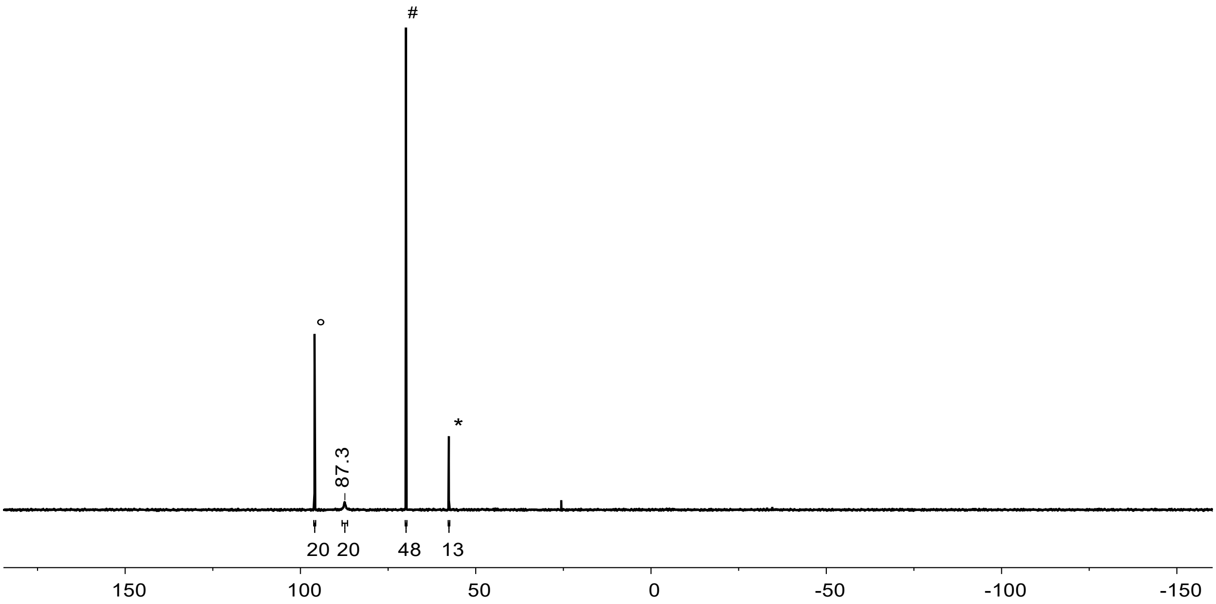

Figure S112. $^{31}P\{^{1}H\}$ NMR spectrum (202.4 MHz, 305 K, chlorobenzene-d_5) of the mixture of germylene **16b** (*), phosphole **21b** (#), germylene sulfide **38b** and phosphole sulfide **36b** (°).

NHC-stabilised germylene 40b

A vial was equipped with tetramethyl NHC (25 mg, 0.20 mmol). A toluene solution of germylene **15b** (0.1 M, 1.0 mL) was added and the mixture was stirred for three days. During this time a red precipitate had formed. The precipitates were filtered off using a PTFE syringe filter. The solvent was removed and NMR spectra were recorded in benzene-d_6. Phosphole **20b** was identified as main product alongside 10% of another compound. Recrystallisation from toluene at -30°C gave yellow crystals of the byproduct, NHC-stabilised germylene **40b**. NMR spectra were recorded of the remaining crystals and residue of the mother liquor, showing a 2:1 mixture of phosphole **20b** and germylene **40b**.

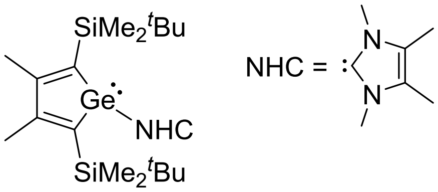

^{1}H NMR (500.1 MHz, 305 K, C_6D_6): δ = -0.01 (s, 6H, SiMe_2^{t}Bu), 0.46 (s, 6H, SiMe_2^{t}Bu), 1.18 (s, 18H, $SiMe_2$^{t}Bu), 1.23 (6H, NHC-Me), 2.51 (s, 6H, $C^{2/3}$-Me), 2.73 (br, 3H, NHC-Me), 3.65 (br, 3H, NHC-Me).
^{13}C NMR (from ^{1}H^{13}C HMBC NMR: 500.1 MHz, 305 K, C_6D_6): δ = 156.6 ($C^{2/3}$), 164.2 ($C^{1/4}$).
^{29}Si{^{1}H} INEPT NMR (99.3 MHz, 305 K, C_6D_6): δ = -3.0.

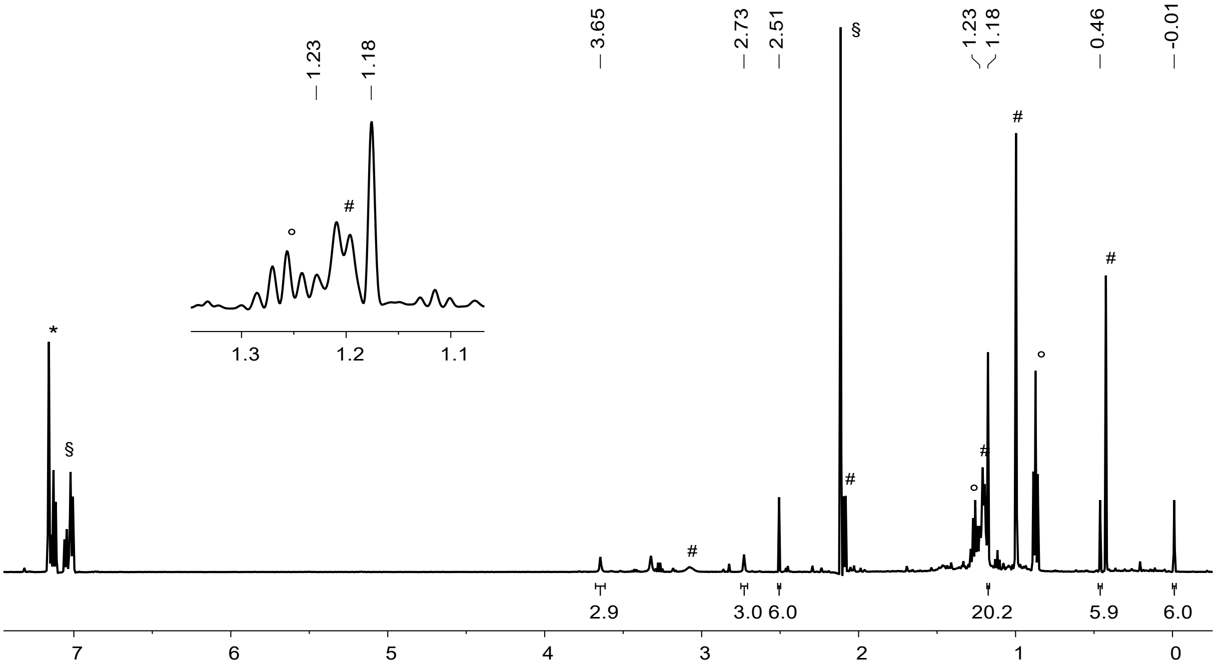

Figure S113. ^{1}H NMR spectrum (500.1 MHz, 305 K, benzene-d_6) of the obtained mixture of NHC-stabilised germylene **40b** and phosphole **20b** (#) (°pentane, § toluene).

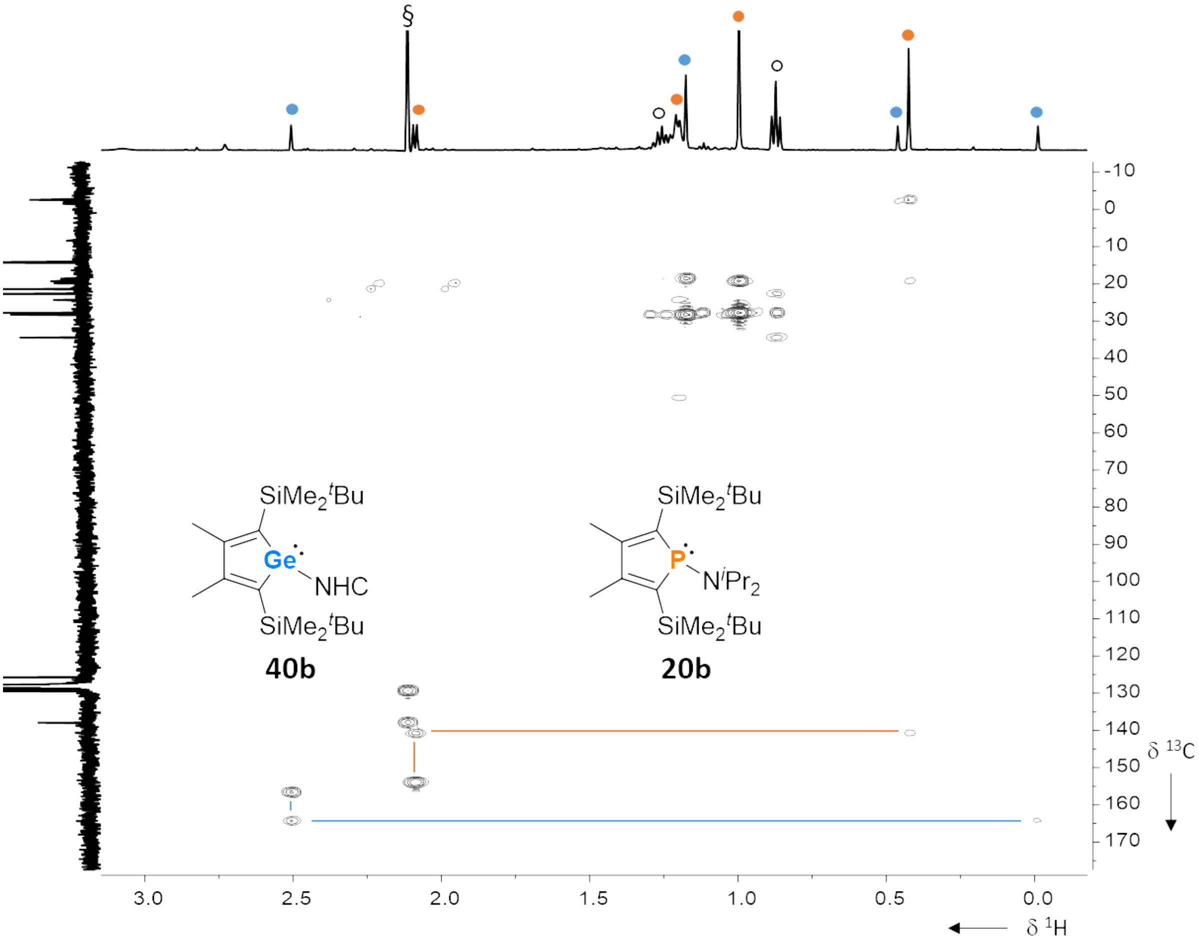

Figure S114. $^{1}H^{13}C$ HMBC NMR spectrum (500.1 MHz, 305 K, benzene-d_6) of the obtained mixture of NHC-stabilised germylene **40b** and phosphole **20b** (•) (°pentane, § toluene).

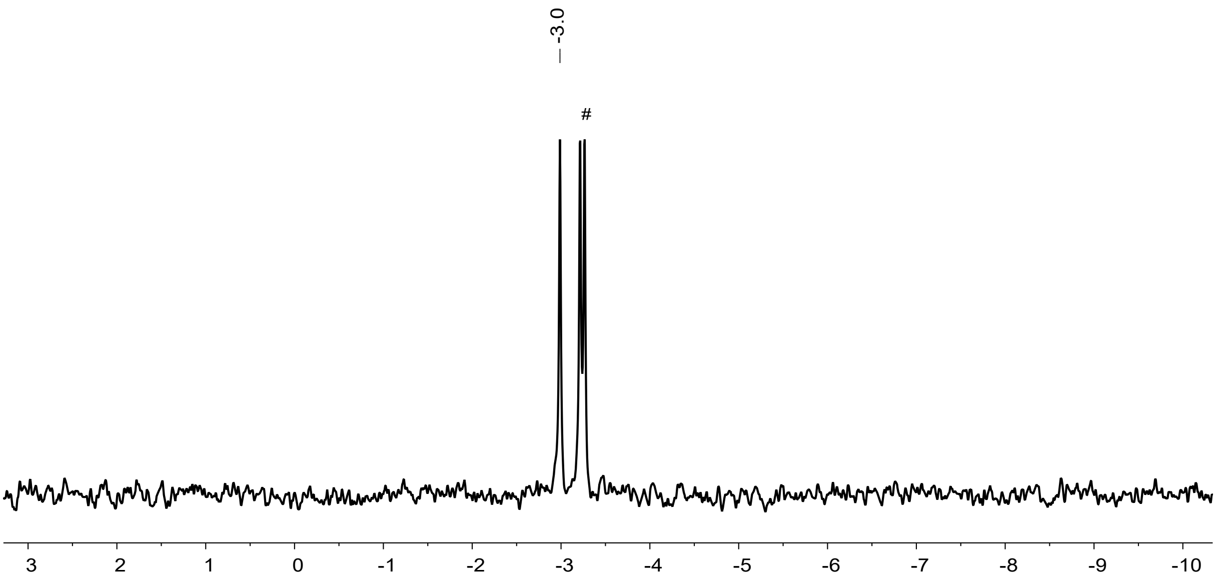

Figure S115. $^{29}Si\{^{1}H\}$ INEPT NMR spectrum (99.3 MHz, 305 K, benzene-d_6) of NHC-stabilised germylene **40b** (#phosphole **20b**).

Crystallographic Data

Single crystal X-ray data for **23a** and **[25a][GaCl$_4$]** were measured on a Bruker AXS Apex II diffractometer (graphite monochromator, Mo-Kα radiation, λ = 0.71073 Å, Kappa 4-circle goniometer, Apex II CCD detector). Single crystal data for **15a**, **21a**, **[25b][SbF$_6$]**, **34a** and **40b** were measured on a Bruker AXS D8 Venture diffractometer (multilayer optics, Mo-Kα with λ = 0.71073 Å, Kappa 4-circle goniometer, Photon III C14 CPAD detector). All crystals were measured at a temperature of 100 K. Absorption corrections using equivalent reflections were performed with the program SADABS.[1] All structures were solved with the program SHELXS[2] and refined with SHELXL[3] using the OLEX2[4] GUI.

All non H atoms were refined using anisotropic atomic displacement parameters (ADPs). The minor sites of the disordered piperidine ligand in **23a** were refined using isotropic ADPs. H atoms bonded to C were located in the difference Fourier maps and placed on idealized geometric positions with idealized atomic displacement parameters using the riding model. H atoms bonded to P were refined freely.

The crystallographic data can be obtained free of charge from https://www.ccdc.cam.ac.uk/structures/ quoting the CCDC numbers 2363314-2363320.

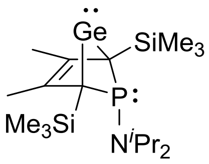

15a

CCDC code	2363319	
Empirical formula	C18 H38 Ge N P Si2	
Formula weight	428.23	
Temperature	100(2) K	
Wavelength	0.71073 Å	
Crystal system	Orthorhombic	
Space group	Pnma	
Unit cell dimensions	a = 12.4246(9) Å	α = 90°.
	b = 18.0809(13) Å	β = 90°.
	c = 10.2515(7) Å	γ = 90°.
Volume	2303.0(3) $Å^3$	
Z	4	
Density (calculated)	1.235 Mg/m^3	
Absorption coefficient	1.504 mm^{-1}	
F(000)	912	
Crystal size	0.160 x 0.160 x 0.090 mm^3	
Theta range for data collection	2.253 to 40.249°	
Index ranges	-22<=h<=22, -32<=k<=32, -18<=l<=18	
Reflections collected	228190	
Independent reflections	7412 (R(int) = 0.0301)	
Observed reflections (I > 2(I))	6932	
Completeness to theta = 40.249°	100.0 %	
Absorption correction	Semi-empirical from equivalents	
Max. and min. transmission	1.0000 and 0.9007	
Refinement method	Full-matrix least-squares on F^2	
Data / restraints / parameters	7412 / 0 / 118	
Goodness-of-fit on F^2	1.076	
Final R indices (I>2sigma(I))	R1 = 0.0173, wR2 = 0.0498	
R indices (all data)	R1 = 0.0191, wR2 = 0.0505	
Extinction coefficient	n/a	
Largest diff. peak and hole	0.567 and -0.340 e.$Å^{-3}$	

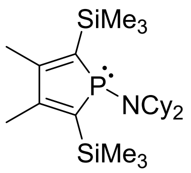

21a

CCDC code	2363317	
Empirical formula	C24 H46 N P Si2	
Formula weight	435.77	
Temperature	100(2) K	
Wavelength	0.71073 Å	
Crystal system	Triclinic	
Space group	P-1	
Unit cell dimensions	a = 9.8664(4) Å	α = 83.1135(16)°.
	b = 10.8301(5) Å	β = 86.3992(16)°.
	c = 13.2382(5) Å	γ = 69.5125(16)°.
Volume	1315.19(10) Å^3	
Z	2	
Density (calculated)	1.100 Mg/m^3	
Absorption coefficient	0.206 mm^{-1}	
F(000)	480	
Crystal size	0.090 x 0.070 x 0.040 mm^3	
Theta range for data collection	1.550 to 33.726°	
Index ranges	-15<=h<=15, -16<=k<=16, -20<=l<=20	
Reflections collected	121480	
Independent reflections	10513 (R(int) = 0.0464)	
Observed reflections (I > 2(I))	9104	
Completeness to theta = 33.726°	100.0 %	
Absorption correction	Semi-empirical from equivalents	
Max. and min. transmission	1.0000 and 0.9609	
Refinement method	Full-matrix least-squares on F^2	
Data / restraints / parameters	10513 / 0 / 261	
Goodness-of-fit on F^2	1.059	
Final R indices (I>2sigma(I))	R1 = 0.0345, wR2 = 0.0824	
R indices (all data)	R1 = 0.0428, wR2 = 0.0862	
Extinction coefficient	n/a	
Largest diff. peak and hole	0.481 and -0.231 e.Å^{-3}	

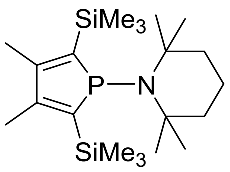

23a

CCDC code	2363314	
Empirical formula	C21 H42 N P Si2	
Formula weight	395.70	
Temperature	100(2) K	
Wavelength	0.71073 Å	
Crystal system	Monoclinic	
Space group	$P2_1/c$	
Unit cell dimensions	a = 12.7167(3) Å	α = 90°.
	b = 30.5644(8) Å	β = 90.0130(13)°.
	c = 12.3609(3) Å	γ = 90°.
Volume	4804.4(2) $Å^3$	
Z	8	
Density (calculated)	1.094 Mg/m^3	
Absorption coefficient	0.219 mm^{-1}	
F(000)	1744	
Crystal size	0.300 x 0.260 x 0.200 mm^3	
Theta range for data collection	1.601 to 34.971°	
Index ranges	-20<=h<=19, -48<=k<=49, -19<=l<=19	
Reflections collected	125982	
Independent reflections	20288 (R(int) = 0.0392)	
Observed reflections (I > 2(I))	17938	
Completeness to theta = 34.971°	96.1 %	
Absorption correction	Semi-empirical from equivalents	
Max. and min. transmission	1.0000 and 0.9623	
Refinement method	Full-matrix least-squares on F^2	
Data / restraints / parameters	20288 / 0 / 556	
Goodness-of-fit on F^2	1.040	
Final R indices (I>2sigma(I))	R1 = 0.0490, wR2 = 0.1227	
R indices (all data)	R1 = 0.0577, wR2 = 0.1277	
Extinction coefficient	n/a	
Largest diff. peak and hole	1.062 and -0.471 e.$Å^{-3}$	

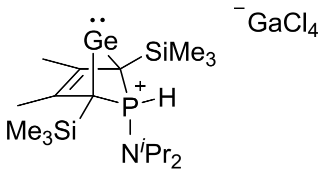

[25a][GaCl$_4$]

CCDC code	2363315	
Empirical formula	C18 H39 Cl4 Ga Ge N P Si2	
Formula weight	640.76	
Temperature	100(2) K	
Wavelength	0.71073 Å	
Crystal system	Monoclinic	
Space group	P2$_1$/n	
Unit cell dimensions	a = 15.9762(10) Å	α = 90°.
	b = 12.1459(8) Å	β = 111.3991(12)°.
	c = 16.1325(10) Å	γ = 90°.
Volume	2914.6(3) Å^3	
Z	4	
Density (calculated)	1.460 Mg/m^3	
Absorption coefficient	2.467 mm^{-1}	
F(000)	1312	
Crystal size	0.360 x 0.260 x 0.220 mm^3	
Theta range for data collection	1.535 to 34.971°	
Index ranges	-25<=h<=25, -19<=k<=19, -25<=l<=26	
Reflections collected	145900	
Independent reflections	12810 (R(int) = 0.0358)	
Observed reflections (I > 2(I))	10999	
Completeness to theta = 34.971°	100.0 %	
Absorption correction	Numerical	
Max. and min. transmission	0.7002 and 0.4994	
Refinement method	Full-matrix least-squares on F^2	
Data / restraints / parameters	12810 / 0 / 269	
Goodness-of-fit on F^2	1.020	
Final R indices (I>2sigma(I))	R1 = 0.0221, wR2 = 0.0546	
R indices (all data)	R1 = 0.0298, wR2 = 0.0579	
Extinction coefficient	n/a	
Largest diff. peak and hole	1.035 and -0.645 e.Å^{-3}	

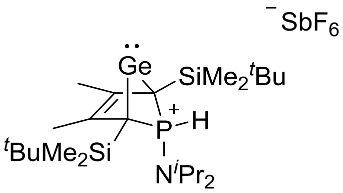

[25b][SbF$_6$]

CCDC code	2363318	
Empirical formula	C24 H51 F6 Ge N P Sb Si2	
Formula weight	749.14	
Temperature	100(2) K	
Wavelength	0.71073 Å	
Crystal system	Monoclinic	
Space group	$P2_1/c$	
Unit cell dimensions	a = 12.0024(9) Å	α = 90°.
	b = 11.8358(9) Å	β = 99.704(3)°.
	c = 23.4871(18) Å	γ = 90°.
Volume	3288.8(4) Å^3	
Z	4	
Density (calculated)	1.513 Mg/m^3	
Absorption coefficient	1.905 mm^{-1}	
F(000)	1528	
Crystal size	0.120 x 0.120 x 0.090 mm^3	
Theta range for data collection	1.721 to 40.249°	
Index ranges	-21<=h<=21, -21<=k<=21, -42<=l<=42	
Reflections collected	217854	
Independent reflections	20716 (R(int) = 0.0509)	
Observed reflections (I > 2(I))	17712	
Completeness to theta = 40.249°	100.0 %	
Absorption correction	Semi-empirical from equivalents	
Max. and min. transmission	1.0000 and 0.9017	
Refinement method	Full-matrix least-squares on F^2	
Data / restraints / parameters	20716 / 0 / 409	
Goodness-of-fit on F^2	1.033	
Final R indices (I>2sigma(I))	R1 = 0.0328, wR2 = 0.0864	
R indices (all data)	R1 = 0.0404, wR2 = 0.0902	
Extinction coefficient	n/a	
Largest diff. peak and hole	2.893 and -1.525 e.Å^{-3}	

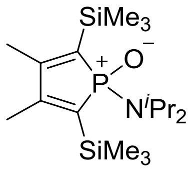

34a

CCDC code	2363316	
Empirical formula	C18 H38 N O P Si2	
Formula weight	371.64	
Temperature	100(2) K	
Wavelength	0.71073 Å	
Crystal system	Monoclinic	
Space group	$P2_1/n$	
Unit cell dimensions	a = 10.2065(3) Å	α = 90°.
	b = 19.2905(5) Å	β = 96.0698(10)°.
	c = 11.5732(3) Å	γ = 90°.
Volume	2265.86(11) $Å^3$	
Z	4	
Density (calculated)	1.089 Mg/m^3	
Absorption coefficient	0.232 mm^{-1}	
F(000)	816	
Crystal size	0.130 x 0.110 x 0.060 mm^3	
Theta range for data collection	2.061 to 36.317°	
Index ranges	-17<=h<=14, -32<=k<=32, -19<=l<=19	
Reflections collected	138771	
Independent reflections	10977 (R(int) = 0.0395)	
Observed reflections (I > 2(I))	9886	
Completeness to theta = 36.317°	100.0 %	
Absorption correction	Semi-empirical from equivalents	
Max. and min. transmission	1.0000 and 0.9489	
Refinement method	Full-matrix least-squares on F^2	
Data / restraints / parameters	10977 / 0 / 240	
Goodness-of-fit on F^2	1.082	
Final R indices (I>2sigma(I))	R1 = 0.0364, wR2 = 0.0901	
R indices (all data)	R1 = 0.0418, wR2 = 0.0933	
Extinction coefficient	n/a	
Largest diff. peak and hole	0.588 and -0.580 e.$Å^{-3}$	

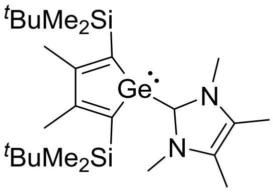

40b

CCDC code	2363320	
Empirical formula	C25 H48 Ge N2 Si2	
Formula weight	505.42	
Temperature	100(2) K	
Wavelength	0.71073 Å	
Crystal system	Monoclinic	
Space group	$P2_1/n$	
Unit cell dimensions	a = 11.0704(3) Å	α = 90°.
	b = 12.4659(3) Å	β = 101.0413(11)°.
	c = 21.0500(6) Å	γ = 90°.
Volume	2851.18(13) $Å^3$	
Z	4	
Density (calculated)	1.177 Mg/m^3	
Absorption coefficient	1.172 mm^{-1}	
F(000)	1088	
Crystal size	0.170 x 0.120 x 0.120 mm^3	
Theta range for data collection	1.908 to 40.248°	
Index ranges	-20<=h<=20, -22<=k<=22, -38<=l<=38	
Reflections collected	218320	
Independent reflections	17959 (R(int) = 0.0338)	
Observed reflections (I > 2(I))	15933	
Completeness to theta = 40.248°	100.0 %	
Absorption correction	Semi-empirical from equivalents	
Max. and min. transmission	1.0000 and 0.9182	
Refinement method	Full-matrix least-squares on F^2	
Data / restraints / parameters	17959 / 0 / 287	
Goodness-of-fit on F^2	1.057	
Final R indices (I>2sigma(I))	R1 = 0.0209, wR2 = 0.0548	
R indices (all data)	R1 = 0.0268, wR2 = 0.0573	
Extinction coefficient	n/a	
Largest diff. peak and hole	0.538 and -0.565 e.$Å^{-3}$	

Computational Details

All quantum chemical calculations were carried out using the Gaussian16 package.[11] The natural bond orbital (NBO)[12] analysis was done with the program NBO 7.0.[13] For the topological analysis of the calculated electron density[14] the AIMAll program was used.[15]

The molecular structure optimizations of all compounds that are relevant for the discussion were performed using the Minnesota 2006 with non-local exchange functional (M06-2X)[16] along with the 6-311+G(d,p) basis set for all elements except Rh (def2-tzvp). Every stationary point was identified by a subsequent frequency calculation either as minimum (Number of imaginary frequencies NIMAG = 0). The self-consistent field SCF energies, E(SCF), and the absolute computed Gibbs free energies, G^{298}, at T = 298 K and p = 0.101 MPa in the gas phase, for all optimized structures obtained with these methods are given in Table S3. All corresponding computed molecular structures are given in the xyz-files.

Table S3. Calculated self-consistent field SCF energies and absolute computed Gibbs free energies.

Compound	E(SCF) [a.u.]	G(298 K) [a.u.]
germolediide **[8a]**	-3127.55614	-3127.29139
germolediide **[8b]**	-3363.38575	-3362.95650
phospha-BCH-germylene **15a**	-3760.76027	-3760.30009
phospha-BCH-germylene **15b**	-3996.57319	-3995.94454
phospha-BCH-germylene **16a**	-3994.19581	-3993.60584
phospha-BCH-germylene **16b**	-4230.00910	-4229.25190
phospha-BCH-germylene **17a**	-3720.26093	-3719.84463
phospha-BCH-germylene **17b**	-3956.07705	-3955.49538
phospha-BCH-germylene **18a**	-3877.46387	-3876.93696
aminophosphole **19a**	-1526.54959	-1526.19812
aminophosphole **20a**	-1683.76499	-1683.29881
aminophosphole **20b**	-1919.58035	-1918.95264
aminophosphole **21a**	-1917.19553	-1916.60126
aminophosphole **21b**	-2153.01485	-2152.25617
aminophosphole **22a**	-1643.27125	-1642.85400
aminophosphole **22b**	-1879.09013	-1878.50713
aminophosphole **23a**	-1800.47812	-1799.94768
phosphonium-BCH-germylene **[25a]$^+$**	-3761.15799	-3760.68449
model phosphonium-BCH-germylene **[25(M)]$^+$**	-2708.01459	-2707.88980
ammonium ion **[27a]$^+$**	-3761.17013	-3760.69792
germylium ion **[29a]$^+$**	-3761.10801	-3760.63962
carbenium ion **[28a]$^+$**	-3761.12028	-3760.64639
model phospha-BCH-germylene **15(M)**	-2707.63815	-2707.52288
model phospha-BCH-germylene **15-H-A**	-2708.81591	-2708.67813
model phospha-BCH-germylene **15-H-B**	-2710.02362	-2709.86845
model sila-BCH-germylene **24**	-2601.06524	-2600.95910
model sila-BCH-germylene **24-H-A**	-2602.24714	-2602.11854
model sila-BCH-germylene **24-H-B**	-2603.45928	-2603.31307
germylene Rh complex **32a**	-4643.50920	-4642.86867
germylene Rh complex **33a**	-4643.50584	-4642.87728

Structural Data

Tables S4 and S5 display selected structural data of the x-ray structures, the calculated structures and the calculated model compounds of germylene **15a** and phosphonium germylene **[25a]**$^+$. The data is in good agreement.

Table S4. Comparison of experimental (from XRD analysis) and computed structural parameters (M06-2X/6-311+G(d,p) of phospha-BCH-germylene **15a**.

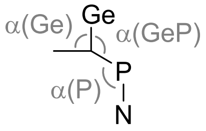	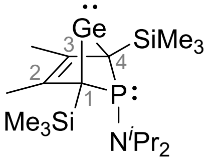 experimental	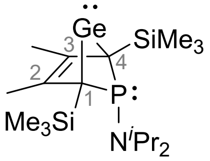 calculated	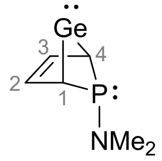 calculated (model)
C^1–C^2	146.53(6)	146.2	144.7
C^2–C^3	142.33(8)	142.0	141.1
Ge–C^1	216.32(4)	216.3	216.4
Ge–C^2	219.64(5)	220.6	219.4
P–C^1	184.11(4)	186.0	184.0
P–N	170.18(7)	172.6	170.8
Ge–P	280.29(3)	281.8	283.9
α(Ge)	82.012(7)	82.0	81.2
α(GeP)	127.121(12)	126.9	125.9
α(P)	114.914(23)	114.4	115.0

Table S5. Comparison of experimental (from XRD analysis) and computed structural parameters (M06-2X/6-311+G(d,p) of phosphonium-BCH-germylene **[25a]**$^+$.

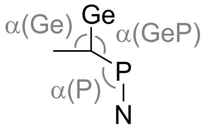	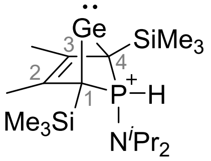 experimental	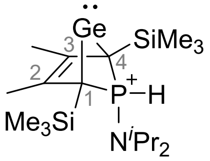 calculated	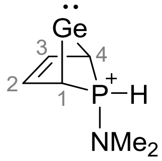 calculated (model)
C^1–C^2	147.69(18)	146.7	144.8
C^2–C^3	141.40(17)	142.1	140.6
Ge–C^1	220.51(13)	219.7	221.1
Ge–C^2	222.41(12)	221.1	221.2
P–C^1	177.91(14)	178.2	177.7
P–N	163.98(13)	166.1	163.8
P–Ge	276.11(5)	276.8	281.5
α(Ge)	81.919(11)	81.8	80.9
α(GeP)	129.648(12)	130.2	128.7
α(P)	135.863(47)	135.9	137.1

Electronic Structure of Bicyclic Germylenes 15(M) and [25(M)]+

The principal shape of the important molecular orbitals of the protonated bicyclic germylene [**25**(**M**)]+ differs only slightly from that of the germylene **15**(**M**) (see Figure S116). This indicate the general electronic structure of the germylene. As an expected result of the protonation the energy of all orbitals is significantly lowered.

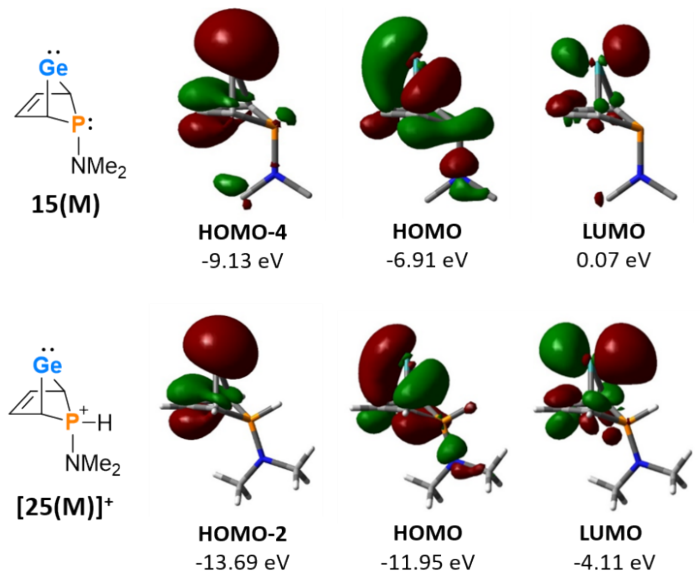

Figure S116. Shape and energies of selected molecular orbitals of phospha BCH germylene **15**(**M**) and of phosphonium BCH germylene [**25**(**M**)]+ (M06-2X/6-311+G(d,p), isodensity value 0.04).

^{31}P NMR Spectroscopic Data

NMR chemical shift computations were performed using the GIAO method as implemented in Gaussian16 and the M06-L functional[16] along with the 6-311+G(2d,p) basis set for molecular structures obtained at the M06-2X/6-311+G(d,p) level of theory. The calculated shifts were referenced against PH_3 (85% H_3PO_4 in H_2O): δ $^{31}P(PH_3)$ = -239.1 (M06-L/6-311+G(2d,p)//M06-2X/6-311+G(d,p)). Previously, we have shown that the M06-L functional provides very accurate estimates of carbon, aluminum and silicon NMR chemical shieldings.[17-18]

Due to the structural similarity of the X-ray structure and the calculated structure of germylene **15a** and phosphonium germylene **[25a]**$^+$, the conformity of the experimental and calculated ^{31}P NMR chemical shift was examined, too. The goal was to evaluate the predictability of the ^{31}P NMR chemical shift of the phospha-BCH-germylenes, the phospholes (Table S6 and Figure S117) as well as possible products of the reactivity studies.

Table S6. Overview of the experimental (measured in benzene-d_6) and calculated ^{31}P NMR chemical shifts.

**Germylenes**		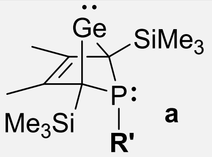	a		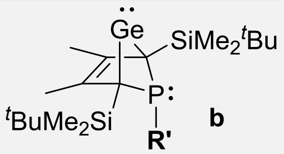	b	
	**R’**	**δ $^{31}P_{exp}$**	***δ $^{31}P_{calc}$***	**$\Delta(\delta$ $^{31}P)$**	**δ $^{31}P_{exp}$**	***δ $^{31}P_{calc}$***	**$\Delta(\delta$ $^{31}P)$**
**15**	N^iPr_2	35.4	*31*	4	53.8	*48*	6
**16**	NCy_2	39.4	*37*	2	57.7	*50*	8
**17**	Pip	-	*50*	-	59.8	*50*	10
**18**	TMP	30.8	*28*	3	39.7	-	-
**Phospholes**		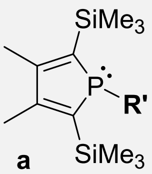	a		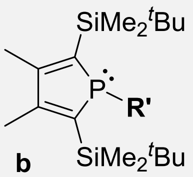	b	
	**R’**	**δ $^{31}P_{exp}$**	***δ $^{31}P_{calc}$***	**$\Delta(\delta$ $^{31}P)$**	**δ $^{31}P_{exp}$**	***δ $^{31}P_{calc}$***	**$\Delta(\delta$ $^{31}P)$**
**19**	NMe_2	94.3	*88*	6	98.5	-	-
**20**	N^iPr_2	65.6	*56*	10	71.7	*56*	16
**21**	NCy_2	63.8	*61*	3	69.7	*60*	10
**22**	Pip	92.2	*85*	7	95.9	*87*	9
**23**	TMP	54.2	*46*	8	61.2	-	-

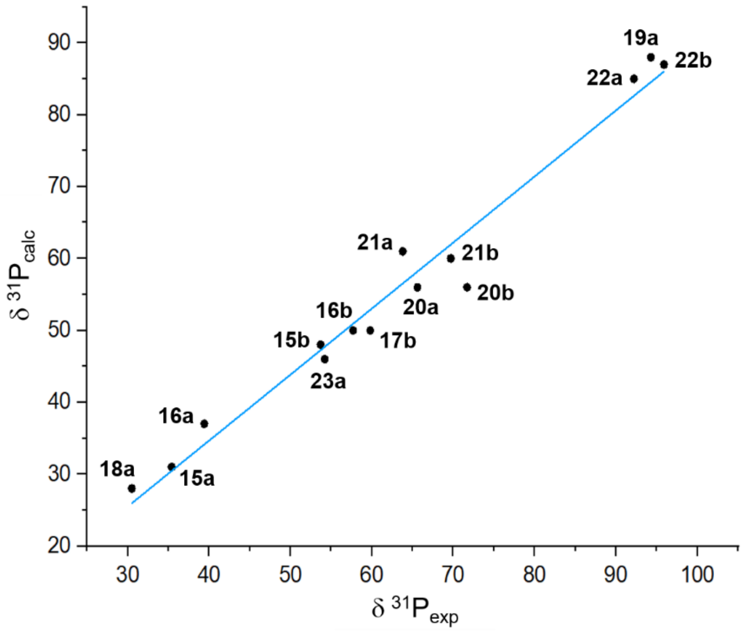

Figure S117. Correlation of experimental (x axis) and calculated (y axis) ^{31}P NMR chemical shifts of the aminophospha-BCH-germylenes **15-18** and aminophospholes **19-23**. Linear regression: $\delta\,^{31}P_{calc}$ **= (2.054 ± 2.94) + (0.918 ± 0.04)** $\delta\,^{31}P_{exp}$.

2nd Order Perturbation Analysis of the homoconjugation

Figure 118 summarises the results of the 2nd order perturbation analysis of BCHGe **15**(**M**).

On the top, the delocalization of electron density from the π(C2C3) NBO (occupation 1.65 e) into the 4p(Ge) NBO (occupation 0.30 e) is shown. This delocalization (2nd order perturbation energy E(2) = 4.80 eV) leads to the π(C2C3) NLMO with significant contributions from orbitals of the germanium atom (16%) (through space delocalization).

At the bottom, the delocalization of electron density from the σ(C1Ge) NBO (occupation 1.72 e) into the π*(C2C3) NBO (occupation 0.43 e) is shown. This delocalization (2nd order perturbation energy E(2) = 2.54 eV) leads to the σ(C1Ge) NLMO with significant contributions from orbitals of the carbon atoms C2 and C3 (10%) (σ/π* hyperconjugation).

In summary, the homoconjugation between the germanium atom and the remote C=C double bond can be described as donation from the π-orbital of the C2=C3 double bond into the empty 4pGe orbital and back donation form the σ–GeC1 and σ–GeC4 bonds into the π*-orbital of the C2=C3 bond.

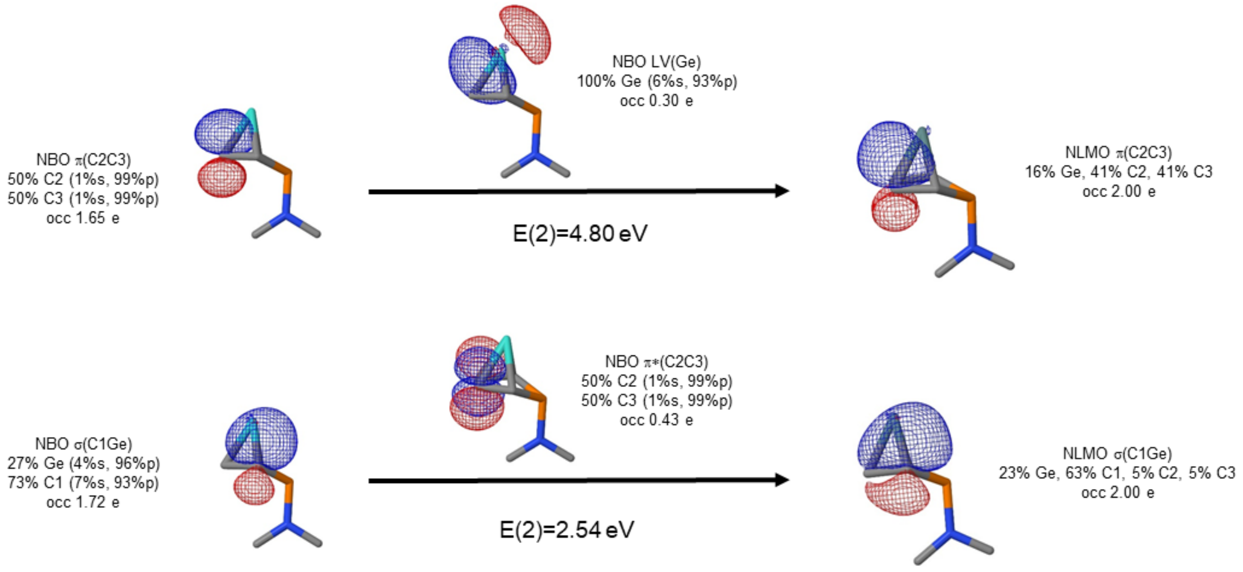

Figure S118. Results of the 2nd order perturbation analysis of BCH germylene **15(M)** indicating the through space electron donation from the π(C2C3) into the empty 4p orbital at Ge (LV(Ge))(top); and the σ/π* hyperconjugation between σ(Ge-C1) and π*(C2C3)(bottom) (at M06-2X/6-311+G(d,p), isodensity value 0.05).

Internal comparison of the steric demand of selected nucleophiles

To access the steric demand of the compounds, present in the reaction mixture, which can potentially initiate the germanium elimination, their steric demand was internally compared. Therefore, their buried volume (%V_{bur}) was calculated using the *SambVca 2.1* web program.[19] The optimised structures (M06-2X/6-311+G(d,p)) were uploaded to the website. The coordinating atom and the axes were defined on the interactive interface. Here, the sphere was set to a radius (R) of 350 pm. The ligand-metal distance (d) was determined to 210 pm. Mesh spacing for numerical integration was set to 0.05 and bond-radii were scaled to 1.17. Hydrogen atoms were included in the calculation. The calculated values can be found in Figure S119, ordered by ascending steric demand. In each case, the coordinating atom is highlighted in colour.

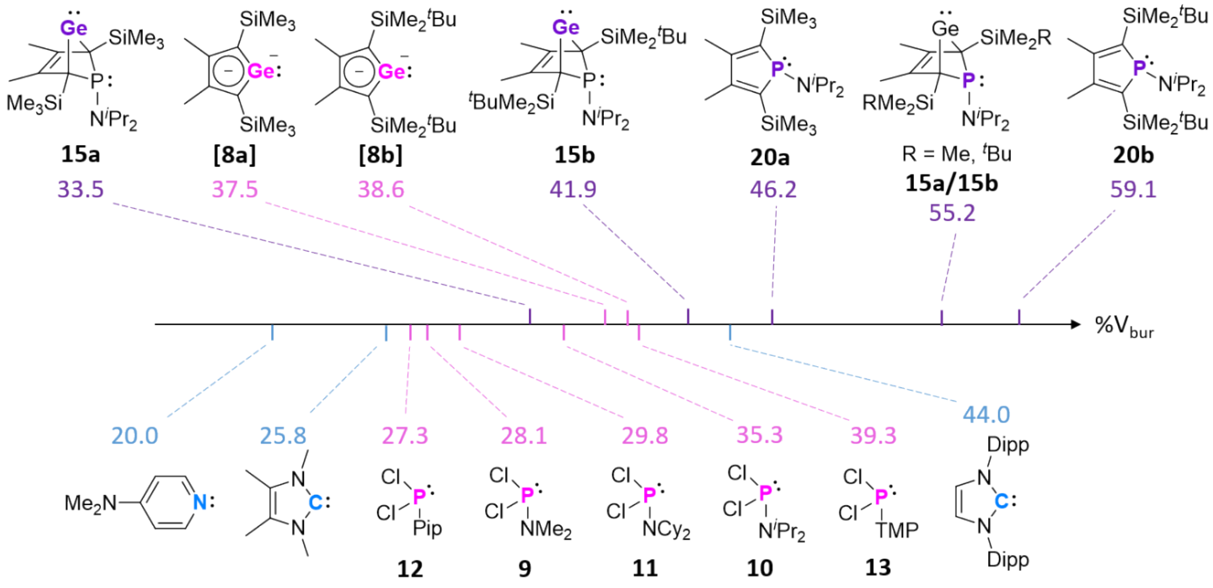

Figure S119. Calculated buried volume (%V_{bur}) of selected nucleophiles.

References

[1] Z. Dong, C. R. W. Reinhold, M. Schmidtmann, T. Müller, *Organometallics* **2018**, *37*, 4736-4743.

[2] C. Reinhold, *Dissertation*, Carl von Ossietzky Universität Oldenburg **2017**.

[3] M. Westerhause, B. Stein, M. W. Ossberger, H. Görls, J. C. G. Ruiz, H. Nöth, P. Mayer, *ARKIVOC* **2006**, *2007*, 46-59.

[4] G. Märkl, B. Alig, *J. Organomet. Chem.* **1984**, *273*, 1-29.

[5] R. B. King, N. D. Sadanani, *Synth. React. Inorg. Met.-Org. Chem.* **1985**, *15*, 149-153.

[6] S. Stadlbauer, R. Frank, I. Maulana, P. Lönnecke, B. Kirchner, E. Hey-Hawkins, *Inorg. Chem.* **2009**, *48*, 6072-6082.

[7] L. Krause, R. Herbst-Irmer, G. M. Sheldrick, D. Stalke, Journal of Applied Crystallography 2015, 48, 3-10.

[8] G. M. Sheldrick, Acta Crystallographica Section A 2008, 64, 112-122.

[9] G. M. Sheldrick, Acta Crystallographica Section C 2015, 71, 3-8.

[10] O. V. Dolomanov, L. J. Bourhis, R. J. Gildea, J. A. K. Howard, H. Puschmann, Journal of Applied Crystallography 2009, 42, 339-341.

[11] M. J. Frisch, G. W. Trucks, H. B. Schlegel, G. E. Scuseria, M. A. Robb, J. R. Cheeseman, G. Scalmani, V. Barone, G. A. Petersson, H. Nakatsuji, X. Li, M. Caricato, A. V. Marenich, J. Bloino, B. G. Janesko, R. Gomperts, B. Mennucci, H. P. Hratchian, J. V. Ortiz, A. F. Izmaylov, J. L. Sonnenberg, Williams, F. Ding, F. Lipparini, F. Egidi, J. Goings, B. Peng, A. Petrone, T. Henderson, D. Ranasinghe, V. G. Zakrzewski, J. Gao, N. Rega, G. Zheng, W. Liang, M. Hada, M. Ehara, K. Toyota, R. Fukuda, J. Hasegawa, M. Ishida, T. Nakajima, Y. Honda, O. Kitao, H. Nakai, T. Vreven, K. Throssell, J. A. Montgomery Jr., J. E. Peralta, F. Ogliaro, M. J. Bearpark, J. J. Heyd, E. N. Brothers, K. N. Kudin, V. N. Staroverov, T. A. Keith, R. Kobayashi, J. Normand, K. Raghavachari, A. P. Rendell, J. C. Burant, S. S. Iyengar, J. Tomasi, M. Cossi, J. M. Millam, M. Klene, C. Adamo, R. Cammi, J. W. Ochterski, R. L. Martin, K. Morokuma, O. Farkas, J. B. Foresman, D. J. Fox, Wallingford, CT, **2016**.

[12] A. E. Reed, L. A. Curtiss, F. Weinhold, *Chem. Rev.* **1988**, *88*, 899-926.

[13] E. D. Glendening, J. K. Badenhoop, A. E. Reed, J. E. Carpenter, J. A. Bohmann, C. M. Morales, P. Karafiloglu, C. R. Landis, F. Weinhold, Theoretical Chemistry Institute, University of Wisconsin, Madison, **2018**.

[14] R. F. W. Bader, *Atoms in Molecules: A Quantum Theory*; Clarendon Press: Oxford, New York, **1990**.

[15] T. A. Keith, *AIMALL*; TK Gristmill Software: Overland Park KS, **2017**.

[16] Y. Zhao, D. G. Truhlar, *Theor. Chem. Acc.* **2008**, *120*, 215-241.

[17] L. Albers, S. Rathjen, J. Baumgartner, C. Marschner, T. Müller, *J. Am. Chem. Soc.* **2016**, *138*, 6886-6892.

[18] L. Albers, P. Tholen, M. Schmidtmann, T. Müller, *Chem. Sci.* **2020**, *11*, 2982-2986.

[19] L. Falivene, Z. Cao, A. Petta, L. Serra, A. Paoter, R. Oliva, V. Scarano, L. Cavallo, *Nature Chemistry* **2019**, *11*, 872-879.

computed structures (xyz files).xyz
This file cannot be rendered in this PDF. Please download the source file.
